# Virus Genomes from Deep Sea Sediments Expand the Ocean Megavirome and Support Independent Origins of Viral Gigantism

**DOI:** 10.1128/mBio.02497-18

**Published:** 2019-03-05

**Authors:** Disa Bäckström, Natalya Yutin, Steffen L. Jørgensen, Jennah Dharamshi, Felix Homa, Katarzyna Zaremba-Niedwiedzka, Anja Spang, Yuri I. Wolf, Eugene V. Koonin, Thijs J. G. Ettema

**Affiliations:** a Department of Cell and Molecular Biology, Science for Life Laboratory, Uppsala University, Uppsala, Sweden; b National Center for Biotechnology Information, National Library of Medicine, National Institutes of Health, Bethesda, Maryland, USA; c Department of Biology, Centre for Geobiology, University of Bergen, Bergen, Norway; d Department of Marine Microbiology and Biogeochemistry, NIOZ, Royal Netherlands Institute for Sea Research, Yerseke, The Netherlands; e Utrecht University, Den Burg, The Netherlands; Skirball Institute of Biomolecular Medicine, New York University Medical Center; Max Planck Institute; UFMG

**Keywords:** giant viruses, nucleocytoplasmic large DNA viruses, deep sea sediments, metagenomics, virus evolution

## Abstract

The nucleocytoplasmic large DNA viruses (NCLDV) of eukaryotes (proposed order, “Megavirales”) include the families *Poxviridae*, *Asfarviridae*, *Iridoviridae*, *Ascoviridae*, *Phycodnaviridae*, *Marseilleviridae*, and *Mimiviridae*, as well as still unclassified pithoviruses, pandoraviruses, molliviruses, and faustoviruses. Several of these virus groups include giant viruses, with genome and particle sizes exceeding those of many bacterial and archaeal cells. We explored the diversity of the NCLDV in deep sea sediments from the Loki’s Castle hydrothermal vent area. Using metagenomics, we reconstructed 23 high-quality genomic bins of novel NCLDV, 15 of which are related to pithoviruses, 5 to marseilleviruses, 1 to iridoviruses, and 2 to klosneuviruses. Some of the identified pithovirus-like and marseillevirus-like genomes belong to deep branches in the phylogenetic tree of core NCLDV genes, substantially expanding the diversity and phylogenetic depth of the respective groups. The discovered viruses, including putative giant members of the family *Marseilleviridae*, have a broad range of apparent genome sizes, in agreement with the multiple, independent origins of gigantism in different branches of the NCLDV. Phylogenomic analysis reaffirms the monophyly of the pithovirus-iridovirus-marseillevirus branch of the NCLDV. Similarly to other giant viruses, the pithovirus-like viruses from Loki’s Castle encode translation systems components. Phylogenetic analysis of these genes indicates a greater bacterial contribution than had been detected previously. Genome comparison suggests extensive gene exchange between members of the pithovirus-like viruses and *Mimiviridae*. Further exploration of the genomic diversity of Megavirales in additional sediment samples is expected to yield new insights into the evolution of giant viruses and the composition of the ocean megavirome.

## Abstract (precis)

Genomics and evolution of giant viruses are two of the most vigorously developing areas of virus research. Lately, metagenomics has become the main source of new virus genomes. Here we describe a metagenomic analysis of the genomes of large and giant viruses from deep sea sediments. The assembled new virus genomes substantially expand the known diversity of the nucleocytoplasmic large DNA viruses of eukaryotes. The results support the concept of independent evolution of giant viruses from smaller ancestors in different virus branches.

## INTRODUCTION

The nucleocytoplasmic large DNA viruses (NCLDV) comprise an expansive group of viruses that infect diverse eukaryotes (1). Most of the NCLDV share the defining biological feature of reproducing (primarily) in the cytoplasm of the infected cells as well as several genes encoding proteins involved in the key roles in virus morphogenesis and replication, leading to the conclusion that the NCLDV are monophyletic, that is, evolved from a single ancestral virus (2, 3). As originally defined in 2001, the NCLDV included 5 families of viruses: *Poxviridae*, *Asfarviridae*, *Iridoviridae*, *Ascoviridae*, and *Phycodnaviridae* (2). Subsequent isolation of viruses from protists has resulted in the stunning discovery of giant viruses, with genome sizes exceeding those of many bacteria and archaea (4–8). The originally discovered group of giant viruses forms the family *Mimiviridae* (9–13). Subsequently, 3 additional other groups of giant viruses have been identified, namely, pandoraviruses (14–16), pithoviruses, cedratviruses, orpheoviruses (here, the latter 3 groups of related viruses are collectively referred to as the putative family “Pithoviridae”) (17–19), and *Mollivirus sibericum* (20), along with two new groups of NCLDV with genomes of moderate size, the family *Marseilleviridae* (21, 22) and the faustoviruses (23, 24). Most of the NCLDV have icosahedral virions composed of a double-jelly-roll major capsid protein(s) (MCP), but poxviruses have distinct brick-shaped virions, ascoviruses have ovoid virions, molliviruses have a spherical virion, and, finally, pandoraviruses and pithoviruses have unusual, amphora-shaped virions. The pithovirus virions are the largest among the currently known viruses. Several of the recently discovered groups of NCLDV, in particular, the putative family “Pithoviridae” (25), are likely to eventually become new families, and reclassification of the NCLDV into a new virus order, “Megavirales,” has been proposed (26, 27).

Phylogenomic reconstruction of gene gain and loss events resulted in mapping about 50 of the genes that are responsible for the key viral functions to the putative last common ancestor of the NCLDV. The existence of this large common gene contingent strongly supports the idea of the monophyly of the NCLDV despite the fact that their genome sizes differ by more than an order of magnitude and that their virions demonstrate remarkable morphological diversity (1, 3, 28–31). However, detailed phylogenetic analysis of the core genes of the NCLDV has revealed considerable evolutionary complexity, including numerous cases of displacement of ancestral genes with homologs from other sources and even some cases of independent capture of homologous genes (32). The genomes of the NCLDV encompass about 100 (some iridoviruses) to nearly 2,500 genes (pandoraviruses), including, in addition to the 50 or so core genes, numerous genes involved in various aspects of virus-host interaction, in particular, suppression of the host defense mechanisms, as well as many genes for which no function could be identified (1, 33).

The NCLDV include some viruses that are agents of devastating human and animal diseases, such as smallpox virus or African swine fever virus (34, 35), as well as viruses that infect algae and other planktonic protists and are important ecological agents (12, 36–38). Additionally, NCLDV elicit the strong interest of many researchers due to their large genome size, which, in the case of the giant viruses, falls within the range of typical genome sizes of bacteria and archaea. This apparent exceptional position of the giant viruses in the virosphere, together with the fact that they encode multiple proteins that are universal among cellular organisms (in particular, translation system components), has led to the devising of provocative scenarios of the origin and evolution of giant viruses. It has been proposed that the giant viruses were descendants of a hypothetical, probably extinct fourth domain of cellular life that evolved via drastic genome reduction, and support of this scenario has been claimed from phylogenetic analysis of aminoacyl-tRNA synthetases (aaRS) encoded by giant viruses (5, 26, 39–43). However, even apart from the conceptual difficulties inherent in the postulated cell-to-virus transition (44, 45), phylogenetic analysis of expanded sets of translation-related proteins encoded by giant viruses has resulted in tree topologies that were poorly compatible with the fourth domain hypothesis but that instead suggest piecemeal acquisition of these genes, likely from different eukaryotic hosts (29, 30, 46–49).

More generally, probabilistic reconstruction of gene gains and losses during the evolution of the NCLDV has revealed a highly dynamic evolutionary regime (3, 28, 30, 32, 48) that has been conceptualized in the so-called “genomic accordion” model, according to which virus evolution proceeds via alternating phases of extensive gene capture and gene loss (50, 51). In particular, in the course of the NCLDV evolution, giant viruses appear to have evolved from smaller ones on multiple, independent occasions (29, 30, 52).

In recent years, metagenomics has become the principal route of new virus discovery (53–55). However, in the case of giant viruses, *Acanthamoeba* coculturing has remained the main source of new virus identification, and this methodology has been refined to allow high-throughput giant virus isolation (56, 57). To date, over 150 species of giant viruses have been isolated from various environments, including water towers, soil, sewage, rivers, fountains, seawater, and marine sediments (58). The true diversity of giant viruses is difficult to assess, but the explosion of giant virus discovery during the last 10 years and data from large-scale metagenomic screens of viral diversity indicate that a major part of the virome of Earth remains unexplored (59). The core genes of the NCLDV can serve as bait for screening environmental sequences, and pipelines have been developed for large-scale screening of metagenomes (58, 60). Although these efforts have given indications of the presence of uncharacterized giant viruses in samples from various environments, few of these putative novel viruses can be characterized due to the lack of genomic information. Furthermore, giant viruses tend to be overlooked in viral metagenomic studies since samples are typically filtered according to the preconception of typical virion sizes (52).

To gain further insight into the ecology, evolution, and genomic content of giant viruses, it is necessary to retrieve more genomes rather than simply establish their presence by detection of single marker genes. Metagenomic binning is the process of clustering environmental sequences that belong to the same genome, based on features such as base composition and coverage. Binning has previously been used to reconstruct the genomes of large groups of uncharacterized bacteria and archaea in a culture-independent approach (61, 62). Only one case of binning has been reported for NCLDV, when the genomes of the klosneuviruses, distant relatives of the mimiviruses, were reconstructed from a simple wastewater sludge metagenome (48). More-complex metagenomes from all types of environments remain to be explored. However, standard methods for screening and binning of NCLDV have not yet been developed, and sequences of these viruses can be difficult to classify because of the occurrence of substantial horizontal gene transfer from bacteria and eukaryotes (13, 32, 46, 52) and also because a large proportion of the NCLDV genes (known as ORFans [open reading frames [ORFs] with no detectable homology to other ORFs in a database]) have no detectable homologs (25, 33).

We identified NCLDV sequences in deep sea sediment metagenomes from Loki’s Castle, a sample site that has been previously shown to be rich in uncharacterized prokaryotes (63, 64) (J. E. Dharamshi, D. Tamarit, L. Eme, C. Stairs, J. Martijn, F. Homa, S. L. Jørgensen, A. Spang, T. J. G. Ettema, submitted for publication). The complexity of the data and genomes required a combination of different binning methods, assembly improvement by reads profiling, and manual refinement of each bin to minimize contamination from nonviral sequences. As a result, 23 high-quality genomic bins of novel NCLDV were reconstructed, including (mostly) distant relatives of “*Pithoviridae*,” *Orpheovirus*, and *Marseilleviridae*, as well as two relatives of klosneuviruses. These findings substantially expand the diversity of the NCLDV, in particular, the pithovirus-iridovirus-marseillevirus (PIM) branch, further supporting the scenario of independent evolution of giant viruses from smaller ones in different branches of the NCLDV, and provide an initial characterization of the ocean megavirome.

## RESULTS

### Putative NCLDV in the Loki’s Castle metagenome.

**FIG 1:**
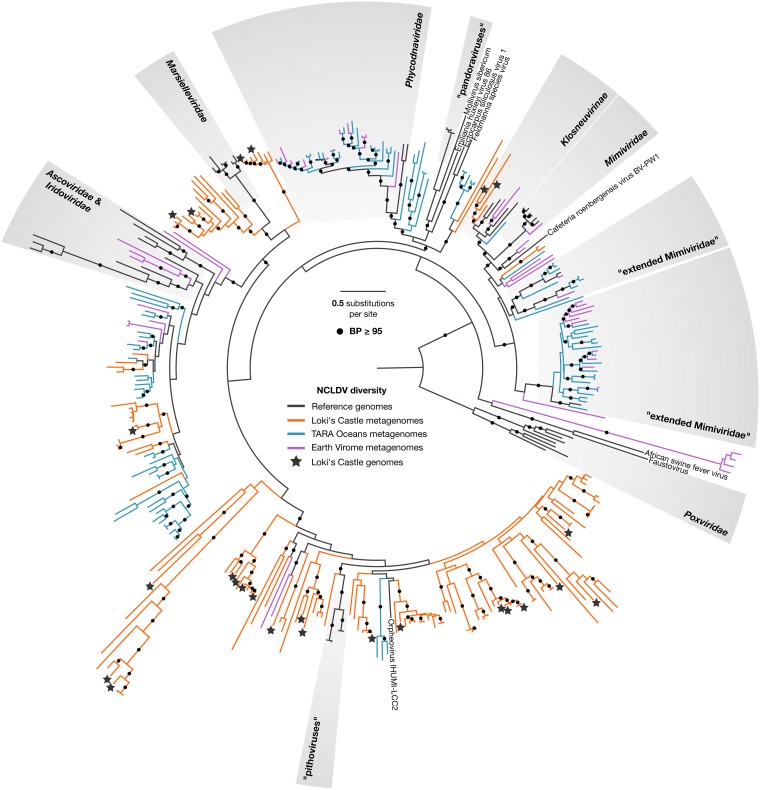
Diversity of the NCLDV DNAP sequences in the Loki’s Castle sediment metagenomes (orange) and in the Tara Oceans (turquoise) and EarthVirome (purple) databases. Reference sequences are shown in black. The binned NCLDV genomes are marked with a star. Branches with bootstrap values above 95 are marked with a black circle. The maximum likelihood phylogeny was constructed as described in Materials and Methods.

**FIG 2:**
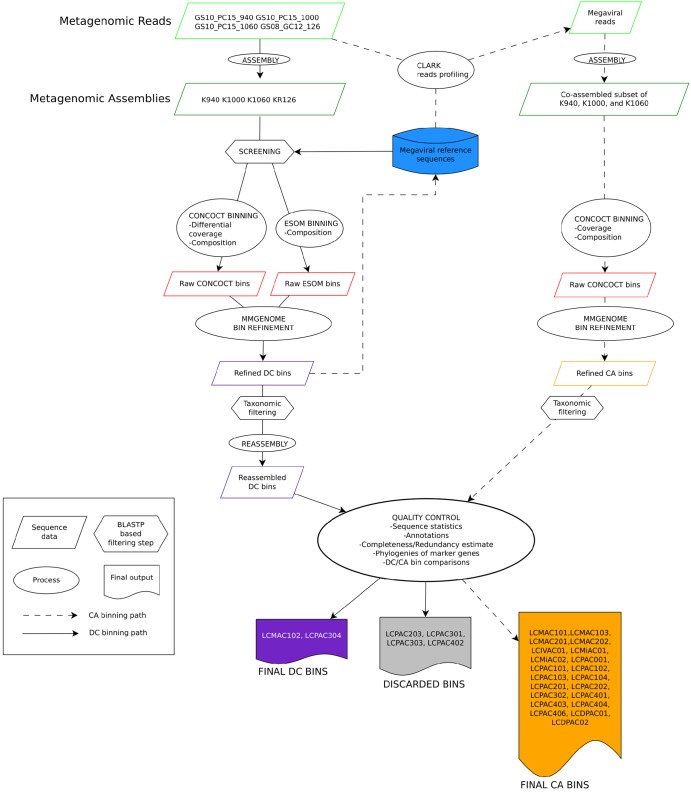
Flowchart of the metagenomic binning procedures. Two main binning approaches were used: differential coverage (DC) binning and coassembly (CA) binning. For DC binning, reads from four different samples were assembled into four metagenomes. The metagenomes were screened for NCLDV DNAP, and contigs were binned with CONCOCT and ESOM. The raw CONCOCT and ESOM bins were combined and refined using mmgenome. The refined bins were put through taxonomic filtering, keeping only the contigs encoding at least one NCLDV gene, and were finally reassembled. For CA binning, a database containing the refined DC bins and NCLDV reference genomes was used to create profiles to extract reads from the metagenomes. The reads were combined and coassembled. This step was followed by CONCOCT binning, mmgenome bin refinement, and taxonomic filtering. Finally, the DC bins and CA bins were annotated and the best bins were chosen by comparing sequence statistics, completeness and redundancy of marker genes, and marker gene phylogenies (see Text S1 for details).

Screening of the Loki’s Castle metagenomes for NCLDV DNA polymerase (DNAP) sequences revealed remarkable diversity (Fig. 1; see also Fig. S2 in Text S1 in the supplemental material). Using two main binning approaches, namely, differential coverage (DC) binning and coassembly (CA) binning (Fig. 2), we retrieved 23 high-quality bins of putative new NCLDVs (Table 1). The highest-quality bins were identified by comparing the DC and the CA bins, based on decreasing the total number of contiguous sequences (contigs) and the number of contigs without NCLDV hits, while preserving completeness (see Table S6 in Text S1).

**TABLE 1:** The 23 NCLDV bins from Loki’s Castle^*a*^

Bin or virus	Category	No. of contigs	Min contig length, nt	Max contig length, nt	Total contig length, nt	No. of predicted proteins	No. of paralogs of hallmark NCLDV genes														
							MCP	DNAP	ATP	RNApA	RNApB	D5hel	A18hel	VLTF3	VLTF2	RNAp5	Erv1	RNAlig	TopoII	FLAP	TFIIB
LCPAC001	Pitho-like	12	8,088	60,499	249,064	227	1	1	0	1	1	0	1	0	0	0	2	1	1	0	0
LCPAC101	Pitho-like	26	6,043	46,492	466,072	373	1	1	0	1	1	1	1	0	1	1	0	1	1	1	1
LCPAC102	Pitho-like	12	6,510	44,810	285,593	229	1	1	0	1	1	0	0	0	1	0	3	1	0	1	1
LCPAC103	Pitho-like	17	5,380	23,680	204,602	186	1	1	0	1	1	1	1	1	1	1	0	1	1	0	1
LCPAC104	Pitho-like	4	6,208	129,049	218,903	194	1	1	0	1	1	1	1	1	1	1	1	1	1	1	1
LCPAC201	Pitho-like	11	5,186	168,698	428,611	327	1	1	0	1	2	1	1	1	1	1	1	1	1	1	1
LCPAC202	Pitho-like	26	5,141	72,684	443,964	354	1	2	0	1	2	1	1	1	1	1	1	1	1	0	1
LCPAC302	Pitho-like	30	5,274	20,428	290,561	294	0	1	0	1	1	0	0	1	1	1	1	0	1	0	0
LCPAC304	Pitho-like	12	11,737	173,767	638,759	688	1	1	0	1	1	1	1	1	1	1	0	1	1	1	1
LCPAC401	Pitho-like	11	7,155	114,453	484,752	504	1	1	0	1	1	1	1	1	1	2	1	0	1	1	1
LCPAC403	Pitho-like	6	24,087	117,884	420,388	430	1	1	0	1	1	1	1	1	1	2	0	1	1	1	1
LCPAC404	Pitho-like	10	11,211	84,762	436,585	390	1	1	0	1	1	1	1	1	1	2	0	1	1	1	1
LCPAC406	Pitho-like	10	11,113	75,955	384,297	401	1	1	0	1	1	1	1	1	1	2	1	0	1	1	1
LCDPAC01	Pitho-like	21	5,383	31,931	282,320	282	1	1	0	1	1	1	1	1	1	0	1	0	1	1	0
LCDPAC02	Pitho-like	9	6,786	90,916	367,310	390	1	1	0	1	1	1	1	0	0	1	1	0	1	0	0
LCMAC101	Marseille-like	7	15,190	393,561	763,048	793	1	1	1	1	1	1	1	1	1	1	1	1	1	1	1
LCMAC102	Marseille-like	1	395,459	395,459	395,459	465	1	1	1	1	1	1	1	1	1	1	1	1	1	1	1
LCMAC103	Marseille-like	9	14,346	69,824	389,984	427	1	1	1	1	1	1	1	1	0	1	0	1	1	1	0
LCMAC201	Marseille-like	25	6,728	57,873	565,697	566	1	1	1	1	1	0	1	1	0	1	2	1	1	1	1
LCMAC202	Marseille-like	19	6,906	153,726	705,352	672	1	1	2	1	1	1	1	1	0	1	2	1	1	1	1
LCIVAC01	Irido-like	19	5,375	17,223	198,495	222	0	1	0	1	1	1	1	0	1	0	1	1	1	0	1
LCMiAC01	Mimivirus-like	18	8,458	85,120	672,112	571	6	1	1	1	1	1	1	1	1	1	1	1	1	1	0
LCMiAC02	Mimivirus-like	21	8,237	131,456	642,939	583	6	1	2	1	1	2	1	2	1	1	1	1	1	1	1
Cedratvirus A11					589,068	574	1	1	0	1	1	1	1	1	1	1	1	1	1	1	1
Orpheovirus IHUMI LCC2					1,473,573	1199	1	1	0	1	1	1	1	1	1	1	1	1	1	1	1
Pithovirus sibericum					610,033	425	1	1	0	1	1	1	1	1	1	1	1	1	1	1	1
Marseillevirus					369,360	403	1	1	1	1	1	1	1	1	1	1	1	1	1	1	1
Diadromus pulchellus ascovirus 4a					119,343	119	1	1	1	1	1	1	0	1	1	1	1	1	0	0	1
*Heliothis virescens* ascovirus 3e					186,262	180	1	1	1	1	1	1	0	1	1	1	1	1	0	1	1
Lymphocystis disease virus					186,250	239	1	1	1	1	1	1	1	1	1	1	1	1	0	1	0
Frog virus 3					105,903	99	1	1	1	1	1	1	0	1	1	1	1	1	0	1	0
Wiseana iridescent virus					205,791	193	1	1	1	1	1	1	1	1	1	1	1	1	1	1	1
Cafeteria roenbergensis virus BV PW1					617,453	544	3	1	1	1	1	1	1	1	0	1	1	0	1	1	1
*Acanthamoeba polyphaga* mimivirus					1,181,549	979	4	1	2	1	1	1	1	1	1	1	1	0	1	1	1
Klosneuvirus KNV1					1,573,084	1545	7	1	3	1	1	2	1	1	1	1	3	2	1	2	1

a MCP, NCLDV major capsid protein (NCVOG0022); DNAP, DNA polymerase family B, elongation subunit (NCVOG0038); ATP, A32-like packaging ATPase (NCVOG0249); RNApA, DNA-directed RNA polymerase subunit alpha (NCVOG0274); RNApB, DNA-directed RNA polymerase subunit beta (NCVOG0271); D5hel, D5-like helicase-primase (NCVOG0023); A18hel, A18-like helicase (NCVOG0076); VLTF3, poxvirus late transcription factor (TF) VLTF3 (NCVOG0262); VLTF2, A1L TF/late TF VLTF-2 (NCVOG1164); RNAp5, DNA-directed RNA polymerase subunit 5 (NCVOG0273); Erv1, Erv1/Alr family disulfide (thiol) oxidoreductase (NCVOG0052); RNAlig, RNA ligase (NCVOG1088); TopoII, DNA topoisomerase II (NCVOG0037); FLAP, Flap endonuclease (NCVOG1060); TFIIB, transcription initiation factor IIB (NCVOG1127); Min, minimum; Max, maximum; Pitho-like, pithovirus-like virus; Marseille-like, marseillevirus-like virus; Irido-like, iridovirus-like virus; nt, nucleotides. Data on representative complete NCLDV genomes from the relevant families are included for comparison.

10.1128/mBio.02497-18.1 TEXT S1 Supplemental methods, supplemental results, Fig. S1 to S20, and Tables S1 to S6. Download Text S1, PDF file, 2.3 MB. Copyright © 2019 Bäckström et al. 2019 Bäckström et al. This content is distributed under the terms of the Creative Commons Attribution 4.0 International license.

Differential coverage binning was performed first, resulting in 29 genomic bins. Initial quality assessment showed that most of the bins were inflated and fragmented and contained many short (<5-kb) contigs which were difficult to classify as contamination or *bona fide* NCLDV sequences and that some bins were likely to contain sequences from two or more viral genomes, as judged by the presence of marker genes belonging to different families of the NCLDV (see Fig. S19 to S20 in Text S1). The more contigs a bin contains, the higher the risk is that some could represent contaminants that bin together because of similar nucleotide compositions and levels of read coverage. Therefore, sequence read profiling followed by coassembly binning was performed in an attempt to increase the size of the contigs and thus to obtain additional information for binning and bin refinement. For most of the bins, the coassembly led to a decrease in the number of contigs without loss of completeness or even led to improvement in the data (see Table S6 in Text S1).

A key issue with metagenomic binning is whether contigs are binned together because they belong to the same genome or are binned together because they simply display similar nucleotide compositions and levels of read coverage. In general, contigs were retained if they contained at least one gene with BLASTP top hits corresponding to NCLDV proteins. Some contigs encoded proteins with only bacterial, archaeal, and/or eukaryotic BLASTP top hits, and because the larger NCLDV genomes contain islands enriched in genes of bacterial origin (46, 52), it was unclear which sequences potentially represented contaminants. A combination of gene content, read coverage, and composition information was used to identify potential contaminating sequences. Contigs shorter than 5 kb were also discarded because such contigs generally do not contain enough information to reliably establish their origin, but this strict filtering also means that the size of the genomes could be underestimated and some genomic information lost. Reassuringly, no traces of rRNA or ribosomal protein genes were identified in any of the NCLDV genome bins, which would have been represented a clear case of contaminating cellular sequences. Altogether, of the 336 contigs in the 23 final genome bins, 243 (72%) could be confidently assigned to NCLDV on the basis of the presence of at least one NCLDV-specific gene.

The content of the 23 NCLDV-related bins was analyzed in more depth (Table 1). The bins included 1 to 30 contigs, with the total length of nonoverlapping sequences ranging from about 200 kb to more than 750 kb, suggesting that some might contain (nearly) complete NCLDV genomes, although it is difficult to arrive at any definitive conclusions with respect to completeness on the basis of length alone because the genome sizes of even closely related NCLDV can differ substantially. A much more reliable approach is to assess the representation of core genes that are expected to be conserved in (nearly) all NCLDV. The translated protein sequences from the 23 bins were searched for homologs of conserved NCLDV genes using PSI-BLAST, with profiles of the Nucleo-Cytoplasmic Virus Orthologous Group (NCVOG) collection employed as queries (28) (see Data Set S1 in the supplemental material for protein annotations). In 14 of the 23 bins, (nearly) complete sets of the core NCLDV genes were identified (Table 1), suggesting that those bins contained (nearly) complete genomes of putative new viruses (here, LCV [for “Loki’s Castle viruses”]). Notably, the pithovirus-like LCV lack the packaging ATPase of the FtsK family that is encoded in all other NCLDV genomes but not in the available pithovirus genomes. Several bins contained more than one copy of certain conserved genes. Some of these could represent actual paralogs, but, given that duplication of most of these conserved genes (e.g., DNA polymerase in bin LCPAC202 or RNA polymerase B subunit in bins LCPAC201 and LCPAC202) is unprecedented among NCLDV, it appears likely that several bins are heterogeneous, with each containing sequences from two closely related virus genomes.

10.1128/mBio.02497-18.9 DATA SET S1 LCV and LC virophage protein annotation. Download Data Set S1, XLSX file, 1.4 MB. Copyright © 2019 Bäckström et al. 2019 Bäckström et al. This content is distributed under the terms of the Creative Commons Attribution 4.0 International license.

With all due caution because of the lack of fully assembled virus genomes, the range of the apparent genome sizes of the pithovirus-like and marseillevirus-like LCV is notable (Table 1). The characteristic size of the genomes in the family “Pithoviridae” is about 600 kb (17–19), but, among the pithovirus-like LCV, only one, LCPAC304, reached and even exceeded that size. The rest of the LCV genomes are substantially smaller, and although some are likely to be incomplete, given that certain core genes are missing, others, such as LCPAC104, with a total length of contigs of only 218 kb, encompass all the core genes (Table 1).

The typical genome size in the family *Marseilleviridae* is between 350 and 400 kb (22), but among the LCV, genomes of two putative marseillevirus-like viruses, LCMAC101 and LCMAC202, appear to exceed 700 kb, well into the giant virus range. Although LCMAC202 contains two uncharacteristic duplications of core genes, raising the possibility of heterogeneity, LCMAC101 contains all core genes in a single copy and thus appears to represent an actual giant virus. Thus, the family *Marseilleviridae* seems to be joining the NCLDV families that evolved virus gigantism.

**FIG 3:**
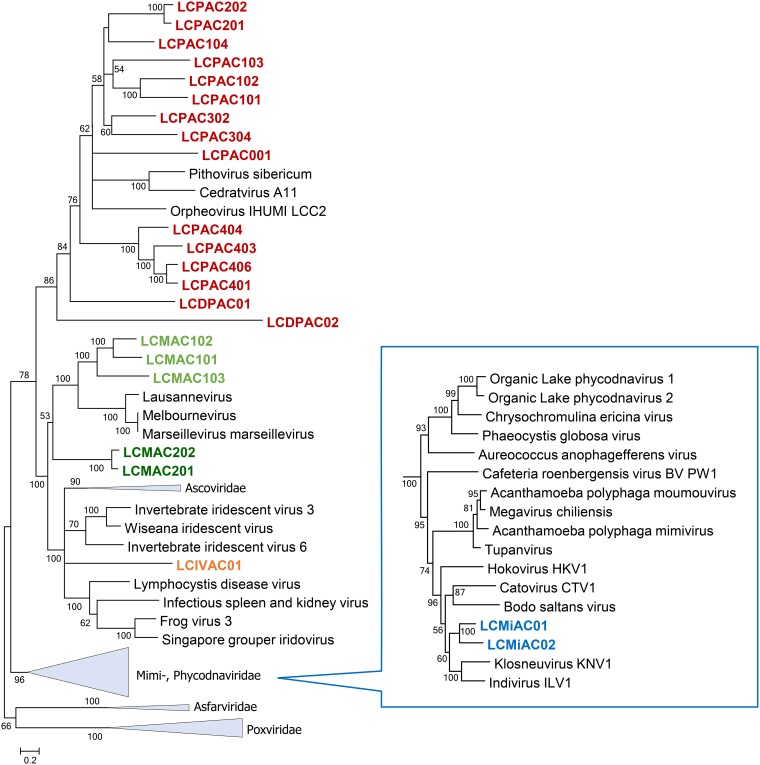
Phylogenetic tree of three concatenated, universally conserved NCLDV proteins: DNA polymerase, major capsid protein, and A18-like helicase. Support values were obtained using 100 bootstrap replications; branches with less than 50% support were collapsed. Scale bars represent the number of amino acid substitutions per site. The inset shows the *Mimiviridae* branch. Triangles show collapsed branches. The LCV sequences are color-coded as follows: red, pithovirus-like virus; green, marseillevirus-like virus (a deep branch is shown in dark green); orange, iridovirus-like virus; blue, mimivirus (klosneuvirus)-like virus.

A concatenation of the three most highly conserved proteins, namely, NCLDV major capsid protein (MCP), DNA polymerase (DNAP), and A18-like helicase (A18Hel), was used for phylogenetic analysis (see Materials and Methods for details). Among the putative new NCLDV, 15 cluster with pithoviruses (Fig. 3). These new representatives greatly expand the scope of the family “Pithoviridae.” Indeed, 8 of the 15 form a putative (weakly supported) clade that is the sister group of all currently known “Pithoviridae” (Pithovirus, Cedratvirus, and Orpheovirus), 5 more comprise a deeper clade, and LCDPAC02 represents the deepest lineage of the pithovirus-like viruses (Fig. 3). Additionally, 5 of the putative new NCLDV are affiliated with the family *Marseilleviridae*, and similarly to the case of pithovirus-like viruses, two of these comprise the deepest branch in the marseillevirus-like subtree (although the monophyly of this subtree is weakly supported) (Fig. 3). Another LCV represents a distinct lineage within the family *Iridoviridae* (Fig. 3). The topologies of the phylogenetic trees for individual conserved NLCDV genes were mostly compatible with these affinities of the putative new viruses (Text S2). Taken together, these findings substantially expand the pithovirus-iridovirus-marseillevirus (PIM) clade of the NCLDV, and the inclusion of the LCV in the phylogeny confidently reaffirms the previously observed monophyly of this branch (Fig. 3). Finally, two LCV belong to the *Klosneuvirus* branch (putative subfamily “Klosneuvirinae”) within the family *Mimiviridae* (Fig. 3, inset).

10.1128/mBio.02497-18.2 TEXT S2 Phylogenetic trees for DNAP, MCP, A18hel, and translation system components. Download Text S2, PDF file, 0.5 MB. Copyright © 2019 Bäckström et al. 2019 Bäckström et al. This content is distributed under the terms of the Creative Commons Attribution 4.0 International license.

### Translation system components encoded by Loki’s Castle viruses.

Similarly to other NCLDV with giant and large genomes, the LCV show patchy distributions of genes coding for translation system components. Such genes were identified in 11 of the 23 bins (Table 2; see also Data Set S1). None of the putative new viruses has a (nearly) complete set of translation-related genes (minus the ribosome) such as have been observed in klosneuviruses (48) or tupanviruses (65). Nevertheless, several of the putative pithovirus-like viruses encode multiple translation-related proteins, e.g., bin LCMAC202, which encompasses 6 aminoacyl-tRNA synthetases (aaRS) and 6 translation factors, and bin LCMAC201, with 4 aaRS and 5 translation factors (Table 2). Additionally, 12 of the 23 bins encode predicted tRNAs, up to 22 in bin LCMAC202 (Table 2).

**TABLE 2:** Translation-related proteins and tRNAs in Loki’s Castle NCLDV

Bin or virus	No. of paralogs of translation-related genes^*a*^																					
	AlaS	AsnS	GRS1	GlnS	HisS	IleS	MetS	ProS	Pth2	RLI1	ThrS	TrpS	TyrS	eIF1	eIF1a	eIF2a	eIF2b	eIF2g	eIF4e	eIF5b	eRF1	tRNA
LCPAC001		1					1															5
LCPAC101			1																			2
LCPAC102																						3
LCPAC103																						
LCPAC104																						4
LCPAC201																						
LCPAC202																						
LCPAC302										1												
LCPAC304		1							1	1		1					1					21
LCPAC401																						
LCPAC403																						
LCPAC404																	1					
LCPAC406																						
LCDPAC01																						
LCDPAC02																						
LCMAC101		3							1													8
LCMAC102																						3
LCMAC103	1															1	1	1	1	1	1	17
LCMAC201		1		1			1						1			1	2	1				11
LCMAC202	1	2						1			1		1	1		1	2	1			1	26
LCIVAC01																						
LCMiAC01					1	1					1		1		1				1			18
LCMiAC02																			2		1	2
*Pithovirus sibericum*																						
Cedratvirus_A11																						
Orpheovirus		1	1		1	1							1	1							1	
Marseillevirus														1			1				1	
Klosneuvirus_KNV1	1	1	1	2	1	1	1	1	3	1	1	1	1	1	1	1	2	1	1		1	25
Mimivirus		1				1	1						1	1					1		2	6
Tupanvirus	1	1	1	2	1	1	1	1			1	1	1	1	1	1	2	1	2		1	
*C. roenbergensis* virus						1								1	1	1	3	1	1	1		16

a Translation-related proteins are abbreviated as follows: AlaS, alanyl-tRNA synthetase; AsnS, aspartyl/asparaginyl-tRNA synthetase; GlnS, glutamyl-tRNA or glutaminyl-tRNA synthetase; GRS1, glycyl-tRNA synthetase (class II); HisS, histidyl-tRNA synthetase; IleS, isoleucyl-tRNA synthetase; MetS, methionyl-tRNA synthetase; ProS, prolyl-tRNA synthetase; ThrS, threonyl-tRNA synthetase; TrpS, tryptophanyl-tRNA synthetase; TyrS, tyrosyl-tRNA synthetase; Pth2, peptidyl-tRNA hydrolase; eIF1, translation eukaryotic initiation factor 1 (eIF-1/SUI1); eIF1a, translation eukaryotic initiation factor 1A/IF-1; eIF2a, translation eukaryotic initiation factor 2, alpha subunit (eIF-2alpha); eIF2b, translation eukaryotic initiation factor 2, beta subunit (eIF-2beta)/eIF-5 N-terminal domain; eIF2g, translation eukaryotic initiation factor 2, gamma subunit (eIF-2gamma; GTPase); eIF4e, translation eukaryotic initiation factor 4E (eIF-4E); eIF5b, translation eukaryotic initiation factor 2/eukaryotic initiation factor 5B (eIF5B) family (IF2/eIF5B); eRF1, peptide chain release factor 1 (eRF1); RLI1, translation initiation factor RLI1. Data for completely sequenced representatives of the relevant NCLDV families are included for comparison.

**FIG 4:**
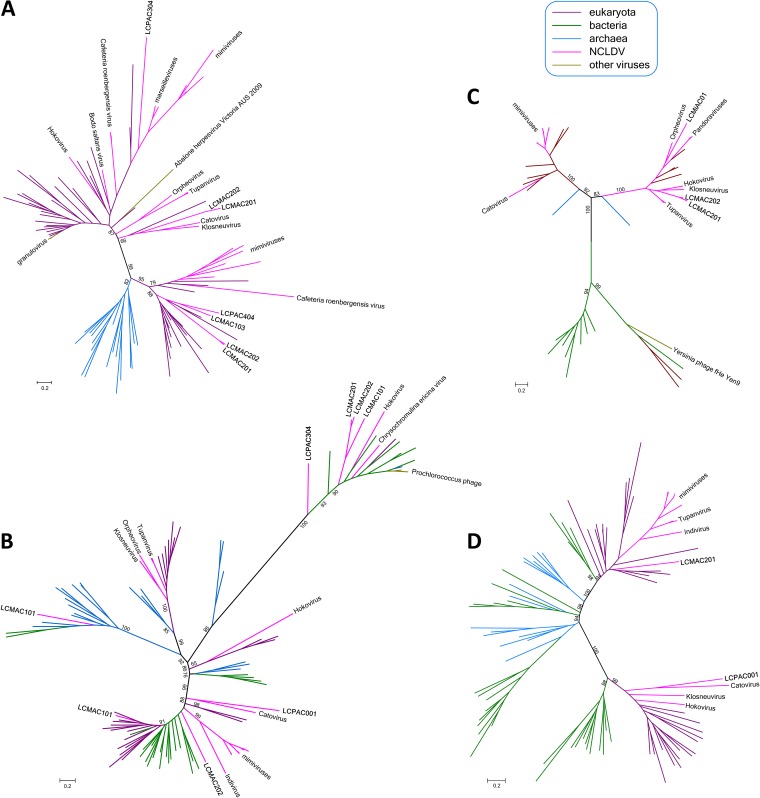
Phylogenies of selected translation system components encoded by Loki’s Castle viruses. (A) Translation initiation factor eIF2b. (B) Aspartyl/asparaginyl-tRNA synthetase (AsnS). (C) Tyrosyl-tRNA synthetase (TyrS). (D) Methionyl-tRNA synthetase (MetS). All branches are color-coded according to taxonomic affinity (see Text S2 for the full trees). The numbers at the internal branches indicate (percent) local likelihood-based support.

Given the special status of the translation system components in the discussions of the NCLDV evolution, we constructed phylogenies for all these genes, including genes corresponding to the LCV and all other NCLDV. The results of this phylogenetic analysis (Fig. 4; see also Text S2) reveal complex evolutionary trends, some of which that have not been apparent in previous analyses of NCLDV evolution. First, in most cases, when multiple LCV encompass genes for homologous translation system components, phylogenetic analysis demonstrates polyphyly of these genes. Notable examples include translation eukaryotic initiation factor 2b (eIF2b), aspartyl/asparaginyl-tRNA synthetase (AsnS), tyrosyl-tRNA synthetase (TyrS), and methionyl-tRNA synthetase (MetS; Fig. 4). Thus, the eIF2b tree includes 3 unrelated LCV branches, one of which, not unexpectedly, clusters with homologs from marseilleviruses and mimiviruses; another is affiliated with two klosneuviruses, and the third appears to have an independent eukaryotic origin (Fig. 4A). The AsnS tree includes a group of LCV that cluster within a mixed bacterial and archaeal branch that also includes two other NCLDV, namely, hokovirus of the klosneuvirus group and a phycodnavirus. Another LCV AsnS belongs to a group of apparent eukaryotic origin, and, finally, one belongs to a primarily archaeal clade (Fig. 4B; see also Text S2). Of the 3 TyrS found in LCV, two cluster with the homologs from klosneuviruses within a branch of apparent eukaryotic origin and the third within another part of the same branch, where it groups with the orpheovirus TyrS; notably, the same branch includes homologs from pandoraviruses (Fig. 4C). Of the two examples of MetS, one groups with homologs from klosneuviruses whereas the other one appears to be of independent eukaryotic origin (Fig. 4D). These observations are compatible with previous conclusions concerning multiple, parallel acquisitions of genes for translation system components by different groups of NCLDV (primarily giant viruses but, to a lesser extent, also those with smaller genomes), apparently under evolutionary pressure for modulation of host translation, which remains to be studied experimentally.

Another clear trend among the translation-related genes of the pithovirus-like LCV is the affinity of several of them with homologs from klosneuviruses and, in some cases, mimiviruses. All 4 examples mentioned about include genes of this provenance, and additional cases include genes encoding GlyS, IleS, ProS, peptidyl-tRNA hydrolase, translation factors eIF1a and eIF2a, and peptide chain release factor eRF1 (Text S2). Given that the LCV set includes two Klosneuvirus-like bins, in addition to the pithovirus-like ones, these observations imply extensive gene exchange between distinct NCLDV in the habitats from which these viruses originate. Klosneuviruses that are conspicuously rich in translation-related genes might serve as the main donors.

### Gene content analysis of the Loki’s Castle viruses.

**FIG 5:**
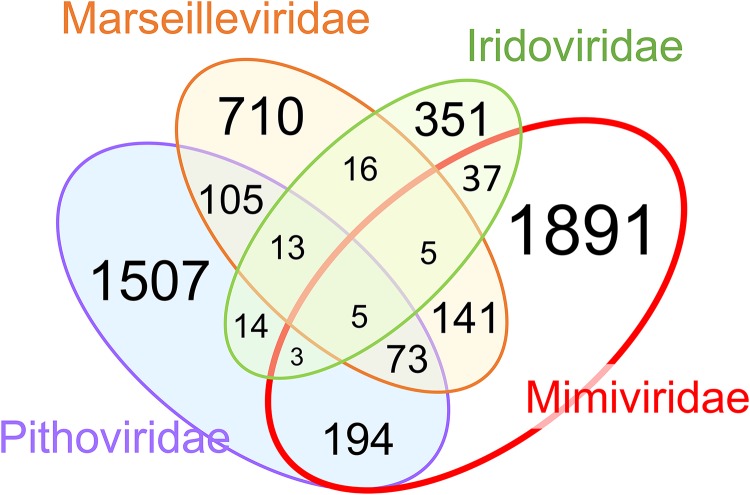
Shared and unique genes in four NCLDV families that include Loki’s Castle viruses. The numbers correspond to NCLDV clusters that contain at least one protein from *Mimiviridae*, *Marseilleviridae*, *Pithoviridae*, and *Iridoviridae* but are absent from other NCLDV families.

Given that the addition of the LCV has greatly expanded the family *Marseilleviridae* and the pithovirus group and has reaffirmed the monophyly of the PIM branch of NCLDV, we constructed, analyzed and annotated clusters of putative orthologous genes for this group of viruses as well as for an automatically generated version of clusters of homologous genes for all NCLDV (ftp://ftp.ncbi.nih.gov/pub/yutinn/Loki_Castle_NCLDV_2018/NCLDV_clusters/). Altogether, 8,066 NCLDV gene clusters were identified, a substantial majority of which were family specific. Nevertheless, almost 200 clusters were found to be shared between the “Pithoviridae” and *Marseilleviridae* families (Fig. 5). The numbers of genes shared by each of these families with *Iridoviridae* were much lower, conceivably because of the small genome size of iridoviruses that could have undergone reductive evolution (Fig. 5). In contrast, there was considerable overlap between the PIM group gene clusters and those of mimiviruses, presumably due to the large genome sizes of the mimiviruses but potentially also reflecting substantial horizontal gene flow between mimiviruses, pithoviruses, and marseilleviruses (Fig. 5). Only 13 genes comprised a genomic signature of the PIM group, that is, of genes that were shared by its three constituent families to the exclusion of the rest of the NCLDV.

**FIG 6:**
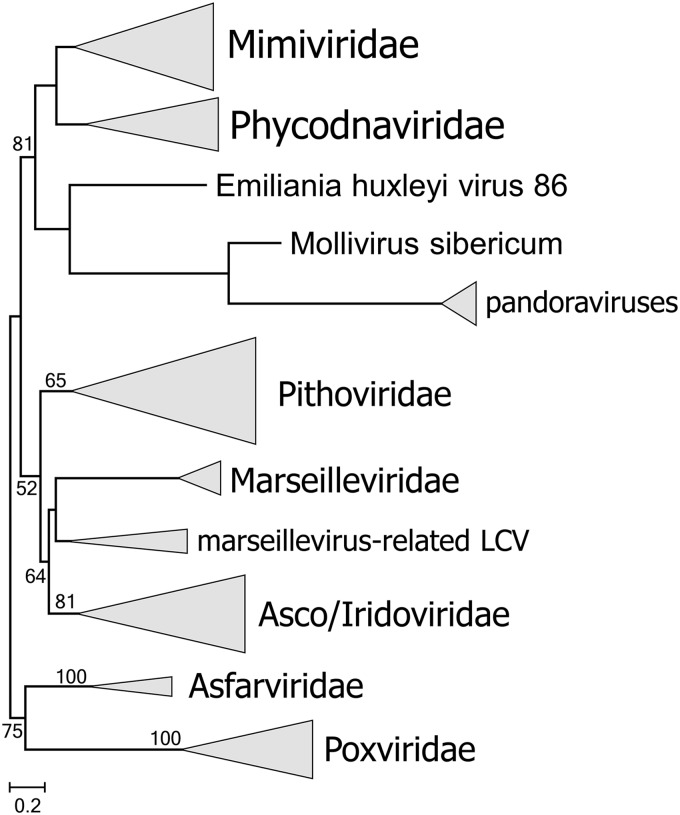
Gene presence-absence tree of the NCLDV that include the Loki’s Castle viruses. The neighbor-joining dendrogram was reconstructed from the matrix of pairwise distances calculated from binary phyletic patterns of the NCLDV clusters. The numbers at internal branches indicate (percent) bootstrap support; data below 50% are not shown.

To further explore the relationships between the gene repertoires of the PIM group and other NCLDV, we constructed a neighbor-joining tree from the data on gene presence-absence (ftp://ftp.ncbi.nih.gov/pub/yutinn/Loki_Castle_NCLDV_2018/NCLDV_clusters/). Notwithstanding the limited gene sharing, the topology of the resulting tree (Fig. 6) closely recapitulated the phylogenetic tree of the conserved core genes (Fig. 3). In particular, the PIM group appears as a clade in the gene presence-absence tree, albeit with comparatively low support (Fig. 6). Thus, despite the paucity of PIM-specific genes and the substantial differences in genome sizes between the three virus families, gene gain and loss processes within the viral genetic core appear to track the evolution of the universally conserved genes.

The genomes of microbes and large viruses encompass many lineage-specific genes (often denoted ORFans) that, in the course of evolution, are lost and gained by horizontal gene transfer at extremely high rates (66). Therefore, the gene repertoire of a microbial or viral species (notwithstanding the well-known difficulties with the species definition) or group is best characterized by the pangenome, i.e., the entirety of genes represented in all isolates in the group (67–69). Most microbes have “open” pangenomes such that every sequenced genome adds new genes to the pangenome (69, 70). The NCLDV pangenomes could be even wider, judging from the high percentage of ORFans, especially in giant viruses (71). Examination of the PIM gene clusters shows that 757 (48%) of the 1,572 clusters were unique to the LCV, that is, had no detectable homologs in other members of the group. Taking into account also the 4,147 ORFans, the LCV represent the bulk of the PIM group pangenome. Among the NCLDV clusters, 1,100 of the 8,066 (14%) are LCV specific. Thus, notwithstanding the limitations of the automated clustering procedure, which could miss some distant similarities between proteins, the discovery of the LCV substantially expands not only the pangenome of the PIM group but also the overall NCLDV pangenome.

**FIG 7:**
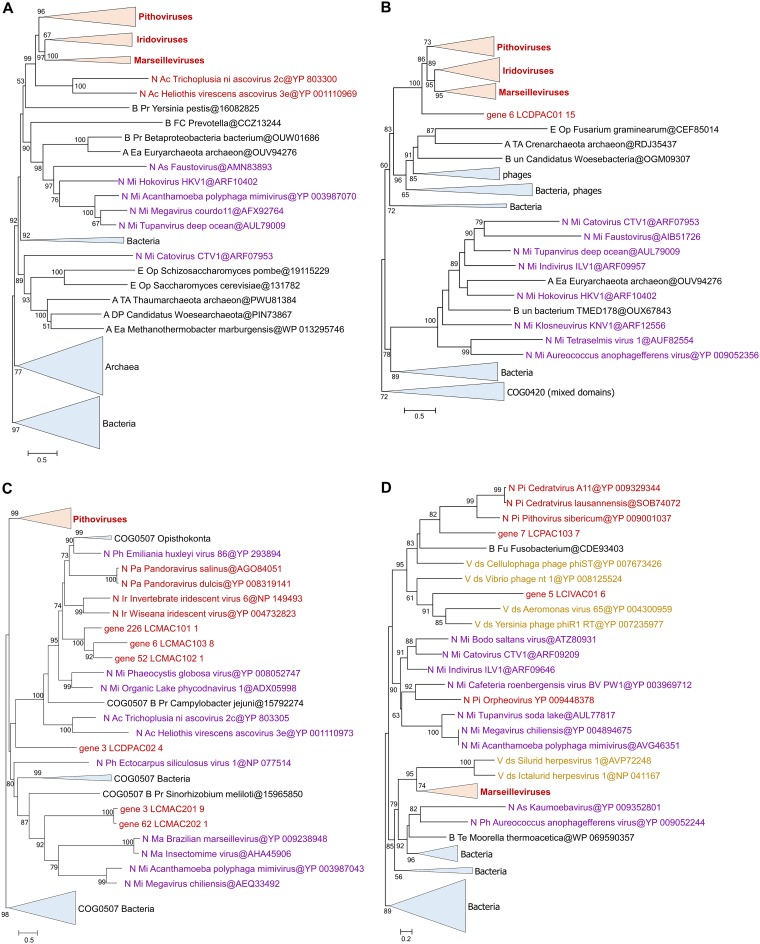
Phylogenies of selected repair and nucleotide metabolism genes of the pithovirus-iridovirus-marseillevirus group that includes Loki’s Castle viruses. (A) SbcCD nuclease, ATPase subunit SbcC. (B) SbcCD nuclease, nuclease subunit SbcD. (C) Exonuclease V. (D) DNMP kinase. The numbers at the internal branches indicate (percent) local likelihood-based support. GenBank protein identifiers (IDs), wherever available, are shown after each “@” symbol. Taxon abbreviations are as follows: A, Archaea; B, Bacteria; E, Eukaryotes; N, NCLDV; DP, DPANN group; TA, Thaumarchaeota; Ea, Euryarchaeota; FC, Bacteroidetes; Fu, Fusobacteria; Pr, Proteobacteria; Te, Firmicutes; un, unclassified Bacteria; Op, Opisthokonta; Pi, “Pithoviridae”; Ac, Ascoviridae; As, Asfarviridae; Ma, Marseilleviridae; Mi, Mimiviridae; Pa, Pandoraviridae; Ph, Phycodnaviridae; V ds, double-strand DNA viruses.

Annotation of the genes characteristic of (but not necessarily exclusive to) the PIM group reveals numerous, highly diverse functions of either bacterial or eukaryotic provenance as suggested by the taxonomic affiliations of homologs detected in database searches (Data Set S2). For example, a functional group of interest shared by the three families in the PIM group includes genes of apparent bacterial origin involved in various DNA repair processes and nucleotide metabolism. The results of phylogenetic analysis of these genes are generally compatible with bacterial origin, although many branches are mixed and also include archaea and/or eukaryotes, indicating horizontal gene transfer (Fig. 7). Notably, these trees illustrate the “hidden complexity” of NCLDV evolution whereby homologous genes are independently captured by different groups of viruses. The PIM group forms a clade in the trees for the two subunits of the SbcCD nuclease, but the homologs in mimiviruses appear to be of distinct origin (Fig. 7A and B), whereas the PIM group itself splits between 3 branches in the trees for exonuclease V and deoxynucleotide monophosphate (DNMP) kinase (Fig. 7C and D). The latter two trees also contain branches in which different groups of the NCLDV, in particular, marseilleviruses and mimiviruses, are mixed, apparently reflecting gene exchange between distinct viruses infecting the same host, such as amoeba.

10.1128/mBio.02497-18.10 DATA SET S2 Taxonomic breakdown of PSI-BLAST hits retrieved with profiles created from selected protein clusters for the PIM group of the NCLDV. Download Data Set S2, XLSX file, 0.6 MB. Copyright © 2019 Bäckström et al. 2019 Bäckström et al. This content is distributed under the terms of the Creative Commons Attribution 4.0 International license.

### Loki’s Castle virophages.

**FIG 8:**
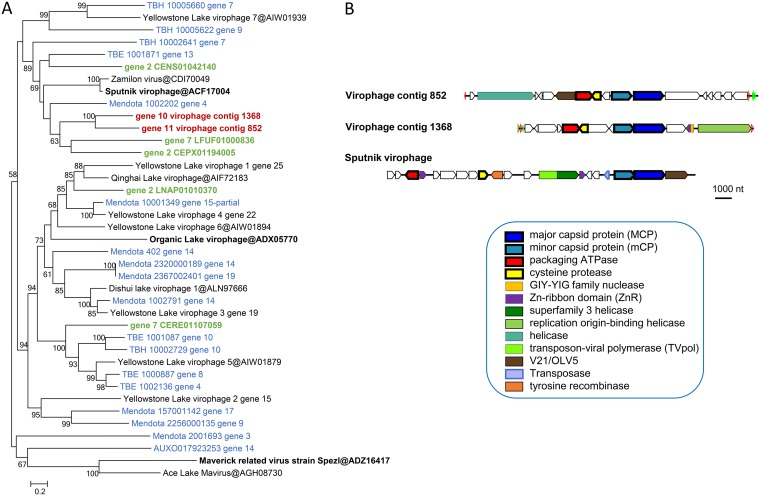
Loki’s Castle virophages. (A) Phylogenetic tree of virophage major capsid proteins. Reference virophages from GenBank are marked with black font (the three prototype virophages are shown in bold); environmental virophages are shown in blue (129) and green (wgs portion of GenBank). (B) Genome maps of Loki’s Castle virophages compared with Sputnik virophage. Green and blue triangles mark direct and inverted repeats. Pentagons with a thick outline represent conserved virophage genes.

Many members of the family *Mimiviridae* are associated with small satellite viruses that became known as virophages (subsequently classified in the family *Lavidaviridae* [72–78]). Two virophage-like sequences were retrieved from Loki Castle metagenomes. According to the MCP phylogeny, they form a separate branch within the Sputnik-like group (Fig. 8A). This affiliation implies that these virophages are parasites of mimiviruses. Besides MCP, both Loki’s Castle virophages encode the proteins involved in virion morphogenesis, namely, minor capsid protein, packaging ATPase, and cysteine protease (Fig. 8B; see also Data Set S1 for protein annotations). Apart from these core genes, however, these virophages differ from Sputnik. In particular, they lack the gene for the primase-helicase fusion protein that is characteristic of Sputnik and its close relatives (79), but each encodes a distinct helicase (Fig. 8B; see also Text S3 for additional virophage genome maps).

10.1128/mBio.02497-18.3 TEXT S3 Virophage genome maps. Download Text S3, PDF file, 0.1 MB. Copyright © 2019 Bäckström et al. 2019 Bäckström et al. This content is distributed under the terms of the Creative Commons Attribution 4.0 International license.

### Putative promoter motifs in LCV and Loki’s Castle virophages.

To identify possible promoter sequences in the LCV genomes, we searched “upstream” regions of the predicted LCV genes for recurring motifs using MEME software (see Materials and Methods for details). In most of the bins, we identified a conserved motif similar to the early promoters of poxviruses and mimiviruses (80) (AAAnTGA) that is typically located within 40 to 20 nucleotides upstream of the predicted start codon (for the search results, see ftp://ftp.ncbi.nih.gov/pub/yutinn/Loki_Castle_NCLDV_2018/meme_motif_search/). To assess possible bin contamination, we calculated the frequencies of the conserved motifs per contig for marseillevirus-like and mimivirus-like bins. None of the contigs showed significantly reduced frequencies of the conserved motif (Text S5), supporting the idea of the virus origin of all the contigs.

Notably, the LCV virophage genomes also contain a conserved AT-rich motif upstream of each gene which is likely to correspond to the late promoter of their hosts, similarly to the case of the Sputnik virophage that carries late mimivirus promoters (81). However, the genomes of the two putative klosneuviruses (LCMiAC01 and LCMiAC02) that are not represented among the LCV do not contain obvious counterparts to these predicted virophage promoters (Text S6). Therefore, it appears most likely that the hosts of these virophages are mimiviruses that are not represented in the LCV sequence set.

Of further interest is the detection of pronounced promoter-like motifs for pithovirus-like LCV (Text S7) and iridovirus-like LCV (Text S8). To our knowledge, no conserved promoter motifs have been identified so far for these groups of viruses.

## DISCUSSION

Metagenomics has become the primary means of new virus discovery (53, 54, 82). Metagenomic sequence analysis has greatly expanded knowledge of many groups of viruses such that the viruses that were identified earlier by traditional methods have become isolated branches in the overall evolutionary trees, in which most of the diversity comes from metagenomic sequences (83–88). The analysis of the Loki’s Castle metagenome reported here similarly expanded the *Pithovirus* branch of the NCLDV, and to a somewhat lesser extent, the *Marseillevirus* branch. Although only one LCV genome, that of a marseillevirus-like virus, appears to be complete and on a single contig, several other genomes seem to be nearly complete, and overall, the LCV genomic data are sufficient to dramatically expand the pangenome of the PIM group, to add substantially to the NCLDV pangenome as well, and to reveal notable evolutionary trends. First, the LCV retain all or most of the NCLDV core genes, reinforcing the previously established monophyly analysis of this vast assemblage of large double-stranded DNA (dsDNA) viruses infecting diverse eukaryotes (1–3, 28–31, 51). The conservation of the gene core inherited from the common virus ancestor of the NCLDV contrasts with the dynamic character of the NCLDV evolution, which involved extensive gene gain and loss, yielding viruses that span a range of about 100 to about 2,500 genes (25, 31, 51). More specifically, the results determined in the present work demonstrate the independent origin of giant viruses in more than one clade within both the *Pithovirus* and the *Marseillevirus* branches. Although this observation should be interpreted with caution, given the lack of fully assembled LCV genomes, it supports and extends the previous conclusions with respect to the evolution of the NCLDV in the genomic accordion regime that led to the independent, convergent evolution of viral gigantism in several or perhaps even all NCLDV families (30, 31, 51, 89). Conversely, these findings are incompatible with the concept of reductive evolution of NCLDV from giant viruses as the principal evolutionary mode. Another notable evolutionary trend emerging from the LCV genome comparison is the apparent extensive gene exchange between pithovirus-like and marseillevirus-like viruses and the members of the *Mimiviridae*. Finally, note that the LCV analysis reaffirms, on a greatly expanded data set, the previously proposed monophyly of the PIM group of the NCLDV, demonstrating robustness of the evolutionary analysis of conserved NCLDV genes (28, 30). Furthermore, a congruent tree topology was obtained by gene content analysis, indicating that, despite the open pangenomes and the dominance of unique genes, the evolution of the genetic core of the NCLDV appears to track the sequence divergence of the universal marker genes.

Like other giant viruses, several LCV encode multiple translation system components. Although none of them rivals the nearly complete translation systems encoded by klosneuviruses (48), orpheoviruses (19), and, especially, tupanviruses (65), some are comparable, in this regard, to the mimiviruses (30). The diverse origins of the translation system components in LCV suggested by phylogenetic analysis are compatible with the previous conclusions on the piecemeal capture of these genes by giant viruses as opposed to inheritance from a common ancestor (30, 46).

The 23 NCLDV genome bins reconstructed in the present study represent only a small fraction of the full NCLDV diversity as determined by analysis of DNA polymerase sequences present in marine sediments (Fig. 1). Notably, sequences closely matching the sequences in the NCLDV genome bins were identified only in the Loki’s Castle metagenomes and not in Tara Oceans water column metagenomes or Earth Virome sequences. Thus, the deep sea sediments represent a unique and unexplored habitat for NCLDVs. Further studies targeting deep sea sediments will bring new insights into the diversity and genomic potential of these viruses.

Identification of the host range is one of the most difficult problems in metaviromics and also in the study of giant viruses, even by traditional methods. Most of the giant viruses have been isolated by cocultivation with model amoeba species, and the natural hosts remain unknown. Notable exceptions are the giant viruses isolated from the marine flagellates *Cafeteria roenbergensis* (12) and *Bodo saltans* (38). The principal approach for inferring the virus host range from metagenomics data is the analysis of co-occurrence of virus sequences with those of potential hosts (90, 91). However, virtually no 18S rRNA gene sequences of eukaryotic origin were detected in the Loki’s Castle sediment samples, in sharp contrast to the results of analysis of rich prokaryotic microbiota (63, 64). The absence of potential eukaryotic hosts of the LCV strongly suggests that these viruses do not reproduce in the sediments but rather might originate from virus particles that precipitate from different parts of the water column. So far, however, no closely related sequences have been found in water column metagenomes (Fig. 1). The eukaryotic hosts might have inhabited the shallower sediments, and although they would have decomposed over time, the resilient virus particles remain as a “fossil record.” Clearly, the hosts of these viruses remain to be identified. An obvious and important limitation of this work—as in any such metagenomic study—is that the viruses discovered here (we are now in a position to refer to the viruses without quotation marks, given the recent decisions of the International Committee on Taxonomy of Viruses [ICTV]) have not been grown in a host culture. Accordingly, our understanding of their biology is limited to the inferences made from the genomic sequence which, perforce, cannot yield the complete picture. In the case of the NCLDV, the effects of these limitations are exacerbated by the fact that their genomic DNA is not infectious; therefore, even the availability of the complete genome does not enable growth of the virus. The metagenomic analyses must complement rather than replace traditional virology and newer culturomic approaches.

Although the sediment samples used in this study have not been dated directly, determinations of sedimentation rates in nearby areas show that these rates range between 1 and 5 cm per 1,000 years (92, 93). With the highest sedimentation rate considered, the sediments could be over 20,600 years old at the deepest level (103 cm). Considering that *Pithovirus sibericum* and *Mollivirus sibericum* were revived from 30,000-year-old permafrost (17, 20), it might be possible to resuscitate some of the LCVs using similar methods. Isolation experiments performed with giant viruses from deep sea sediments, now that we are aware of their presence, would be the natural next step in learning more about their biology.

Regardless, the discovery of the LCV substantially expands the known ocean megavirome and demonstrates the previously unsuspected high prevalence of pithovirus-like viruses. Given that all this diversity comes from a single site on the ocean floor, it appears clear that the megavirome is large and diverse and that metagenomics analysis of NCLDV from other sites will bring many surprises.

## MATERIALS AND METHODS

### Sampling and metagenomic sequencing.

In the previous studies of microbial diversity in the deep sea sediments, samples were retrieved from three sites about 15 km northeast of the Loki's Castle hydrothermal vent field (see Table S1 in Text S1 in the supplemental material) by gravity (GS10_GC14 and GS08_GC12) and by piston coring (GS10_PC15) (63, 94, 95).

DNA was extracted and sequenced, and metagenomes were assembled as part of the previous studies (63 [for GS10_GC14], Dharamshi et al. [submitted] [for GS08_GC12 and GS10_PC15]), resulting in the assemblies LKC75, KR126, K940, K1000, and K1060. Contiguous sequences (contigs) longer than 1 kb were selected for further processing.

### Identification of viral metagenomic sequences.

Protein sequences of the metagenomic contigs were predicted using Prodigal v.2.6.3 (96) in the metagenomics mode. A collection of DNAP sequences from 11 NCLDV was used to query the metagenomic protein sequence with BLASTP (97) (see Table S1 in Text S1). The BLASTP hits were filtered according to E value (maximum, 1e^−5^), alignment length (at least 50% of the query length), and identity (greater than 30%). The sequences were aligned using MAFFT-LINSI software (98). Reference NCLDV DNAP sequences were extracted from the NCVOG collection (28). Highly divergent sequences and those containing large gap insertions were removed from the alignment, followed by realignment. The terminal regions of the alignments were trimmed manually using Jalview (99), and internal gaps were removed using trimAl (v.1.4.rev15 [100]) with the option “gappyout.” IQTree version 1.5.0a (101) was used to construct maximum likelihood (ML) phylogenies with 1,000 ultrafast bootstrap replications (102). The built-in model test (103) was used to select the best evolutionary model according to the Bayesian information criterion (LG+F+I+G4; see Fig. S1 in Text S1). Contigs belonging to novel NCLDVs were identified and used for binning.

### Composition-based binning (ESOM).

All sequences of the KR126, K940, K1000, and K1060 assemblies were split into fragments of minimum lengths of 5 or 10 kb at intervals of 5 or 10 kb and were clustered using tetranucleotide frequencies and Emergent Self Organizing maps (ESOM [104]), generating one map per assembly (see Text S1). Bins were identified by viewing the maps using the Databionic ESOM viewer (http://databionic-esom.sourceforge.net/) and manually choosing the contigs clustering together with the putative NCLDV contigs in an “island” (see Fig. S3 in Text S1).

### Differential coverage binning of metagenomic contigs.

Differential coverage (DC) bins were generated for the KR126, K940, K1000, and K1060 metagenomes, according to the method of Dharamshi et al. (submitted). Briefly, Kallisto version 0.42.5 (105) was used to get the differential coverage data for each read mapped onto each focal metagenome, with CONCOCT version 0.4.1 used to collect sequences into bins (106). CONCOCT was run with three different contig size thresholds (2 kb, 3 kb, and 5 kb), and longer contigs were cut up into smaller fragments (10 kb), to decrease coverage and compositional bias, and merged again after the CONCOCT binning (see Dharamshi et al. [submitted] for further details). Bins containing contigs with the viral DNAP were selected and refined in mmgenome (107). Finally, to resolve overlapping sequences in the DC bins, the reads of each bin were extracted using seqtk (version 1.0-r82-dirty; https://github.com/lh3/seqtk) and the read-mapping files generated for mmgenome and were reassembled using SPAdes (3.6.0, multi-cell, –careful mode [108]). The coverage and quality of the data corresponding to the bins from KR126 were too low, and the data were discarded from further analysis.

### Coassembly binning of metagenomic contigs.

CLARK (109), a program for classification of reads using discriminative k-mers, was used to identify reads belonging to NCLDV in the metagenomes. A target set of 10 reference genomes that represented klosneuviruses, *Marseilleviridae*, and “Pithoviridae” (see Table S2 in Text S1), as well as the 29 original bins, was used to make a database of spaced k-mers which CLARK used to classify the reads of the K940, K1000, and K1060 metagenomes (full mode, k-mer size 31). Reads classified as related to any of the targets were extracted, and the reads from all three metagenomes were pooled and reassembled using SPAdes (3.9.0 [108]). Because CLARK removes k-mers that are not discriminatory, the reads for sequences that are similar between the bins might not have been included. Therefore, the reads from each original bin that were used for the first set reassemblies were also included and were pooled with the CLARK-classified reads before reassembly.

Four SPAdes modes were tested: metagenomic (–meta), single-cell (–sc), multicell (default), and multicell careful (–careful). The quality of the assemblies was tested by identifying the contigs containing NCVOG0038 (DNA polymerase), using BLASTP (97). The multicell careful assembly had the longest DNAP-containing contigs and was used for CONCOCT binning.

CONCOCT was run as described above, except that only reads from the coassembly were used as the input. Bins containing NCVOG0038 were identified by BLASTP. The smaller the contig size threshold, the greater the number of ambiguous and potentially contaminating sequences observed; therefore, the CONCOCT 5-kb run was chosen to extract and refine new bins. The bins were refined by using mmgenome as described below.

### Quality assessment and refinement of metagenomic NCLDV bins.

General sequence statistics were calculated by Quast (v. 3.2 [110]). Barrnap (v 0.8 [111]) was used to check for the presence of rRNA genes, with a length threshold of 0.1. Prokka (v1.12 [110]) was used to annotate open reading frames (ORFs) of the raw bins. The presence or absence of a megavirus marker gene in each metagenomic bin was estimated by using the micomplete pipeline (https://bitbucket.org/evolegiolab/micomplete) and a set of the 10 conserved NCLDV genes (see Table S3 in Text S1). This information was used to assess completeness and redundancy. The presence of two or more copies of each marker gene was considered an indication of potential contamination or of the presence of two or more copies of viral genomes per bin, and such bins were further refined.

The mmgenome was used to manually refine the metagenomic bins by plotting coverage and GC content, showing read linkages, and highlighting contigs with marker genes (107) (see Text S1 and S4). Overlap of the ESOM binned contigs and the DC bins was also visualized. Bins containing only one genome were refined by removing contigs with different compositions and levels of coverage. In cases in which several genomes were represented in the same CONCOCT bin, they were separated into different bins when distinct clusters were clearly visible (see the supplemental materials and methods in Text S1 for examples of the refining process).

10.1128/mBio.02497-18.4 TEXT S4 Repeat plots. Download Text S4, PDF file, 2.2 MB. Copyright © 2019 Bäckström et al. 2019 Bäckström et al. This content is distributed under the terms of the Creative Commons Attribution 4.0 International license.

10.1128/mBio.02497-18.5 TEXT S5 Conserved promoter-like motif frequencies in selected LCV bins. Download Text S5, PDF file, 0.2 MB. Copyright © 2019 Bäckström et al. 2019 Bäckström et al. This content is distributed under the terms of the Creative Commons Attribution 4.0 International license.

10.1128/mBio.02497-18.6 TEXT S6 Conserved promoter-like motifs in the LCMiAC01 and LCMiAC02 bins and LCV virophages. Download Text S6, PDF file, 0.3 MB. Copyright © 2019 Bäckström et al. 2019 Bäckström et al. This content is distributed under the terms of the Creative Commons Attribution 4.0 International license.

10.1128/mBio.02497-18.7 TEXT S7 Conserved promoter-like motifs in pithovirus-like LCV. Download Text S7, PDF file, 1.7 MB. Copyright © 2019 Bäckström et al. 2019 Bäckström et al. This content is distributed under the terms of the Creative Commons Attribution 4.0 International license.

10.1128/mBio.02497-18.8 TEXT S8 Conserved promoter-like motifs in marseillevirus-like and iridovirus-like LCV. Download Text S8, PDF file, 0.5 MB. Copyright © 2019 Bäckström et al. 2019 Bäckström et al. This content is distributed under the terms of the Creative Commons Attribution 4.0 International license.

Read linkages were determined by mapping the metagenomic reads onto the assembly using bowtie2 (version 2.3.2 [112]) and samtools (version 1.2 [113]) to index and convert the mapping file into bam format; finally, a script provided by the CONCOCT suite was used to count the number of read pairs that mapped to the first or last kilobase of two different contigs (bam_to_linkage.py, –regionlength 1000).

Diamond aligner BLASTP (114) was used to query the protein sequences of the refined bins against the NCBI nonredundant protein database (latest date of search, 13 Febuary 2018), with a maximum E value of 1e^−5^. Taxonomic information from the top BLASTP hit for each gene was used for taxonomic filtering. Contigs that had 50% or more bacterial or archaeal hits (compared to an absence of significant hits) and no viral or eukaryotic hits were identified as likely contaminants and removed.

The assemblies of the DC and CA bins were compared by aligning the contigs with nucmer (part of MUMmer3.23 [115]), using an in-house script for visualization (see Text S1 for more details).

### Assessment of NCLDV diversity.

Environmental sequences, downloaded in March 2017 from Tara Oceans (116) (https://www.ebi.ac.uk/ena/about/tara-oceans-assemblies) and from EarthVirome (59) (https://img.jgi.doe.gov/vr/), were combined with the metagenomic sequences from Loki’s Castle (see Table S1 in Text S1) and screened for sequences related to the Loki’s Castle NCLDVs using BLASTP searches with the bin DNAP sequences as queries. The BLASTP hits were filtered according to E value (maximum, 1e^−5^), high-scoring segment pair (HSP) length (at least 50% of the query length), and identity above 30%. The sequences were extracted using blastdbcmd, followed by alignment and phylogenetic tree reconstruction performed as described above (Fig. 1).

### Sequence annotation and phylogenetic analysis.

The sequences of the selected bins were translated with MetaGeneMark (117). tRNA genes were predicted using tRNAscan-SE online (118). Predicted proteins were annotated using their best hits to the NCVOG, cdd, and *nr* databases. In addition, pithovirus-, marseillevirus-, and iridovirus-related bins were annotated using protein clusters constructed as described below. Reference sequences were collected from corresponding NCVOG and cdd profiles, and from GenBank, using BLASTP searches initiated using the Loki’s Castle NCLDV proteins. Reference sequences for Loki’s Castle virophages were retrieved by BLAST and tBLASTn searches against genomic (nr) and metagenomic (environmental whole-genome sequence [wgs]) parts of GenBank, with the predicted Loki’s Castle virophage MCP as queries. The retrieved environmental virophage genome fragments were translated with MetaGeneMark. Homologous sequences were aligned using MUSCLE (119). For phylogenetic reconstruction, gapped columns (more than 30% gaps) and columns with low information content were removed from the alignments (120); the filtered alignments were used for tree reconstructions using FastTree (121). The alignments of three conserved NCLDV proteins were concatenated and used for phylogenetic analysis with PhyML (122) (http://www.atgc-montpellier.fr/phyml-sms/) The best model identified by PhyML was LG +G + I+F (LG substitution model, gamma distributed site rates with gamma shape parameter estimated from the alignment; fraction of invariable sites estimated from the alignment; and empirical equilibrium frequencies).

### Protein sequence clusters.

Two sets of viral proteins, namely, pithovirus-iridovirus-marseillevirusvirus group proteins (PIM clusters; ftp://ftp.ncbi.nih.gov/pub/yutinn/Loki_Castle_NCLDV_2018/PIM_clusters/) and NCLDV proteins (NCLDV clusters; ftp://ftp.ncbi.nih.gov/pub/yutinn/Loki_Castle_NCLDV_2018/NCLDV_clusters/), were used separately to obtain two sets of protein clusters, using an iterative clustering and alignment procedure, organized as follows.

### (i) Initial sequence clustering.

Initially, sequences were clustered using UCLUST (123) with a similarity threshold of 0.5; clustered sequences were aligned using MUSCLE, and singletons were converted to pseudoalignments consisting of just one sequence. Sites containing more than 67% gaps were temporarily removed from alignments, and the pairwise similarity scores were obtained for clusters using HHSEARCH. Scores for a pair of clusters were converted to distances {the *d_A_*_,_*_B_* = −log[*s_A_*_,_*_B_*/min(*s_A_*_,_*_A_*,*s_B_*_,_*_B_*)] formula was used to convert scores *s* to distances *d*}, and a unweighted pair group method using average linkages (UPGMA) guide tree was produced from a pairwise distance matrix. A progressive pairwise alignment of the clusters at the tree leaves was constructed using HHALIGN (124), resulting in larger clusters. The procedure was repeated iteratively until all sequences with detectable similarity over at least 50% of their lengths were clustered and aligned together. Starting from this set of clusters, several rounds of the following procedures were performed.

### (ii) Cluster merging and splitting.

PSI-BLAST (125) searches using the cluster alignments to construct Position-Specific Scoring Matrices (PSSMs) were run against the database of cluster consensus sequences. Scores for pairs of clusters were converged to a distance matrix as described above, UPGMA trees were cut using at the threshold depth, and unaligned sequences from the clusters were collected and aligned together. An approximate ML phylogenetic tree was constructed from each of these alignments using FastTree (WAG evolutionary model, gamma-distributed site rates). The tree was split into subtrees to minimize paralogy and maximize species (genome) coverage. Formally, for a subtree containing *k* genes belonging to *m* genomes (*k* ≥ *m*) in the tree with the total of *n* genomes (*n* ≥ *m*) genomes, the “autonomy” value was calculated as (*m*/*k*)(*m*/*n*)(*a*/*b*)^1/6^ (where *a* is the length of the basal branch of the subtree and *b* is the length of the longest internal branch in the entire tree). This approach gives an advantage to subtrees with the maximum representation of genomes and the minimum number of paralogs and that are separated by a long internal branch. In cases in which a subtree with the maximum autonomy value differed from the complete tree, it was pruned from the tree and recorded as a separate cluster, and the remaining tree was analyzed again.

### (iii) Cluster cutting and joining.

Results of PSI-BLAST searches whereby the cluster alignments were used as PSSMS and run against the database of cluster consensus sequences were analyzed for instances where a shorter cluster alignment had a full-length match to a longer cluster containing fewer sequences. This situation triggered cutting the longer alignment into fragments matching the shorter alignment(s). The alignment fragments were then subjected to the merge-and-split procedure described above. If the fragments of the cluster that was cut did not merge into other clusters, the cut was rolled back, and the fragments were joined.

### (iv) Cluster mapping and realignment.

PSI-BLAST searches performed using the cluster alignments as PSSMs were run against the original database. Footprints of cluster hits were collected, assigned to the respective highest-scoring query clusters, and aligned, forming the new set of clusters mirroring the original set.

### (v) Postprocessing.

The PIM group clusters were manually curated and annotated using the NCVOG, CDD, and HHPRED matches as guides. For the NCLDV clusters, the final round clusters with strong reciprocal PSI-BLAST hits and with compatible phyletic patterns (using the same autonomy value criteria as described above) were combined into clusters of homologs that maximized genome representation and minimized paralogy. The correspondence between the previous version of the NCVOGs and the current clusters was established by running PSI-BLAST with the NCVOG alignments as PSSMs against the database of cluster consensus sequences.

### Genome similarity dendrogram.

Binary phyletic patterns of the NCLDV clusters (where 1 indicates the presence of the given cluster in the given genome) were converted to intergenomic distances using the equation *d_X_*_,_*_Y_* = −log[*N_X_*_,_*_Y_*/(*N_X_N_Y_*)^1/2^], where *N_X_* and *N_Y_* are the numbers of COGs present in genomes *X* and *Y*, respectively, and *N_X_*_,_*_Y_* is the number of COGs shared by these two genomes. A genome similarity dendrogram was reconstructed from the matrix of pairwise distances using the neighbor-joining method (126).

### Conserved motif search.

The sequences from the LCV genomic bins were searched for potential promoters as follows. For every predicted ORF, upstream genome fragments (from 250 nucleotides upstream to 30 nucleotides downstream of the predicted translation start codons) were extracted, short fragments (i.e., those with fewer than 50 nucleotides) were excluded, and the resulting sequence sets were searched for recurring ungapped motifs using MEME software, with the motif width set to 25, 12, or 8 nucleotides (127). The putative LCV virophage promoter was used as a template to search upstream fragments of LCMiAC01 and LCMiAC02 with the FIMO online tool (127). The motifs were visualized using the Weblogo tool (128).

### Additional supplemental material.

More supplemental material can be found at ftp://ftp.ncbi.nih.gov/pub/yutinn/Loki_Castle_NCLDV_2018/.

### Data availability.

The metagenomic nucleotide sequence bins analyzed in this work are available in GenBank under the accession numbers MK500278-MK500613 (BioProject PRJNA504765).

Raw sequence reads have been deposited to the NCBI Sequence Read Archive repository under BioProject PRJNA504765. Whole Genome Shotgun projects for metagenome assemblies KR126, K940, K1000, and K1060 have been deposited at DDBJ/ENA/GenBank under the accession numbers SDBU00000000, SDBV00000000, SDBS00000000, and SDBT00000000, respectively. The versions described in this paper are versions SDBU01000000, SDBV01000000, SDBS01000000, and SDBT01000000. The NCLDV genome bins analyzed in this work are available in GenBank under the accession numbers MK500278-MK500613.
