# Supplementary material for: Virus Genomes from Deep Sea Sediments Expand the Ocean Megavirome and Support Independent Origins of Viral Gigantism
Source: mBio. 2019 Mar 5;10(2):e02497-18. doi: 10.1128/mBio.02497-18 (PMC6401483; doi:10.1128/mBio.02497-18)
Supplement: TEXT S1 [file mBio.02497-18-s0001.pdf]

**Table of contents**

**Supplementary methods**

ESOM binning
Differential coverage binning (DC)
Co-assembly binning (CA)
Mmgenome bin refinement
Nucmer alignments
Repeats analysis

**Supplementary results**

Bin refinement examples
Examples 1-6

**Supplementary figures**

Figure S1. ESOM binning
Figure S2. Screening result
Figure S3-S18. Binning example figures
Figure S19-S20. Phylogenies of marker genes showing signs of contamination

**Supplementary tables**

Table S1. Sample information
Table S2. DNAP sequences for screening
Table S3. Megavirales reference genomes for reads profiling
Table S4. Marker NCVOGs
Table S5. DC bins before and after reassembly
Table S6. DC bins compared to CA bins

**ESOM binning**
ESOM (emergent self-organizing maps), is a composition-based binning method that clusters sequences according to tetra-nucleotide frequencies (TNF). Contigs are first cut up into smaller pieces (minimum 5kb, maximum 10kb or minimum 10 kb, maximum 20kb), since the average TNF signal will be less accurate for longer sequences. The ESOM program will then draw a map representing each contig as a dot, with ”islands” that represent sequences with similar TNF. The sequences within one ”island” are manually selected and extracted as a raw bin (Figure S1).
Megaviral ESOM bins were detected by using BLASTP to screen for contigs containing Megaviral DNA polymerase family B (DNAP, NCVOG0038). Contigs containing the DNAP were highlighted on the ESOM maps for each Metagenome, and bins were extracted if the sequences were clustering in a clear island. Lists of the contigs in each ESOM bins were used to refine the differential coverage CONCOCT bins in Mmgenome.

**Differential coverage binning (DC)**
The principle behind differential coverage binning is based on the assumption that the species of interest is present in several samples. Assuming that the whole genome will have similar reads coverage depending on how abundant the species is in each sample, sequences that have the same co-variance in coverage across samples are seen as belonging to the same bin.

CONCOCT binning is based on both coverage and composition, but does not take differential coverage into account. In our experiences, raw CONCOCT bins tend to be inflated and include many false-positive contaminating contigs.
By mapping the reads of each sample onto each metagenomic assembly, the differential coverage can be visualized in Mmgenome and be used for bin refinement (Albertsen et. al. 2013).

**Co-assembly binning (CA)**
The bins extracted during DC binning were used as an input for reads profiling, together with ten Megaviral reference genomes (Table S2). Reads matching the profile were extracted from all three metagenomes, and different assembly parameters were tried with SPAdes (single cell mode, multi cell mode, careful mode). BLASTP was used to find the contigs containing the bin Megaviral DNAP, and the assembly which had longest contigs on average containing the DNAPs was chosen for CONCOCT binning (table S4). In some cases longer contigs were present in the other assembly conditions, and might have yielded higher quality bins for some of the genomes, but because of time constraints it was not feasible to do CONCOCT binning with all assemblies.

CONCOCT binning was done with the three different size thresholds: 2kb, 3kb, and 5kb, and bin statistics were compared. The 5kb size threshold bins had overall more bins containing only one marker DNAP per bin, so it was chosen for Mmgenome refinement (not shown).

**Mmgenome bin refinement**

Below I will describe the general refinement steps done in Mmgenome, and highlight examples that illustrate how the binning process was not a one-fit-all pipeline. The cases are: The few DC bins that had higher quality than their CA bins (example 1 and 3), bins that were binned into individual or mixed CONCOCT bins (example 3), cross-clade mixed bins (example 4), within clade mixed bins (example 2, example ), and closely related bins from different samples that assembled into single genomic bins during CA binning (example 5).

In Mmgenome, first the GC content and coverage of each sample were plotted against each other. Data from the 2kb, 3kb, and 5kb contig size thresholds was compared, and the first step was to pick the plot with clearest clusters for sub-binning. Secondly, marker NCVOGs, reads linkage, and ESOM binned contigs (if overlapping with the CONCOCT bins) were visualized on the chosen plot, and clusters corresponding to individual genomes were extracted.

Bin statistics were compared before and after the sub-binning steps, such as: number of contigs, total size, and marker gene completeness and redundancy. The sub-binning was tested several times with different contig size and plots, comparing bin statistics afterwards to make a decision on which bins to move forwards with. In some cases further sub-binning was done in order to get rid of outlier contigs and reduce redundant marker genes. Sub-binning was stopped if completeness was reduced, and in the end the bin contigs were extracted. This was followed by a BLASTP based Taxonomic filtering: manual removal of contigs with genes that had no viral or eukaryotic BLASTP hits, and 50% or more bacterial or archaeal hits.

**Nucmer plots**

Nucmer alignment plots comparing the CA with the DC bins show how they differ in terms of contig number, and contig size. They also show where the assembly algorithm has made different decisions on which sequences to assemble together. With this kind of data it's not possible to determine which assembly that is the more "correct one". In many cases the presence of repeats on the end of contigs correlate with contig breaks, see repeats dot plots below.

**Repeats analysis**

Repetitive nucleotide sequences are common in some Megavirales, especially Pithoviruses (Legendre et. al. 2014). Repeats often cause fragmentation of the assembly, since assembly programs break the contigs where they can't resolve the repeats. Repeat analysis was performed by generating dot plots using Nucmer (MUMmer3.23, (77)), with the maxmatch --nosimplify options. The different Loki's Castle NCLDV bins have different patterns of repeats, shown as dot plots (Additional file 6). Every time a repeat is present between or within contigs it's represented by a dot, and the color of the dot (red or blue) show the directionality of the repeat (same direction or reversed).

## Supplementary results

### Bin refinement examples

A single best binning procedure for all bins was not feasible, since the genomes did not behave in the same way, which is illustrated in the examples below. In a few cases refinement was straightforward, with only one genome present per bin (Example Figure S7), or several genomes with clear clustering (Figures S3, S4, S11, S15), but in many cases bins were mixed with closely related genomes or genomes belonging to different clades (Figures S5, S8, S9, S10, S14, S18). Only one bin out of the 29 DC bins came out as one contig after reassembly: LCMAC102 (395459 nt) (Example 1).

19 of the CA bins had higher quality than the DC bins, and two of the DC bins (LCMAC102, LCPAC304) had higher quality than the CA bins (Example 1 and 3). Two pairs of the differential coverage bins co-assembled into one higher quality bin (LCPAC104+105, and LCPAC404+405, Example 5 and 6). Four of the bins were inflated in both cases and were discarded. A comparison of the DC and CA bins is summarized in Table S6.

### Example 1: LCMAC102

LCMAC102 is an example of one of the bins that were assembled better in the DC binning. The 3kb size threshold was picked for DC binning. It contained 30 contigs, totaling 1160644 nt, with a longest contig of 190364 nt. The K1000 coverage vs K1060 coverage plot was picked for sub-binning, and the DNAP containing contig was highlighted. Two clearly separated clusters were visible. Both clusters were extracted and checked for completeness. The upper cluster contained only three marker NCVOGs and was discarded. The lower cluster contained all 10 marker NCVOGS, and had 6 contigs that assembled into one single contig during reassembly (Figure S3).

In CA binning, the LCMAC102 DNAP containing contig was binned together with LCMAC101 and LCDPAC01. It didn't assemble into a single contig, but into the 169935 nt long contig with the DNAP marker, which clusters with a shorter contig in the Mmgenome coverage vs GC plot (Figure S4). Since a complete genome was retrieved in DC binning, and other bins needed more attention, it was not refined from the CA mixed bin.

### Example 2: LCMAC101

Despite being closely related, the LCMAC101 and 102 bins are very different in size. Since LCMAC101 is much larger than the closely related LCMAC102, and only the two longest contigs contain marker NCVOGS, it is a concern that several genomes might have been binned together. However, the bin looks very similar both in the CA and DC binning, and none of the marker NCVOGs are redundant. Repeats analysis show that there are repeats present on the ends of most contigs in LCMAC101, and I speculate that this might have something to do with why the genome is larger than for LCMAC102, which has no repeats (Additional file 6)

In the DC binning, LCMAC101 was binned out through two sub-binning steps. First it was found in a bin containing two different DNAP. The LCMAC101 DNAP was found in a clearly separate cluster in the K1060 coverage vs GC% plot. The first sub-bin for LCMAC101 had 24

contigs, and was 831595 nt. It did however contain a redundant RPB1 copy, which was identified as an outlier and culled in the second sub-binning step, resulting in 18 contigs that reassembled into 7 contigs (Figure S5). The additional DNAP in this bin was clustering in a diffuse cluster that contained too many small contigs to be cleaned (89 contigs, 1031603 nt). In the DNAP diversity phylogeny it's the DNAP sequence that branches closest to the LCMAC101 sequence, showing that there is more diversity of this clade (Figure S2).

LCMAC101 could be extracted from the CA bin as a clearly separate cluster containing 7 contigs (763048 nt in total, Figure S4). There were no big differences between the CA and DC bins (Table S6), but the CA bin was picked since it had overall the longest contigs than the DC bin (Figure S6).

**Example 3: LCPAC304**

LCPAC304 was the second case when the DC bin was better than the CA bin. The DC Mmgenome plot had clear clustering, and some outlier contigs could be removed (Figure S7). In the CA binning, LCPAC304 binned together with LCPAC303 and they could not be separated (Table S6).

**Example 4: LCPAC101 and LCMiAC01, and LCMiAC02**

This example was chosen because it shows how the DC binning resulted in an across-clade mixed bin, which was resolved by CA binning. LCPAC101 it was binning together with LCMiAC01 and 02 in the DC binning. The 2kb plot with coverage of K1000 and K1060 was chosen for cluster isolation. From the DNAP phylogeny it was clear that the DNAP sequences were related to Pithoviruses and Mimiviruses, and there was one ESOM bin overlapping with each. This led to a Klosneuviral contig with DNAP and MCP being extracted with the LCPAC101 bin (Figure S8), which was seen in phylogenies in the bin quality checking step (not shown).

In the CA binning, the Klosneuviral LCPAC101 sequences binned together with the LCMiAC bins, and the *bona fide* LCPAC101 sequences binned together with LCPAC102 in clearly separate clusters (Figure S9), showing how CA binning resolved the across-clades mixing problem of the DC bins.

**Example 5: LCPAC104-105**

This example shows how genomic bins belinging to a species that were present, in high enough abundance, in more than one of the samples were assembled into a single bin in the CA binning. One of the cases was the bins of LCPAC104 and 105, the other was LCPAC405 and 406 (described in example 6).

During DC binning for LCPAC104, both the K1000 coverage vs K1060 coverage, and the K1060 vs GC plots were checked. It was possible to isolate a cluster with lower redundancy in the K1000vsK1060 plot, but overall the clustering was vague (Figure S10). The number of contigs was high, but further sub-binning led to decrease in completeness.

LCPAC105 was DC binned with LCDPAC01, in two clearly separate clusters in the K1000 coverage vs K1060 coverage plot. As seen in figure S11, the plots for 3kb and 5kb were similar. After sub-binning the 3kb bins were chosen because they had highest completeness.

In the CA binning, LCPAC104 and LCPAC105 assembled into one bin with only six contigs. Two of the contigs were removed as contamination because they had only hits to bacteria and archaea (Figure S12). The co-assembled bin is much smaller than the DC-LCPAC104 bin, which was inflated and hard to clean (Table S6).

Despite having identical marker genes, the size of DC-LCPAC104 was almost three times larger than DC-LCPAC105 (611 kb, vs 262 kb). It would not be surprising if the LCPAC104 genome is different than LCPAC105 because they were found at different sediment depths, but a three times size difference seems unlikely. Completeness is lower in the CA-LCPAC104-105 bin, but redundancy was reduced. It's possible that some of the marker genes could have diverged slightly, and thus not co-assembled, so the CA-LCPAC104-105 should perhaps be seen as more of an intersection between the two genomes than a near-complete genome.

**Example 6: LCPAC4 bins**

This example shows a case within-clade mixed bin, where the genomes from one clade were binned into the same CA-CONCOCT bin. Because of this separation was much easier by CA binning than DC binning (which produced several mixed bins).

LCPAC401, LCPAC403, and an unclassified bin without DNAP (later LCPAC206) were binned in the same DC bin. There was an ESOM bin for LCPAC401. The 5kb size threshold was checked first, but the cluster for the LCPAC401 ESOM bin had high redundancy (13 extra marker genes). In the 3kb size threshold three separate clusters were visible (Figure S13). Two clusters containing a DNAP could be extracted (LCPAC401 and LCPAC403), and one lacking DNAP (originally labelled LCUCMV," Loki's Castle Un-Classified MegaVirus").

LCPAC404 and 402 were in the same DC bin, and had ESOM bins. The clustering was quite good for 404, but some of the ESOM binned contigs were drawn into the looser 402-cluster (Figure S14). LCPAC405 was in its own DC bin, but inflated compared to the closely related 404 (not shown).

In the CA binning all LCPAC4 were binned into the same bin (Figure S15). LCPAC403 was assembled into only 6 contigs. LCPAC404 and 405 co-assembled into the same bin, similar to LCPAC104-105. LCPAC402 was a loose cluster with many short contigs and was discarded. LCPAC401 binned in a cluster with an additional DNAP. Could this be the DNAP that was missing in the DC bin LCUCMV? To find out, Nucmer alignments of the CA vs the DC bins were used, to see which of the contigs that belonged to the separate DC bins (Figure S16-S17.The contigs belonging to each of the two bins could be separated manually this way, even though they were too similar in coverage and composition to make separate clusters in Mmgenome (Figure S18).

## Supplementary figures

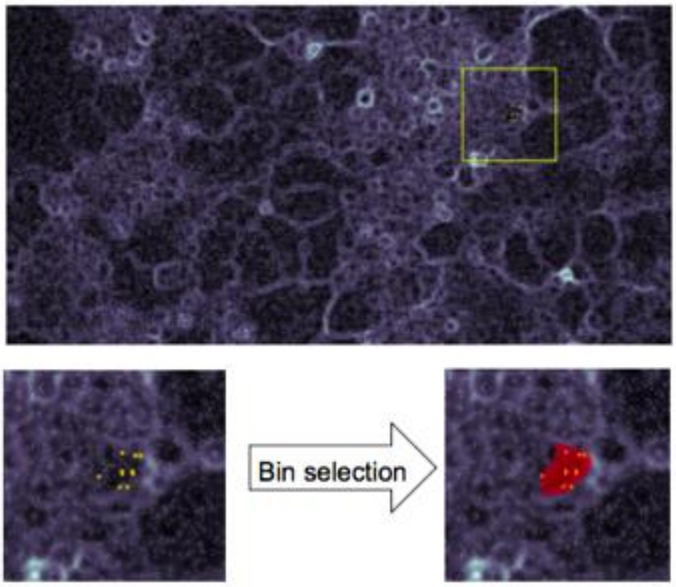


*Figure S1. Schematic of the ESOM binning process. Contigs were split into fragments, in this case with min size 10 kb in intervals of 10 kb, and clustered together by tetra-nucleotide composition. The location of fragment of the contigs containing NCLDV marker genes were visualized on the ESOM (yellow), and contigs putatively belonging to the same genome were marked and extracted as a bin (red).*

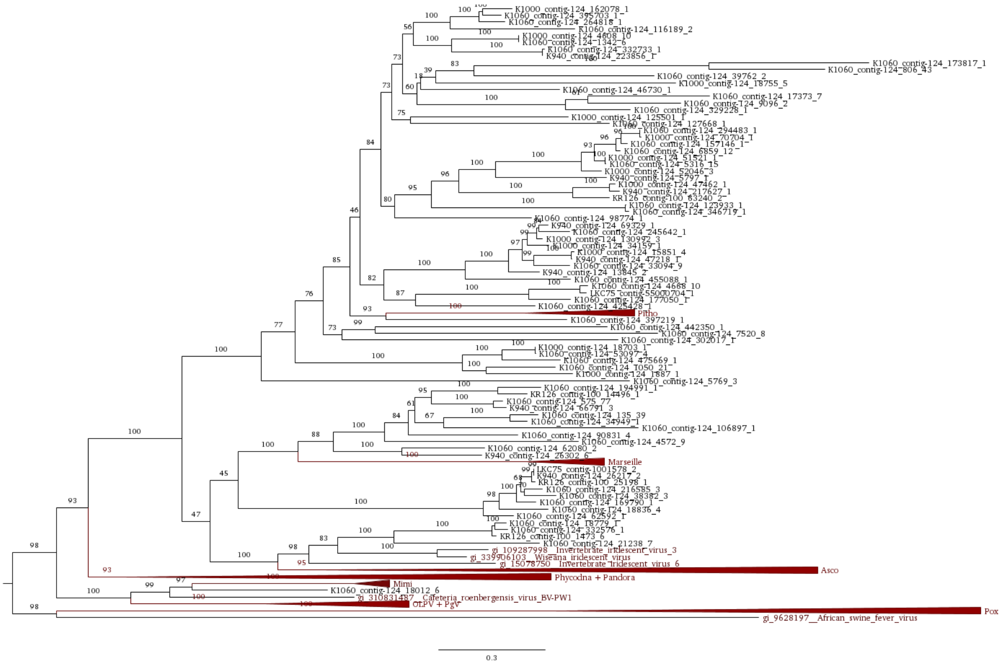


*Figure S2. Maximum likelihood phylogeny of the DNA polymerase protein sequence found when screening for Megavirales sequences in metagenomes from Loki's Castle.*

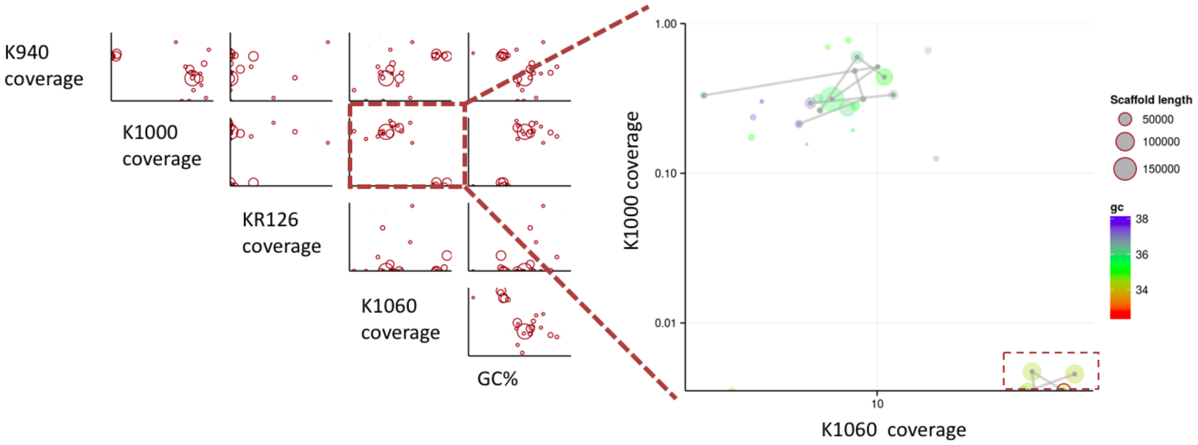


*Figure S3. DC Mmgenome plots for LCMAC102. On the right, the DNAP containing contig is marked with a red circle.*

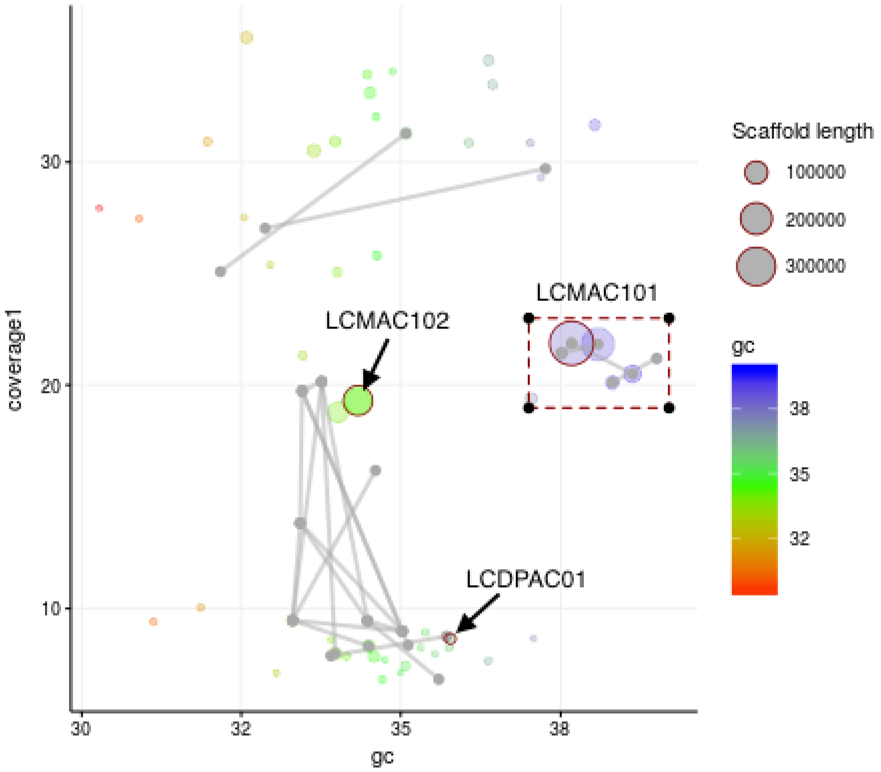


*Figure S4. DC Mmgenome plot of the LCMAC101, LCMAC102 and LCDPAC01 CONCOCT bin. DNAP containing contigs are marked with red circles.*

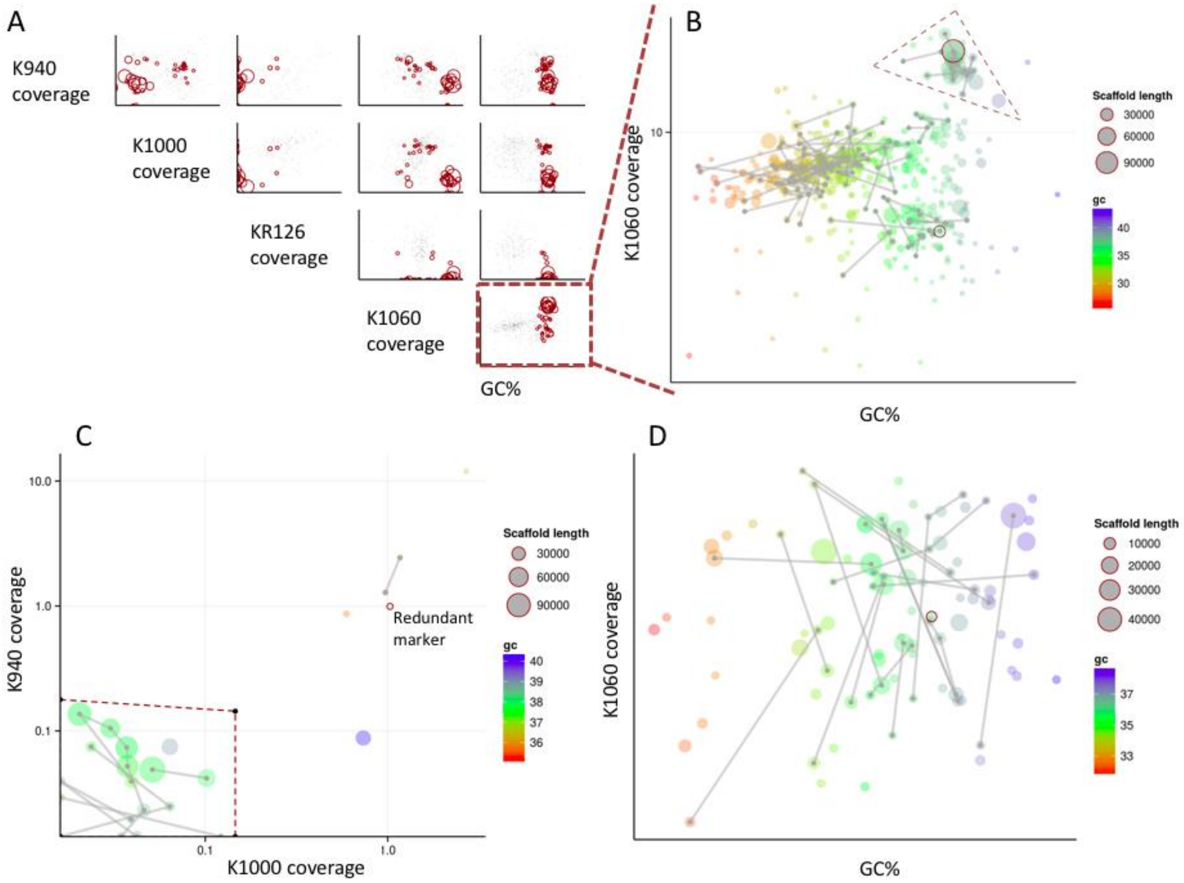


*Figure S5. DC Mmgenome plots for LCMAC101. A) ESOM binned contigs are highlighted. B) DNAP-containing contigs are highlighted. C) LCMAC101 sub bin, with a contig containing a redundant marker highlighted. D) The sub-bin containing an additional DNAP, which was discarded because it had too many contigs to clean.*

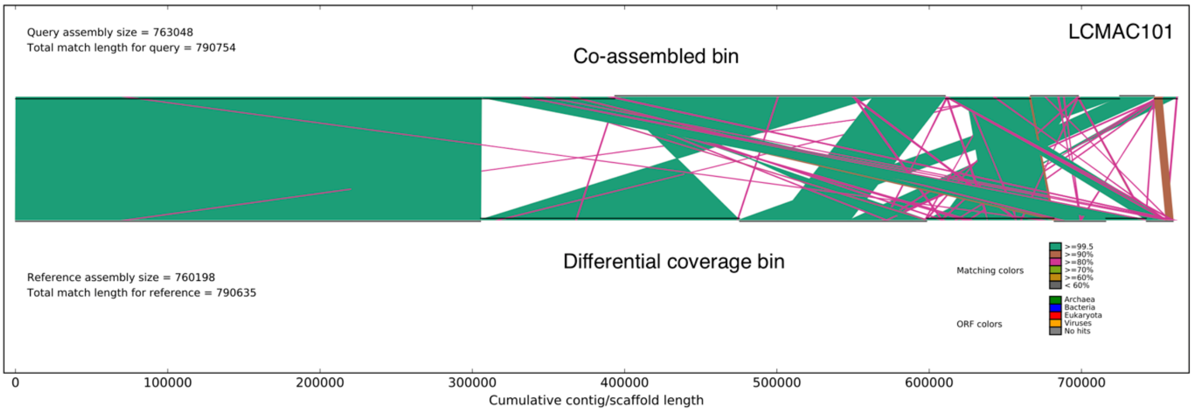


*Figure S6. LCMAC101 alignment plot.*

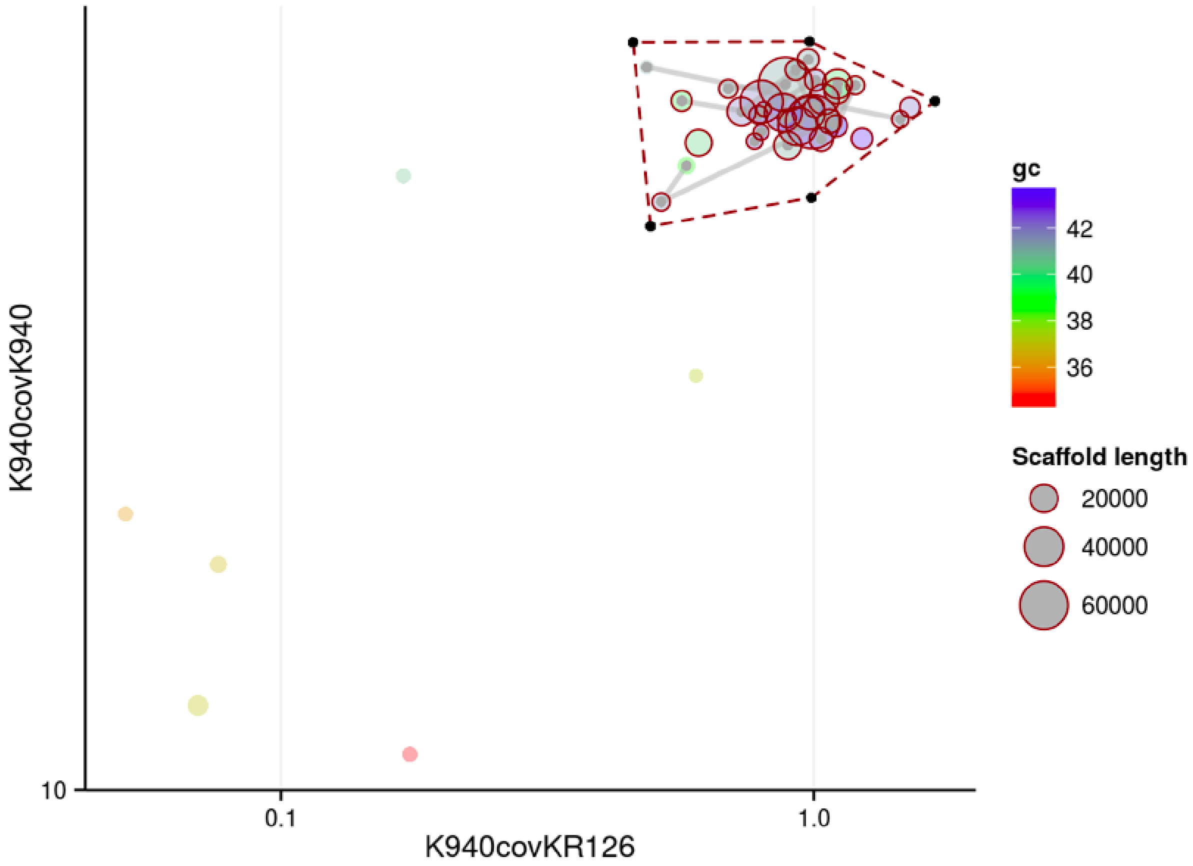


*Figure S7. DC Mmgenome plot for LCPAC304. ESOM binned contigs are highlighted.*

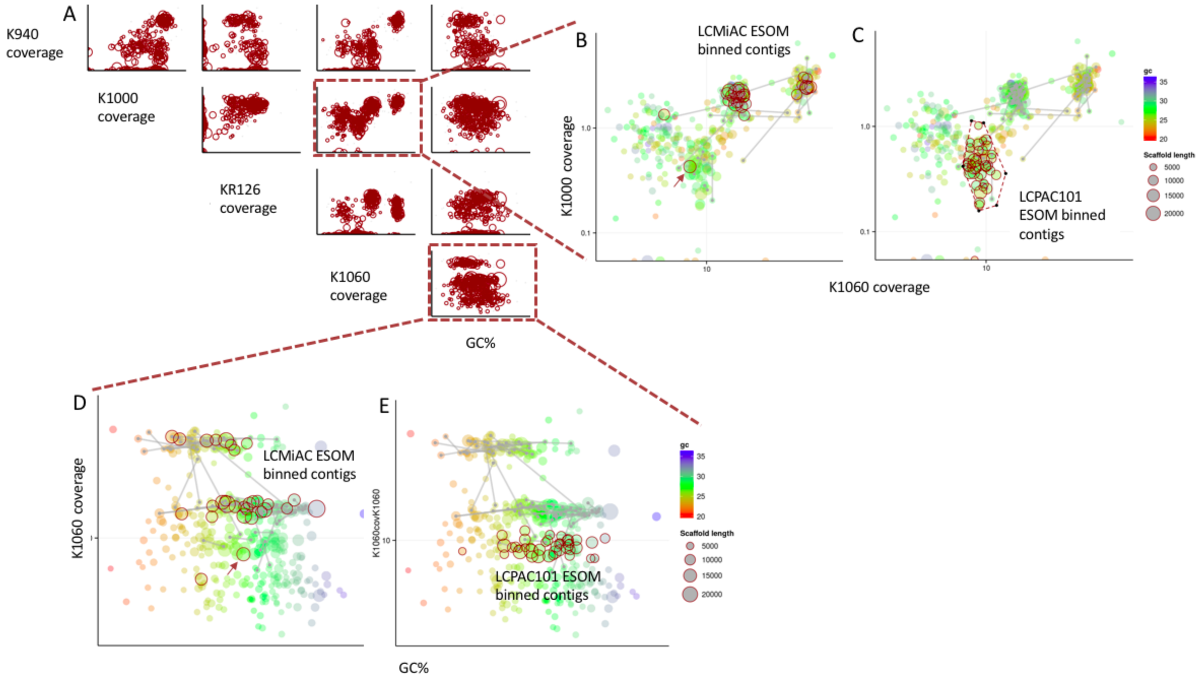


*Figure S8. DC Mmgenome plots for the LCPAC101-LCMiAC mixed bins. A) The coverage and GC% across all metagenomes. B,C) K1000 coverage vs K1060 coverage plots with ESOM contigs for the LCMiAC and LCPAC bins highlighted. Two clusters were extracted. The arrow in B points to an outlier Klosneuviral contig that ended up in the LCPAC101 bin. D-E) K1060 vs GC% plot that was also looked at, but not picked for sub-binning. ESOM contigs for the LCMiAC and LCPAC bins are highlighted.*

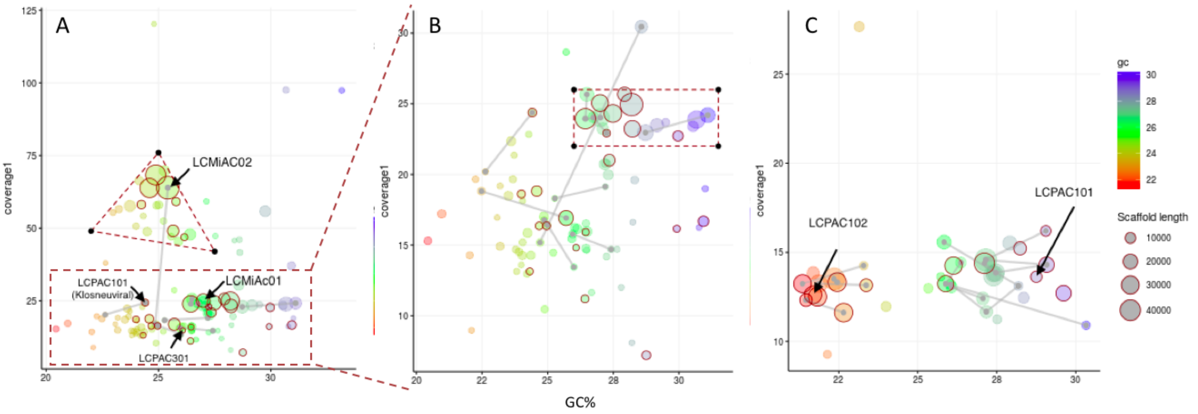


*Figure S9. CA binning plots for LCPAC101 and LCMiAC bins. A) The bin used for isolating LCMiAC01 and 02. NCLDV markers are highlighted, and arrows point to contigs containing DNAP that were identified in the DC bins. The dashed triangle shows contigs extracted as LCMiAC02, and the dashed box shows the area used for LCMiAC01 sub-binning. B) The LCMiAC01 sub-bin. NCLDV markers are highlighted, and the dashed box shows contigs extracted as LCMiAC01. C) The bin containing LCPAC101 and LCPAC102. NCLDV markers are highlighted. Arrows mark the DNAP-containing contigs.*

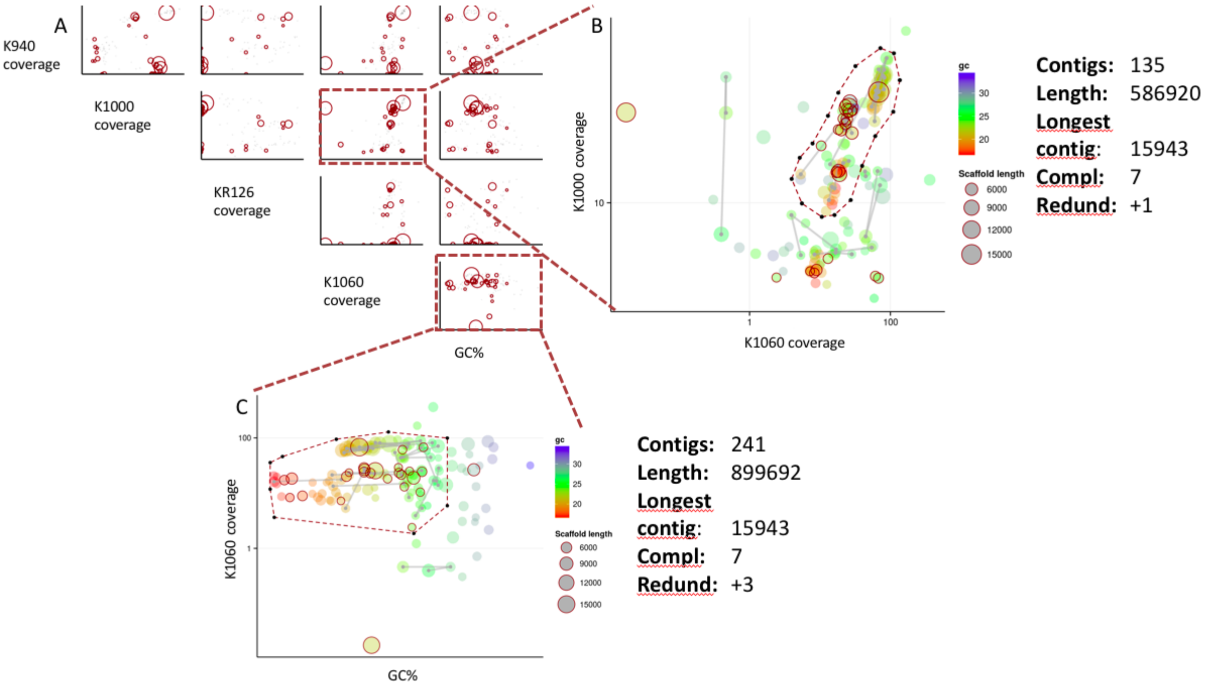


*Figure S10. DC Mmgenome plot for the LCPAC104 bin. Contigs with NCVOGs are highlighted.*

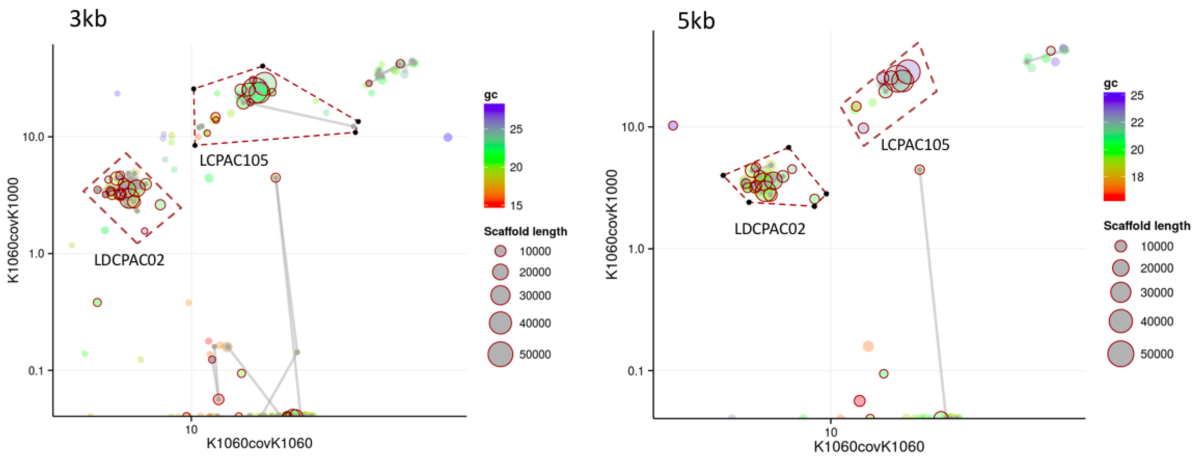


*Figure S11. DC Mmgenome plots for LCPAC105 and LCDPAC02. The K1000 coverage vs K1060 coverage plots with 3kb and 5kb size thresholds are shown. NCVOGs are highlighted with red circles.*

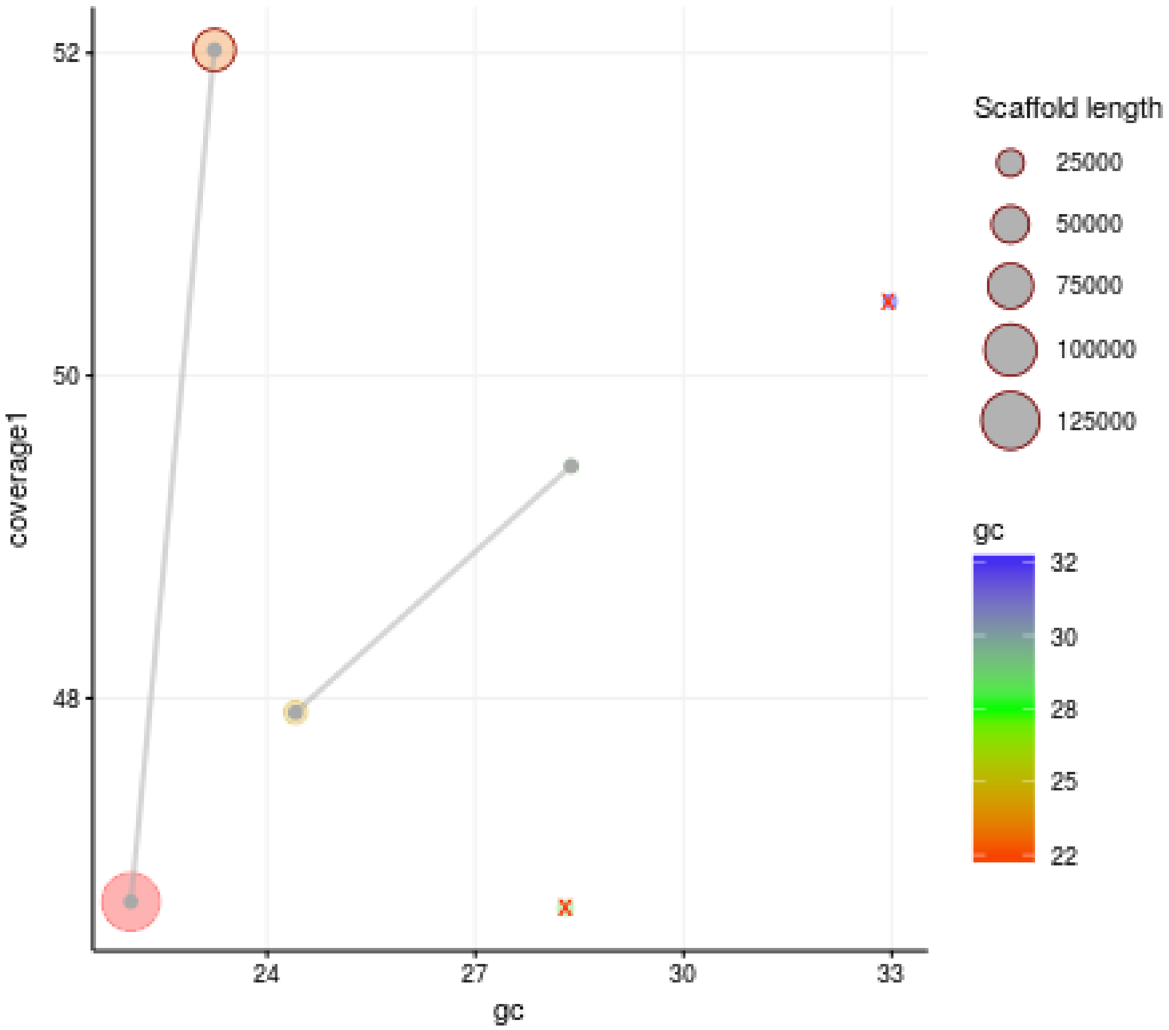


*Figure S12. CA Mmgenome plot for the co-assembled LCPAC104-105 bin. Contigs that were removed in the taxonomic filtering are marked with a red x.*

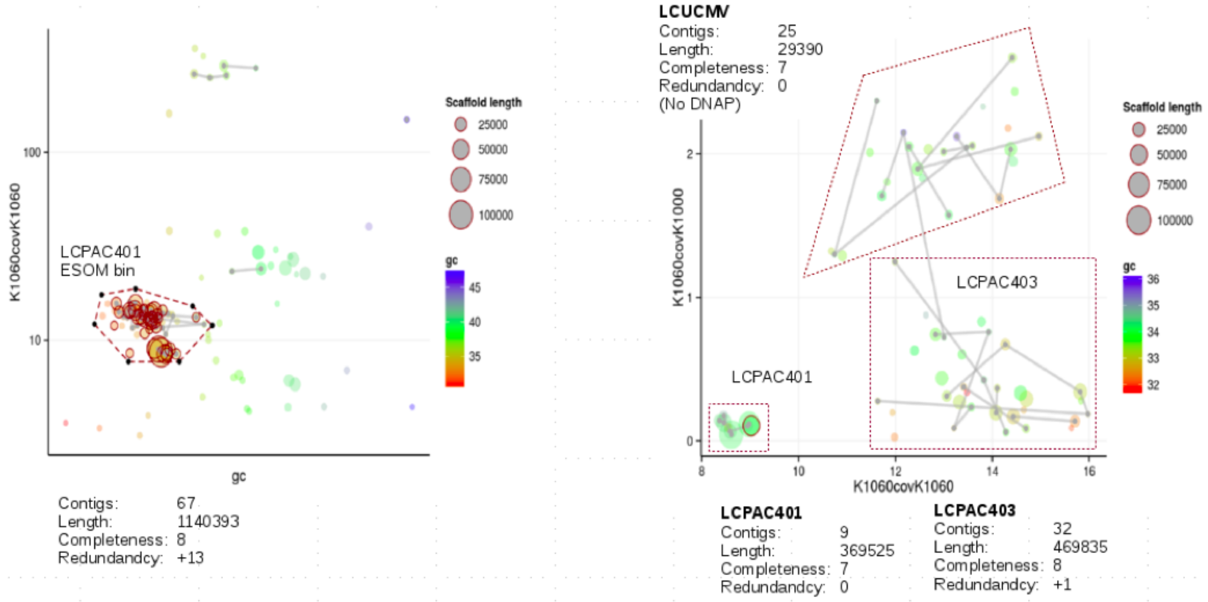


*Figure S13. DC Mmgenome plot for LCPAC401, LCPAC403 and LCPAC406. On the left is the 5kb size threshold plot, with the LCPAC401 ESOM bin contigs highlighted. On the right is the 3kb size threshold plot, showing three clusters that were isolated. The LCPAC401 DNAP is highlighted, and the unlabeled cluster is LCUCMV.*

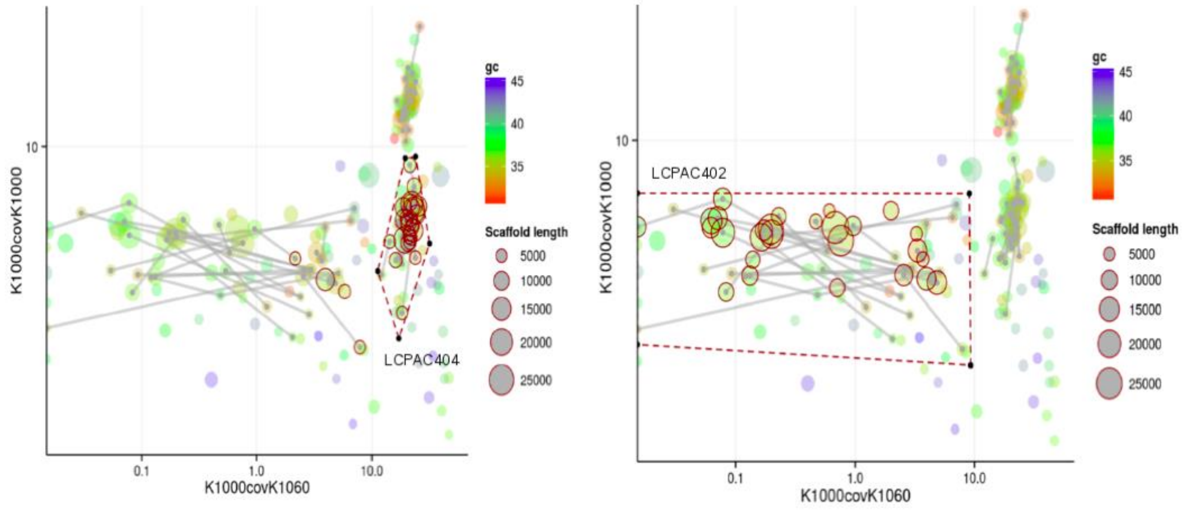


*Figure S14. DC Mmgenome plot for LCPAC404 and LCPAC402. ESOM binned contigs are highlighted, for LCPAC404 (left), and LCPAC402 (right).*

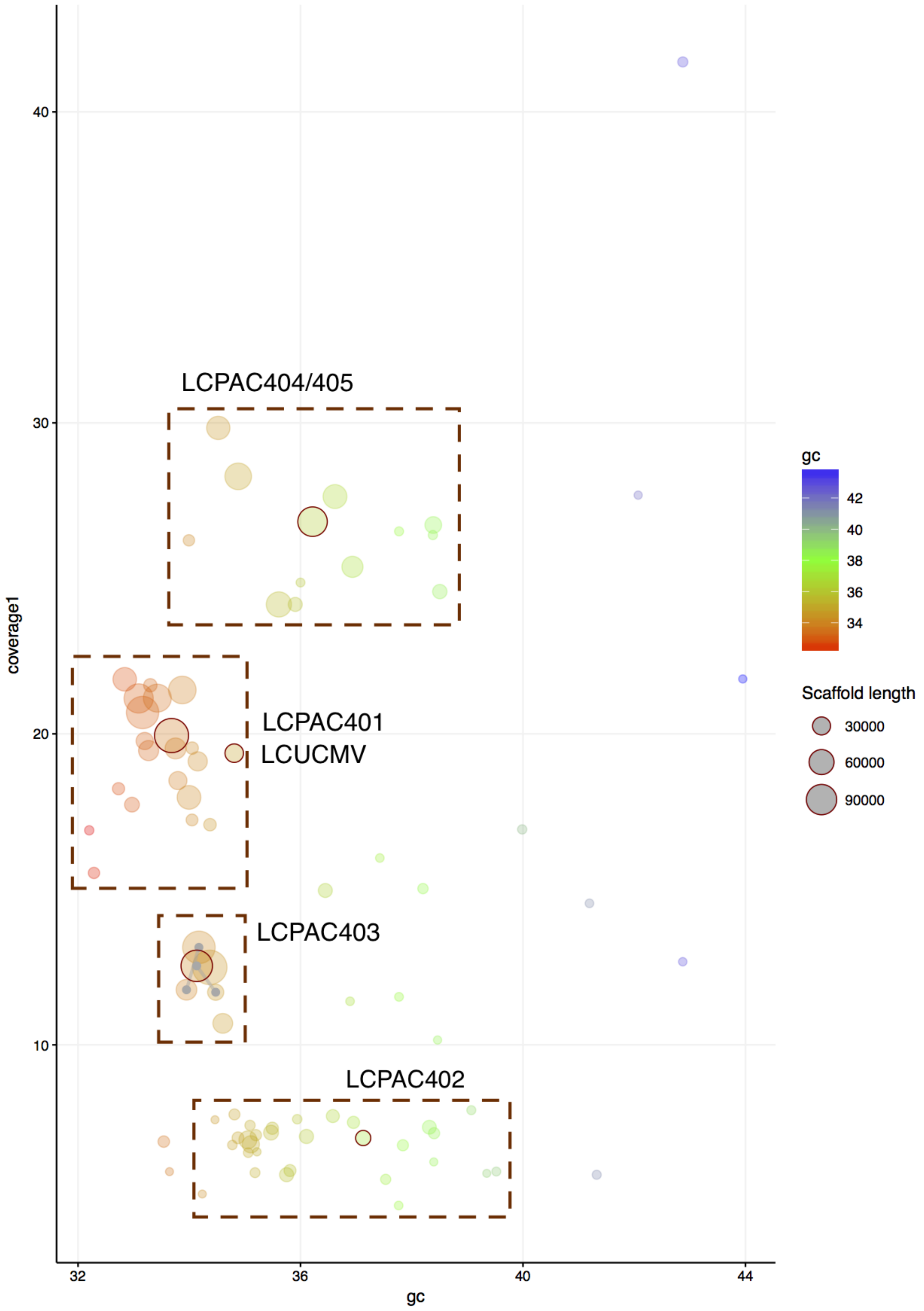


*Figure S15. CA Mmagnome plot for the LCMAC4 bins. DNAP-containing contigs are marked with a red circle.*

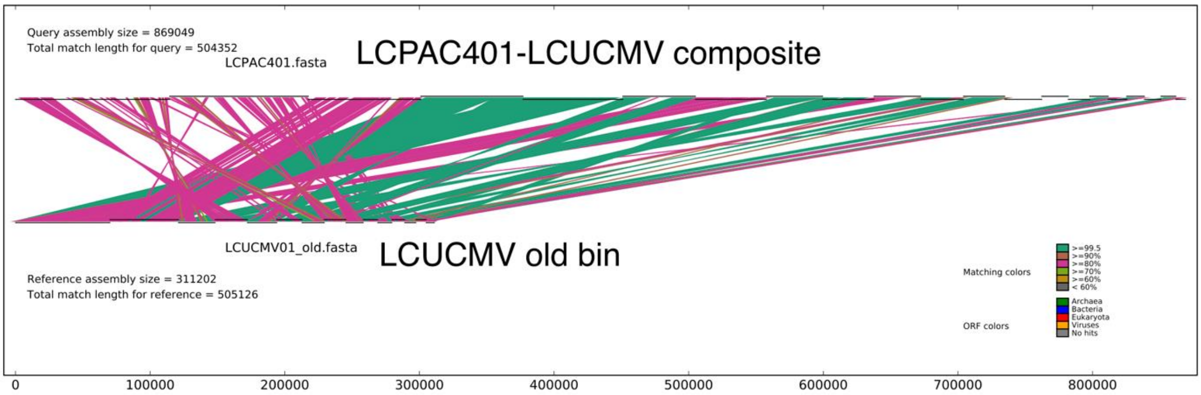


*Figure S16. Nucmer alignment of the DC LCUCMV bin and the CA LCPAC401-LCUCMV composite bin. The colors show how similar the sequences are: green is above 99.5%, brown above 90%, and red above 80%.*

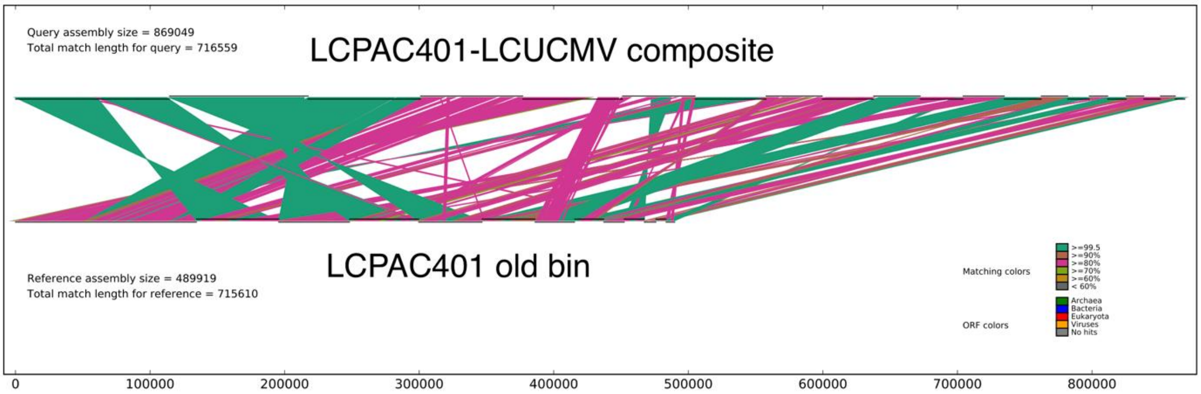


*Figure S17. Nucmer alignment of the DC LCPAC401 bin and the CA LCPAC401-LCUCMV composite bin. The colors show how similar the sequences are: green is above 99.5%, brown above 90%, and red above 80%.*

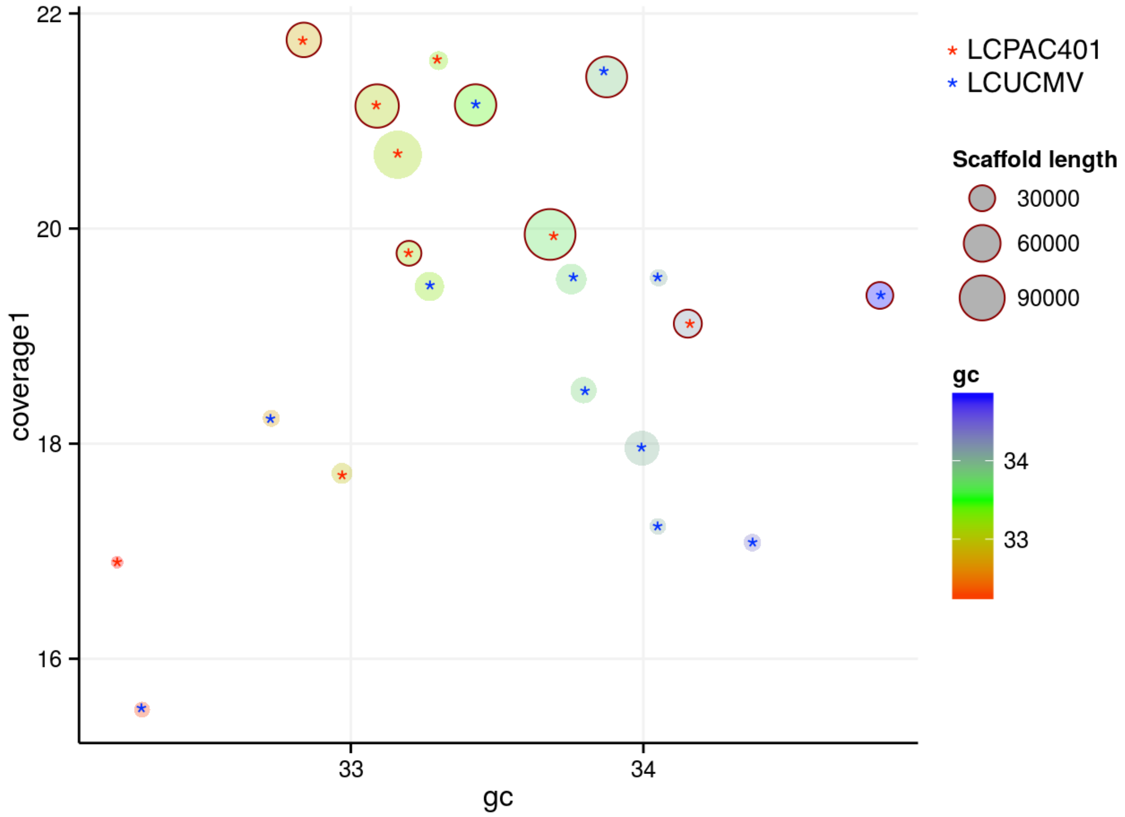


Figure S18. *CA Mmgenome plot for the LCPAC401-LCUCMV composite bin. Contigs containing marker genes are marked with red circles and the contigs assigned to each bin are marked with red (LCPAC401) or blue (LCUVMV/LCPAC406) asterisks.*

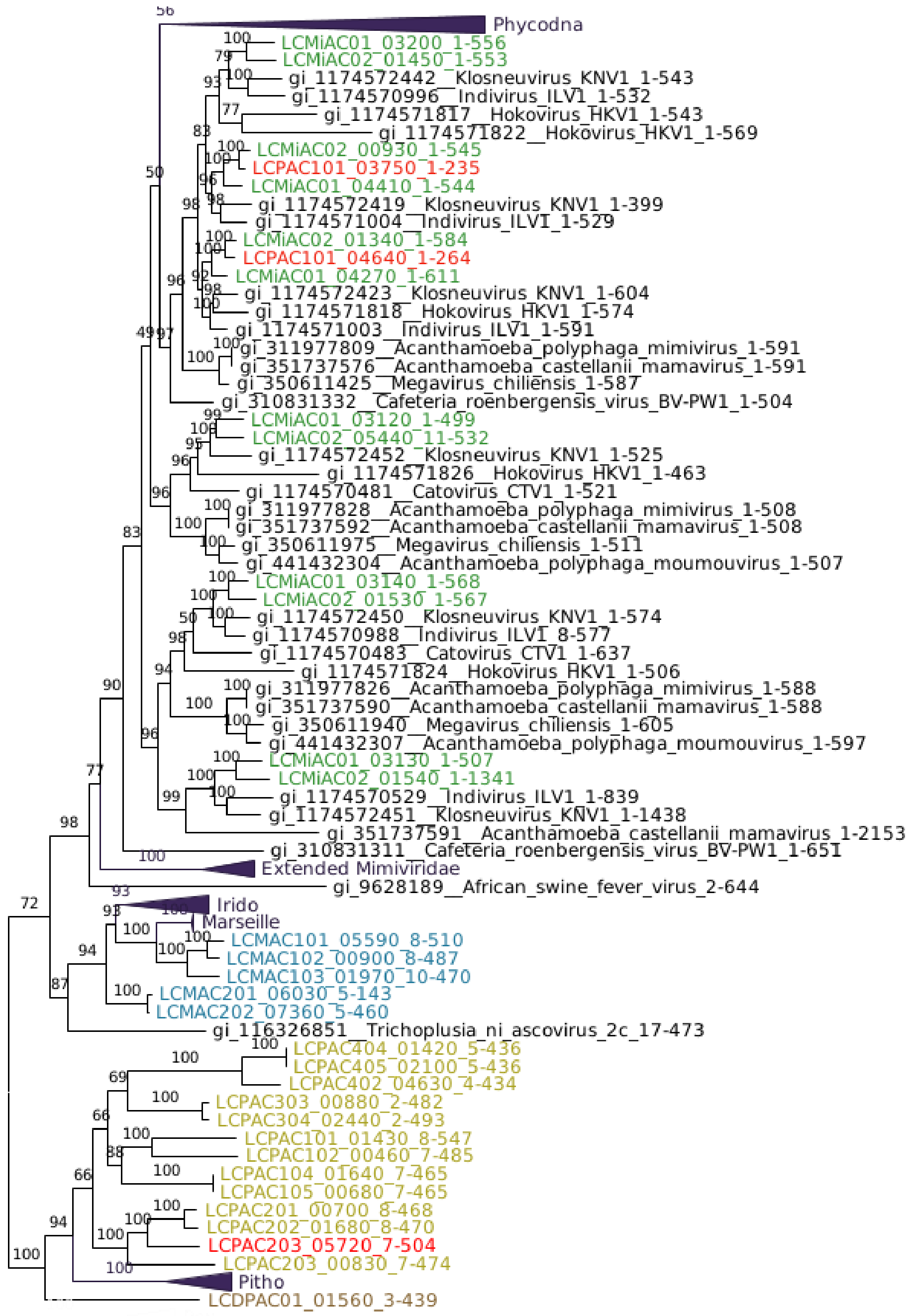


*Figure S19. Maximum likelihood phylogeny of the MCP gene found in the DC bins. Genes highlighted in red show signs of cross-bin contamination.*

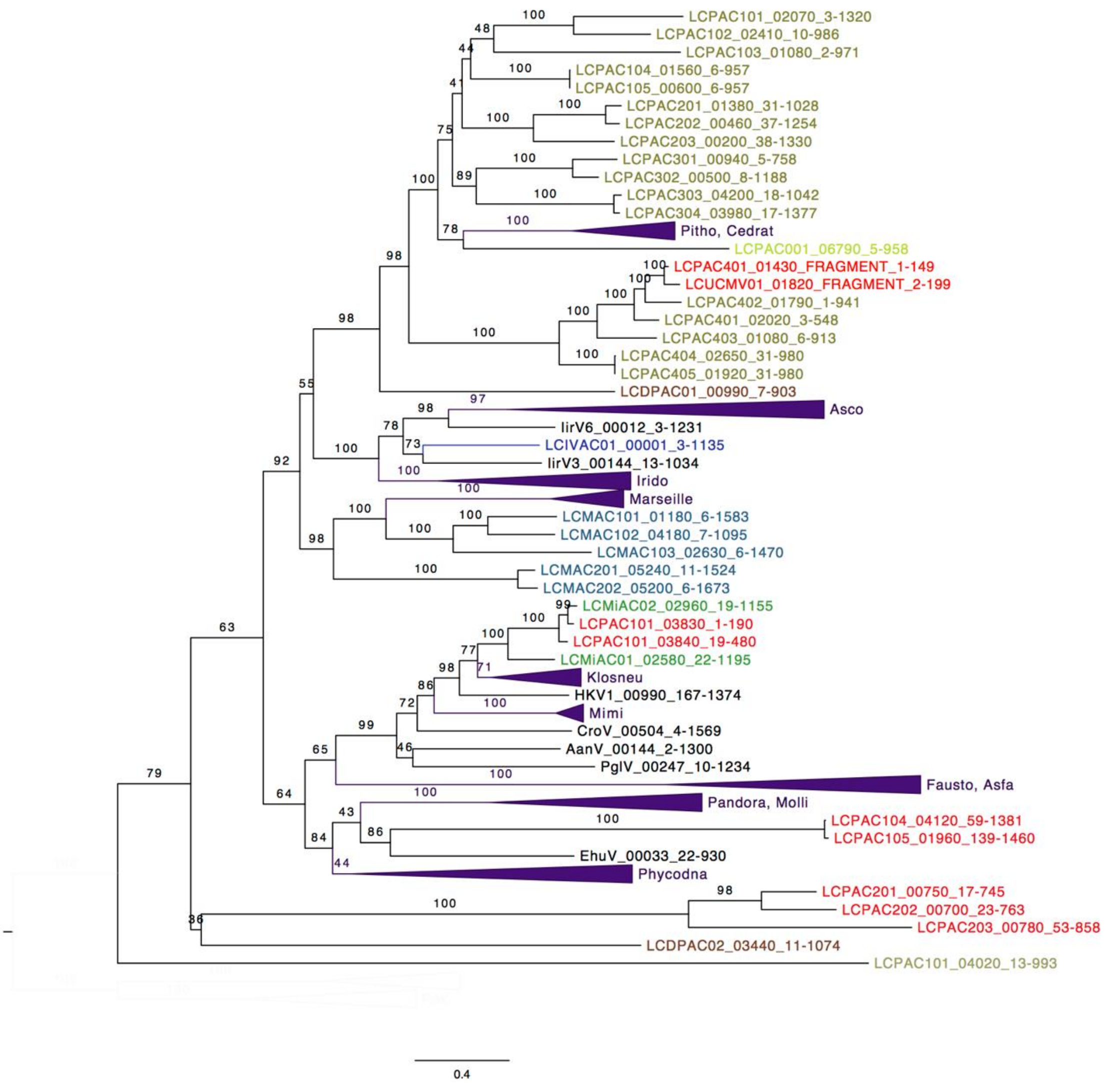


*Figure S20. Maximum likelihood phylogeny of the DNAP gene found in the DC bins. Genes highlighted in red show signs of contamination.*

**Supplementary tables**

Table S1. Sample information, including location, and depth of the sediment samples used for DNA extraction, as well as amount of data generated during sequencing.

| Sample site | Location coordinates | Sediment depth (cm) | Meters below sea level | Assembly name | Sequencing data (Gbp) |
|---|---|---|---|---|---|
| GS10-GC14 | 73.763167N - 8.464000E | 75 | 3283 | LKC75 | 232.3 |
| GS08-GC12 | 73.763333N - 8.463833E | 126 | 3250 | KR126 | 10.1 |
| GS10-PC15 | 73.756333N - 8.455167E | 103 | 3236 | K1060 | 27.0 |
| | | 163 | | K1000 | 50.0 |
| | | 223 | | K940 | 44.0 |

Table S2. DNA polymerase family B sequences used for screening.

| Virus species | Gi number |
|---|---|
| Cedratvirus A11 | 1115261169 |
| Cafeteria roenbergensis virus | 310831487, |
| Acanthamoeba polyphaga mimivirus | 311977705, |
| Megavirus chiliensis | 350611686 |
| Lausannevirus | 327409888 |
| Acanthamoeba castellanii mamavirus | 351737476 |
| Pandoravirus salinus | 516306302 |
| Pandoravirus dulcis | 526119093 |
| Pithovirus sibericum | 585299378 |
| Emiliana huxleyi virus | 73852500 |
| Melbournevirus | 701447420 |

Table S3. Reference genomes used for reads profiling

| **Reference genome** | **Accession numbers** |
|---|---|
| Catovirus | KY684083, KY684084 |
| Hokovirus | KY684103-KY684107 |
| Indivirus | KY684085-KY684102 |
| Klosneuvirus | KY684108-KY684123 |
| Pithovirus sibericum | NC_023423.1 |
| Pithovirus masiliensis | LT598836 |
| Cedratvirus lausanniensis | NC_032108.1 |
| Brazilian marseillevirus | NC_029692.1 |
| Lausannevirus | NC_015326.1 |
| Marseillevirus marseillevirus | NC_013756.1 |

Table S4. Marker NCVOGs used for completeness and redundancy estimate.

| **NCVOG** | **Gene** |
|---|---|
| NCVOG0022 | major capsid protein |
| NCVOG0023 | D5-like helicase-primase |
| NCVOG0038 | DNA polymerase elongation subunit family B |
| NCVOG0076 | DNA or RNA helicases of superfamily II |
| NCVOG0249 | packaging ATPase |
| NCVOG0262 | Poxvirus Late Transcription Factor VLTF3 like |
| NCVOG0271 | DNA-directed RNA polymerase subunit beta |
| NCVOG0274 | DNA-directed RNA polymerase subunit alpha |
| NCVOG0276 | Ribonucleoside diphosphate reductase, beta subunit |
| NCVOG1451 | mRNA capping enzyme |

Table S5. Statistics for the different assemblies tested in the co-assembly. The chosen assembly is marked with green.

| SPAdes parameters | contigs | contigs >1kb | total length | length >1kb | Largest contig | N50 | Longest DNAP contigs |
|---|---|---|---|---|---|---|---|
| Multi-cell mode | 19298 | 3228 | 27246179 | 19274306 | 402618 | 12556 | 5 |
| meta mode | 20364 | 3693 | 26334470 | 18285383 | 367462 | 7871 | 5 |
| Single-cell mode | 26294 | 4900 | 31846578 | 21074751 | 367566 | 5461 | 4 |
| Multi-cell mode, careful | 18897 | 3310 | 27165169 | 19426005 | 393561 | 12487 | 16 |

Table S6. Differential coverage bin statistics before and after reassembly.

| Bin | Before reassembly Contigs | Total length | Longest contig | N50 | After reassembly Contigs | Total Length | Longest contig | N50 |
|---|---|---|---|---|---|---|---|---|
| **LCMAC101** | 18 | 759249 | 116106 | 85244 | 7 | 760198 | 305429 | 170072 |
| **LCMAC102** | 6 | 395694 | 108377 | 105808 | 1 | 395459 | 395459 | 395459 |
| **LCMAC103** | 63 | 757371 | 46952 | 16911 | 65 | 752022 | 41305 | 17546 |
| **LCMAC201** | 53 | 516240 | 26273 | 10959 | 34 | 527121 | 43945 | 19799 |
| **LCMAC202** | 93 | 668569 | 34938 | 8502 | 43 | 739259 | 143245 | 22611 |
| **LCIVAC01** | 31 | 241028 | 16347 | 9317 | 29 | 177022 | 14026 | 6078 |
| **LCMiAC01** | 131 | 785151 | 24171 | 7977 | 74 | 863664 | 59480 | 19465 |
| **LCMiAC02** | 115 | 540843 | 19494 | 5434 | 54 | 647275 | 55280 | 13693 |
| **LCPAC001** | 34 | 561341 | 51654 | 17282 | 58 | 545376 | 60166 | 11403 |
| **LCPAC101** | 82 | 532155 | 17361 | 8456 | 68 | 563977 | 31057 | 10679 |
| **LCPAC102** | 29 | 306926 | 31980 | 13782 | 21 | 306738 | 51048 | 28727 |
| **LCPAC103** | 39 | 223270 | 12852 | 7238 | 29 | 161338 | 11803 | 6050 |
| **LCPAC104** | 125 | 548620 | 15943 | 4894 | 85 | 611073 | 32289 | 8888 |
| **LCPAC105** | 25 | 252924 | 53318 | 34712 | 18 | 261694 | 59935 | 37042 |
| **LCPAC201** | 42 | 502679 | 64234 | 11767 | 22 | 501656 | 166637 | 28783 |
| **LCPAC202** | 46 | 484167 | 31379 | 11425 | 41 | 395648 | 34005 | 9584 |
| **LCPAC203** | 76 | 698337 | 32012 | 12074 | 62 | 708566 | 50947 | 14297 |
| **LCPAC301** | 51 | 178895 | 6668 | 3877 | 41 | 178403 | 10610 | 5161 |
| **LCPAC302** | 87 | 385117 | 12979 | 4681 | 76 | 364120 | 14386 | 5392 |
| **LCPAC303** | 52 | 495788 | 28099 | 14425 | 35 | 509879 | 58727 | 19110 |
| **LCPAC304** | 33 | 622596 | 75053 | 24022 | 12 | 638759 | 173767 | 120110 |
| **LCPAC401** | 32 | 469835 | 34269 | 22831 | 13 | 489919 | 134524 | 52647 |
| **LCPAC402** | 67 | 525421 | 26788 | 11026 | 52 | 512375 | 43066 | 14951 |
| **LCPAC403** | 9 | 369525 | 101352 | 47137 | 14 | 361698 | 69088 | 46461 |
| **LCPAC404** | 46 | 330146 | 19830 | 7347 | 34 | 333578 | 32968 | 12124 |
| **LCPAC405** | 111 | 666898 | 21212 | 6498 | 62 | 715422 | 43487 | 14583 |
| **LCPAC406** | 25 | 293690 | 27193 | 13594 | 15 | 311202 | 70093 | 24004 |
| **LCDPAC01** | 37 | 344355 | 21479 | 12070 | 39 | 279902 | 22099 | 8743 |
| **LCDPAC02** | 41 | 378109 | 31061 | 10397 | 35 | 333796 | 20150 | 13799 |

Table S7. Comparison of bin quality between the two binning methods.

| Bin | Best binning condition | Differential coverage bins | | | | | | Co-assembled bins | | | | |
|---|---|---|---|---|---|---|---|---|---|---|---|---|
| | | Sample | contigs | total size | Longest contig | Complete-ness | Redun-dancy | contigs | total size | Longest contig | Complete-ness | Redun-dancy |
| LCMAC101 | CA | K1060 | 7 | 760198 | 305429 | 10 | 0 | 7 | 763048 | 393561 | 10 | 0.1 |
| LCMAC102 | DC | K1060 | 1 | 395459 | 395459 | 10 | 0 | n.d | n.d | 169935 | 10 | 0 |
| LCMAC103 | CA | K1060 | 65 | 752022 | 389984 | 10 | 0.1 | 9 | 389984 | 69824 | 10 | 0 |
| LCMAC201 | CA | K1060 | 34 | 527121 | 43945 | 9 | 0.1 | 25 | 565697 | 57873 | 8 | 0 |
| LCMAC202 | CA | K940 | 43 | 739259 | 739259 | 10 | 0.1 | 19 | 705352 | 153726 | 9 | 0.1 |
| LCIVAC01 | CA | K1060 | 29 | 177022 | 14026 | 5 | 0.1 | 19 | 198495 | 17223 | 7 | 0.1 |
| LCMiAC01 | CA | K1060 | 74 | 863664 | 59480 | 9 | 0.4 | 18 | 672112 | 85120 | 8 | 0 |
| LCMiAC02 | CA | K1060 | 54 | 647275 | 55280 | 9 | 0.4 | 21 | 642939 | 131456 | 8 | 0.3 |
| LCPAC001 | CA | K1060 | 58 | 545346 | 60166 | 7 | 0.3 | 12 | 249064 | 60499 | 6 | 0 |
| LCPAC101 | CA | K1060 | 68 | 563977 | 31057 | 7 | 0.5 | 26 | 466072 | 46492 | 7 | 0.1 |
| LCPAC102 | CA | K1060 | 21 | 306738 | 51048 | 6 | 0 | 12 | 285593 | 44810 | 7 | 0.2 |
| LCPAC103 | CA | K1060 | 29 | 161338 | 11803 | 8 | 0.1 | 18 | 213370 | 23680 | 8 | 0.1 |
| LCPAC104 | CA | K1000 | 85 | 611073 | 15943 | 7 | 0.3 | 4 | 218903 | 129049 | 6 | 0.1 |
| LCPAC105 | | K1060 | 18 | 261694 | 59935 | 8 | 0.2 | | | | | |
| LCPAC201 | CA | K1060 | 22 | 501565 | 166637 | 8 | 0.1 | 11 | 428611 | 168698 | 6 | 0.2 |
| LCPAC202 | CA | K1060 | 41 | 395648 | 34005 | 8 | 0.3 | 24 | 428280 | 72684 | 7 | 0.3 |
| LCPAC203 | none | K940 | 62 | 708566 | 50947 | 7 | 0.2 | 47 | 712212 | 51371 | 6 | 0.1 |
| LCPAC301 | none | K940 | 41 | 178403 | 10610 | 5 | 0.1 | n.d | n.d | n.d | n.d | n.d |
| LCPAC302 | CA | K940 | 76 | 364120 | 14386 | 6 | 0.3 | 30 | 290561 | 20428 | 4 | 0.1 |
| LCPAC303 | none | K1060 | 35 | 509879 | 58727 | 9 | 0.3 | 64 | 1026844 | 62674 | 8 | 0.1 |
| LCPAC304 | DC | K940 | 12 | 638759 | 173767 | 9 | 0 | | | | | |
| LCPAC401 | CA | K1060 | 13 | 489919 | 134524 | 7 | 0.2 | 11 | 484752 | 114453 | 7 | 0.1 |
| LCPAC402 | none | K1000 | 52 | 512375 | 43066 | 7 | 0.2 | 29 | 351844 | 29851 | 6 | 0.2 |
| LCPAC403 | CA | K1060 | 14 | 361698 | 69088 | 6 | 0 | 6 | 420388 | 117884 | 6 | 0.1 |
| LCPAC404 | CA | K1000 | 34 | 333578 | 32968 | 9 | 0.2 | 10 | 436585 | 84762 | 8 | 0.1 |
| LCPAC405 | | K1060 | 62 | 715422 | 43487 | 8 | 0.1 | | | | | |
| LCPAC406 | CA | K1060 | 15 | 311202 | 70093 | 7 | 0 | 10 | 384297 | 75955 | 8 | 0 |
| LCDPAC01 | CA | K1060 | 39 | 279902 | 22099 | 6 | 0 | 21 | 282320 | 31931 | 8 | 0 |
| LCDPAC02 | CA | K1060 | 35 | 333796 | 20150 | 5 | 0 | 9 | 367310 | 90916 | 6 | 0 |
