# Supplementary material for: Virus Genomes from Deep Sea Sediments Expand the Ocean Megavirome and Support Independent Origins of Viral Gigantism
Source: mBio. 2019 Mar 5;10(2):e02497-18. doi: 10.1128/mBio.02497-18 (PMC6401483; doi:10.1128/mBio.02497-18)
Supplement: TEXT S2 [file mBio.02497-18-s0002.pdf]

This file contains trees for conserved proteins used for the concatenated tree shown on Figure 2 (namely, DNA polymerase, major capsid protein, and A18-like helicase), and trees for translation proteins found in Loki Castle virus (LCV) genomes.

LCV translation proteins are marked with the star whenever at least one bona-fide NCLDV protein was found on the same contig as the translation protein (see the last two slides for the details).

The multiple sequence alignments and the trees in Newick format could be downloaded from here:

ftp://ftp.ncbi.nih.gov/pub/yutinn/Loki_Castle_NCLDV_2018/MCP_DNAp_A18hel_aln_trees/

ftp://ftp.ncbi.nih.gov/pub/yutinn/Loki_Castle_NCLDV_2018/trans_aln_trees/

DNAp

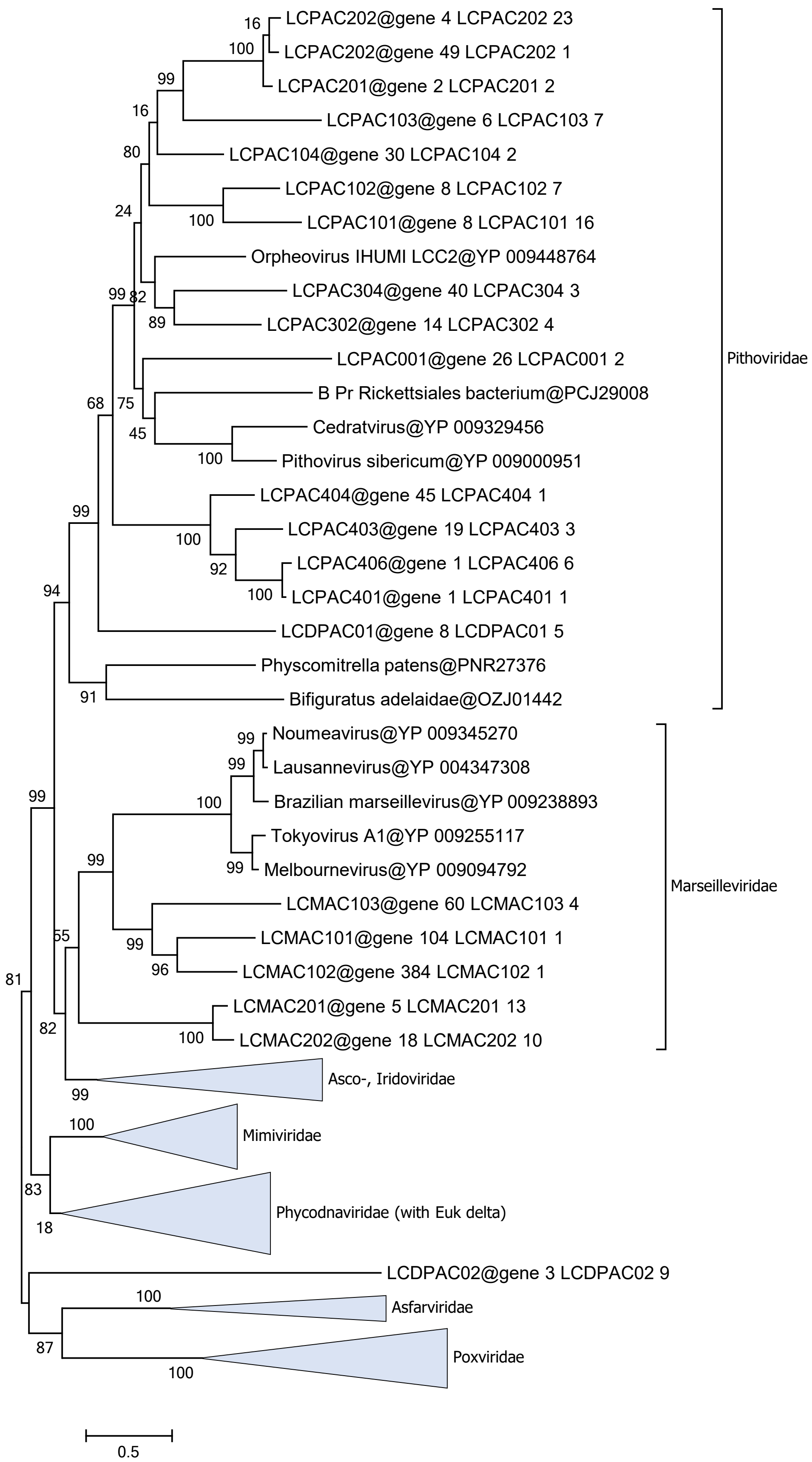

LCPAC202@gene 4 LCPAC202 23
LCPAC202@gene 49 LCPAC202 1
LCPAC201@gene 2 LCPAC201 2
LCPAC103@gene 6 LCPAC103 7
LCPAC104@gene 30 LCPAC104 2
LCPAC102@gene 8 LCPAC102 7
LCPAC101@gene 8 LCPAC101 16
Orpheovirus IHUMI LCC2@YP 009448764
LCPAC304@gene 40 LCPAC304 3
LCPAC302@gene 14 LCPAC302 4
LCPAC001@gene 26 LCPAC001 2
B Pr Rickettsiales bacterium@PCJ29008
Cedratvirus@YP 009329456
Pithovirus sibericum@YP 009000951
LCPAC404@gene 45 LCPAC404 1
LCPAC403@gene 19 LCPAC403 3
LCPAC406@gene 1 LCPAC406 6
LCPAC401@gene 1 LCPAC401 1
LCDPAC01@gene 8 LCDPAC01 5
Physcomitrella patens@PNR27376
Bifiguratus adelaidae@OZJ01442
Pithoviridae
Noumeavirus@YP 009345270
Lausannevirus@YP 004347308
Brazilian marseillevirus@YP 009238893
Tokyovirus A1@YP 009255117
Melbournevirus@YP 009094792
LCMAC103@gene 60 LCMAC103 4
LCMAC101@gene 104 LCMAC101 1
LCMAC102@gene 384 LCMAC102 1
LCMAC201@gene 5 LCMAC201 13
LCMAC202@gene 18 LCMAC202 10
Marseilleviridae
Asco-, Iridoviridae
Mimiviridae
Phycodnaviridae (with Euk delta)
LCDPAC02@gene 3 LCDPAC02 9
Asfarviridae
Poxviridae
16
100
99
16
80
24
100
99
82
89
68
75
45
100
99
100
92
100
94
91
99
99
99
100
99
99
55
99
96
81
82
100
99
100
83
18
100
87
100
0.5

MCP

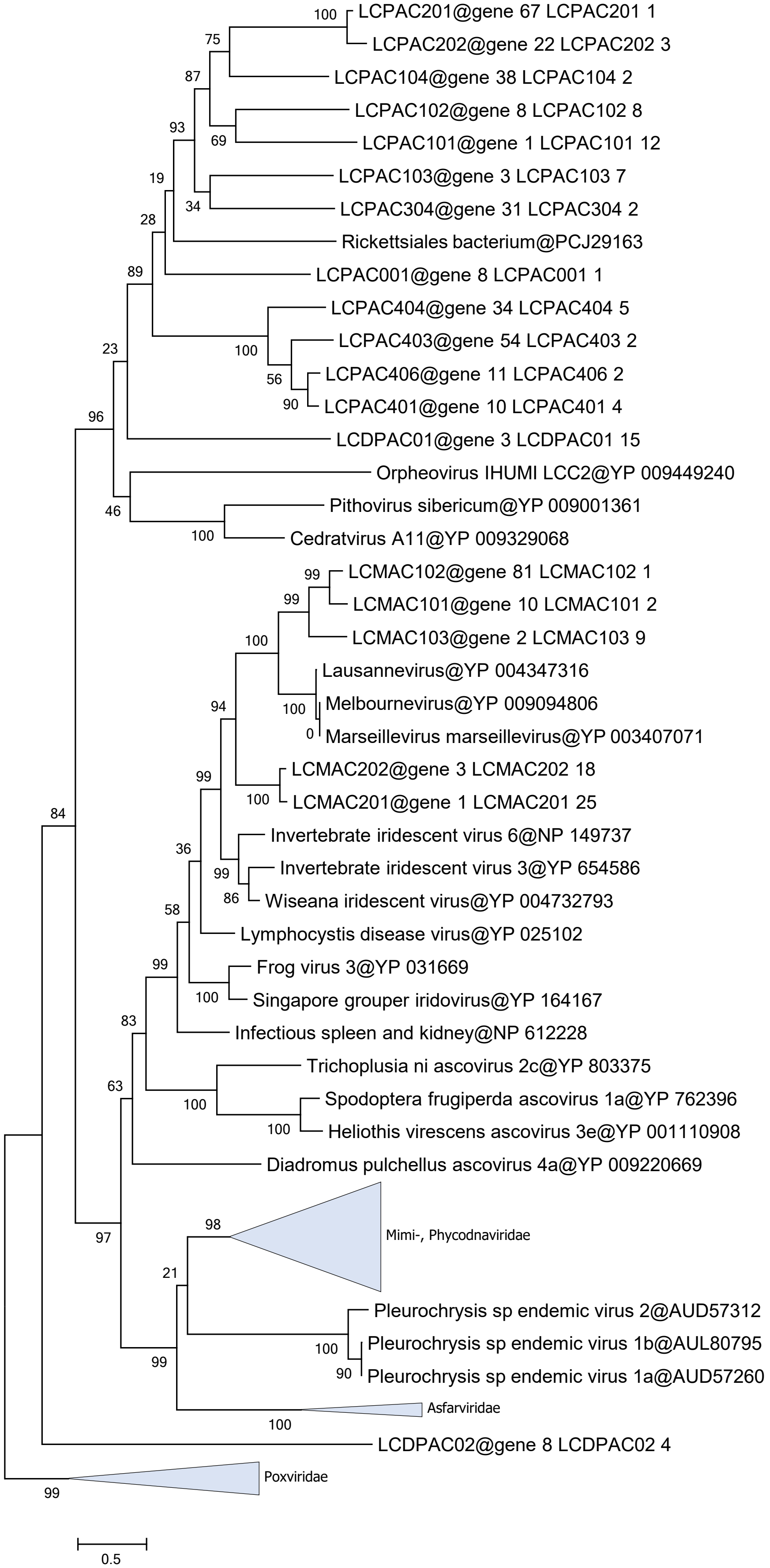

LCPAC201@gene 67 LCPAC201 1
LCPAC202@gene 22 LCPAC202 3
LCPAC104@gene 38 LCPAC104 2
LCPAC102@gene 8 LCPAC102 8
LCPAC101@gene 1 LCPAC101 12
LCPAC103@gene 3 LCPAC103 7
LCPAC304@gene 31 LCPAC304 2
Rickettsiales bacterium@PCJ29163
LCPAC001@gene 8 LCPAC001 1
LCPAC404@gene 34 LCPAC404 5
LCPAC403@gene 54 LCPAC403 2
LCPAC406@gene 11 LCPAC406 2
LCPAC401@gene 10 LCPAC401 4
LCDPAC01@gene 3 LCDPAC01 15
Orpheovirus IHUMI LCC2@YP 009449240
Pithovirus sibericum@YP 009001361
Cedratvirus A11@YP 009329068
LCMAC102@gene 81 LCMAC102 1
LCMAC101@gene 10 LCMAC101 2
LCMAC103@gene 2 LCMAC103 9
Lausannevirus@YP 004347316
Melbournevirus@YP 009094806
Marseillevirus marseillevirus@YP 003407071
LCMAC202@gene 3 LCMAC202 18
LCMAC201@gene 1 LCMAC201 25
Invertebrate iridescent virus 6@NP 149737
Invertebrate iridescent virus 3@YP 654586
Wiseana iridescent virus@YP 004732793
Lymphocystis disease virus@YP 025102
Frog virus 3@YP 031669
Singapore grouper iridovirus@YP 164167
Infectious spleen and kidney@NP 612228
Trichoplusia ni ascovirus 2c@YP 803375
Spodoptera frugiperda ascovirus 1a@YP 762396
Heliothis virescens ascovirus 3e@YP 001110908
Diadromus pulchellus ascovirus 4a@YP 009220669
Mimi-, Phycodnaviridae
Pleurochrysis sp endemic virus 2@AUD57312
Pleurochrysis sp endemic virus 1b@AUL80795
Pleurochrysis sp endemic virus 1a@AUD57260
Asfarviridae
LCDPAC02@gene 8 LCDPAC02 4
Poxviridae
0.5

A18-like helicase

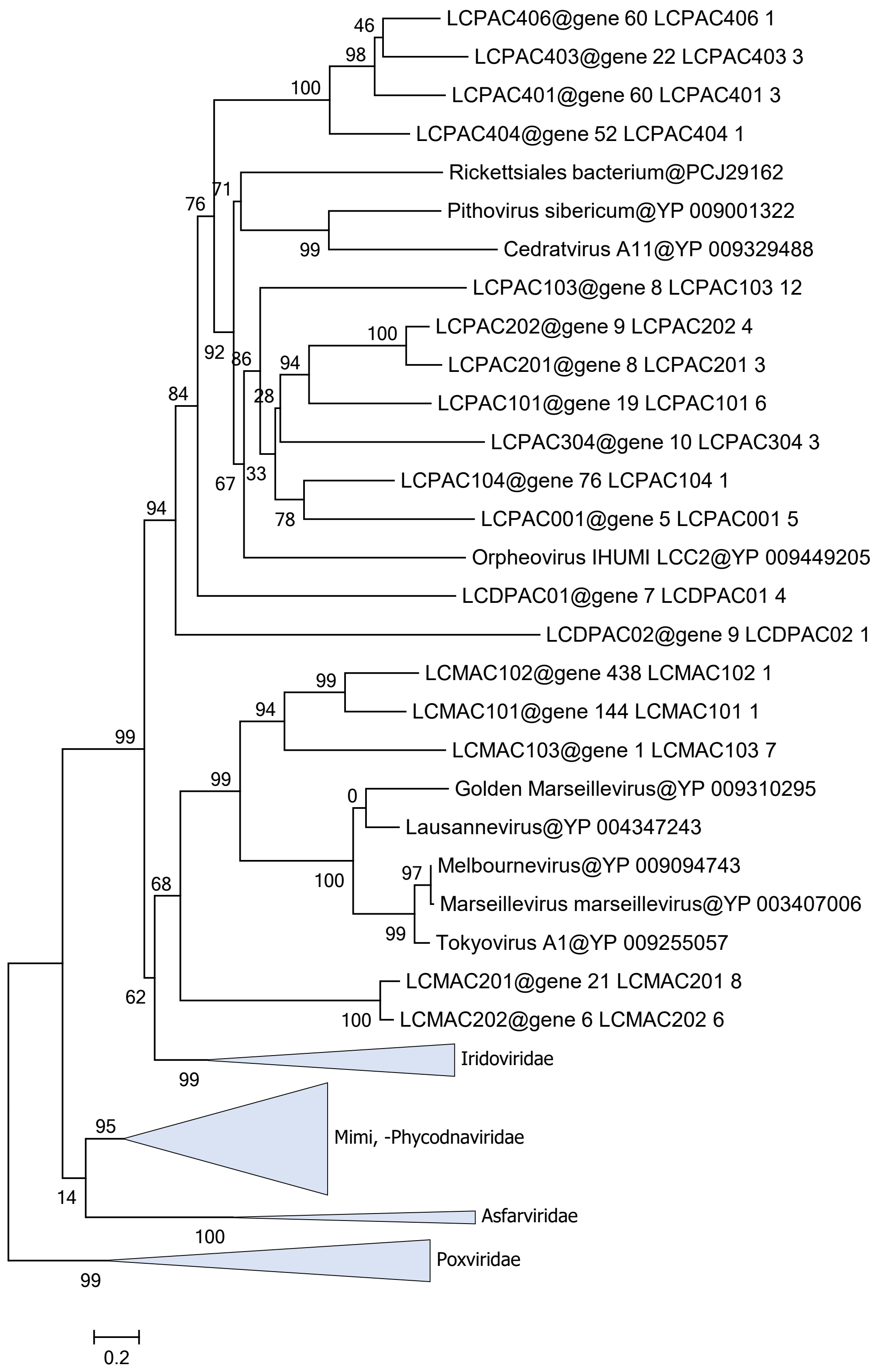

AlaS Alanyl-tRNA synthetase

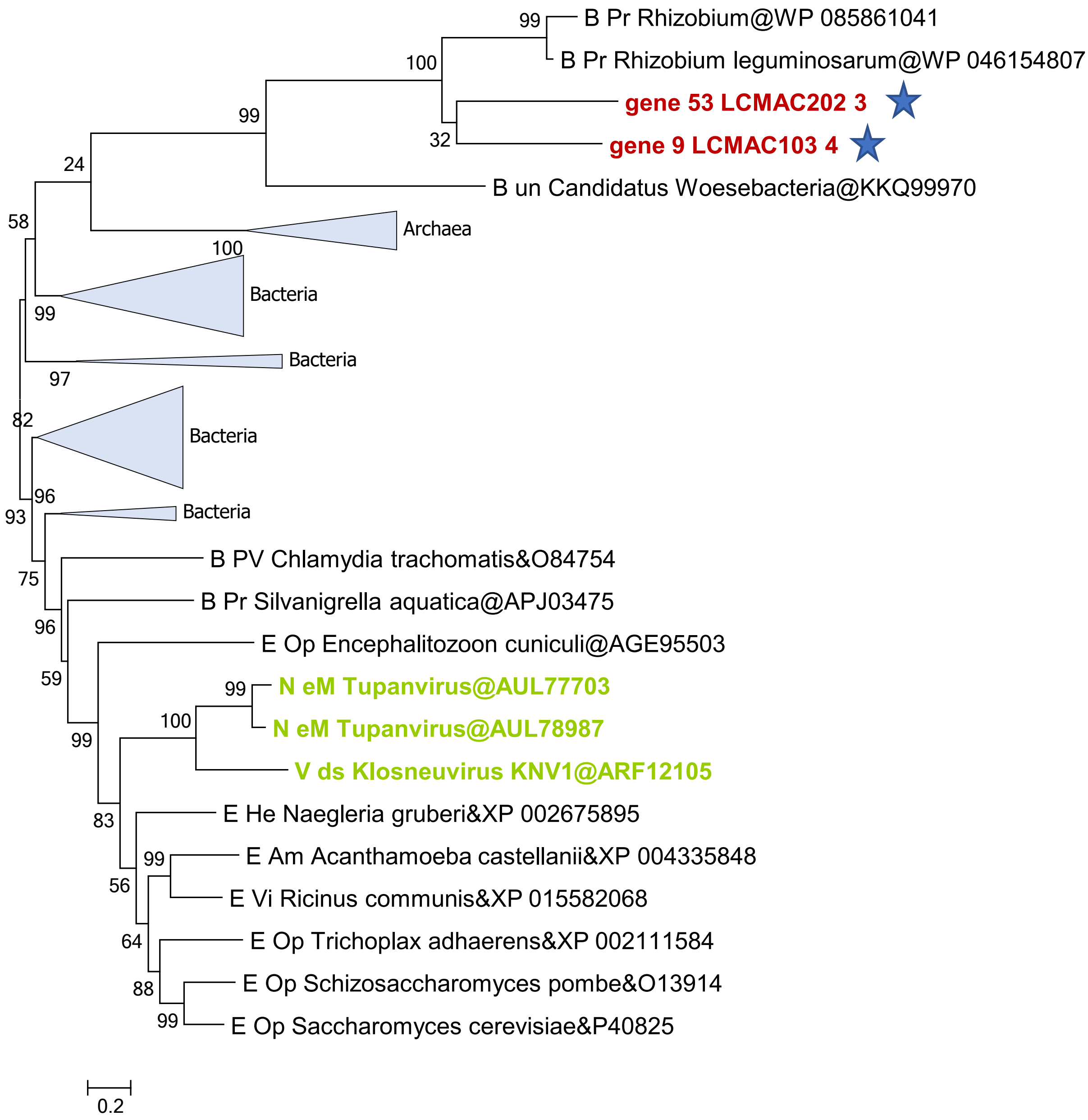

AsnS Aspartyl/asparaginyl-tRNA synthetase

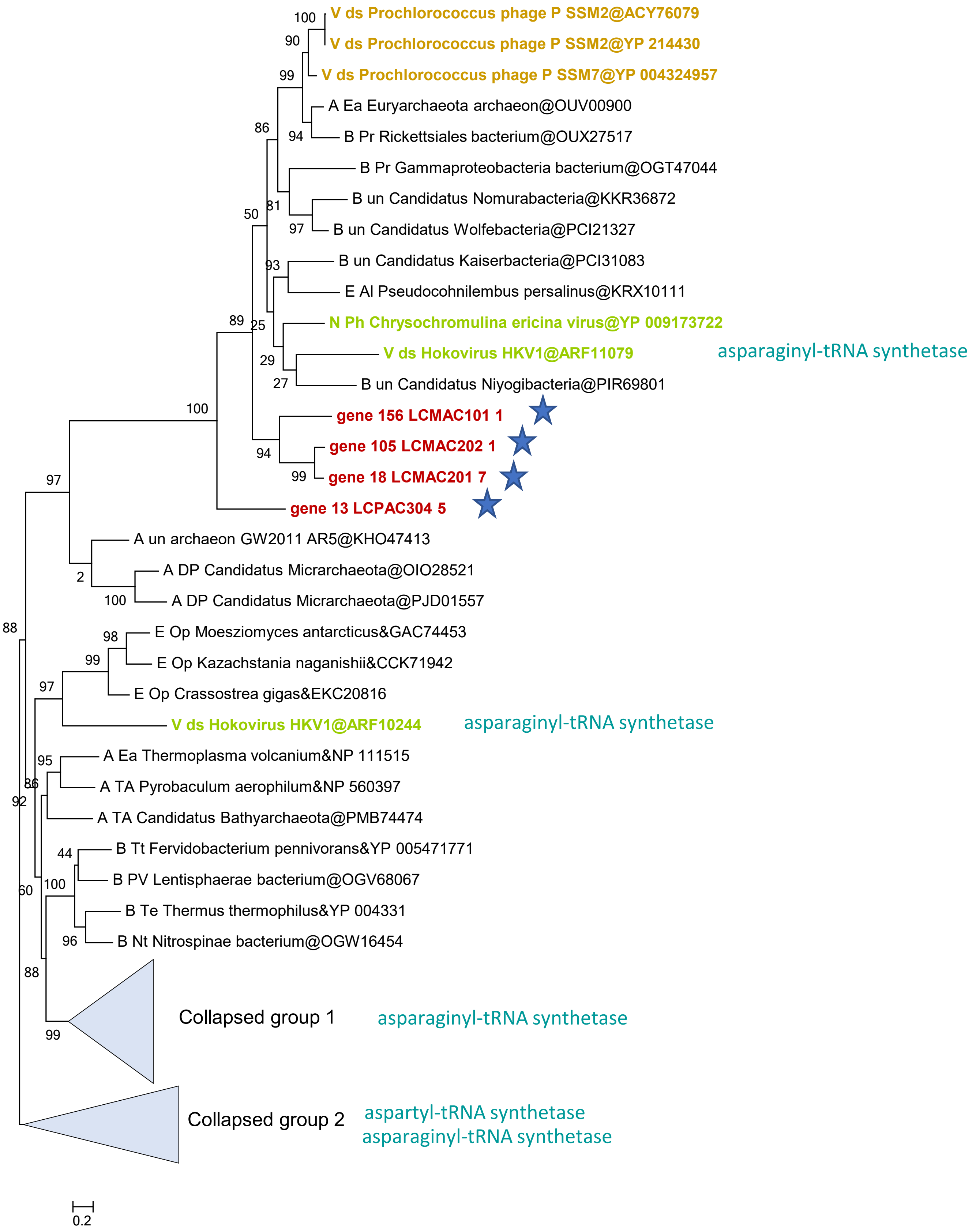

AsnS Aspartyl/asparaginyl-tRNA synthetase

Collapsed group 1

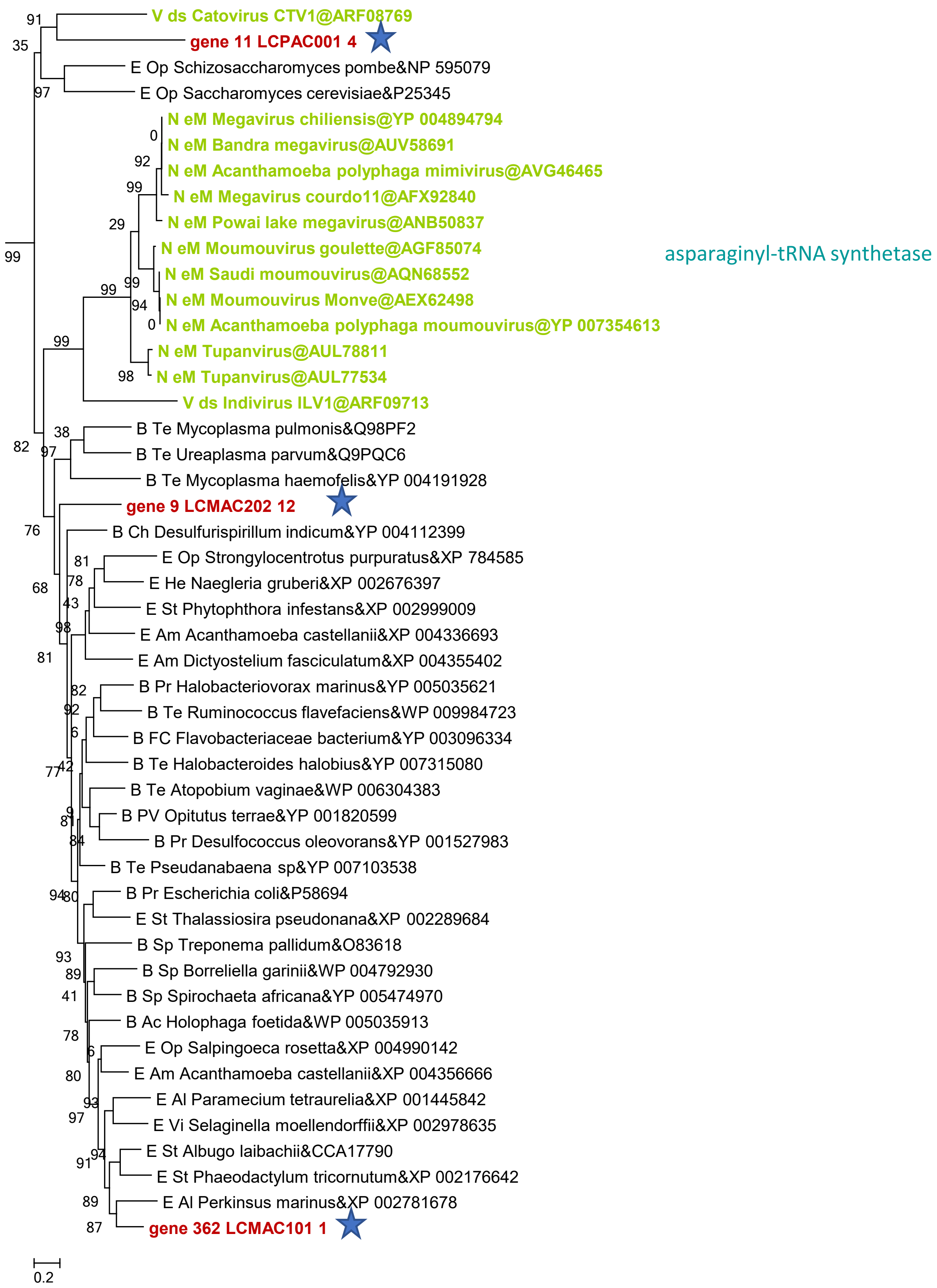

AsnS Aspartyl/asparaginyl-tRNA synthetase

Collapsed group 2

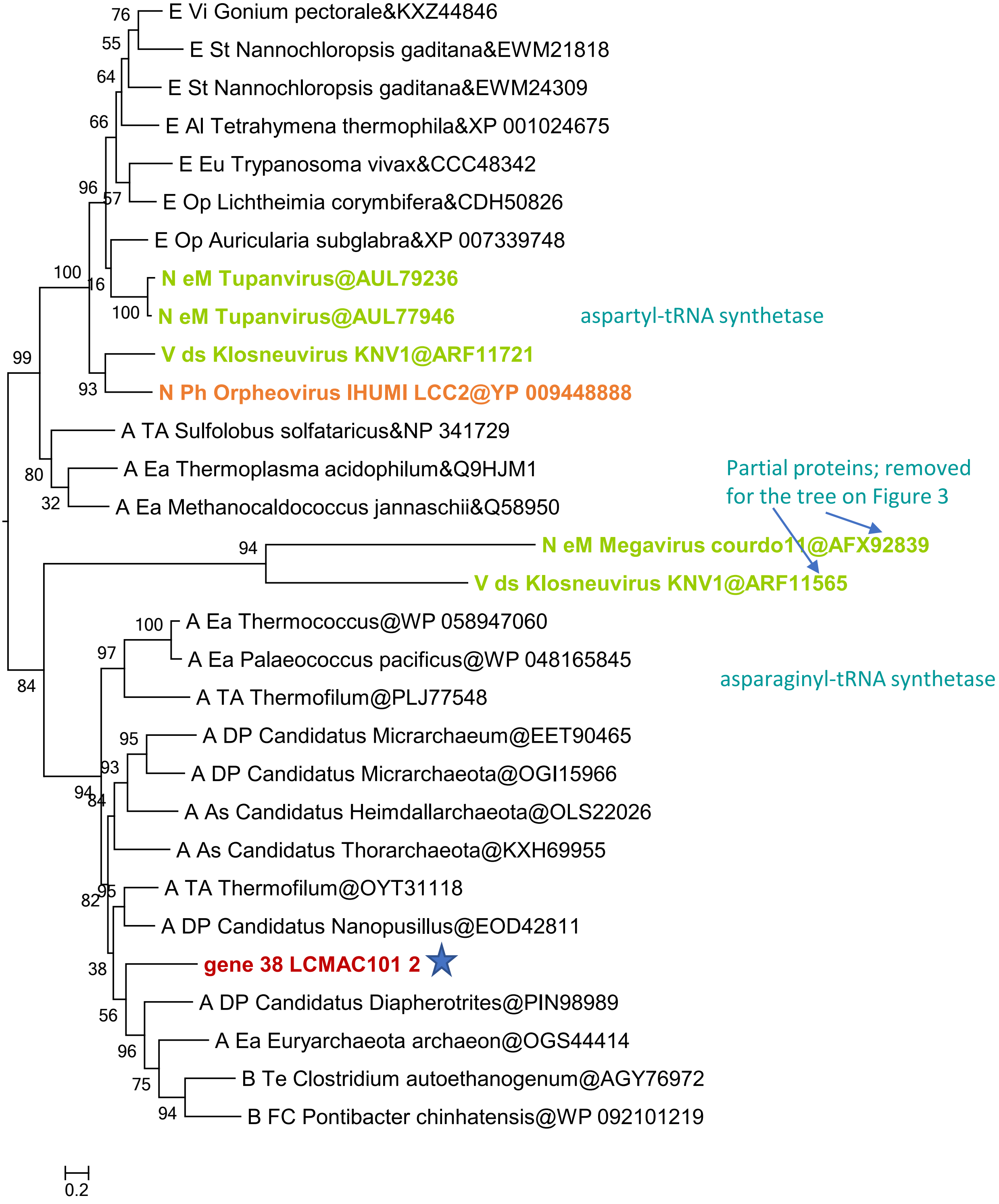

GlnS Glutamyl- or glutaminyl-tRNA synthetase

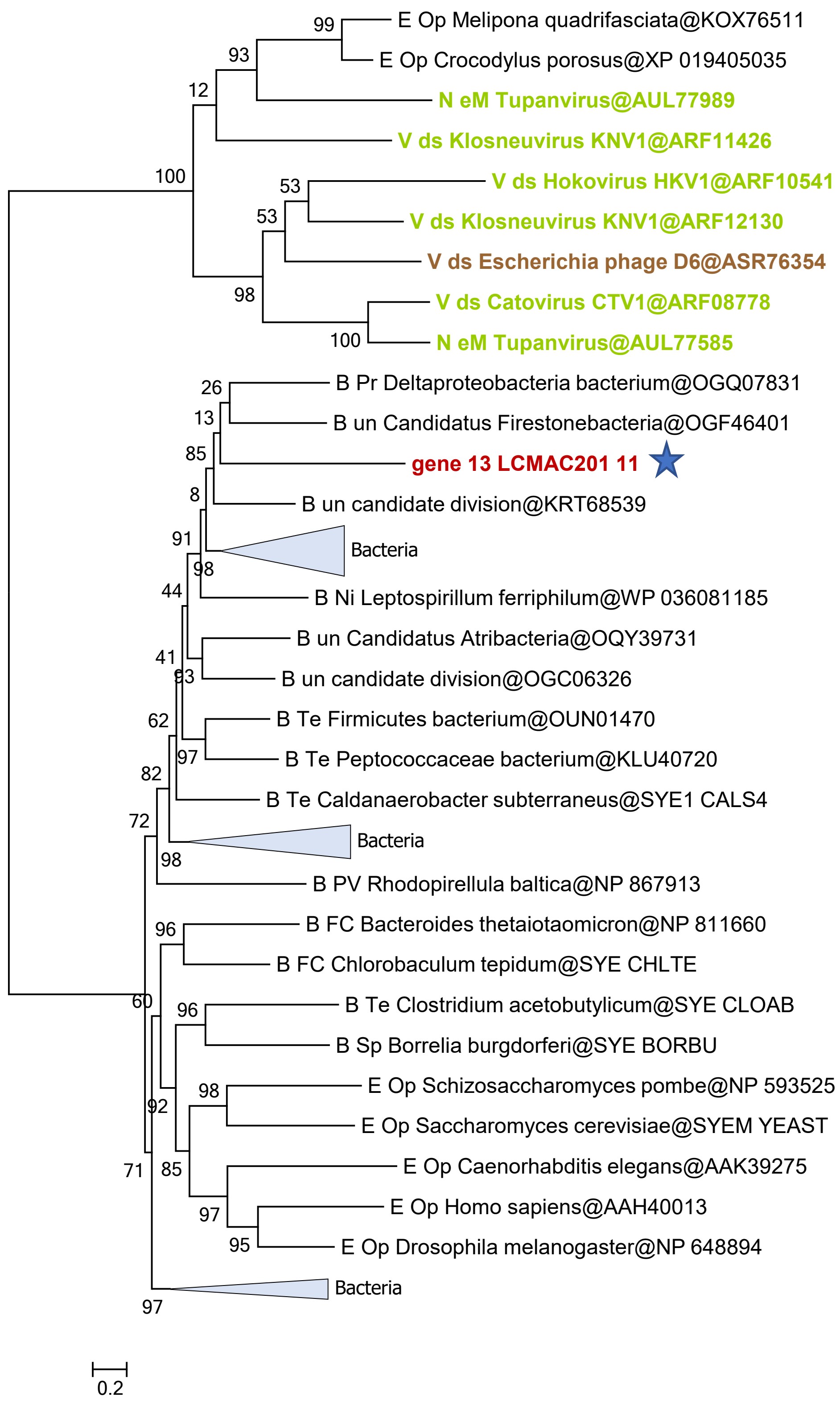

GRS1 Glycyl-tRNA synthetase (class II)

Also Orpheovirus YP_009448415

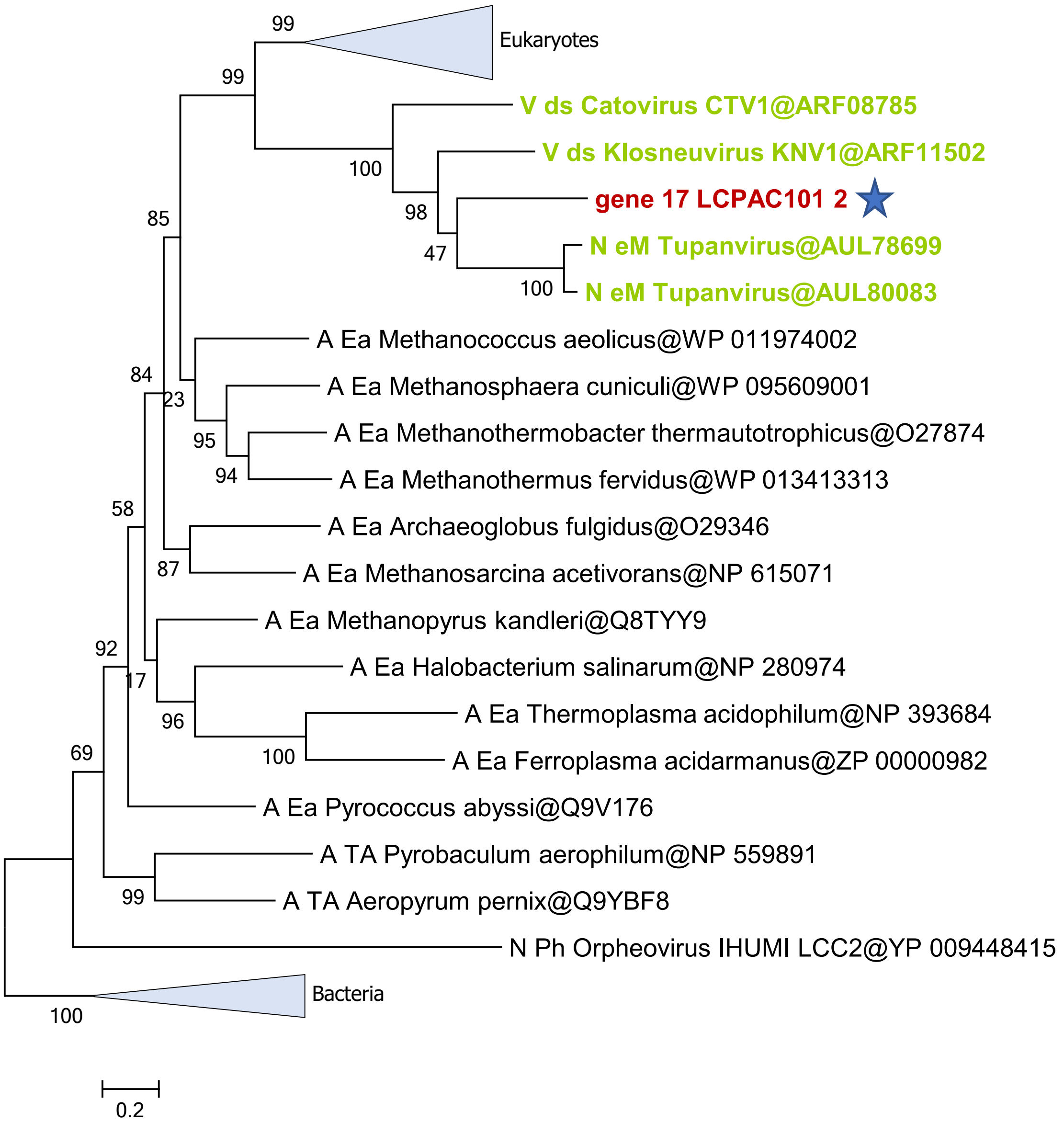

HisS Histidyl-tRNA synthetase

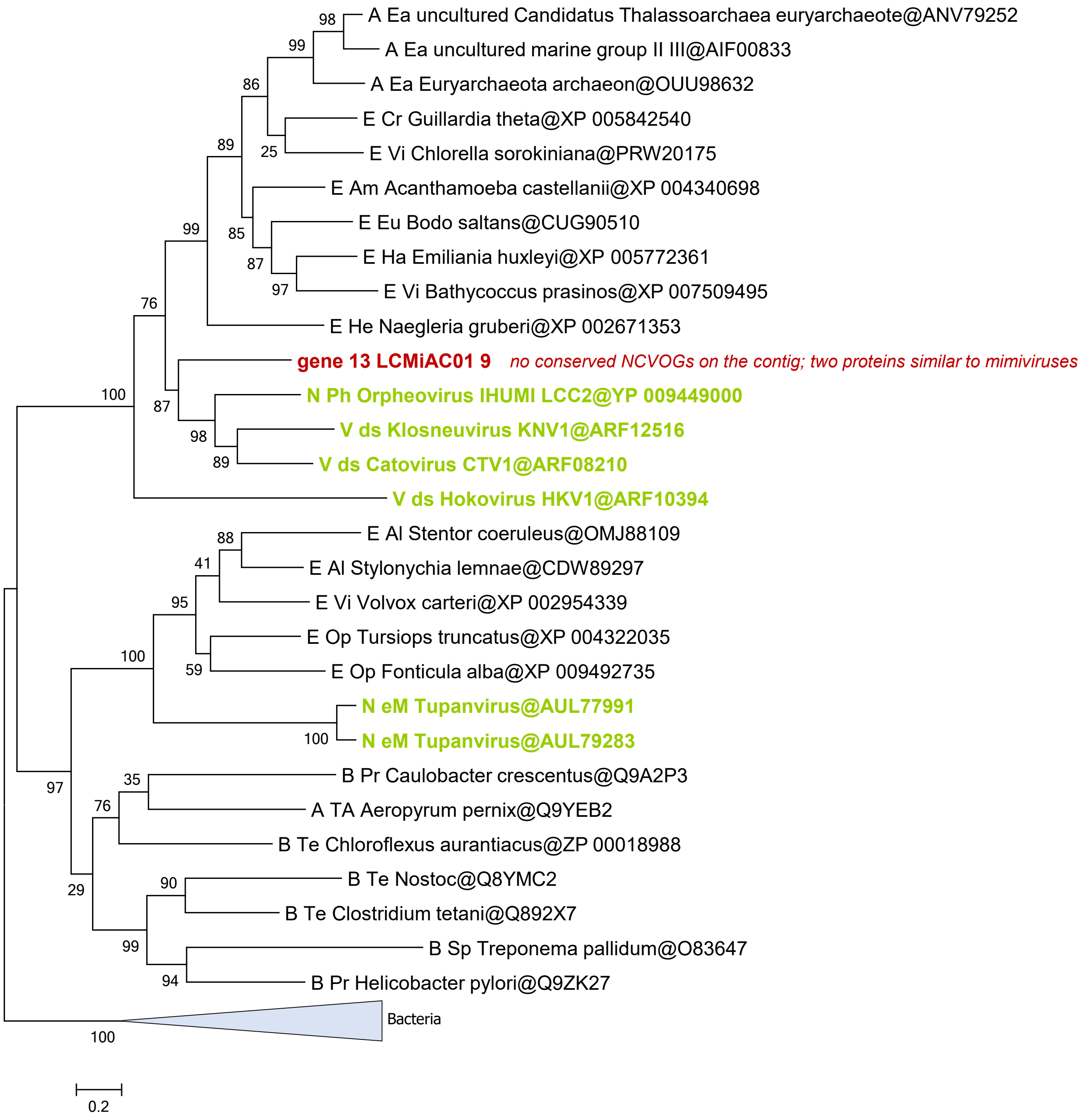

IleS Isoleucyl-tRNA synthetase

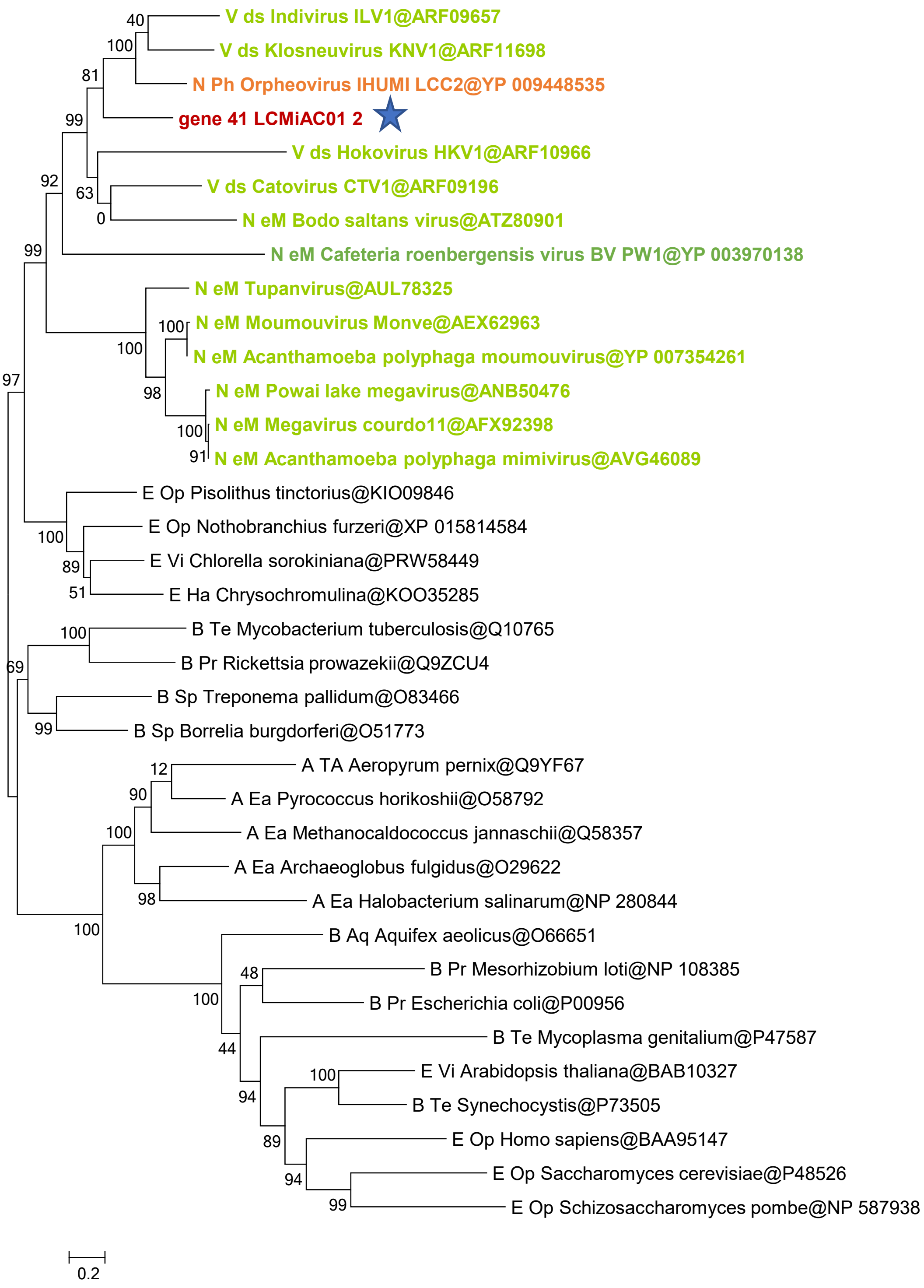

MetS Methionyl-tRNA synthetase

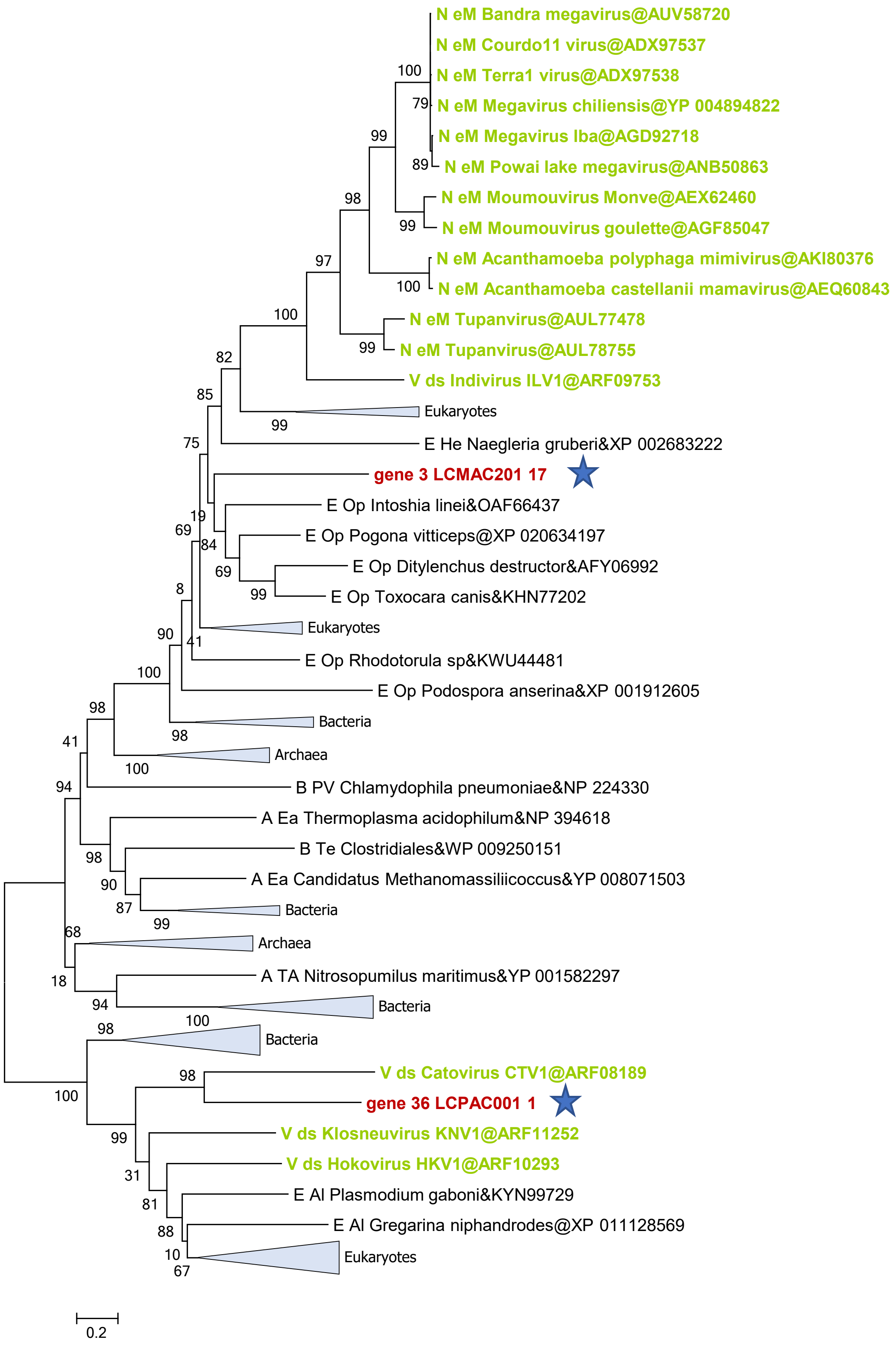

ProS Prolyl-tRNA synthetase

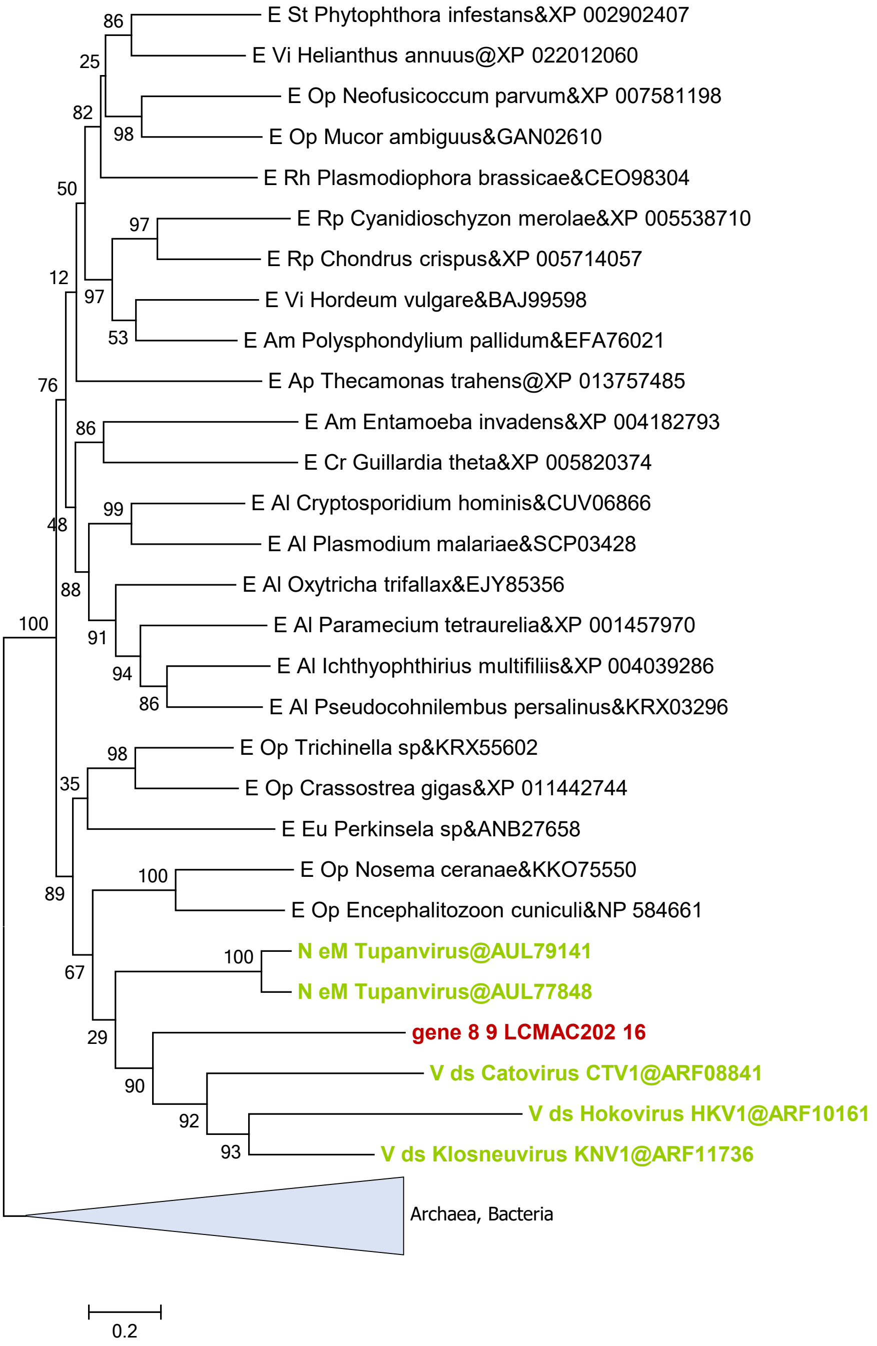

LC_NCLDV_2018/new_trans_trees/ProS.tre

ThrS Threonyl-tRNA synthetase

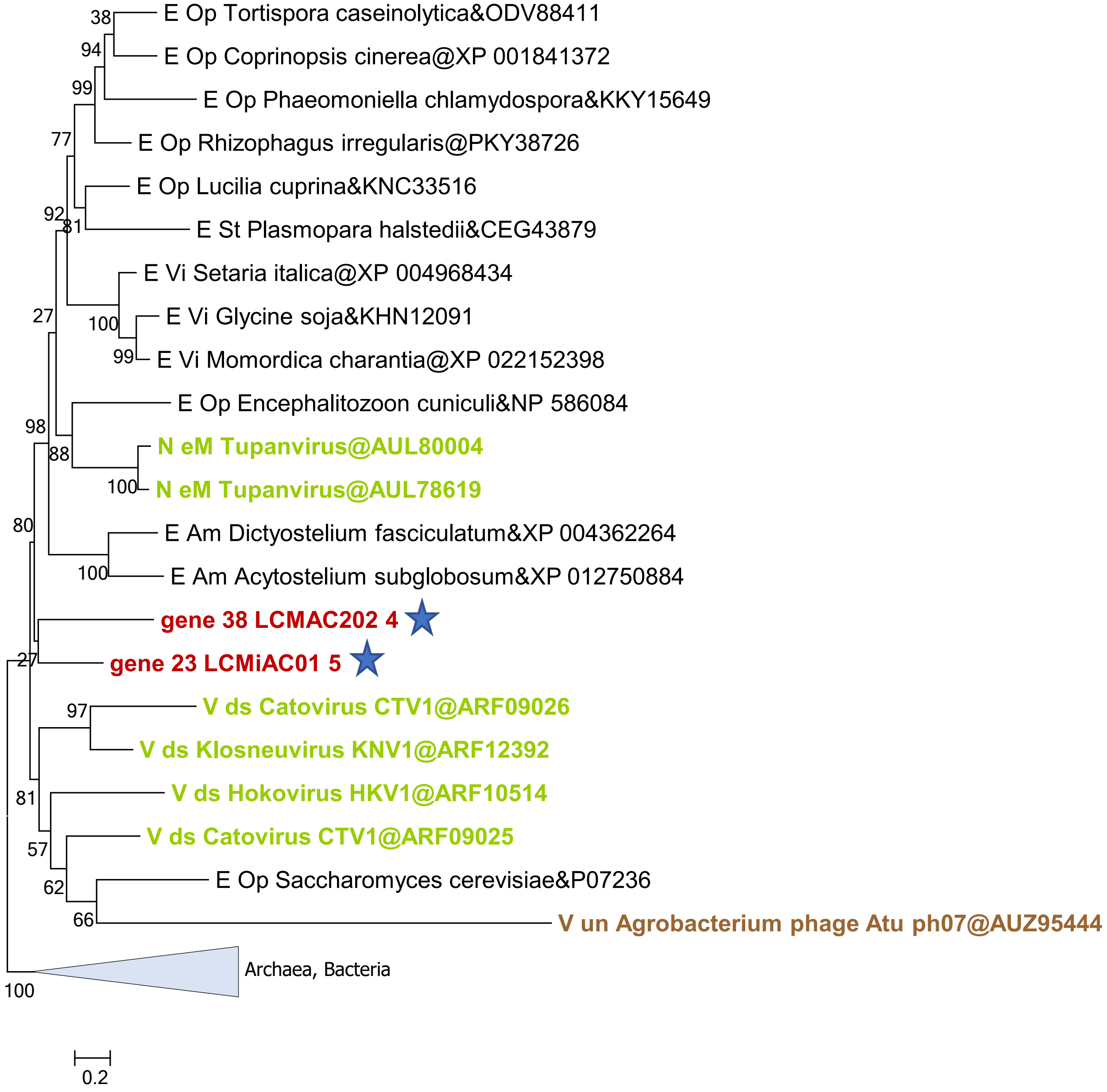

TrpS Tryptophanyl-tRNA synthetase

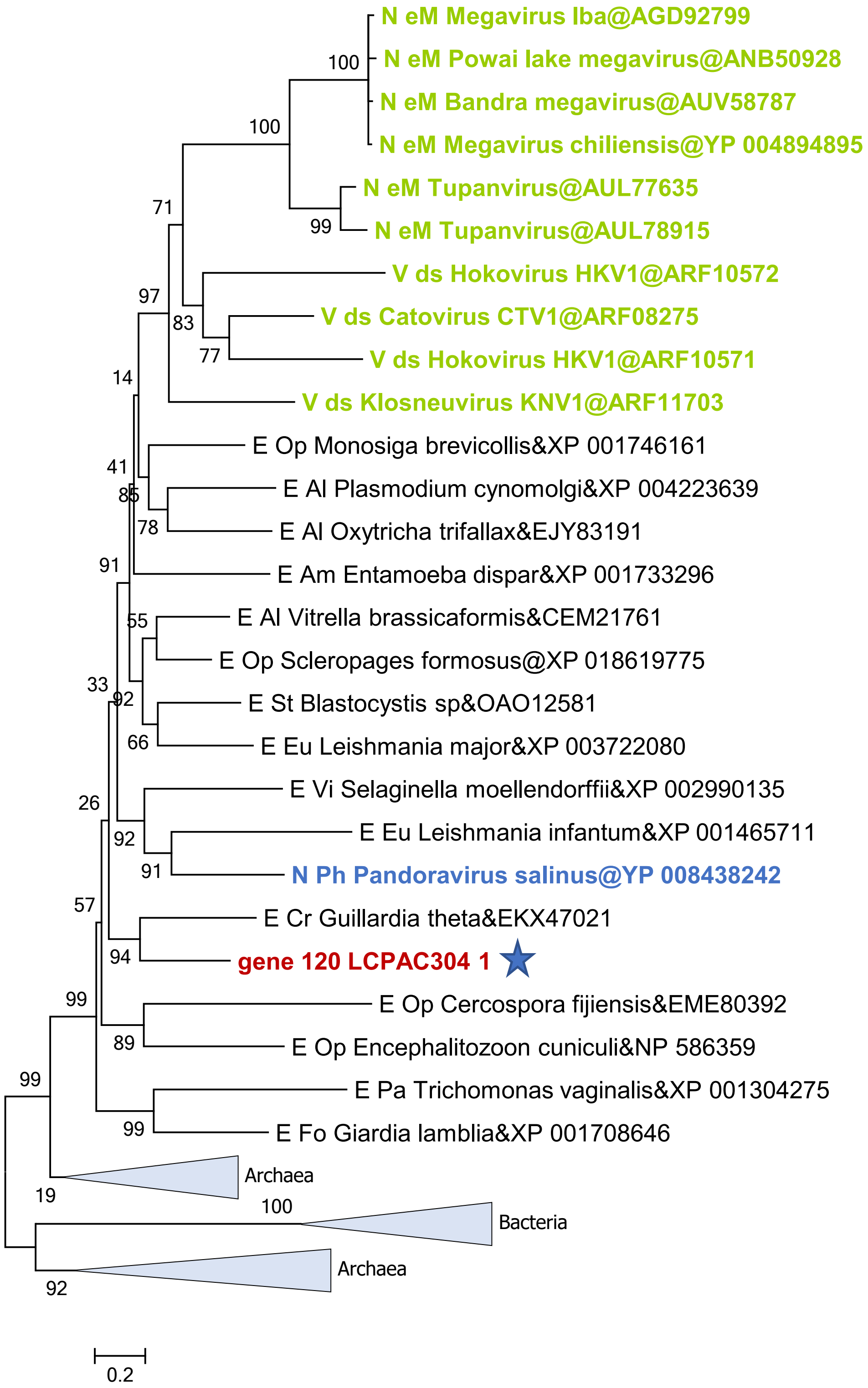

TyrS Tyrosyl-tRNA synthetase

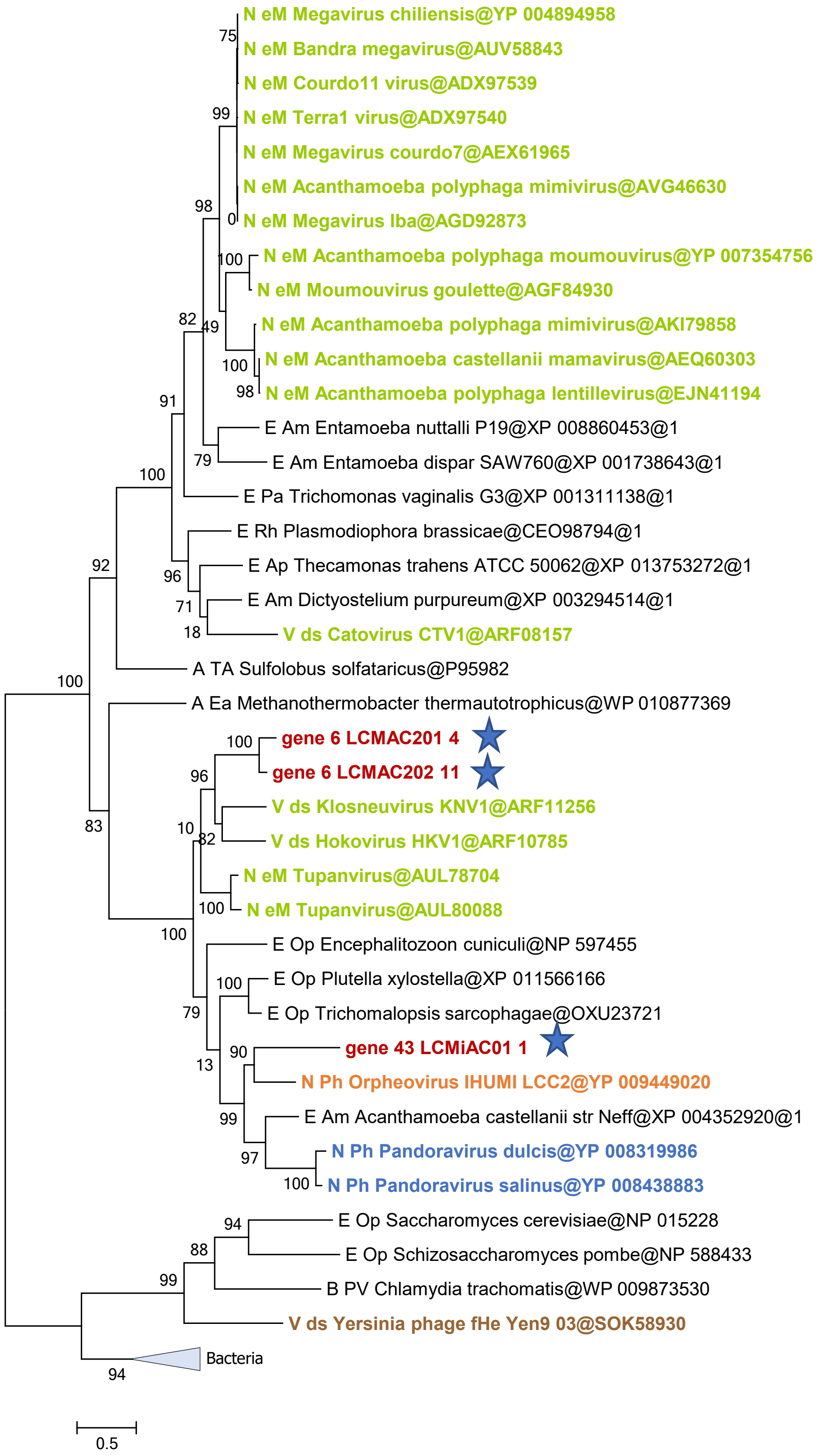

Pth2 Peptidyl-tRNA hydrolase

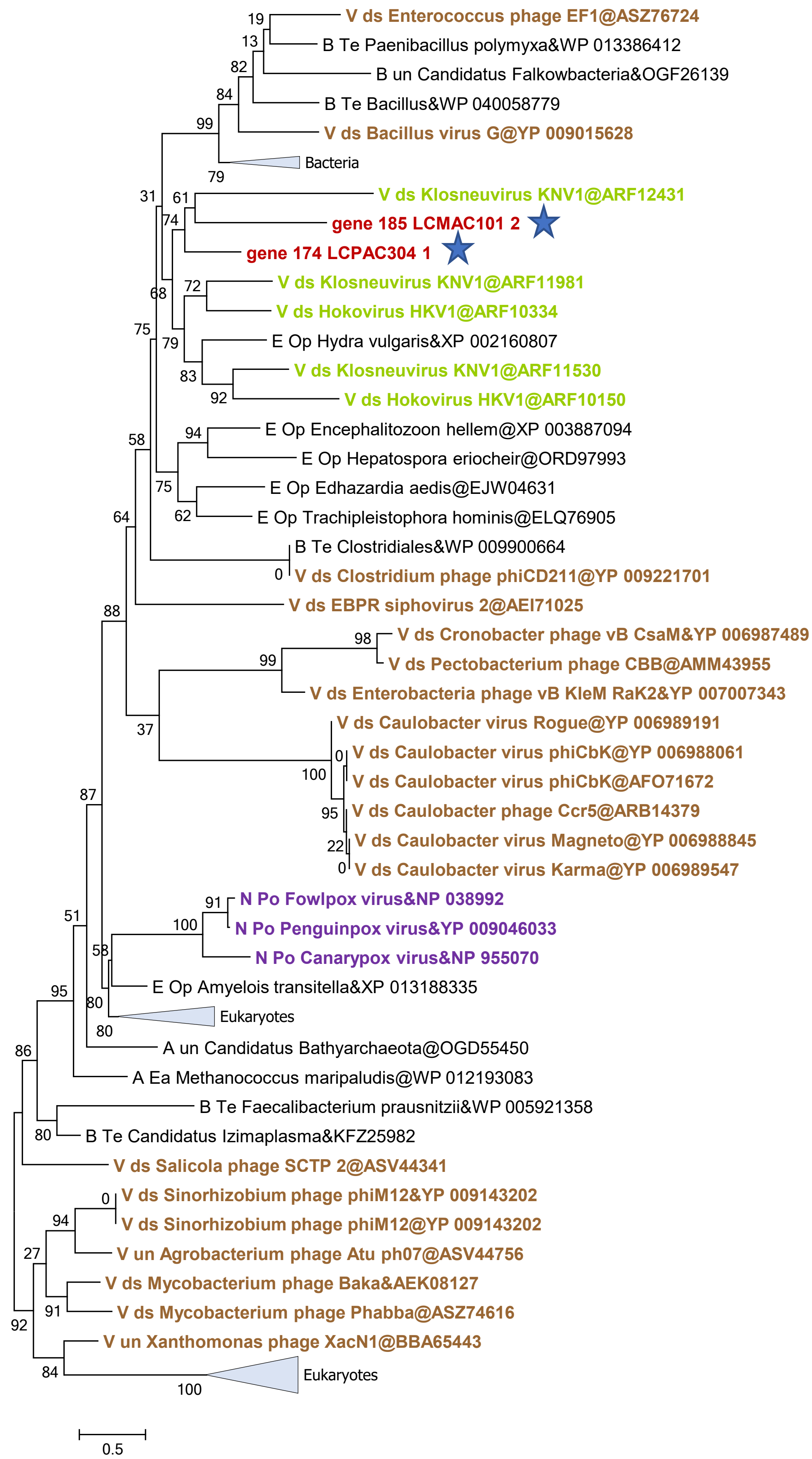

eIF1 Translation initiation factor 1 (eIF-1/SUI1)

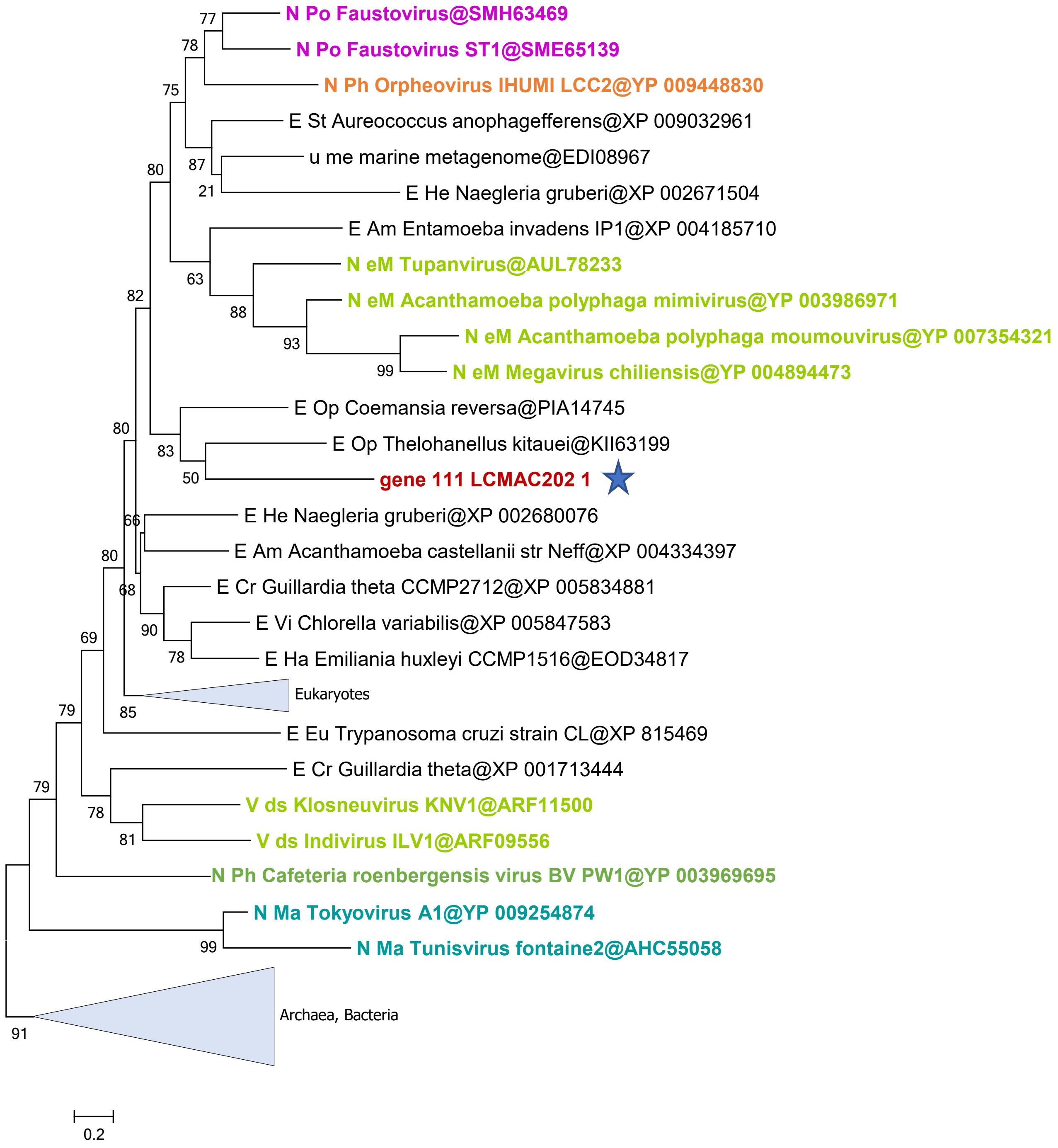

eIF2a Translation initiation factor 2, alpha subunit (eIF-2alpha)

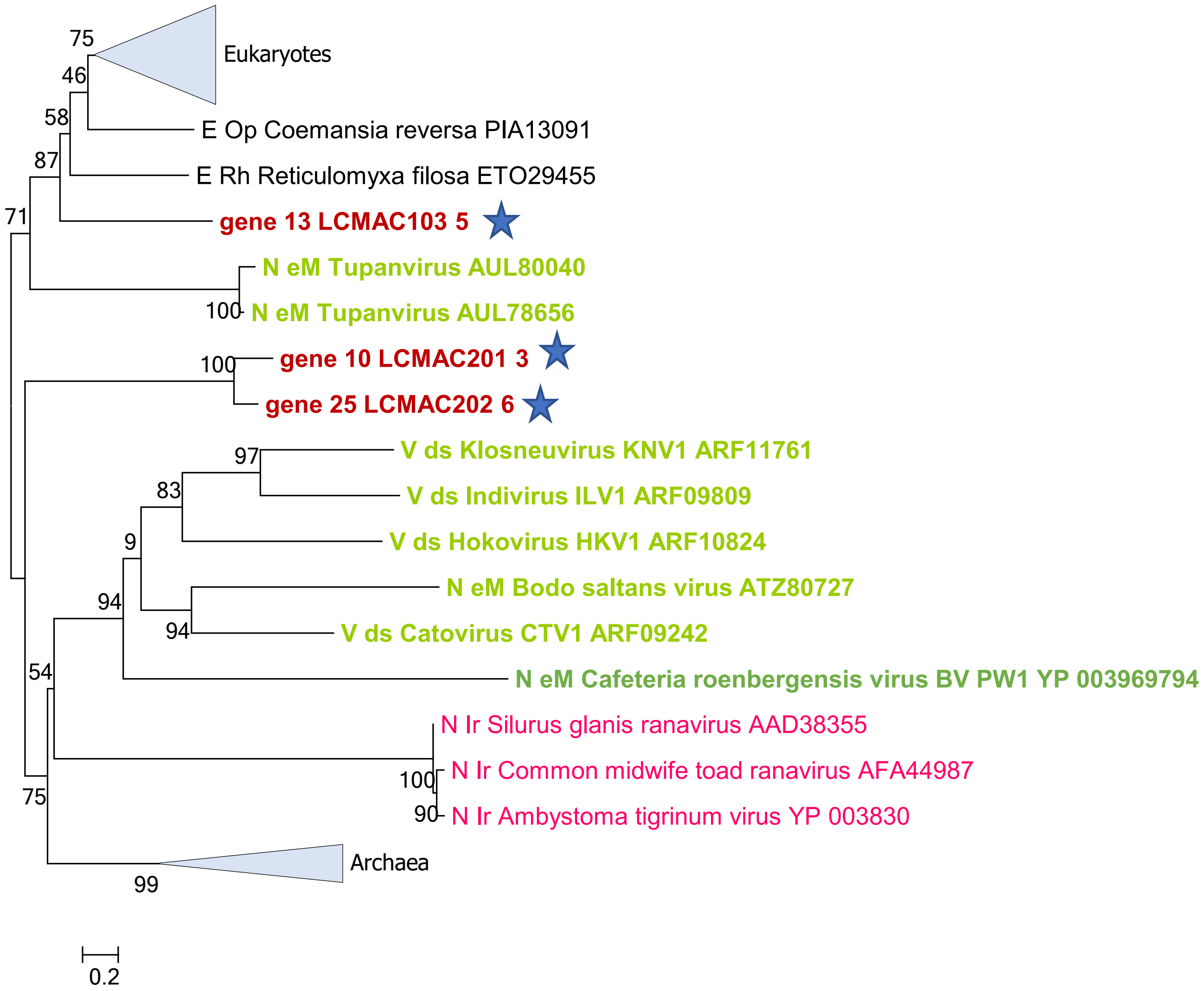

eIF2b Translation initiation factor 2, beta subunit (eIF-2beta)/eIF-5 N-terminal domain

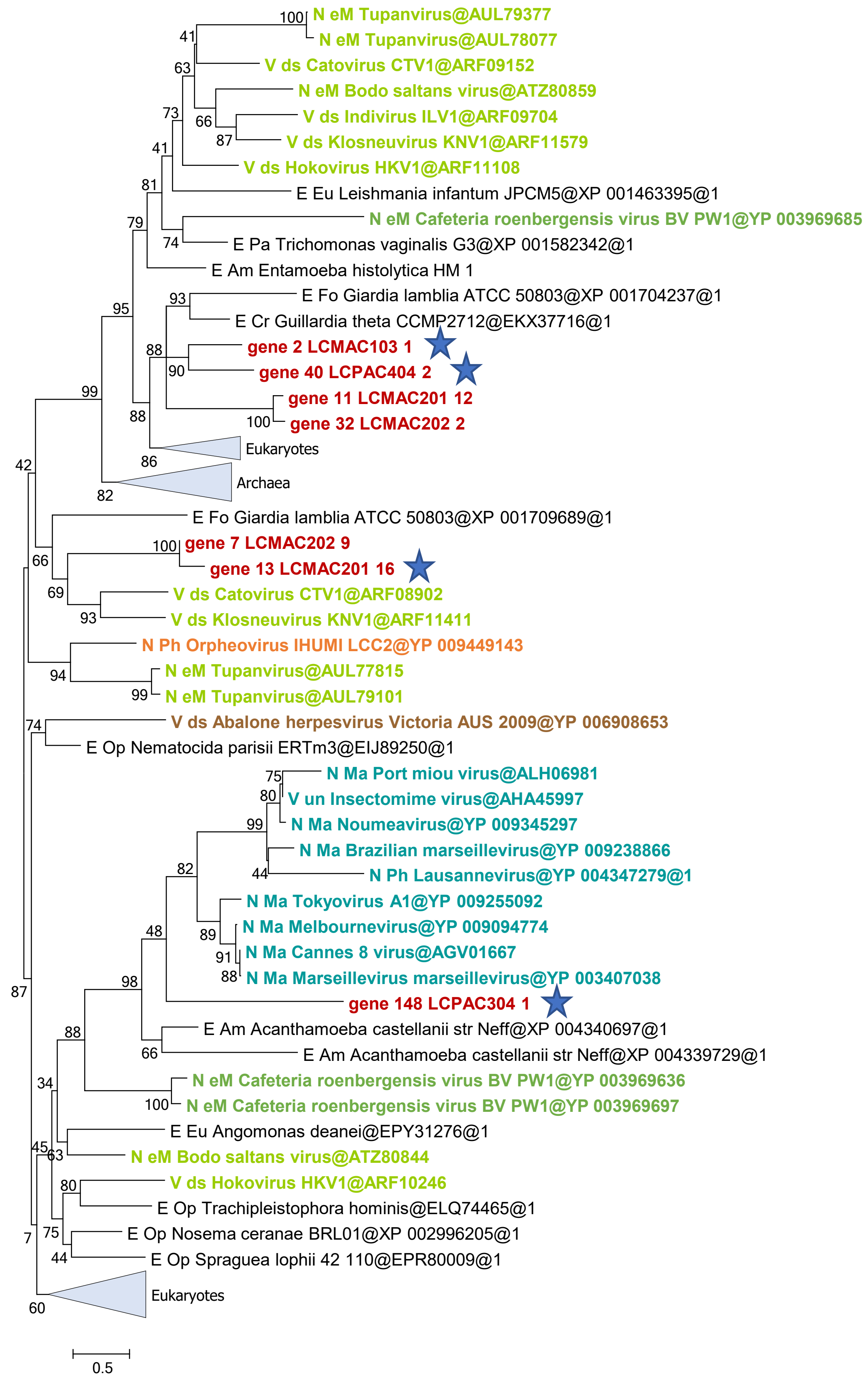

eIF2g Translation initiation factor 2, gamma subunit (eIF-2gamma; GTPase)

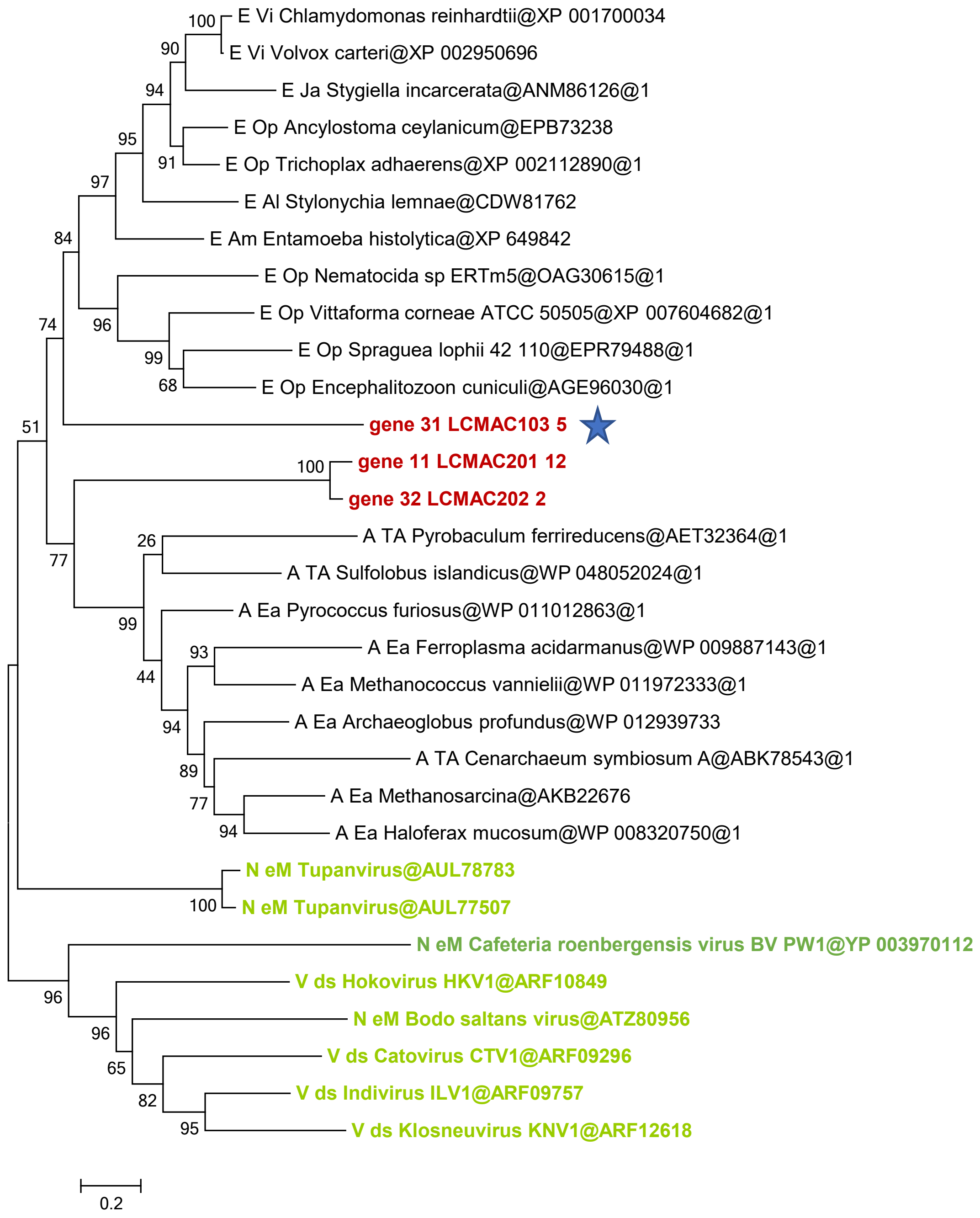

eIF4e Translation initiation factor 4E (eIF-4E)

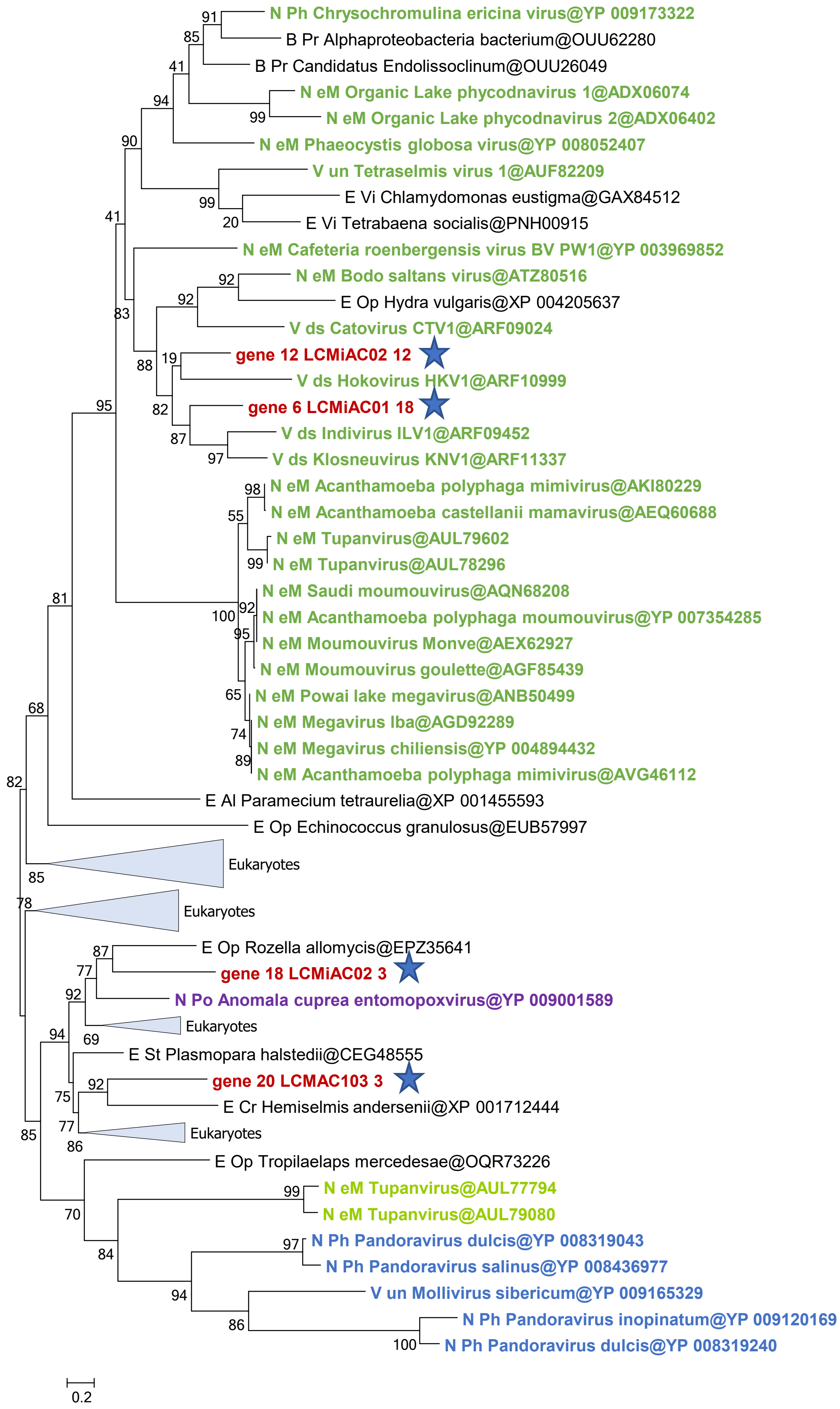

eIF5B / IF2 Translation initiation factor IF-2

Initiation Factor 2 (IF2) eukaryotic Initiation Factor 5B (eIF5B) family; IF2/eIF5B

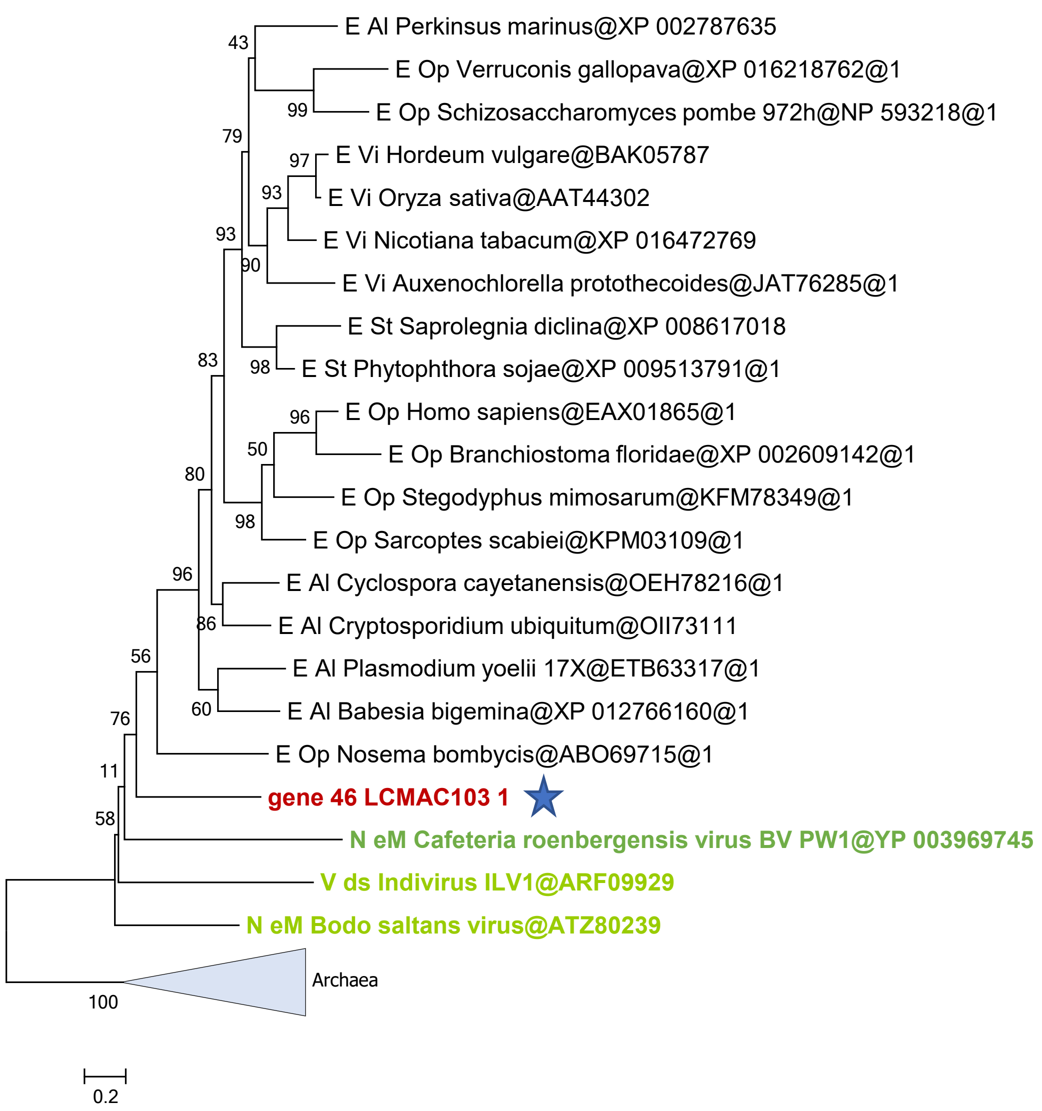

eRF1 Peptide chain release factor 1 (eRF1)

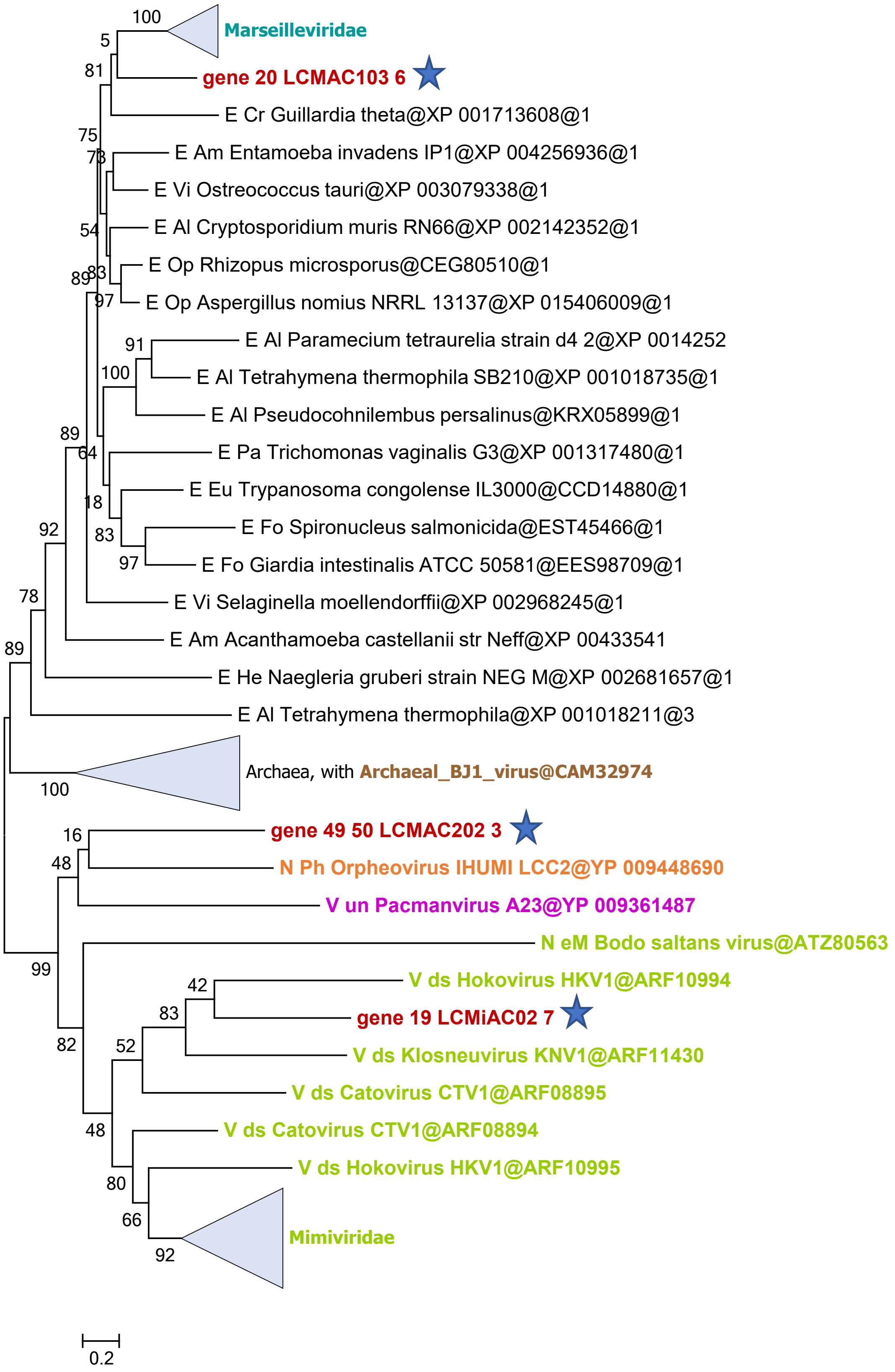

RLI1 Translation initiation factor RLI1

in Archaea: ribosome biogenesis/translation initiation ATPase RLI

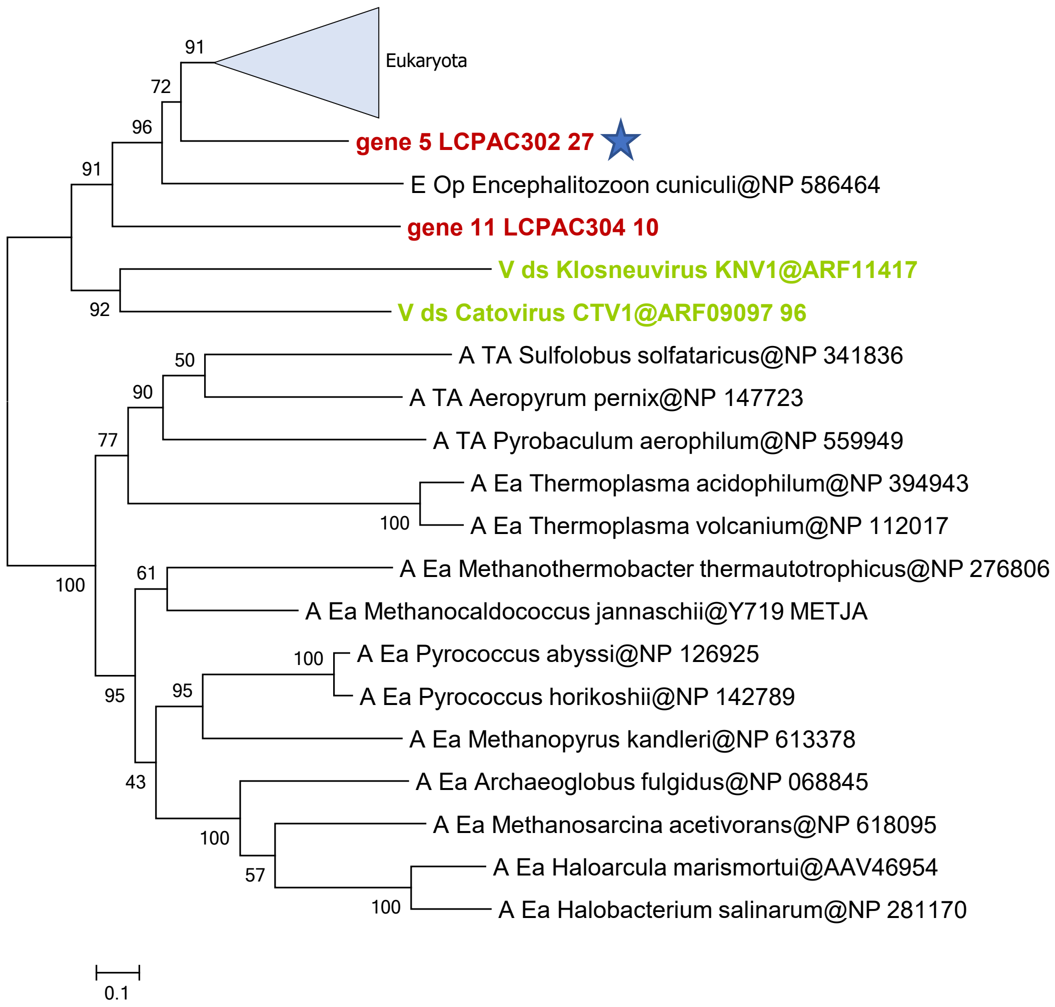

eIF1a Translation initiation factor 1A / IF-1 (eIF-1A for eukaryotes)

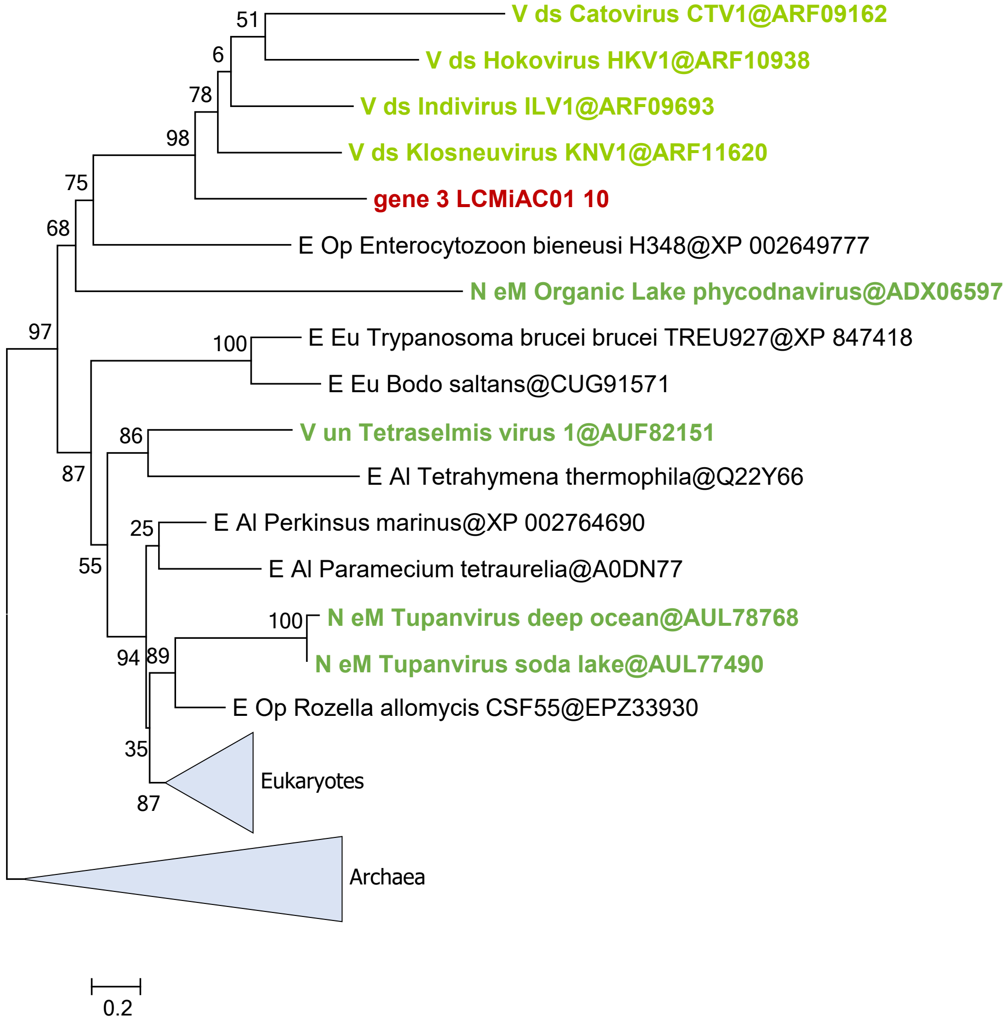

LCV contigs containing a translation protein and a 'bona fide' NCLDV protein, as defined by PIM clusters

LCMAC101_1	pim0002,pim0003,pim0004,pim0007,pim0008,pim0011,pim0014,pim0022, pim0027,pim0030,pim0031,pim0053,pim0054,pim0136,pim0142,pim0144
LCMAC101_2	pim0001,pim0005,pim0013
LCMAC103_1	pim0011,pim0027,pim0054
LCMAC103_3	pim0005,pim0142
LCMAC103_4	pim0002,pim0003,pim0030
LCPAC404_2	pim0004,pim0005,pim0011,pim0013
LCMAC201_11	pim0004,pim0014
LCMAC202_1	pim0004,pim0014,pim0022,pim0027,pim0144
LCMAC202_10	pim0002,pim0031
LCMAC202_18	pim0001,pim0013
LCMAC202_3	pim0004,pim0014,pim0054
LCMAC202_4	pim0007,pim0053,pim0136
LCMAC202_6	pim0003,pim0008,pim0011,pim0030
LCPAC001_1	pim0001,pim0016,pim0030,pim0136
LCPAC001_11	pim0004,pim0019
LCPAC101_2	pim0027,pim0030,pim0035
LCMAC103_5	pim0004
LCMAC201_16	pim0054
LCMAC201_3	pim0003
LCMAC201_4	pim0027
LCPAC101_20	pim0004
LCPAC101_21	pim0142
LCPAC304_1	pim0116
LCPAC304_11	pim0011
LCPAC304_5	pim0054
LCPAC202_1	pim0192
LCPAC201_6	pim0192
LCPAC101_1	pim0192
LCPAC302_27	pim0192
LCPAC104_3	pim0192
LCPAC103_11	pim0192
LCPAC304_5	pim0134
LCPAC201_3	pim0134
LCPAC202_4	pim0134
LCPAC403_2	pim0134
LCPAC001_4	pim0134
LCPAC103_11	pim0134
LCPAC101_2	pim0134
LCPAC401_3	pim0134
LCPAC406_1	pim0134
LCPAC001_4	pim0042
LCDPAC01_7	pim0042
LCPAC103_3	pim0042
LCMAC202_1	pim0042
LCPAC202_5	pim0042
LCPAC302_16	pim0042
LCPAC102_1	pim0042
LCPAC201_4	pim0042
LCMAC101_1	pim0042
CPAC101_5	pim0042
LCMAC102_1	pim0042
LCMAC201_17	pim0042
LCPAC104_2	pim0042
LCPAC304_2	pim0042
LCMAC202_10	pim0071
LCPAC202_1	pim0071
LCMAC101_1	pim0071
LCPAC201_1	pim0071
LCIVAC01_6	pim0071
LCDPAC02_1	pim0071
LCDPAC01_10	pim0071
LCMAC201_7	pim0071
LCPAC103_7	pim0071
LCMAC201_10	pim0224
LCMAC102_1	pim0224
LCPAC304_5	pim0224
LCMAC202_12	pim0224
LCMAC101_1	pim0224
LCMAC103_6	pim0224
LCDPAC01_16	pim0224
LCPAC401_2	pim0015
LCPAC403_1	pim0015
LCPAC104_1	pim0015
LCMAC101_5	pim0015
LCDPAC02_3	pim0015
LCPAC202_1	pim0015
LCPAC201_6	pim0015
LCPAC304_7	pim0015
LCPAC103_1	pim0015
LCMAC201_4	pim0015
LCIVAC01_1	pim0015
LCPAC101_2	pim0015
LCMAC102_1	pim0015
LCPAC406_7	pim0015
LCPAC404_1	pim0015
LCMAC103_8	pim0015
LCMAC202_11	pim0015
LCPAC001_6	pim0015
LCPAC302_19	pim0015
LCDPAC01_8	pim0015

PIM cluster annotations

pim0001	NCLDV major capsid protein
pim0002	DNA polymerase elongation subunit family B
pim0003	packaging ATPase
pim0004	DNA-directed RNA polymerase subunit alpha
pim0005	DNA-directed RNA polymerase subunit beta
pim0007	DNA-directed RNA polymerase subunit 5
pim0008	A18-like helicase
pim0011	Flap endonuclease
pim0013	Poxvirus Late Transcription Factor VLTF3
pim0014	Erv1/Alr family disulfide (thiol) oxidoreductase
pim0015	DNA topoisomerase II
pim0016	YqaJ viral recombinase
pim0019	DNA primase
pim0022	D5-like helicase-primase
pim0027	mRNA capping enzyme
pim0030	T4 phage RNA ligase with polynucleotide kinase domain
pim0031	RuvC, Holliday junction resolvases (HJRs)
pim0035	FtsJ-like methyltransferase
pim0042	Ribonuclease III
pim0053	ribonucleoside diphosphate reductase, large subunit
pim0054	ribonucleoside diphosphate reductase, beta subunit
pim0071	deoxynucleotide monophosphate kinase
pim0116	pfam03003: Pox_G9-A16 ; Pox virus entry-fusion-complex G9/A16
pim0134	uncharacterized transmembrane protein conserved in pithoviruses
pim0136	Poxvirus early transcription factor (VETF), large subunit
pim0142	L1R_F9L; Lipid membrane protein of large eukaryotic DNA viruses
pim0144	transcription initiation factor IIB
pim0192	protein kinase conserved in pithoviruses
pim0224	thermonuclease/nuclease

LCV contigs containing a translation protein and a 'bona fide' NCLDV protein, as defined by NCVOG and/or top blastp hits

LCMiAC02_3	D5-like helicase-primase (NCVOG0023)
LCMiAC01_1	NCLDV major capsid protein (NCVOG0022)
LCMiAC02_12	DNA-directed RNA polymerase subunit alpha (NCVOG0274)
LCMiAC01_18	DNA-directed RNA polymerase subunit alpha (NCVOG0274)
LCMiAC01_2	mRNA capping enzyme (NCVOG1451)
LCMiAC01_5	Phosphoinositide 3-kinase (NCVOG0242)
LCMiAC01_9	gene_16_LCMiAC01_9 is similar to TROVE domain protein [Klosneuvirus KNV1]
LCMiAC02_7	polynucleotide kinase-3'-phosphatase (NCVOG0243)
