# Supplementary material for: Virus Genomes from Deep Sea Sediments Expand the Ocean Megavirome and Support Independent Origins of Viral Gigantism
Source: mBio. 2019 Mar 5;10(2):e02497-18. doi: 10.1128/mBio.02497-18 (PMC6401483; doi:10.1128/mBio.02497-18)

Genome maps of Loki Castle virophages (contig names are in blue), some environmental virophages (wgs; contig names are in green), and reference virophages from GenBank. Green and blue triangles mark direct and inverse repeats. Pentagons with thicker outline represent conserved virophage genes.

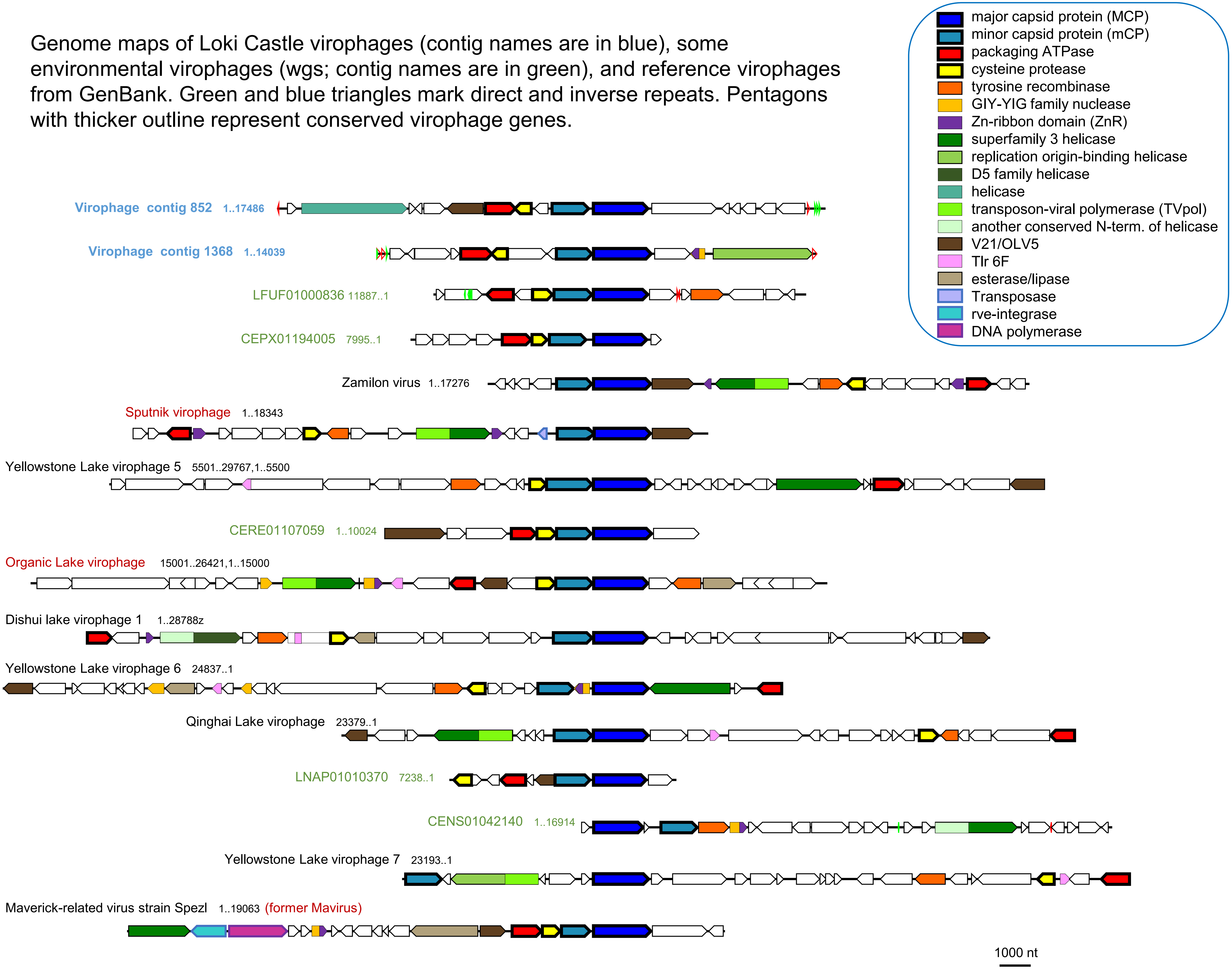

Supplement: TEXT S3 [file mBio.02497-18-s0003.pdf]
