# Supplementary figures and images for: Virus Genomes from Deep Sea Sediments Expand the Ocean Megavirome and Support Independent Origins of Viral Gigantism
Source: mBio. 2019 Mar 5;10(2):e02497-18. doi: 10.1128/mBio.02497-18 (PMC6401483; doi:10.1128/mBio.02497-18)

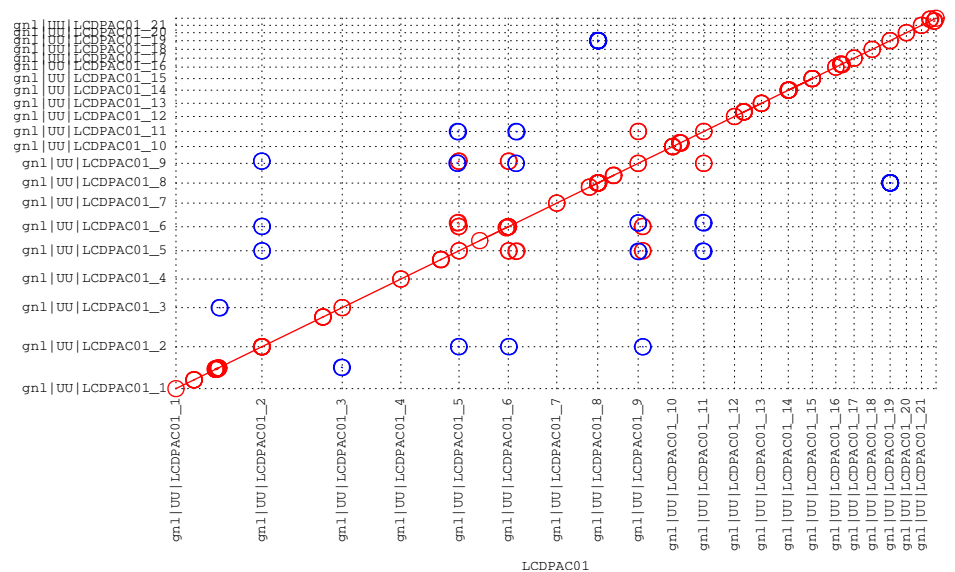

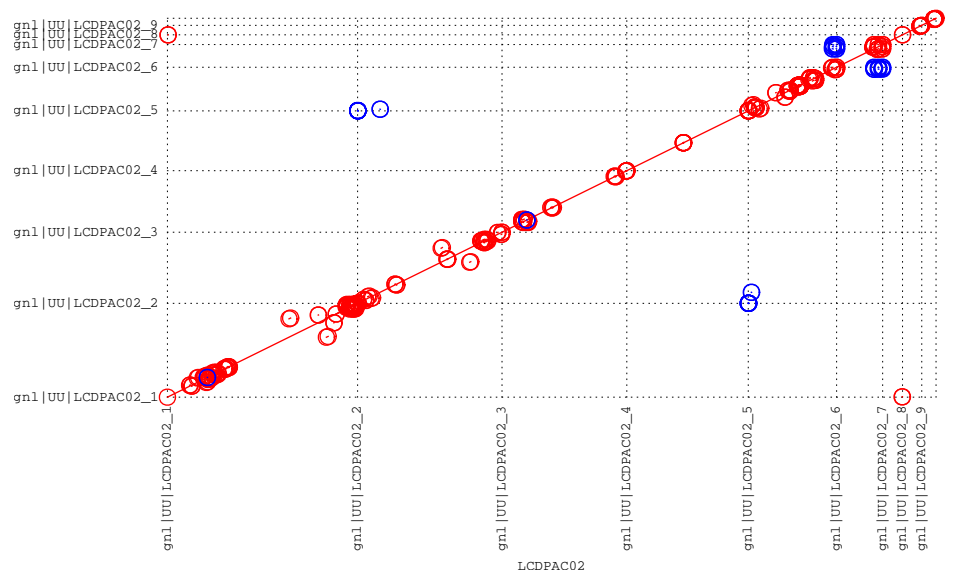

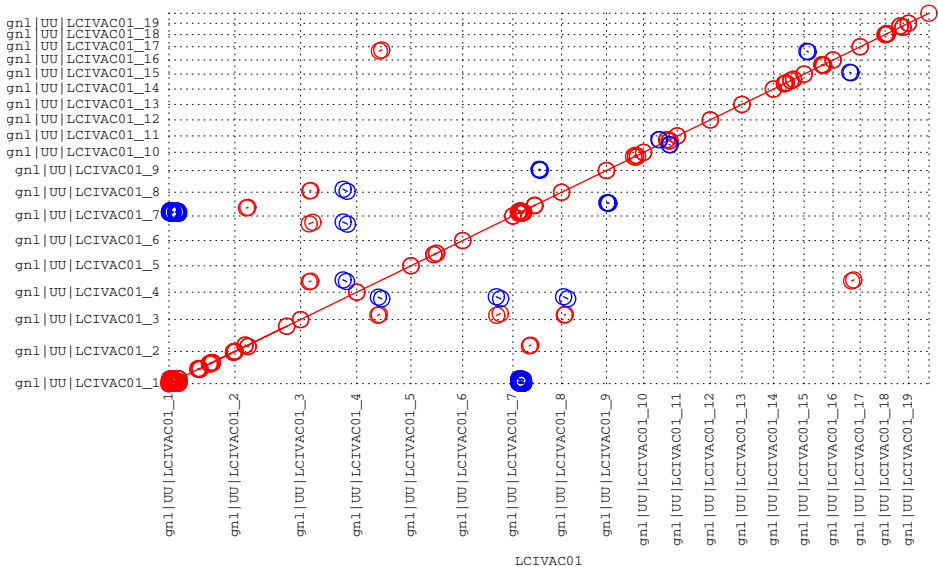

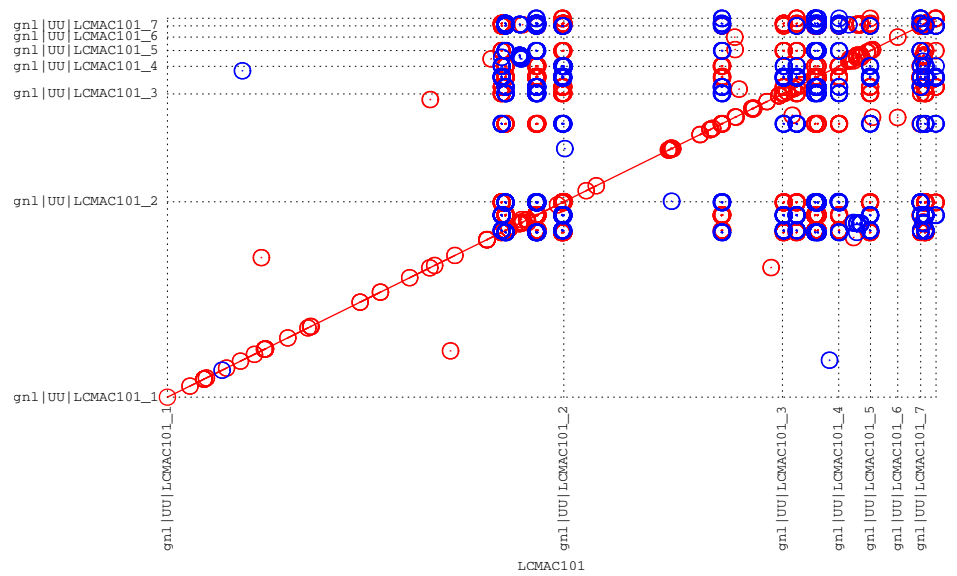

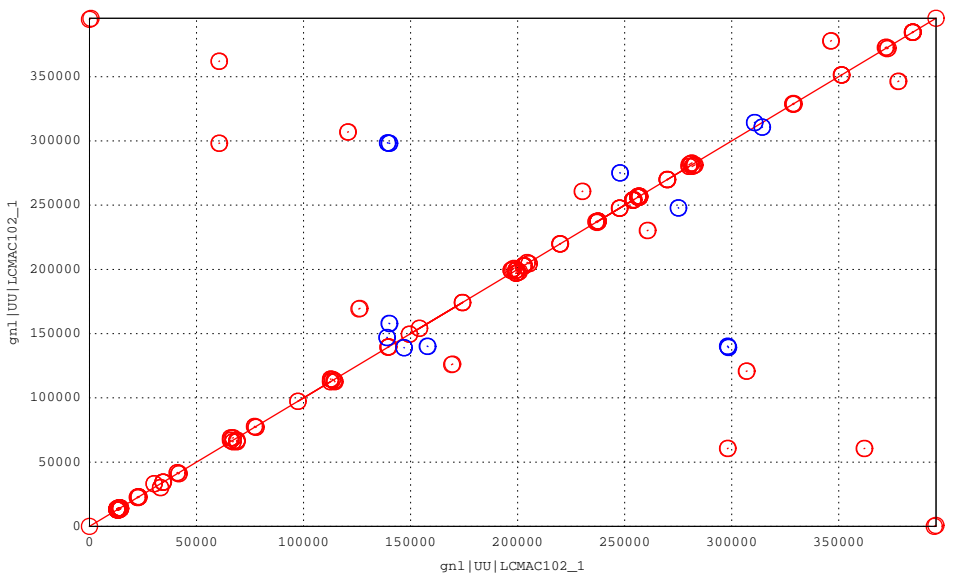

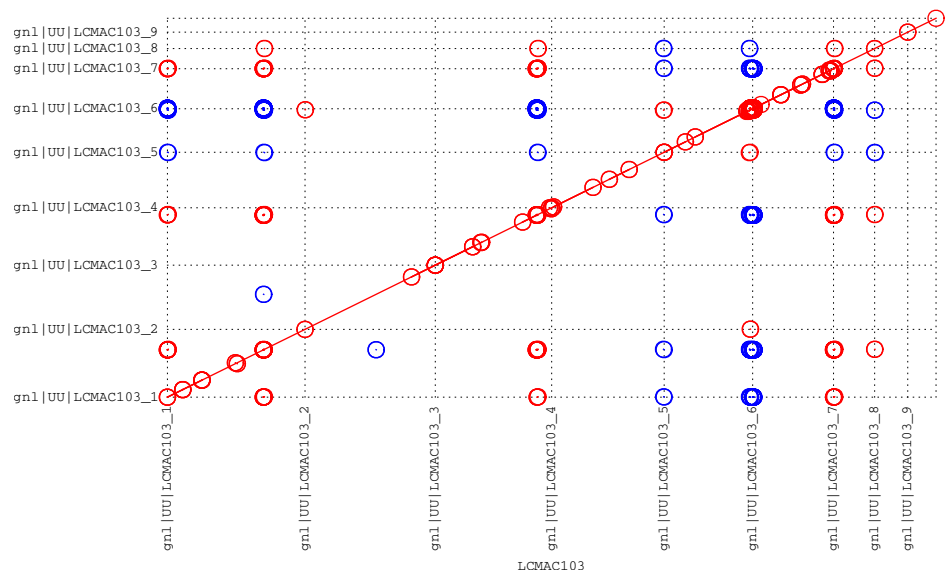

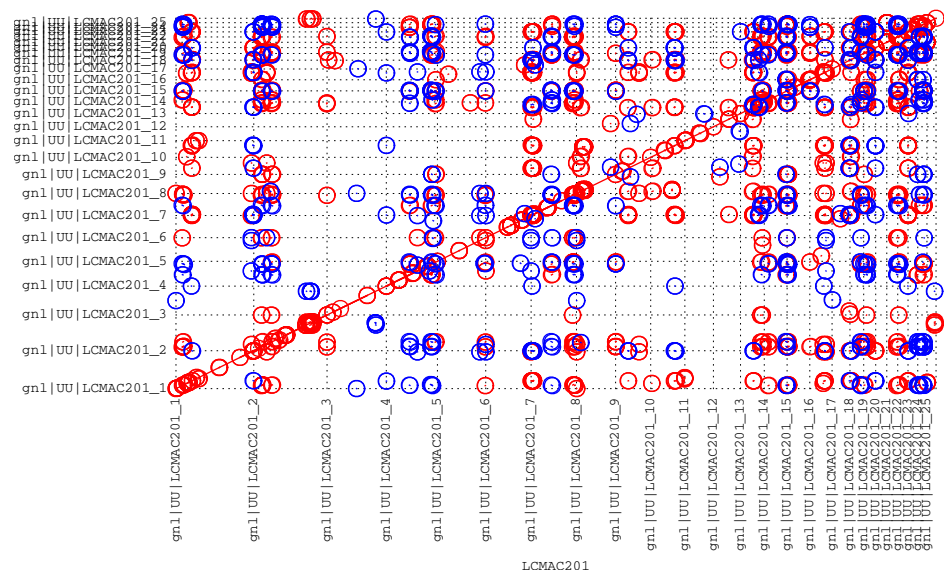



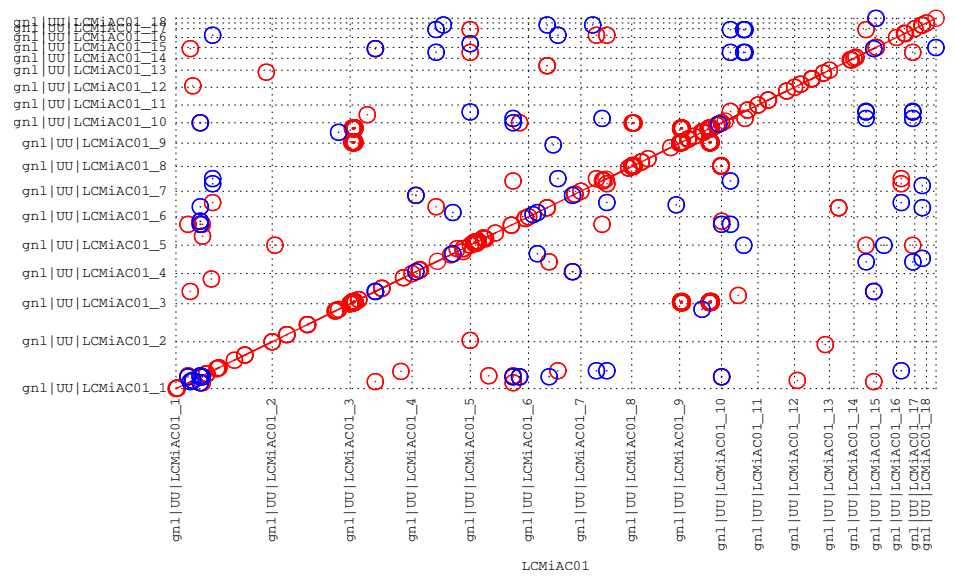

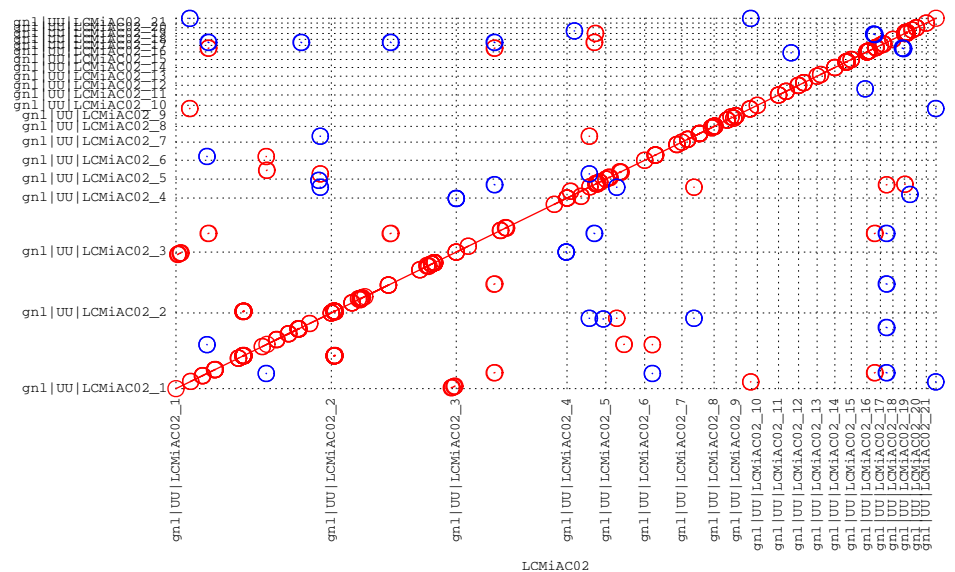

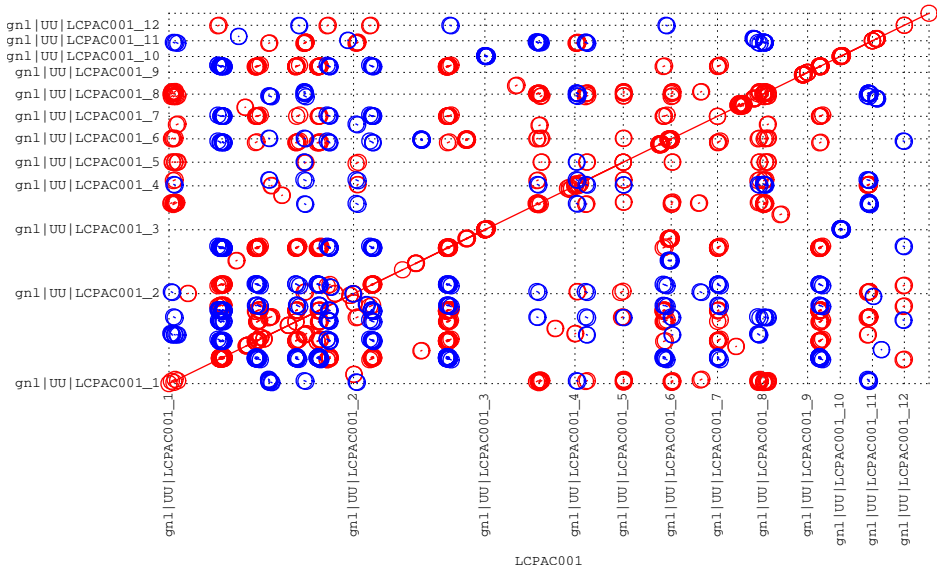

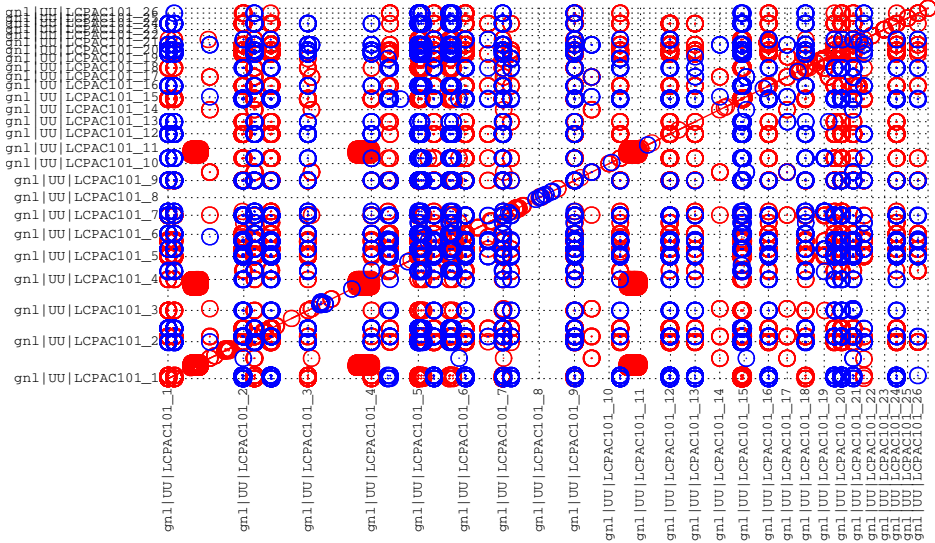

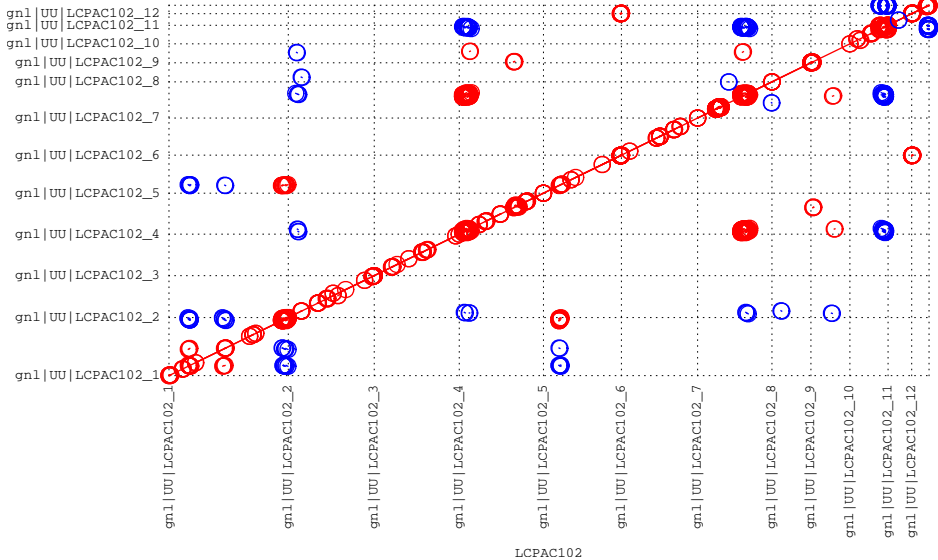

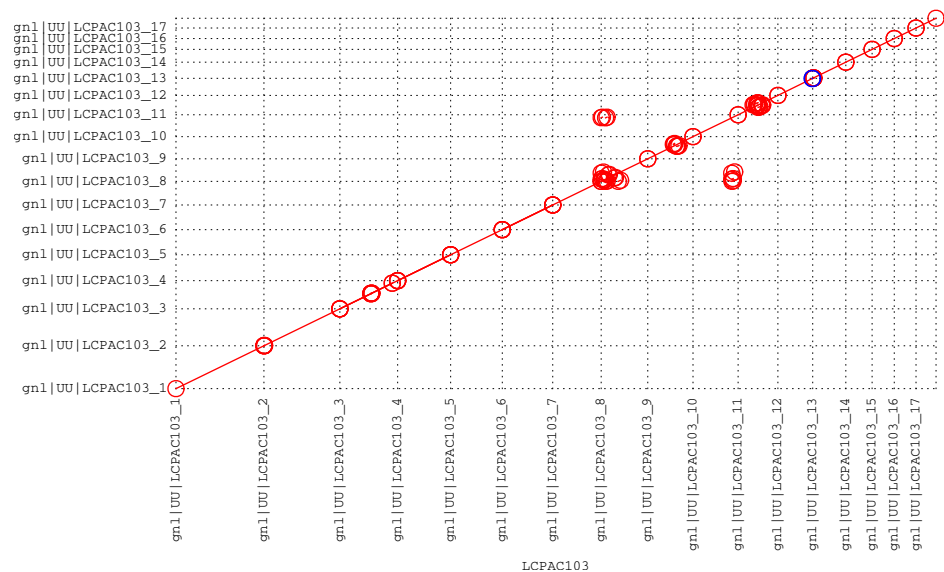

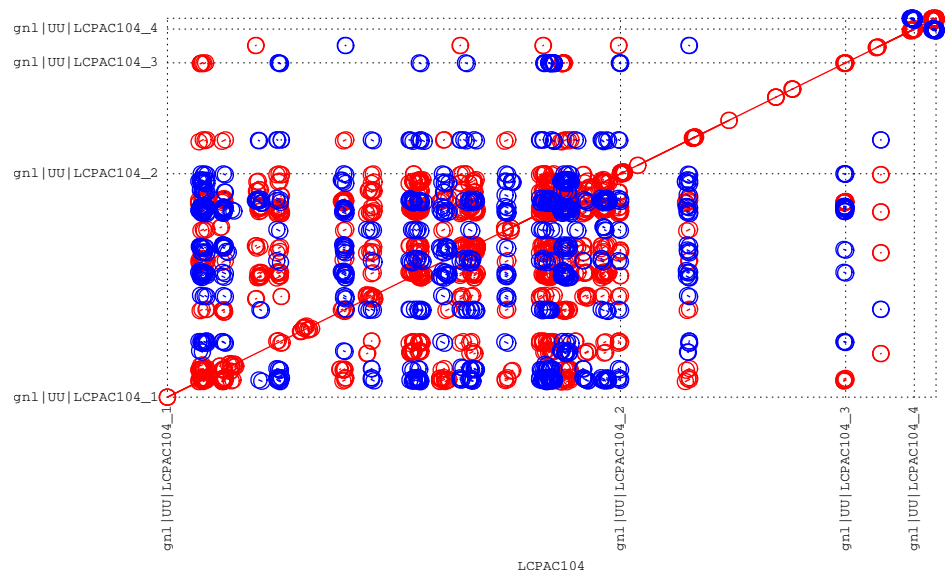

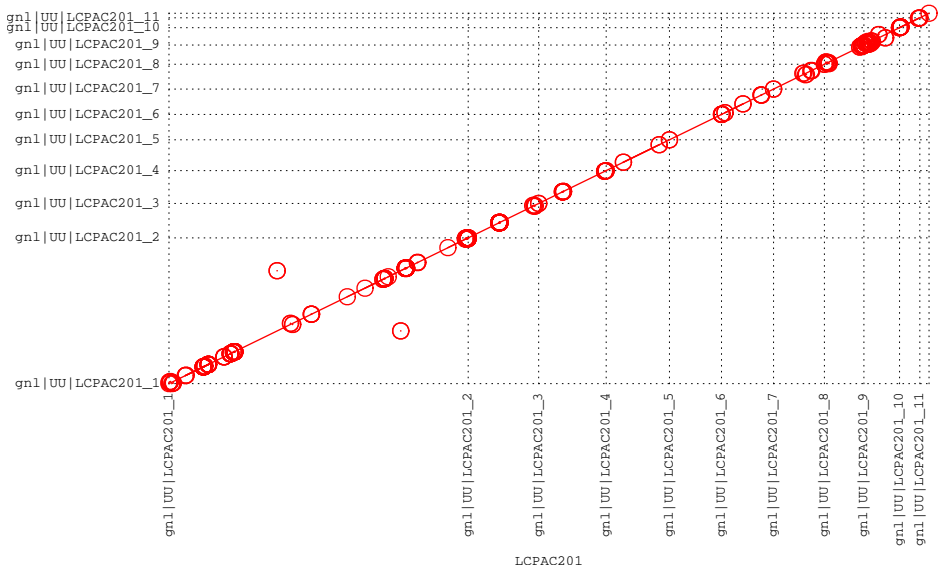

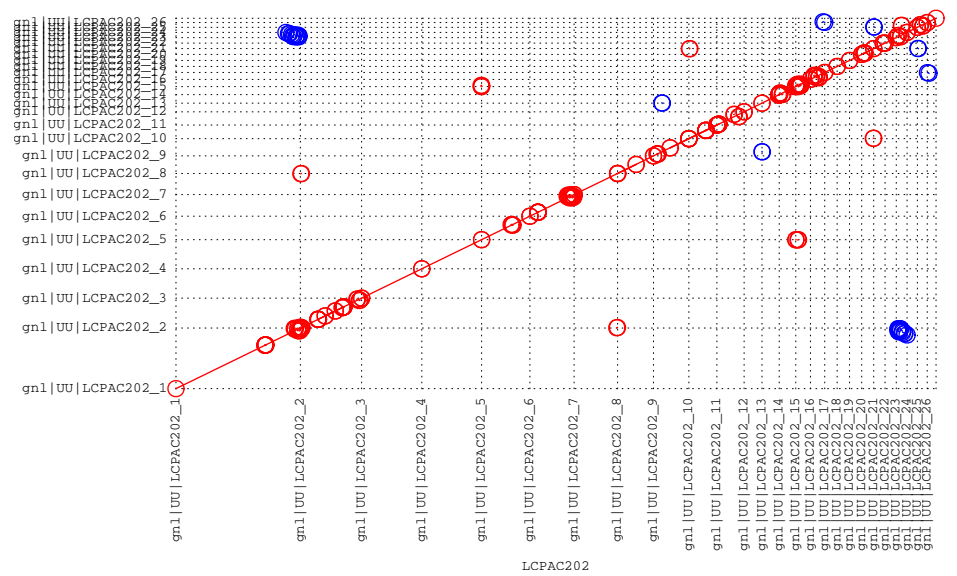

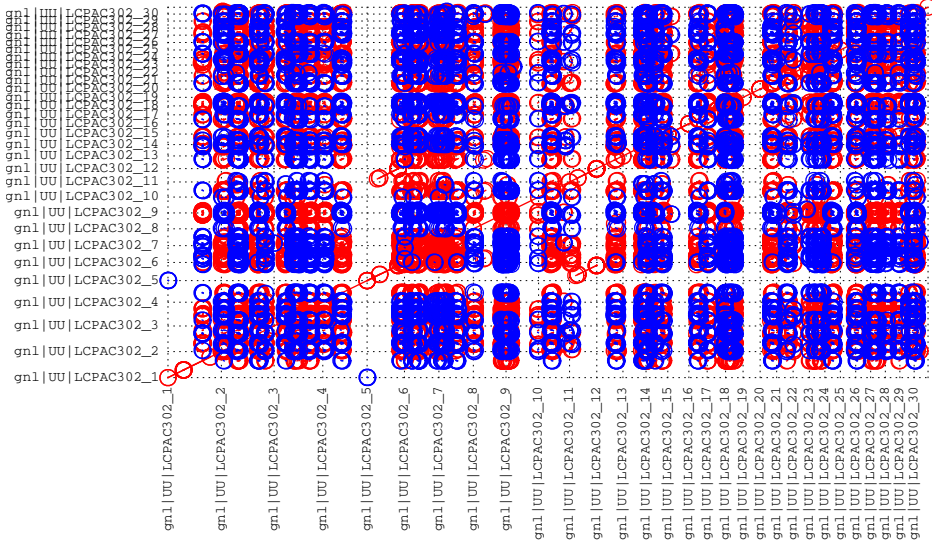

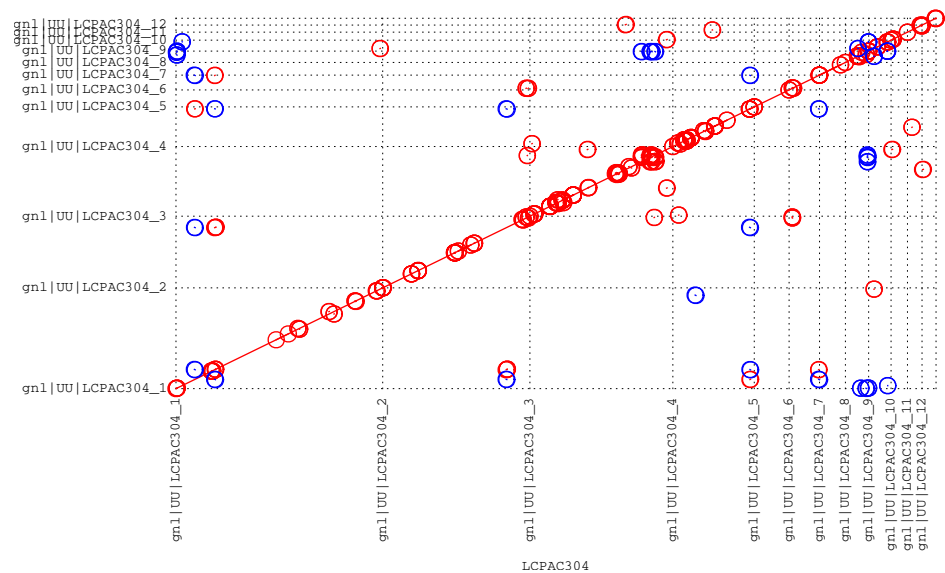

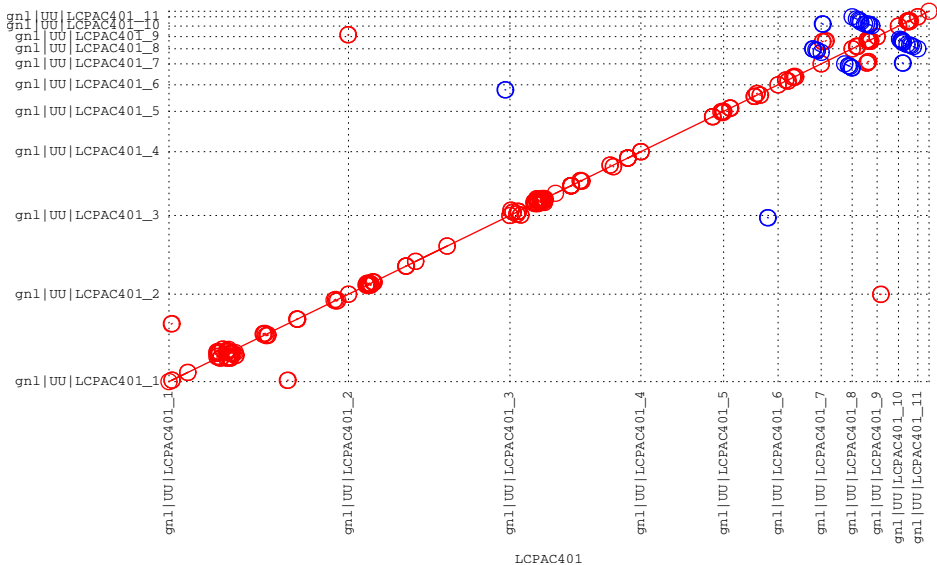

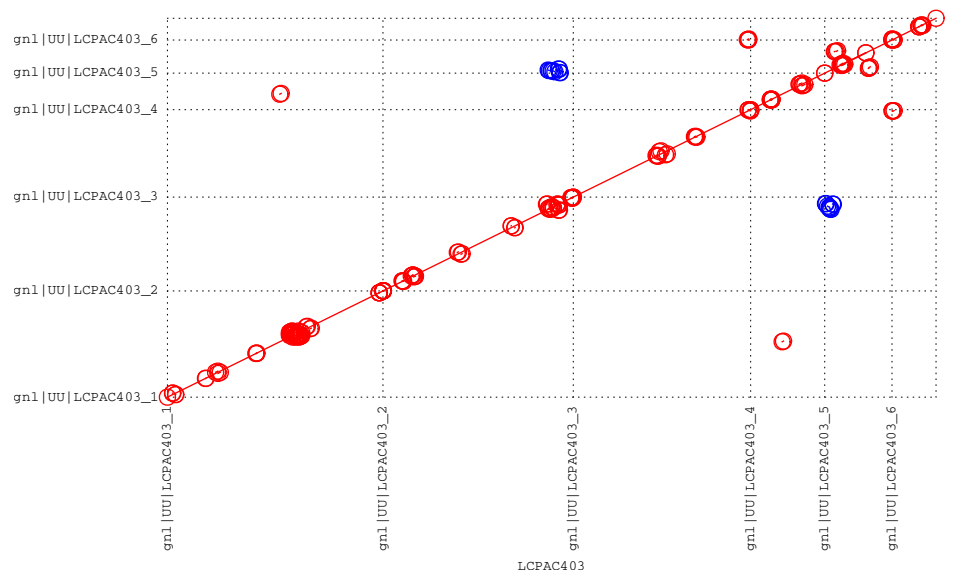

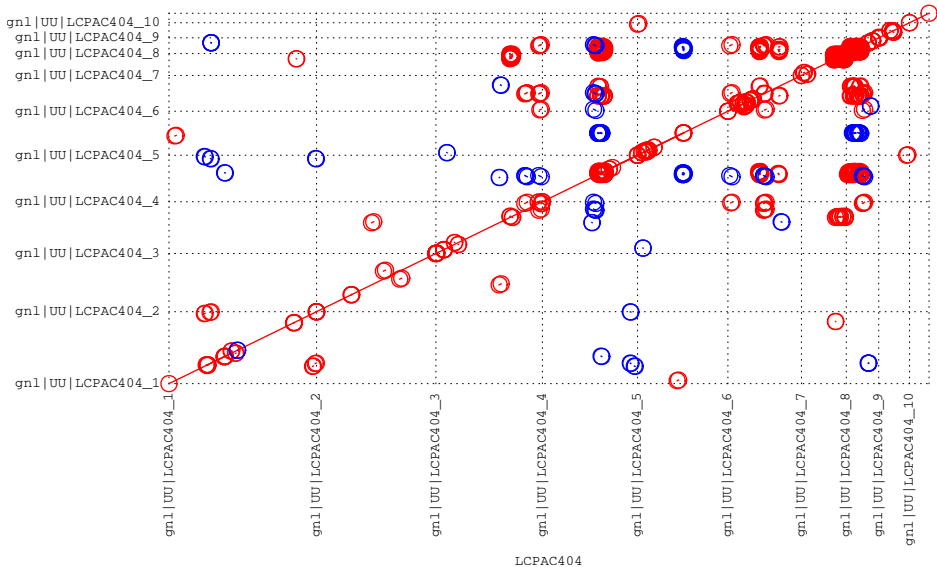

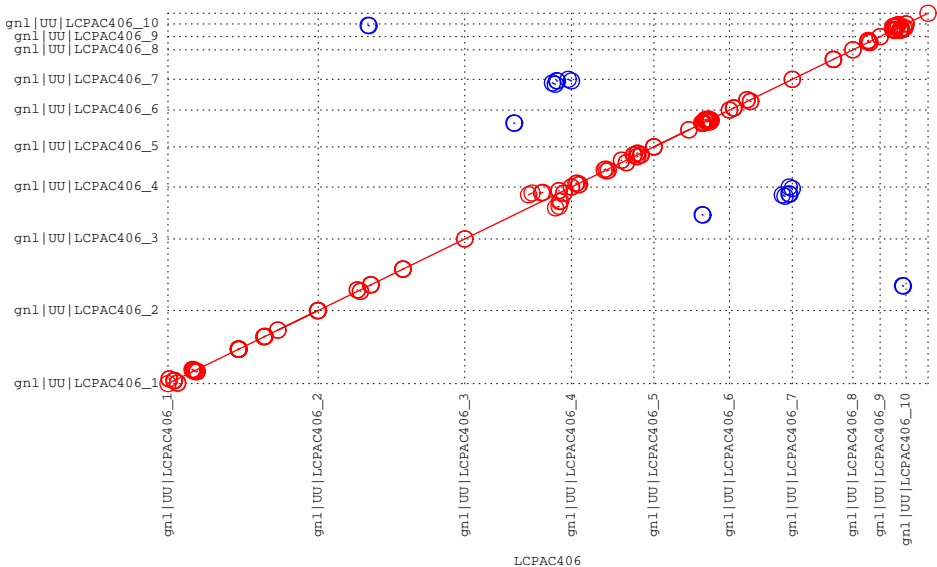

Supplement: TEXT S4 [file mBio.02497-18-s0004.pdf]
