# Supplementary material for: Virus Genomes from Deep Sea Sediments Expand the Ocean Megavirome and Support Independent Origins of Viral Gigantism
Source: mBio. 2019 Mar 5;10(2):e02497-18. doi: 10.1128/mBio.02497-18 (PMC6401483; doi:10.1128/mBio.02497-18)

This file contains data on frequencies of the most conserved motif (probable early promoter) through contigs of Loki Marseillevirus and Mimivirus bins (namely, LCMAC101,102,103, LCMAC201, 202, and LCMiAC01 and 02)

Loki Marseillevirus promoter LCMAC101, 102, 103

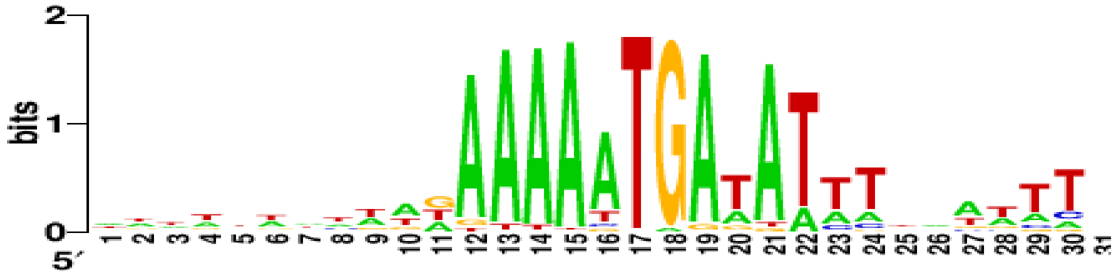

| bin/org | # of contigs | min contig length, nt | max contig length, nt | total genome length, nt | # of proteins |
|---|---|---|---|---|---|
| **LCMAC101** | 7 | 15190 | 393561 | 763048 | 793 |
| **LCMAC102** | 1 | 395459 | 395459 | 395459 | 465 |
| **LCMAC103** | 9 | 14346 | 69824 | 389984 | 427 |

| contig | upstream regions | upstr.reg.with promoter | percent |
|---|---|---|---|
| MAC102_1 | 464 | 208 | 45 |
| | | | |
| MAC101_1 | 392 | 140 | 36 |
| MAC101_2 | 231 | 66 | 29 |
| MAC101_3 | 62 | 24 | 39 |
| MAC101_4 | 32 | 16 | 50 |
| MAC101_5 | 26 | 3 | 12 |
| MAC101_6 | 29 | 9 | 31 |
| MAC101_7 | 15 | 8 | 53 |
| | | | |
| MAC103_1 | 82 | 34 | 41 |
| MAC103_2 | 61 | 23 | 38 |
| MAC103_3 | 57 | 27 | 47 |
| MAC103_4 | 75 | 31 | 41 |
| MAC103_5 | 43 | 15 | 35 |
| MAC103_6 | 47 | 17 | 36 |
| MAC103_7 | 25 | 15 | 60 |
| MAC103_8 | 15 | 7 | 47 |
| MAC103_9 | 17 | 6 | 35 |

Loki Marseillevirus promoter LCMAC101, 102, 103

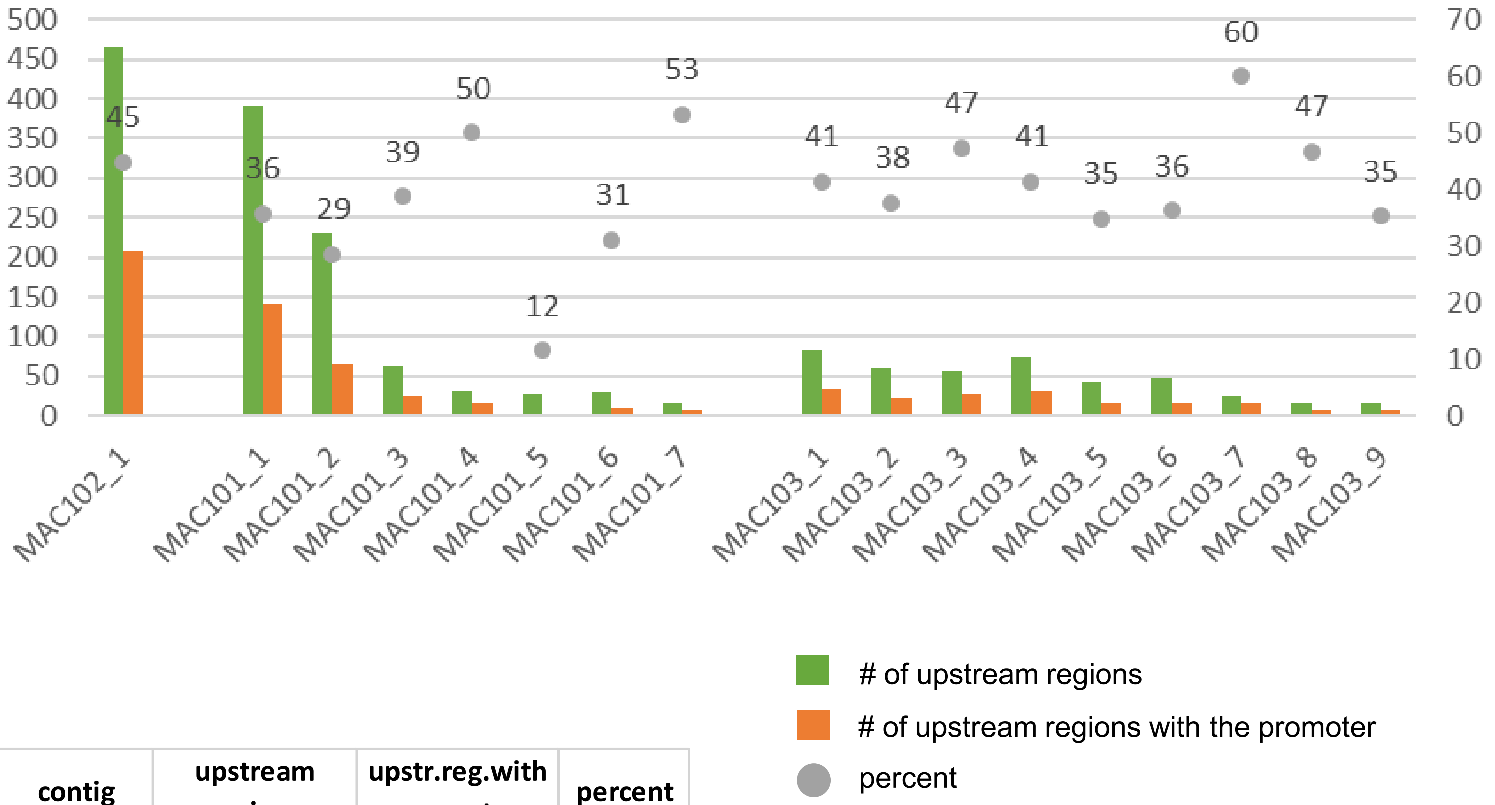


| contig | upstream regions | upstr.reg.with promoter | percent |
|---|---|---|---|
| MAC102_1 | 464 | 208 | 45 |
| | | | |
| MAC101_1 | 392 | 140 | 36 |
| MAC101_2 | 231 | 66 | 29 |
| MAC101_3 | 62 | 24 | 39 |
| MAC101_4 | 32 | 16 | 50 |
| MAC101_5 | 26 | 3 | 12 |
| MAC101_6 | 29 | 9 | 31 |
| MAC101_7 | 15 | 8 | 53 |
| | | | |
| MAC103_1 | 82 | 34 | 41 |
| MAC103_2 | 61 | 23 | 38 |
| MAC103_3 | 57 | 27 | 47 |
| MAC103_4 | 75 | 31 | 41 |
| MAC103_5 | 43 | 15 | 35 |
| MAC103_6 | 47 | 17 | 36 |
| MAC103_7 | 25 | 15 | 60 |
| MAC103_8 | 15 | 7 | 47 |
| MAC103_9 | 17 | 6 | 35 |

Loki Marseillevirus promoter

LCMAC201, 202 – “deep” marseilleviruses

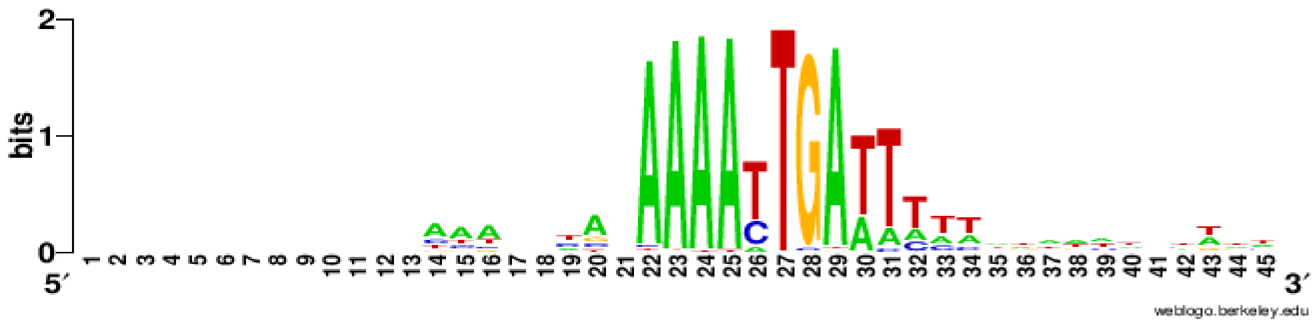


| bin/org | # of contigs | min contig length, nt | max contig length, nt | total genome length, nt | # of proteins |
|---|---|---|---|---|---|
| **LCMAC201** | 25 | 6728 | 57873 | 565697 | 566 |
| **LCMAC202** | 19 | 6906 | 153726 | 705352 | 672 |

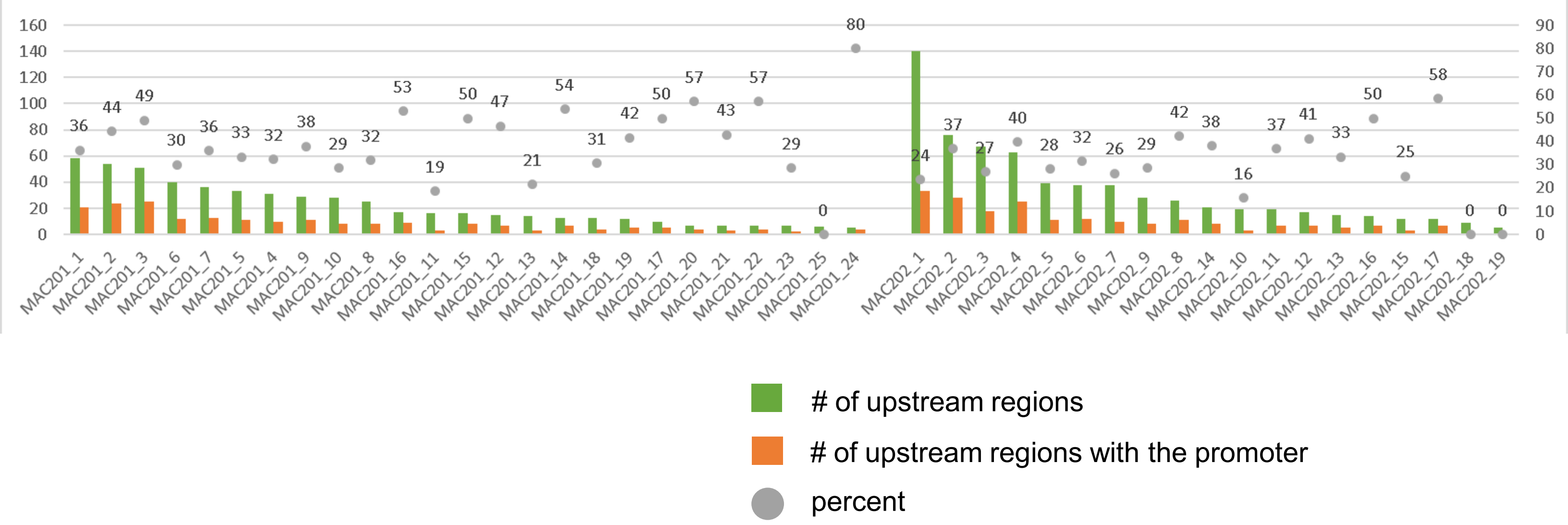

Loki Mimivirus promoter LCMiAC01, 02

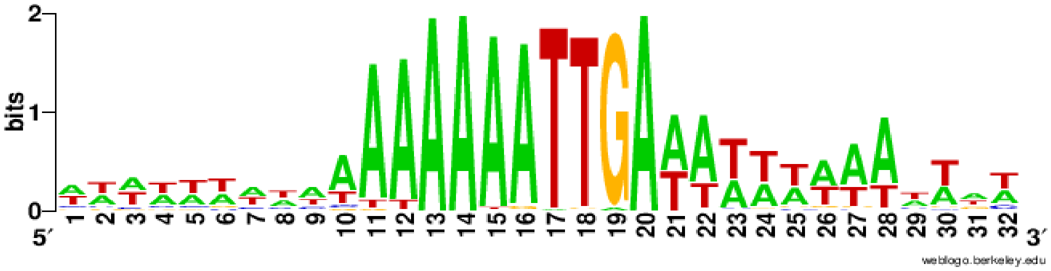


| bin/org | # of contigs | min contig length, nt | max contig length, nt | total genome length, nt | # of proteins |
|---|---|---|---|---|---|
| **LCMiAC01** | 18 | 8458 | 85120 | 672112 | 571 |
| **LCMiAC02** | 21 | 8237 | 131456 | 642939 | 583 |

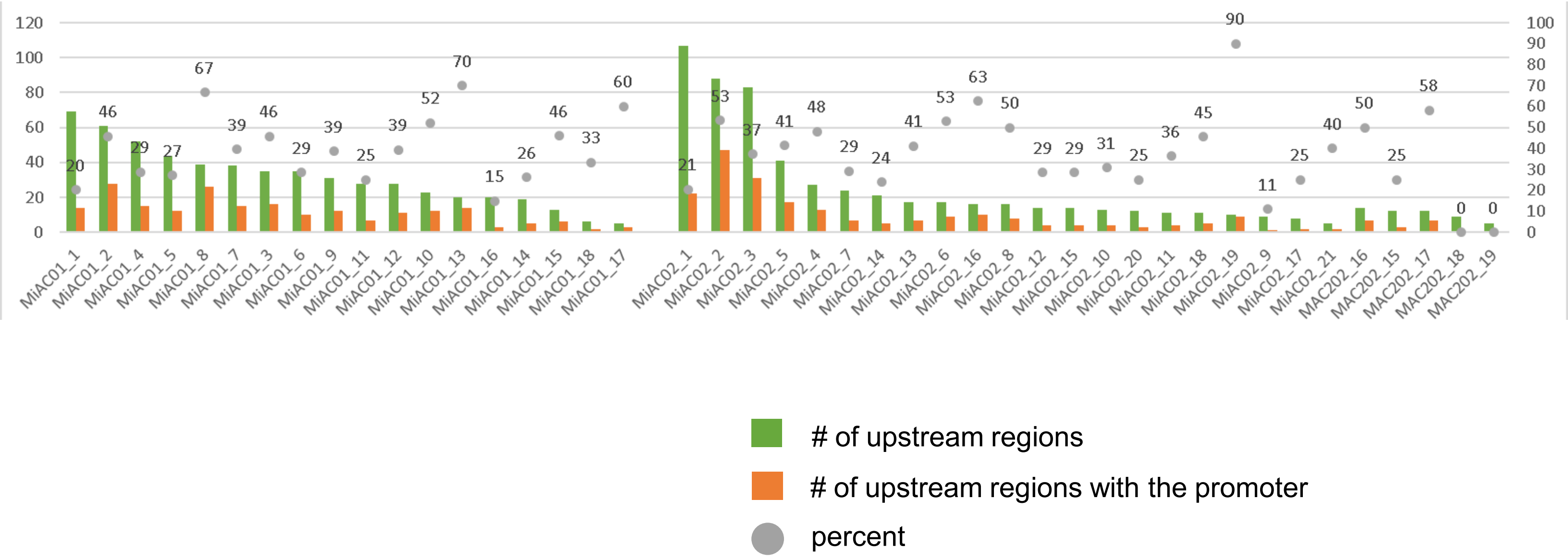

Supplement: TEXT S5 [file mBio.02497-18-s0005.pdf]
