# Supplementary material for: Virus Genomes from Deep Sea Sediments Expand the Ocean Megavirome and Support Independent Origins of Viral Gigantism
Source: mBio. 2019 Mar 5;10(2):e02497-18. doi: 10.1128/mBio.02497-18 (PMC6401483; doi:10.1128/mBio.02497-18)

This file contains meme motif search results for Loki Castle Viruses of Mimivirus group, and for Loki Castle Virophages

The ‘upstream’ regions are from -250 to 30 nt related to the start codon; the regions shorter than 50 nt are filtered out

Meme search was set to 25 nt of motif width

Upstream region sequences and meme output files can be found at:
ftp://ftp.ncbi.nih.gov/pub/yutinn/Loki_Castle_NCLDV_2018/meme_motif_search

Conserved motifs AAA(T/A)TGA are marked with the ‘sun’ sign
Conserved AT-rich motifs are marked with the ‘moon’ sign

note: on all Logo images, the detected motif starts from position #11

LCMiAC01, LCMiAC02

1130 fragments

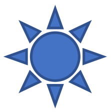

Motif #3 (399 sites)

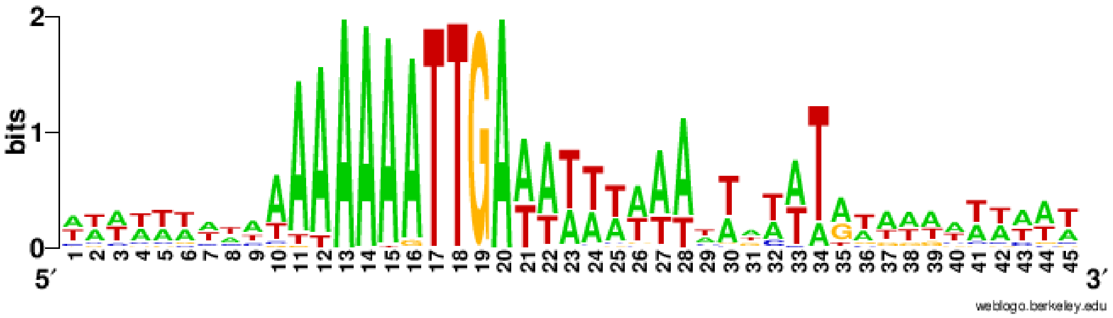

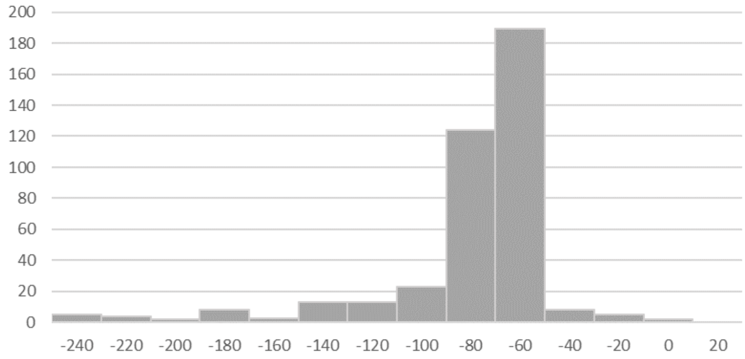

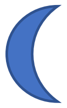

Motif #4 (425 sites)

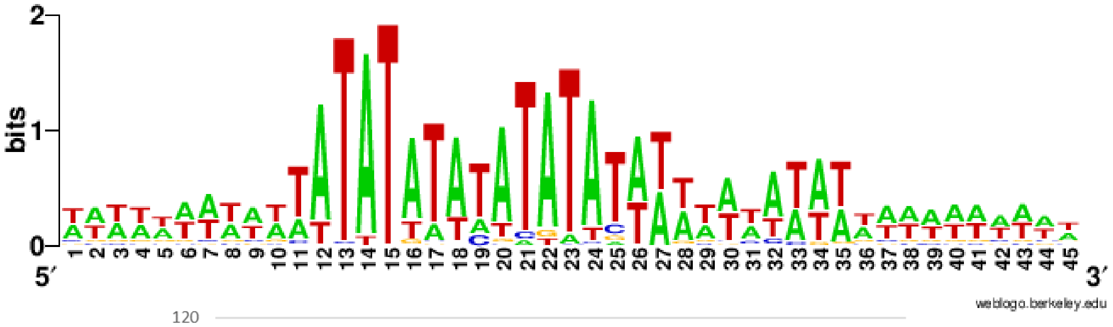

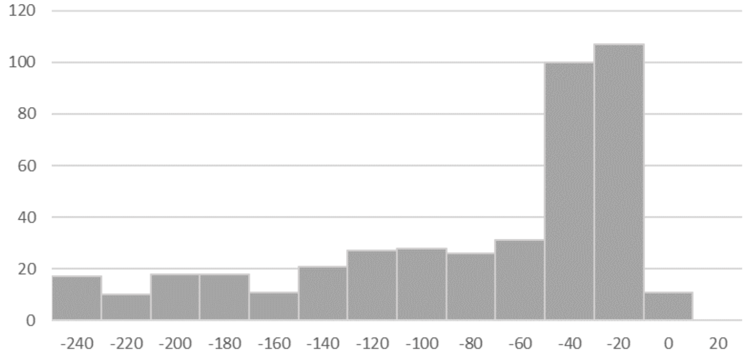

LCMiAC01

566 fragments

Motif #2 (437 sites)

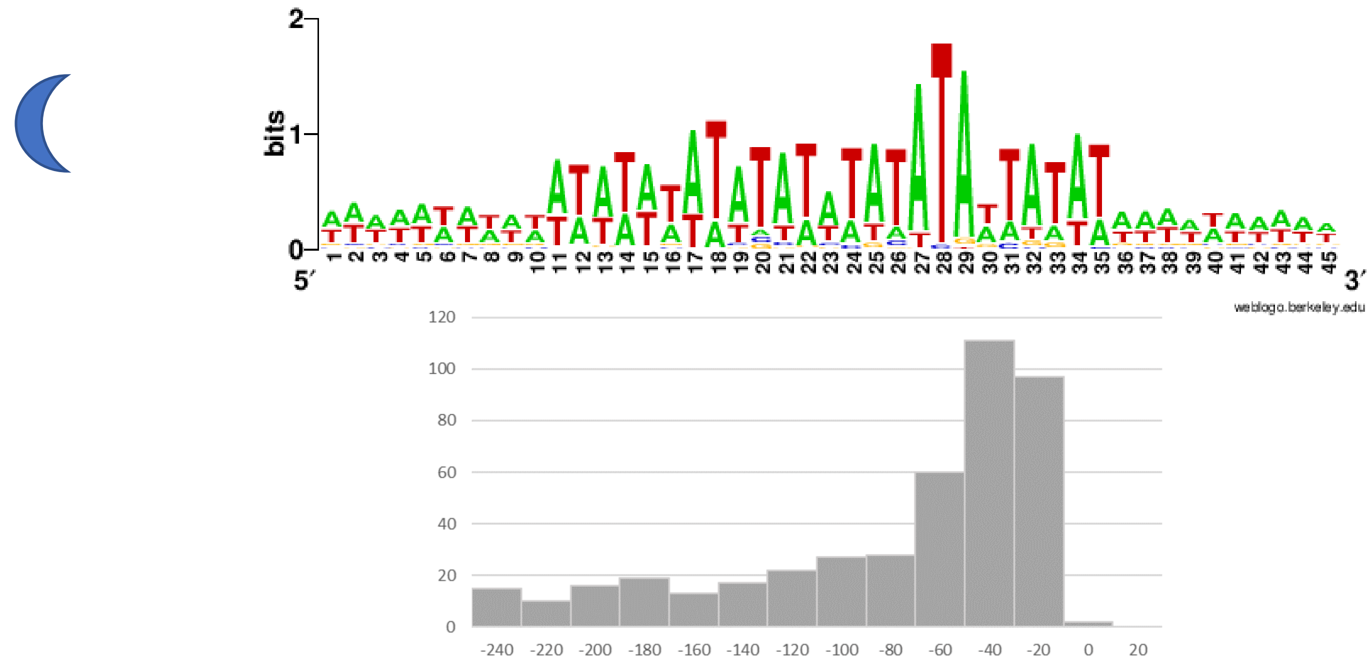

LCMiAC02

564 fragments

Motif #1 (223 sites)

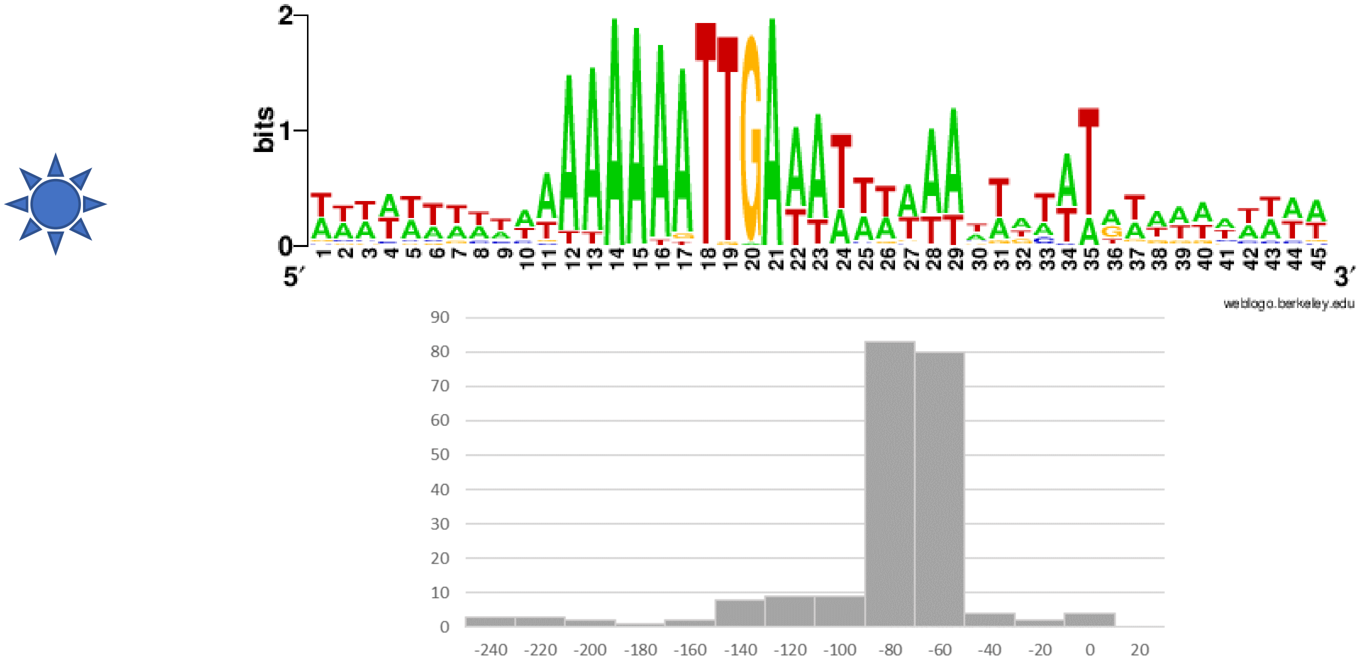

Viro 1368, motif 7
12 out of 13 ORFs

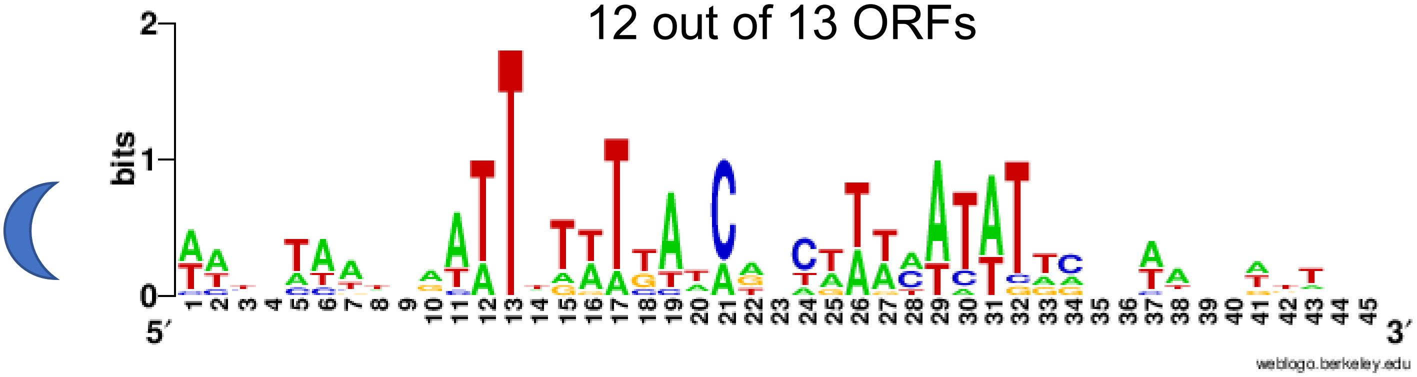

Viro 852, motif 2
12 out of 17 ORFs

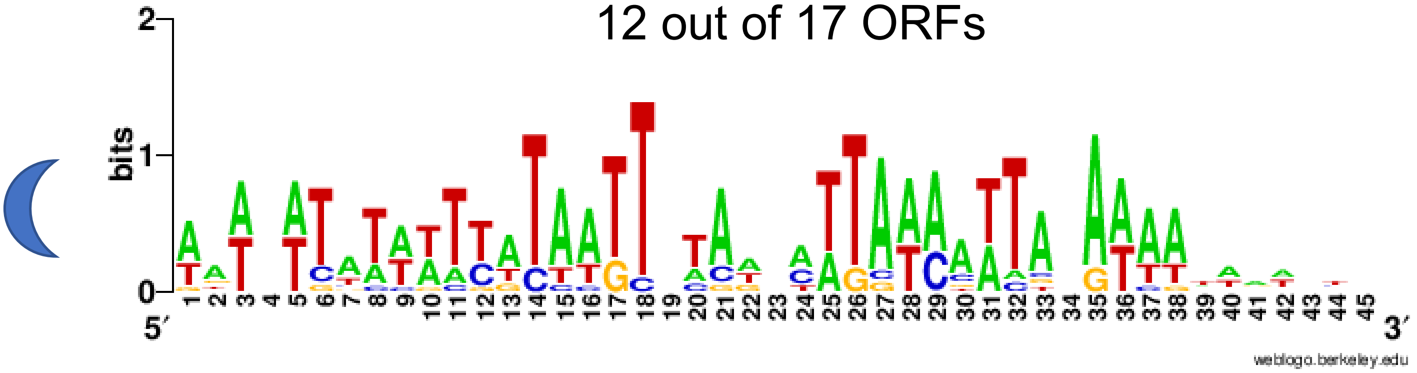

virophages searched together
(30 out of 30 ORFs)

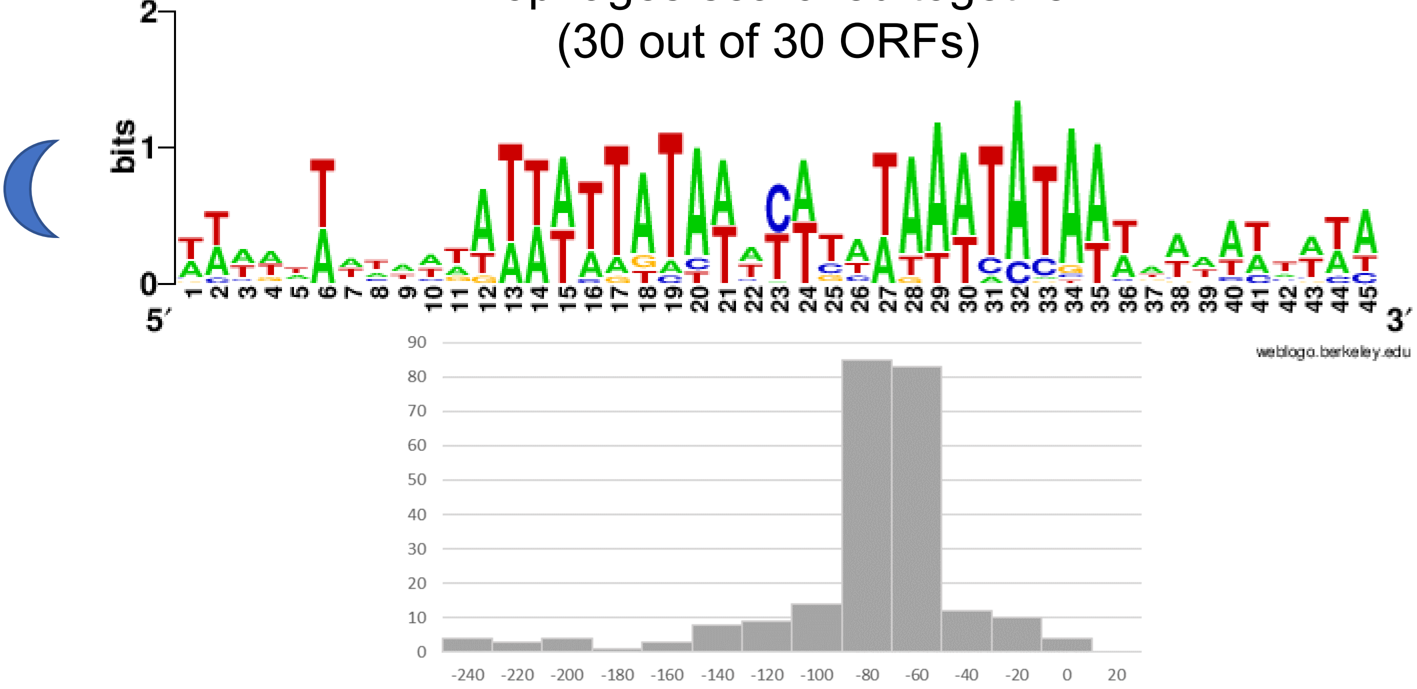

Viro 1368 and viro 852 conserved motifs vs MiAC upstream regions (by FIMO)

Viro 1368

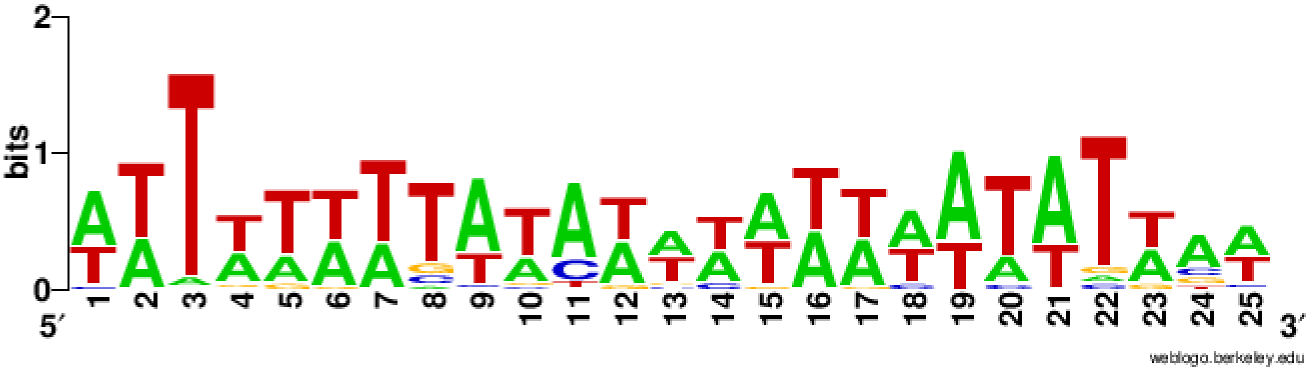

Viro 852

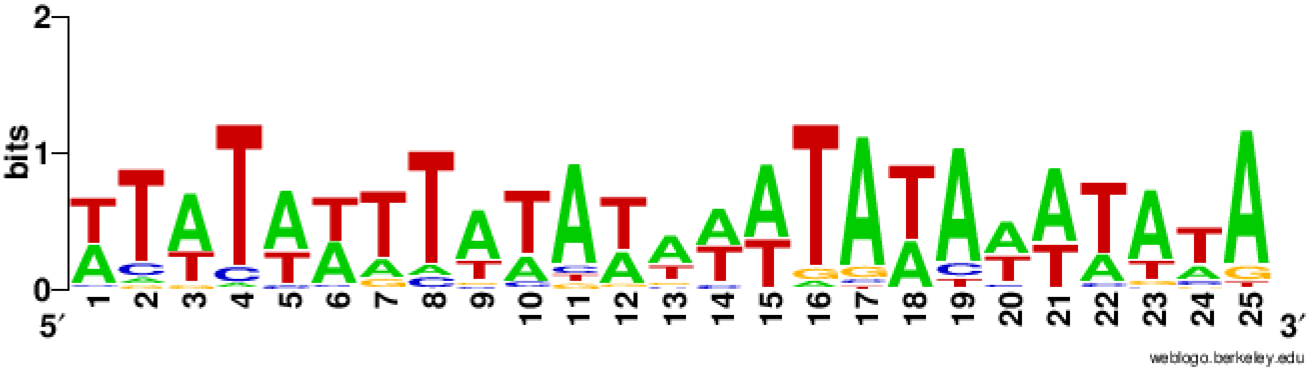

Supplement: TEXT S6 [file mBio.02497-18-s0006.pdf]
