# Supplementary material for: Virus Genomes from Deep Sea Sediments Expand the Ocean Megavirome and Support Independent Origins of Viral Gigantism
Source: mBio. 2019 Mar 5;10(2):e02497-18. doi: 10.1128/mBio.02497-18 (PMC6401483; doi:10.1128/mBio.02497-18)

This file contains meme motif search results for Loki Castle Viruses of Pithovirus group

Pithovirus sibericum (NC_023423.1), Cedratvirus A11 (NC_032108.1), and Orpheovirus (NC_036594.1) are included

The ‘upstream’ regions are from -250 to 30 nt related to the start codon; the regions shorter than 50 nt are filtered out

Meme search was set to 25 nt of motif width

Upstream region sequences and meme output files can be found at:
ftp://ftp.ncbi.nih.gov/pub/yutinn/Loki_Castle_NCLDV_2018/meme_motif_search

Conserved motifs AAA(T/A)TGA are marked with the ‘sun’ sign
Conserved AT-rich motifs are marked with the ‘moon’ sign

*note: on all Logo images, the detected motif starts from position #11*

# LCPAC201_202

654 fragments

## Motif #1 (218 sites)

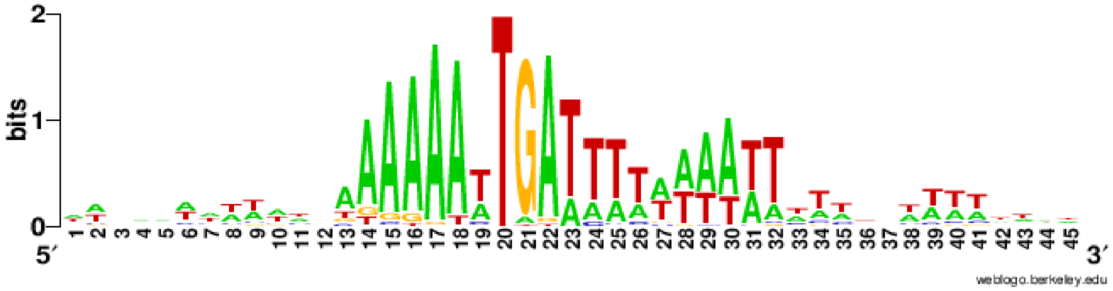


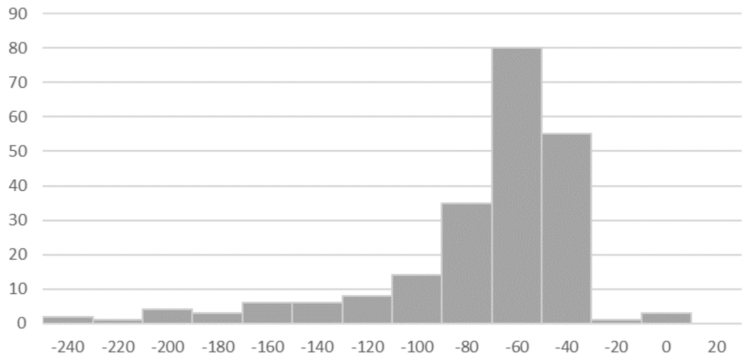


## Motif #2 (174 sites)

*start codon*

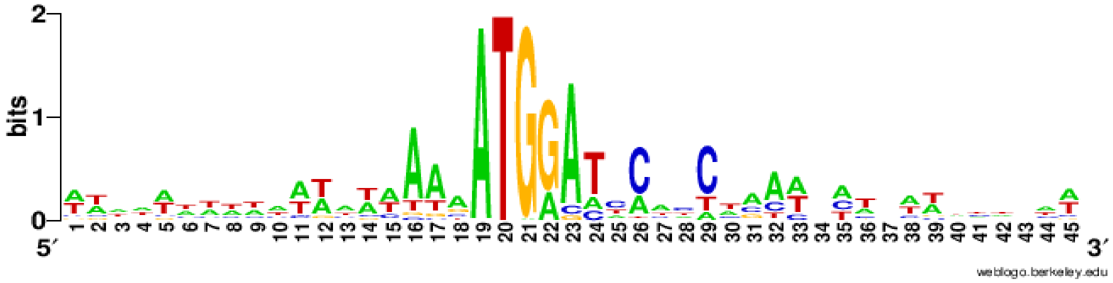


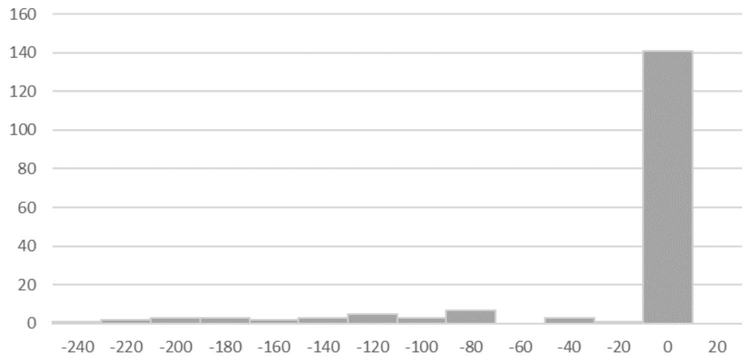


*note: on all Logo images, the detected motif starts from position #11*

**LCPAC201_202**

654 fragments

Motif #3 (70 sites)

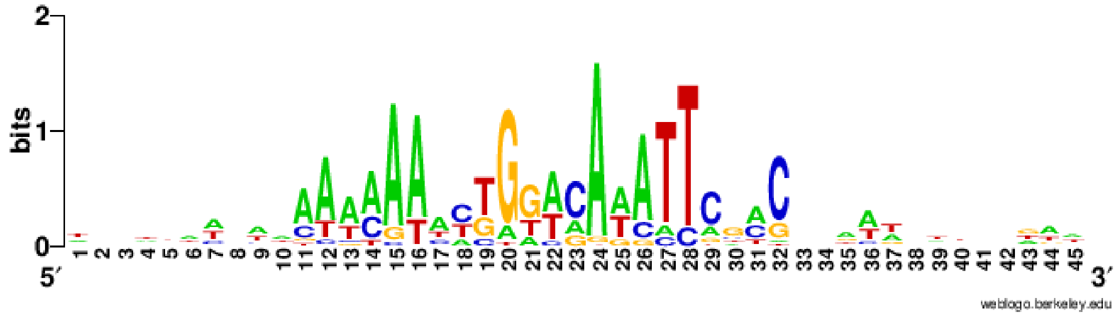

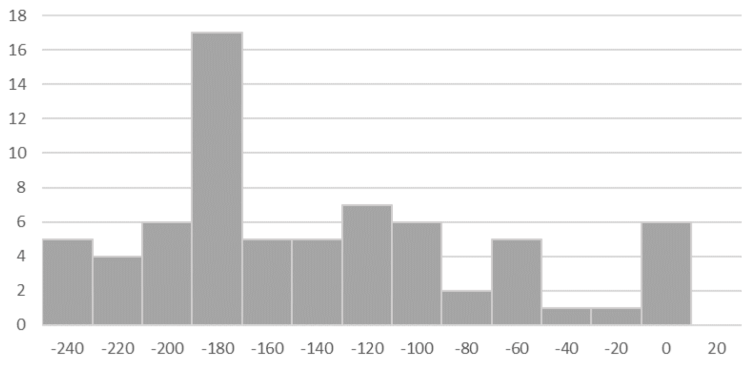

Motif #4 (70 sites)

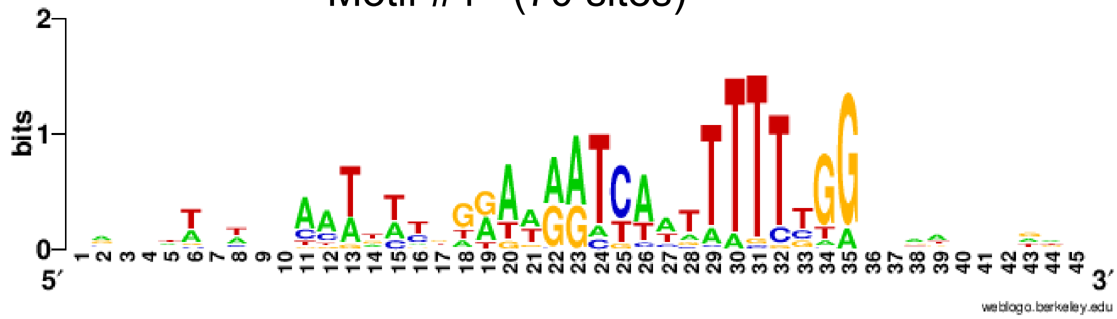

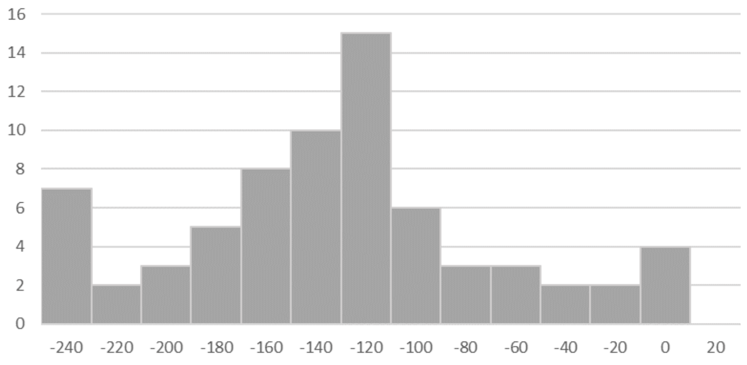

# LCPAC201_202

654 fragments

## Motif #5 (73 sites)

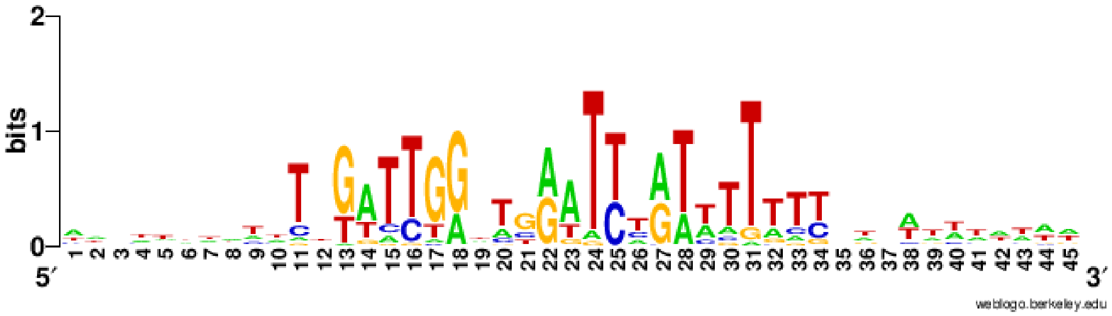


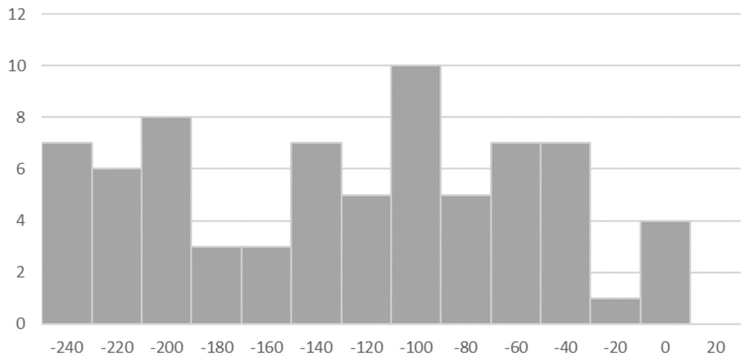


## Motif #6 (70 sites)

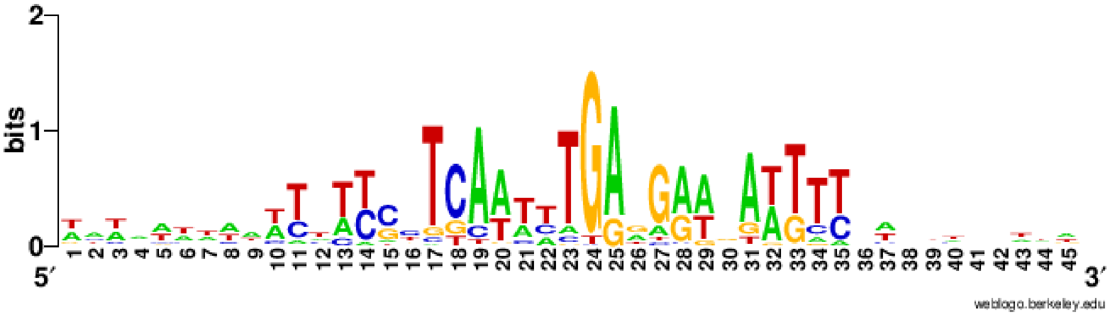


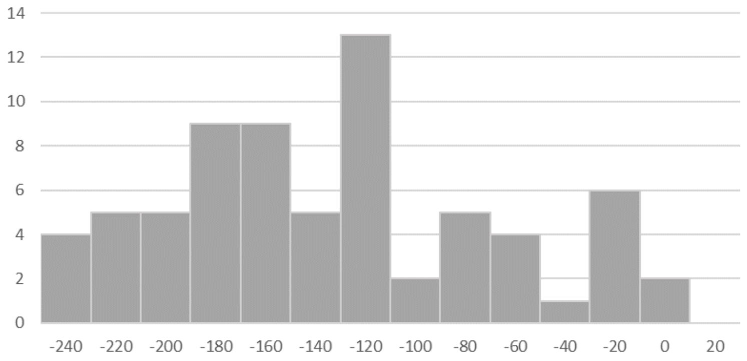

**LCPAC104**

763 fragments

## Motif #1 (54 sites)

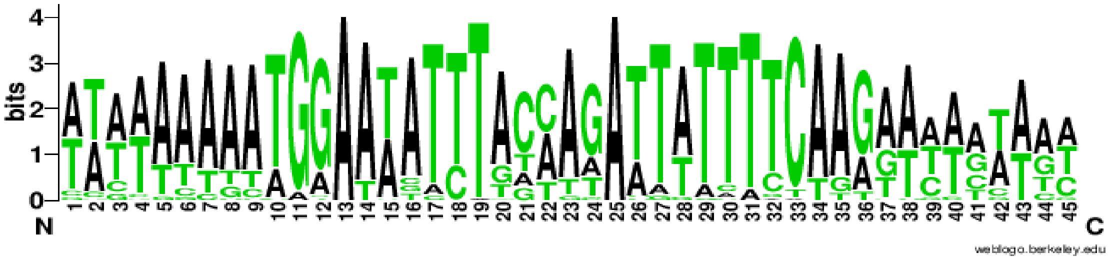


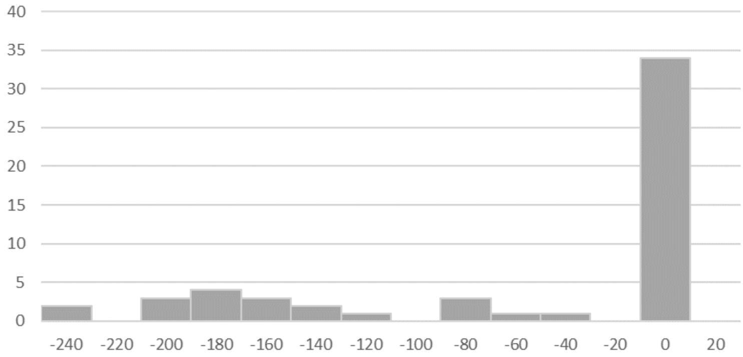


## Motif #2 (86 sites)

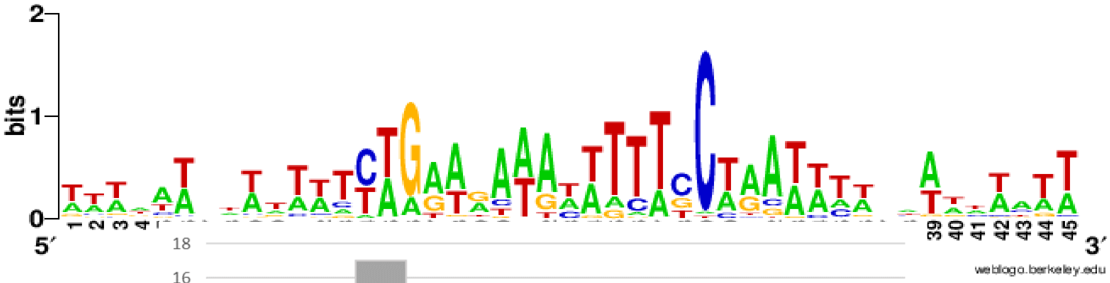


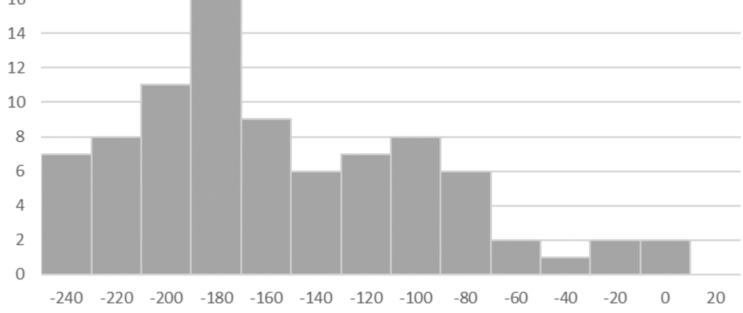

**LCPAC104**

763 fragments

## Motif #3 (151 sites)

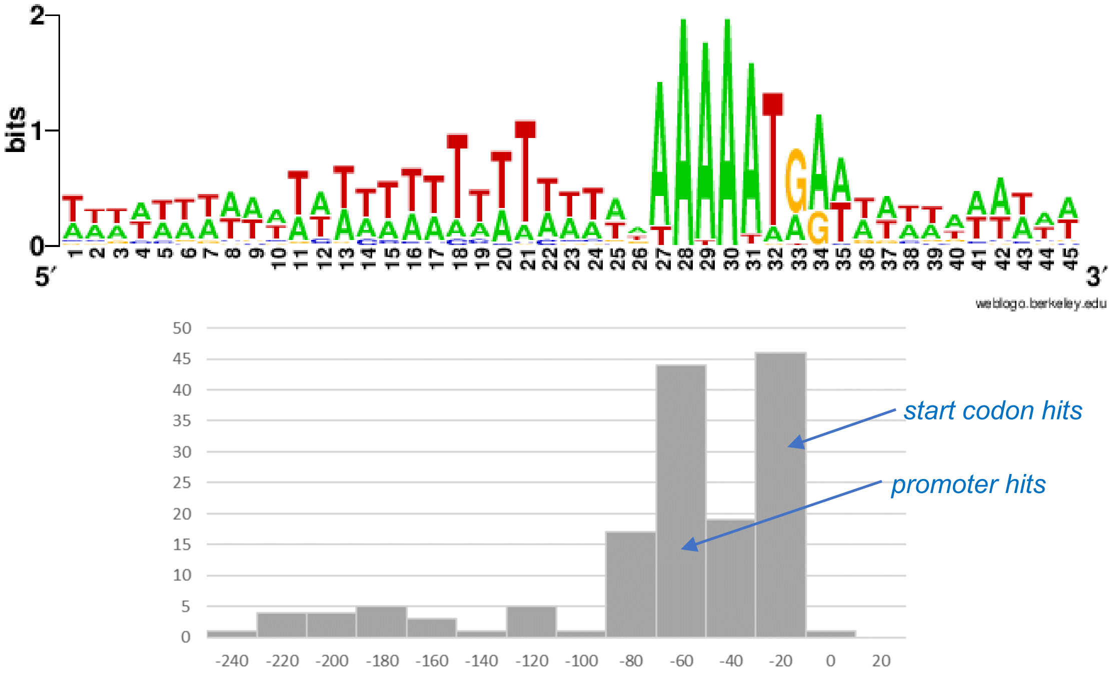


## Motif #7 (53 sites)

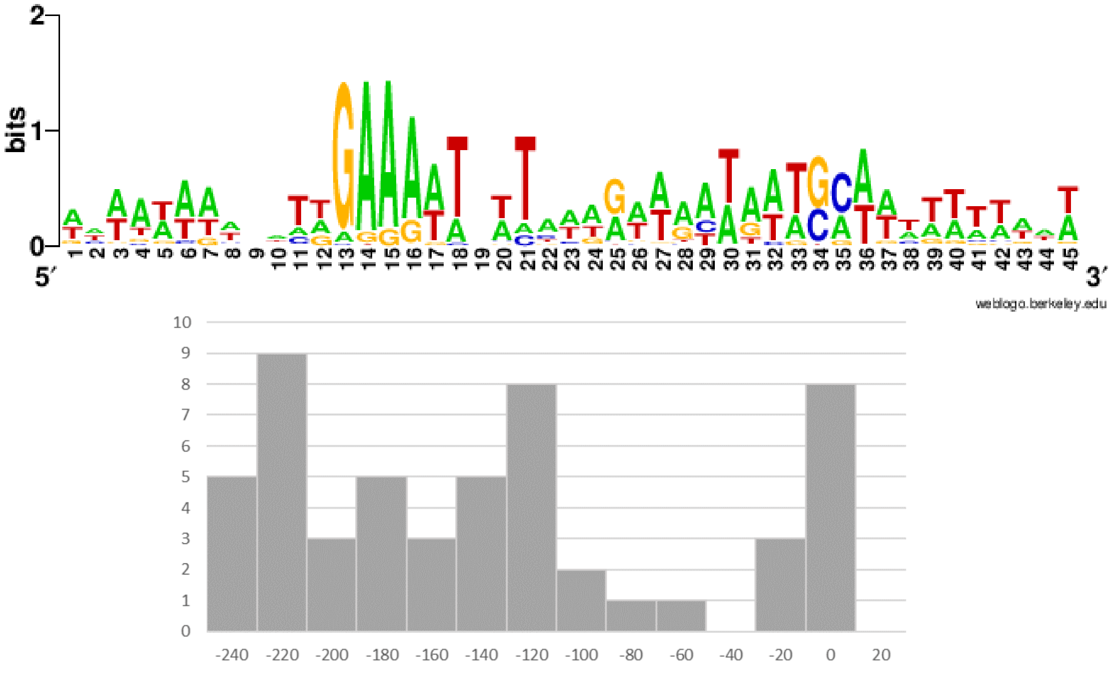

# LCPAC101_102_103

763 fragments

## Motif #1 (247 sites)

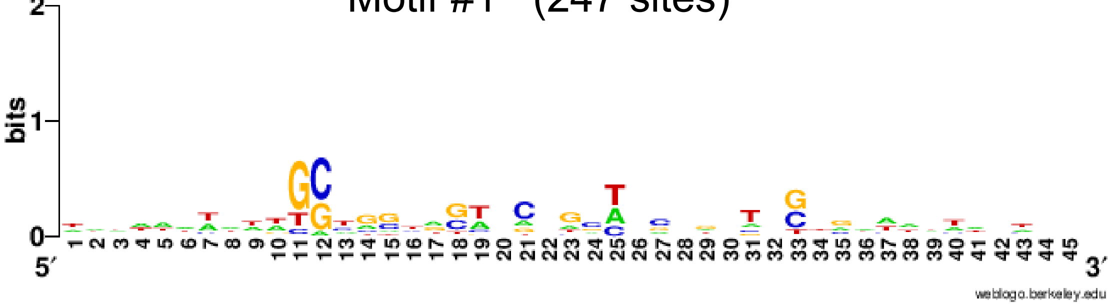


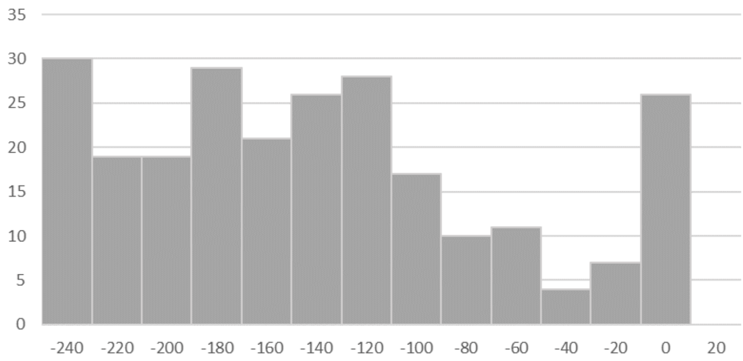


## Motif #2 (540 sites)

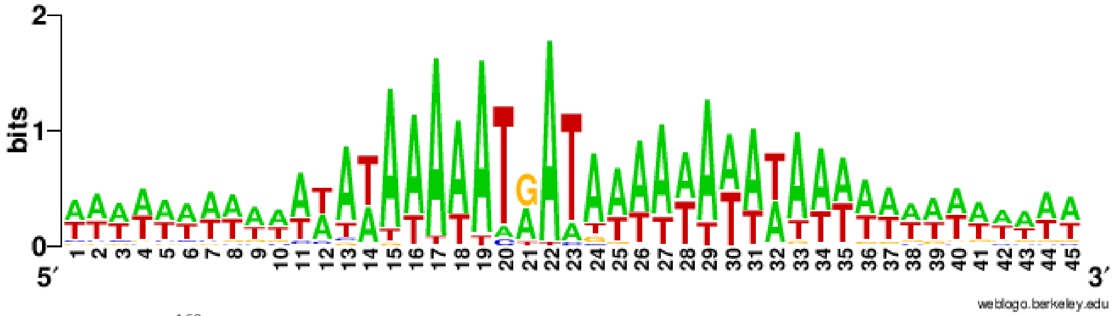


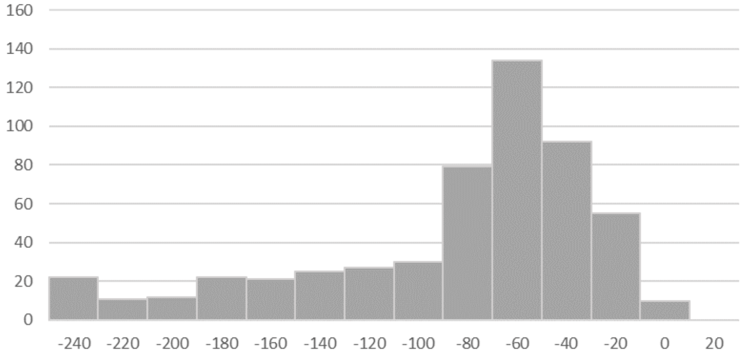

# LCPAC101_102_103

763 fragments

## Motif #3 (76 sites)

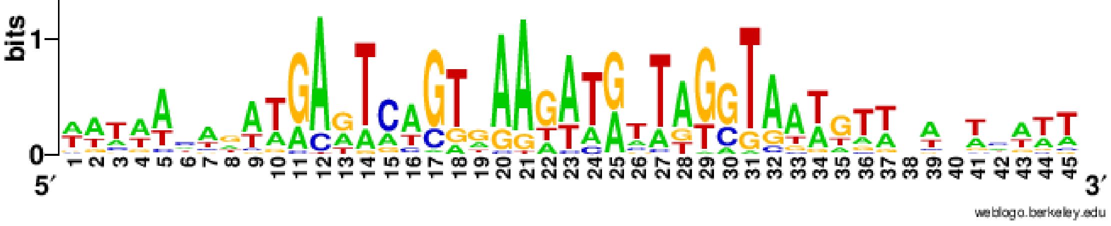


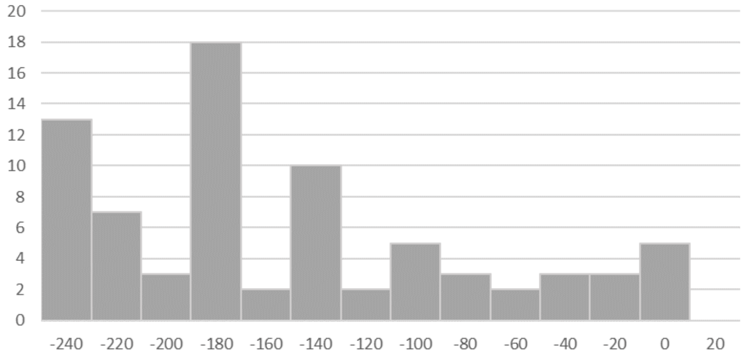


## Motif #4 (76 sites)

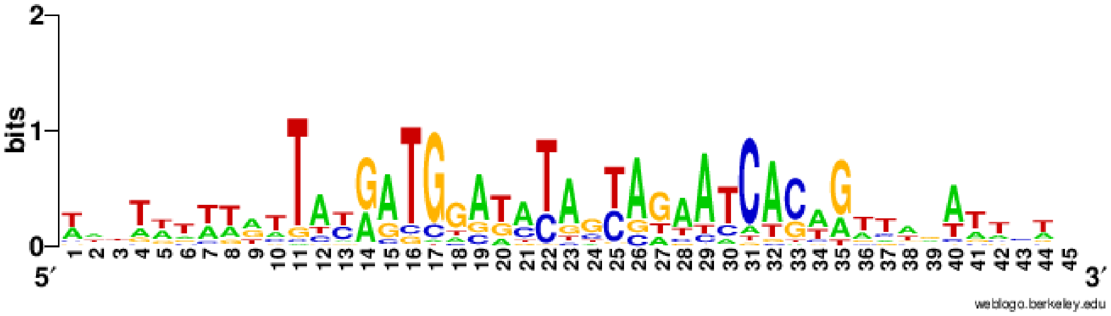


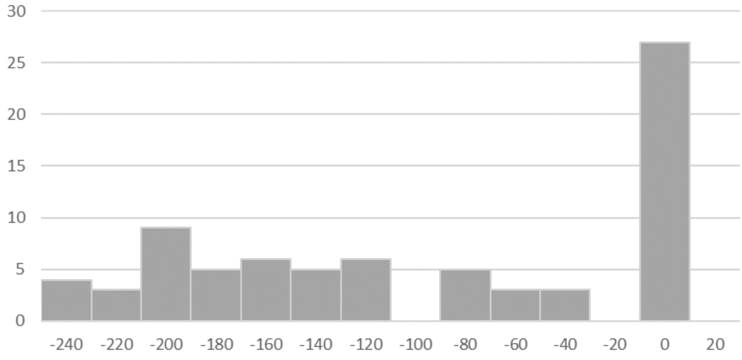

# LCPAC101_102_103

763 fragments

## Motif #5 (76 sites)

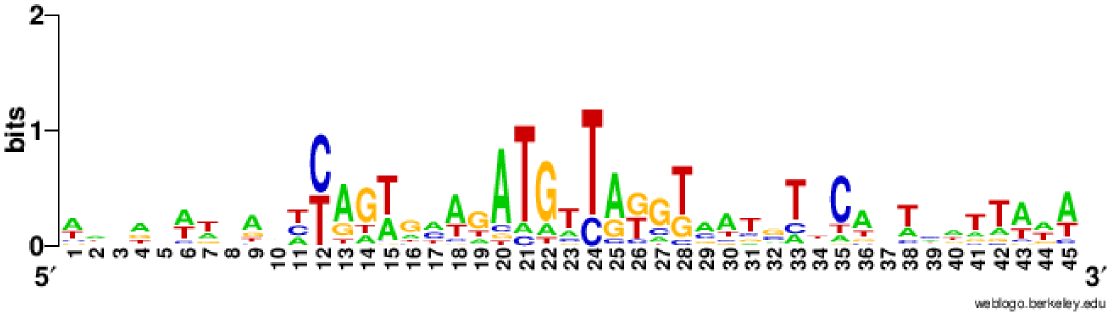


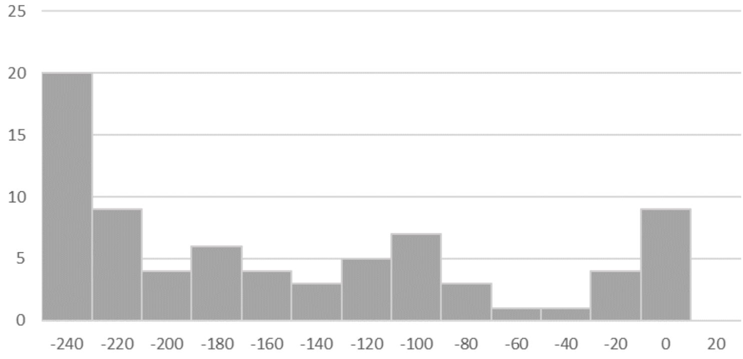


## Motif #6 (443 sites)

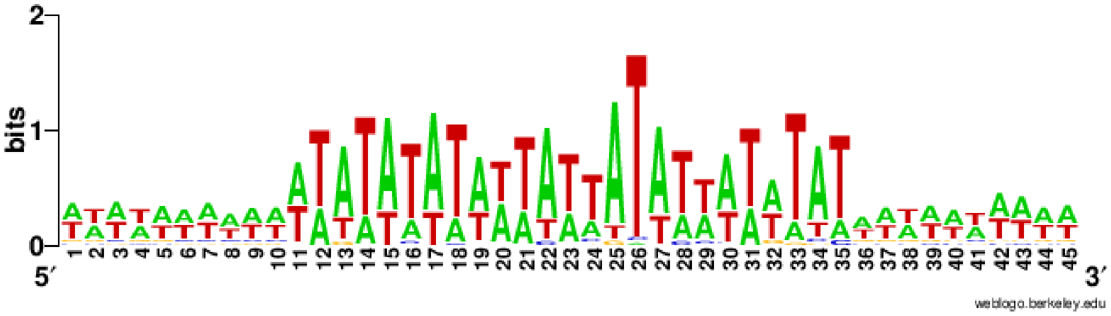


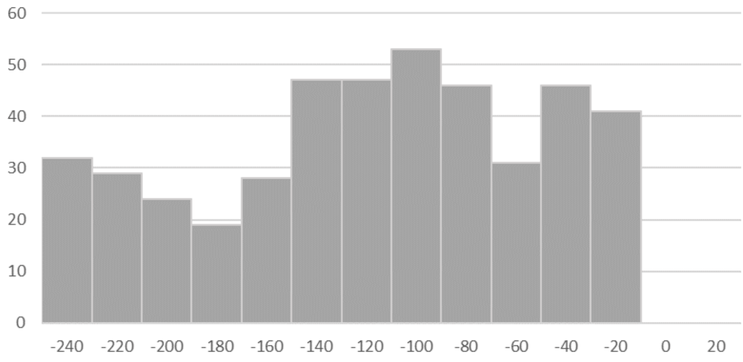

**LCPAC103**

177 fragments

## Motif #1 (50 sites)

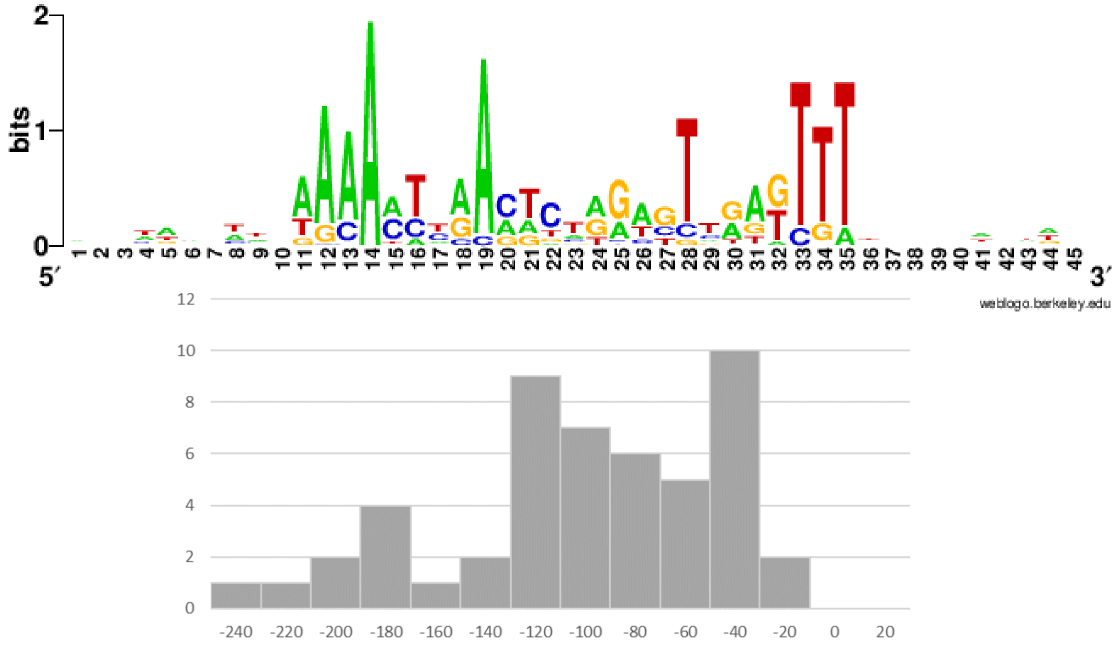


## Motif #2 (61 sites)

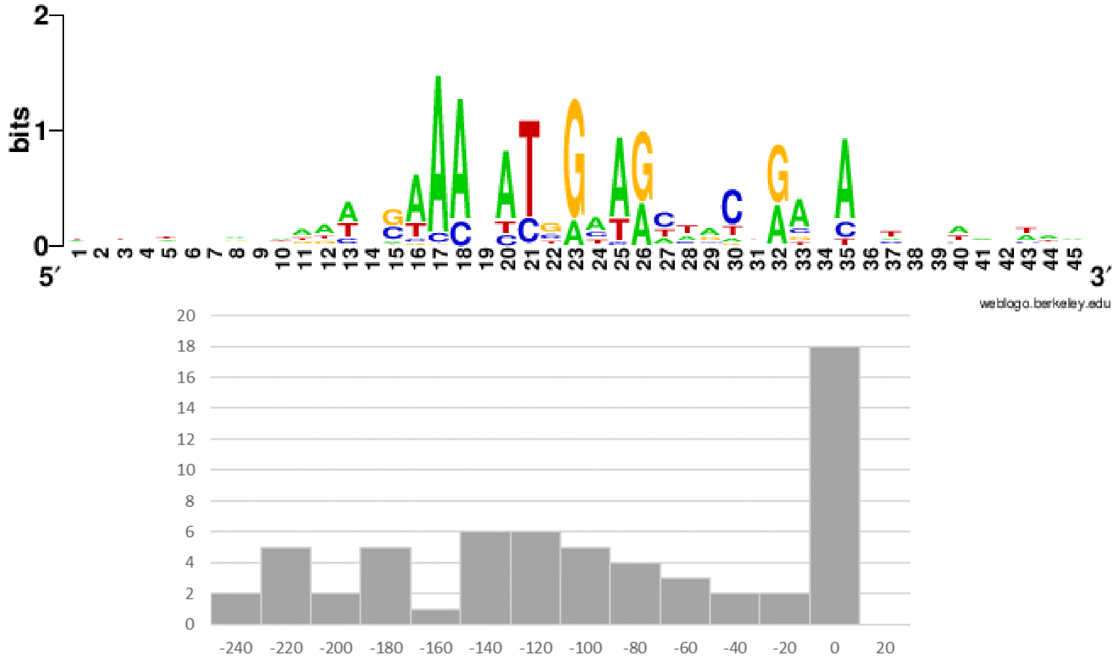

**LCPAC103**

177 fragments

Motif #3 (59 sites)

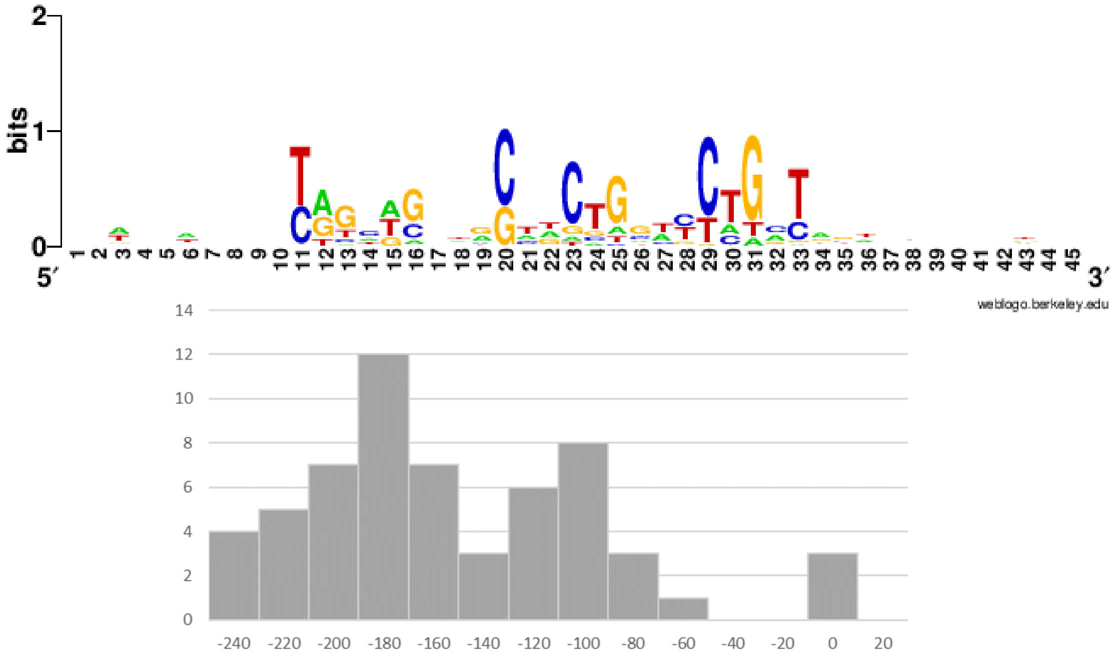


Motif #4 (62 sites)

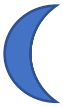

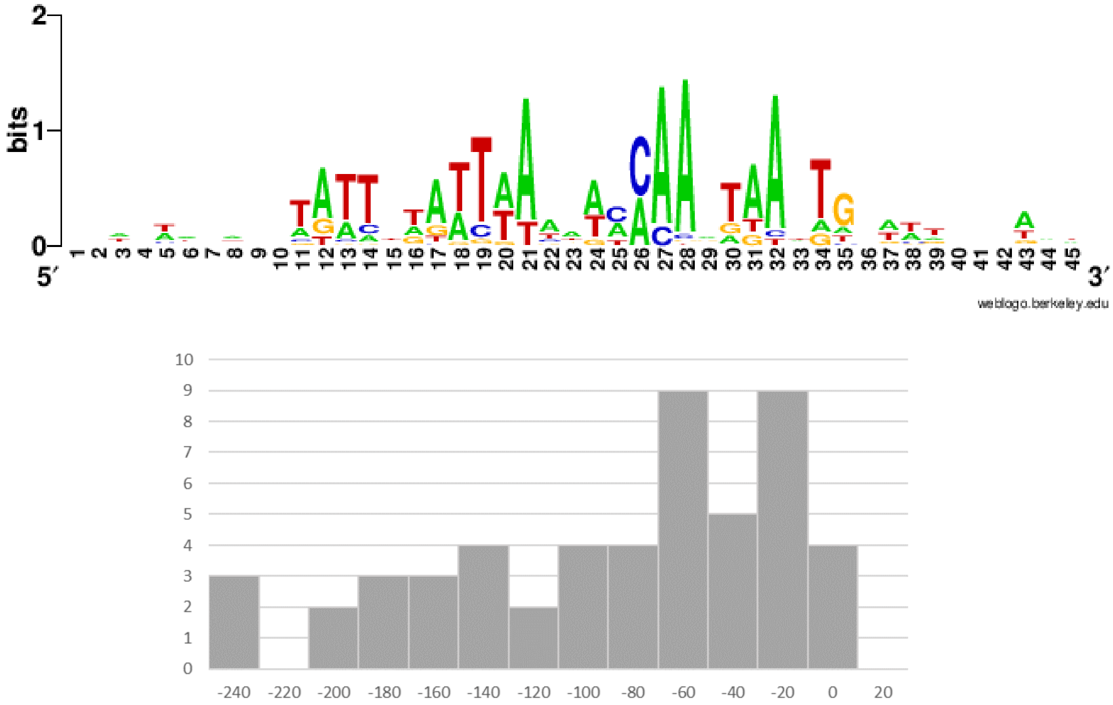

# LCPAC103

177 fragments

Motif #5 (52 sites)

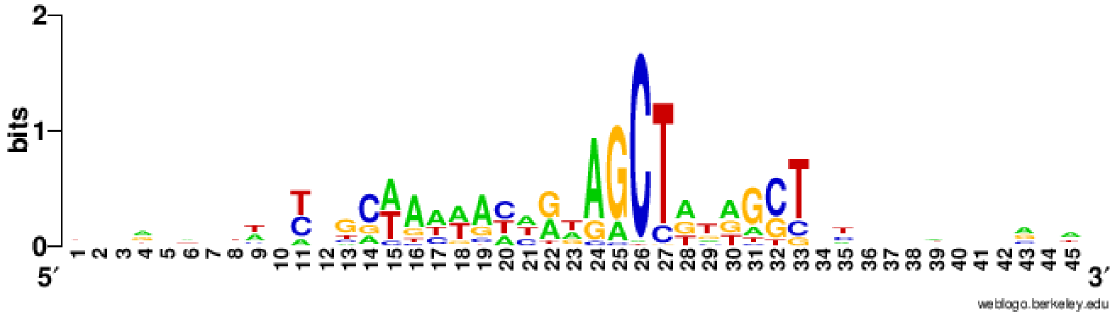


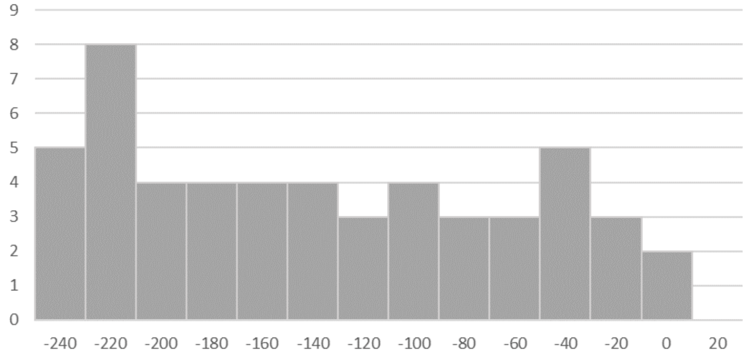

**LCPAC101_102**

586 fragments

Motif #1   (168 sites)

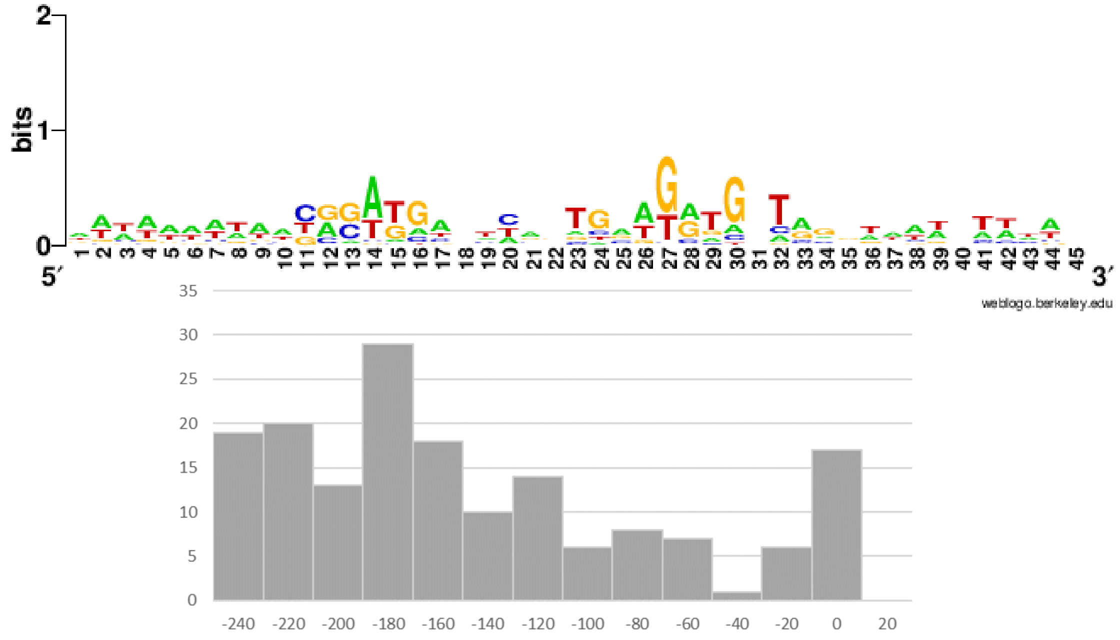


Motif #2   (574 sites)

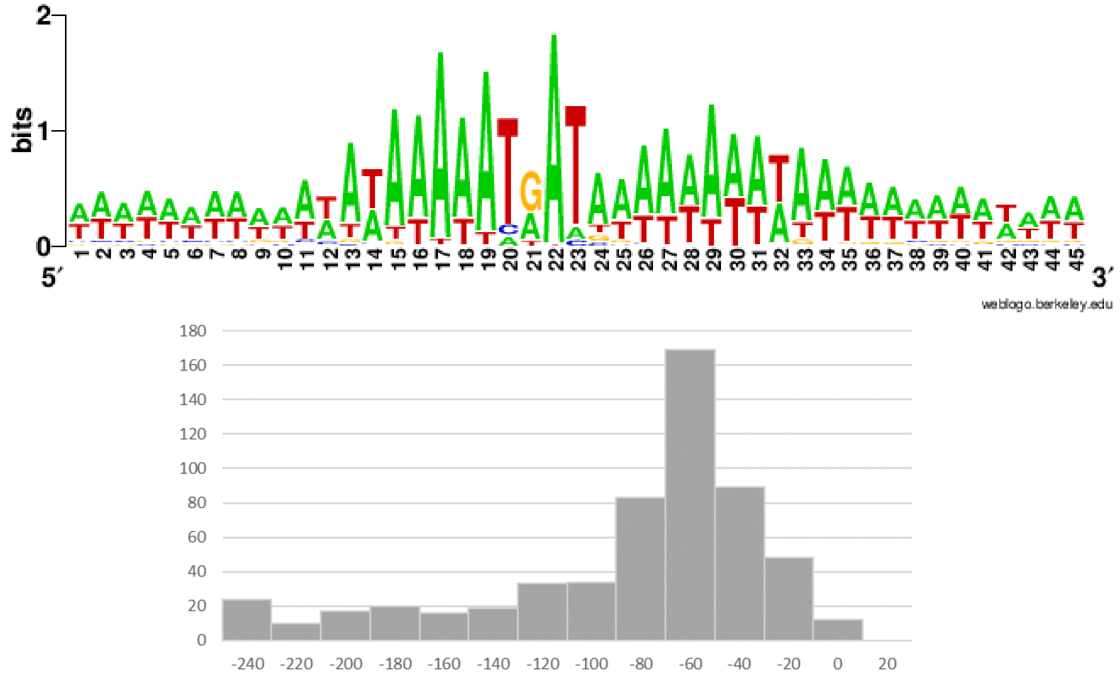

# LCPAC101_102

586 fragments

Motif #3    (103 sites)

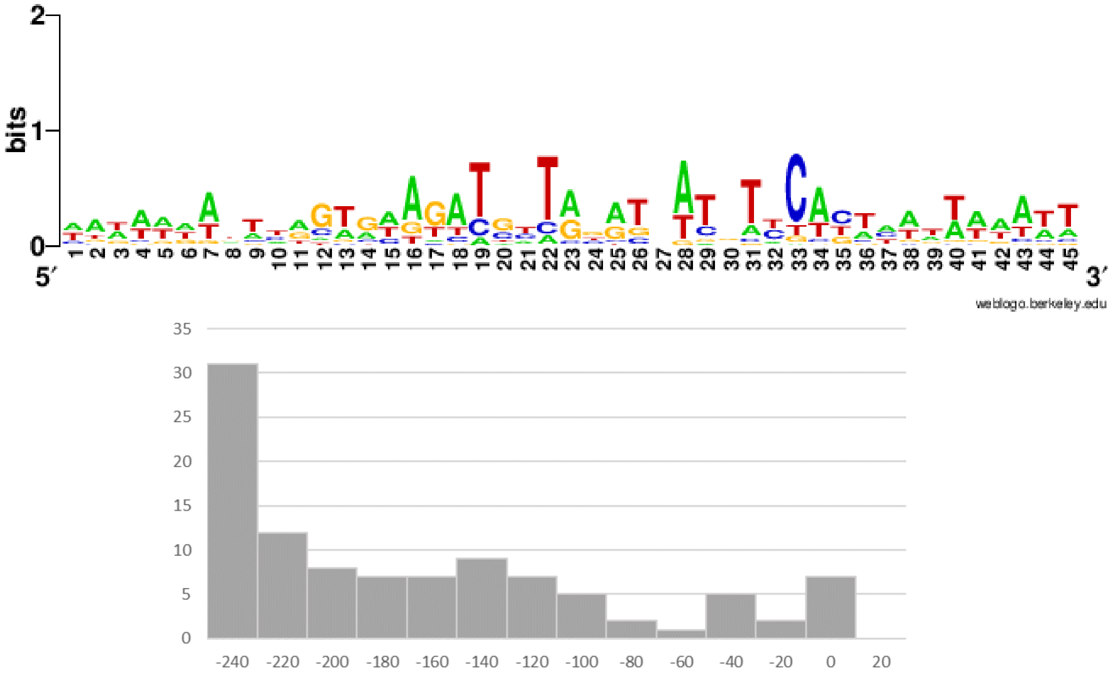


Motif #4    (101 sites)

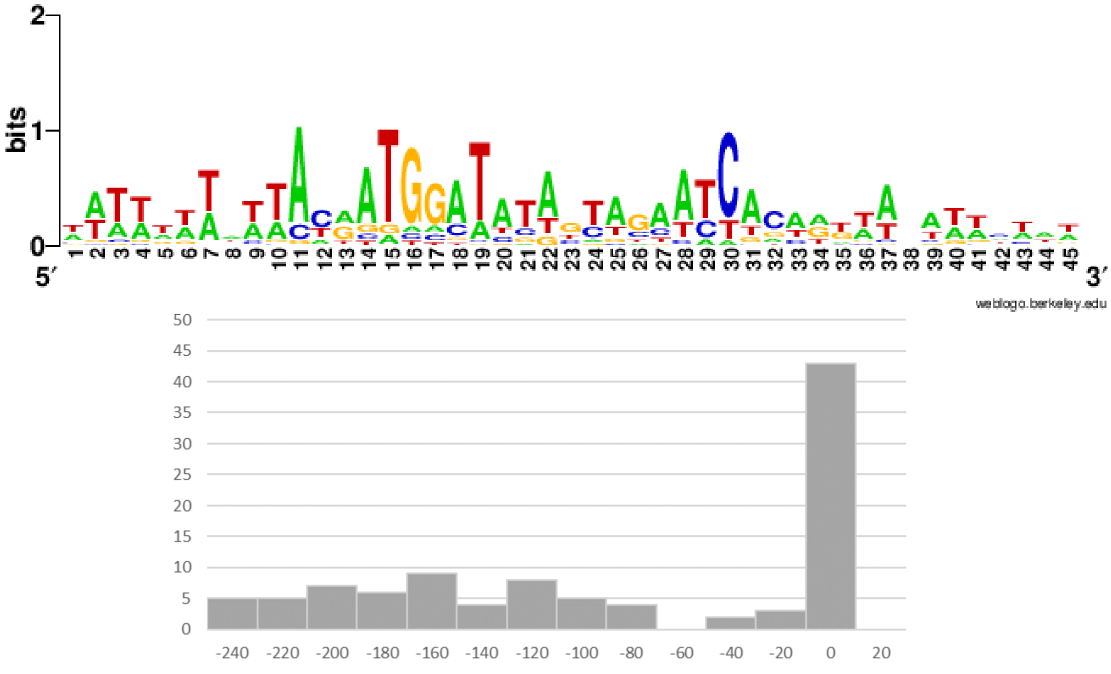

# LCPAC101_102

586 fragments

## Motif #5 (374 sites)

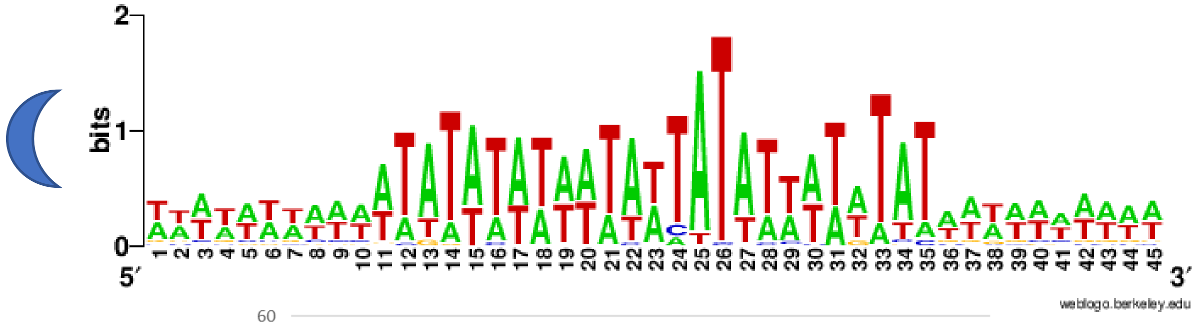


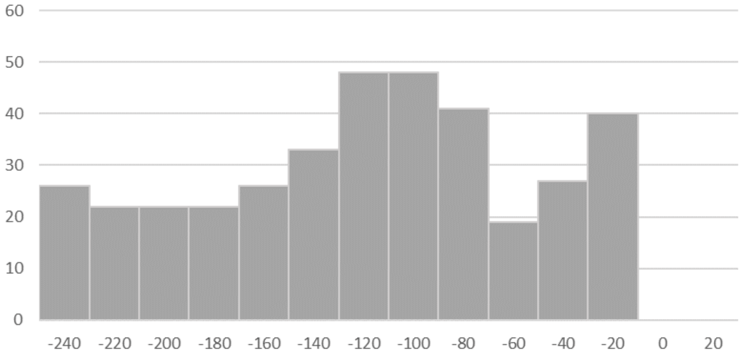


## Motif #9 (101 sites)

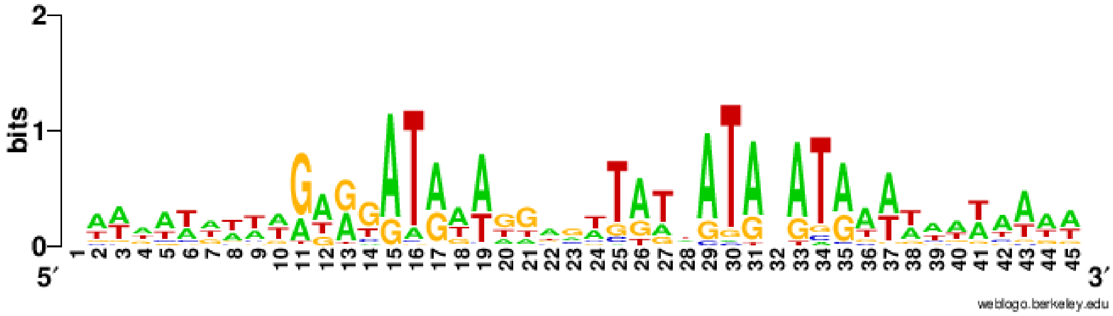


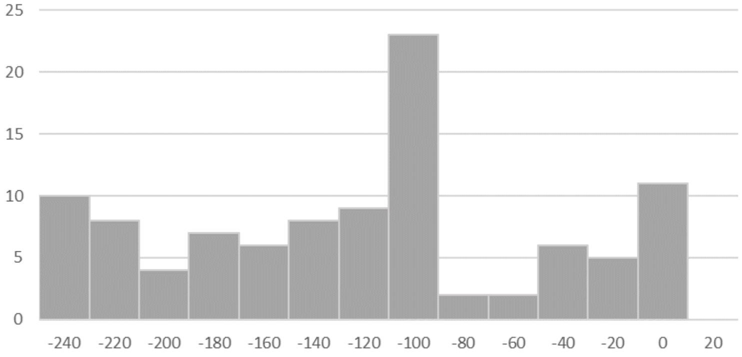

**LCPAC302_304**

949 fragments

## Motif #1   (211 sites)

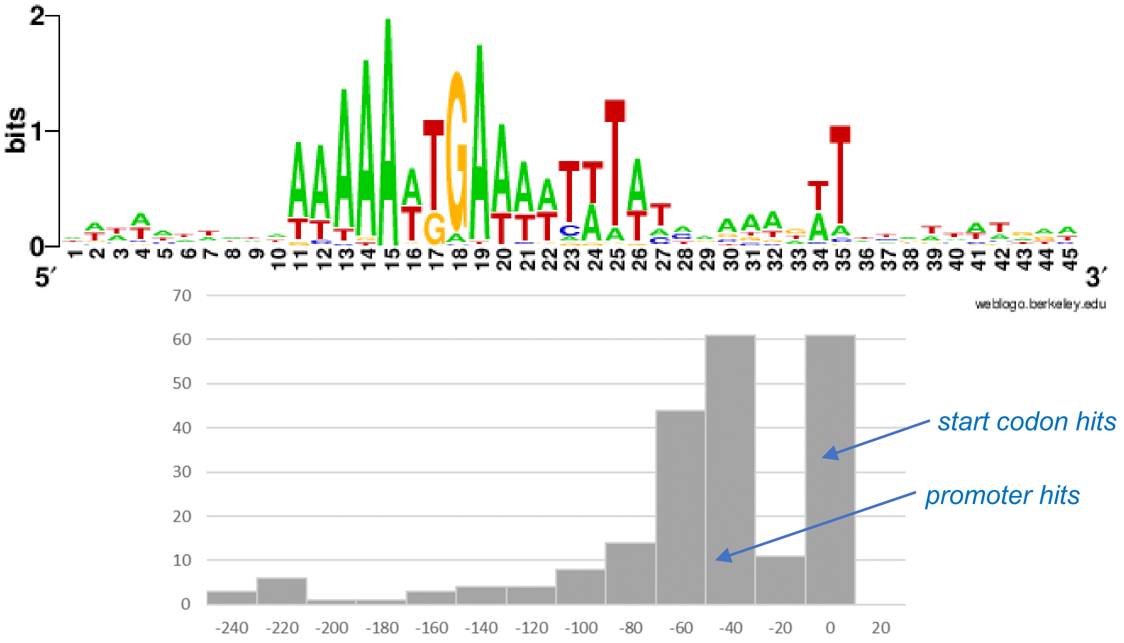


## Motif #2   (110 sites)

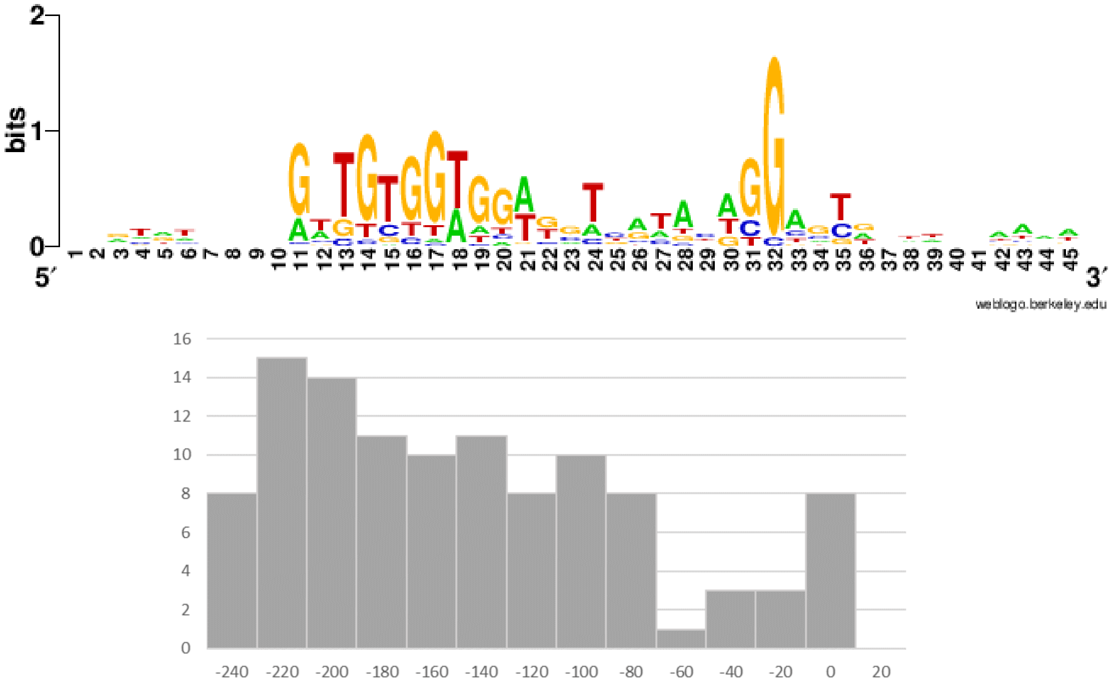

# LCPAC302_304

949 fragments

## Motif #3 (263 sites)

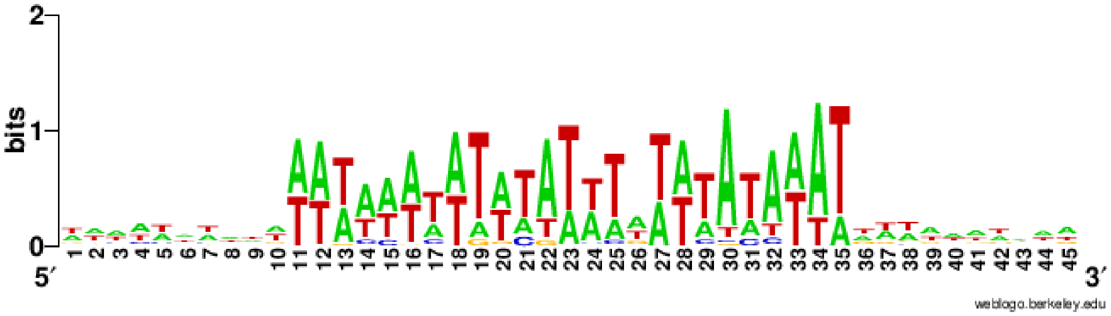


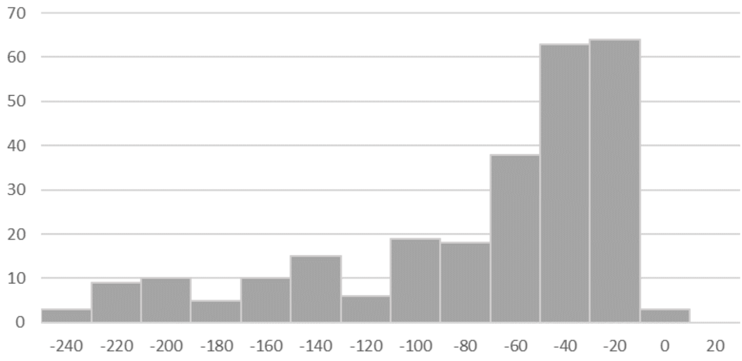


## Motif #4 (107 sites)

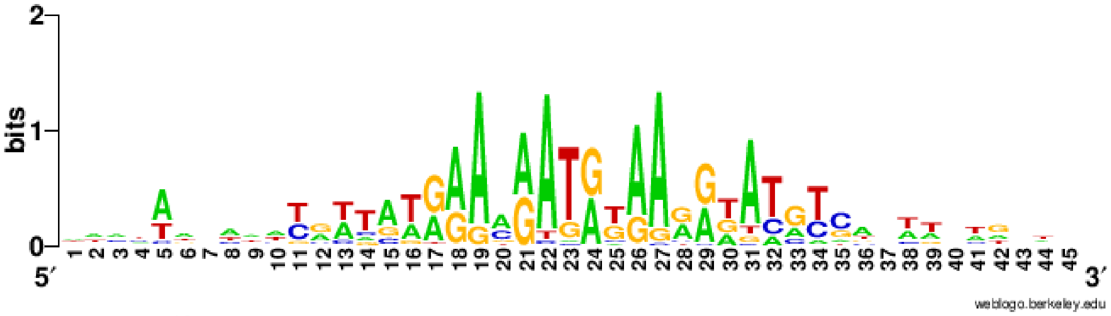


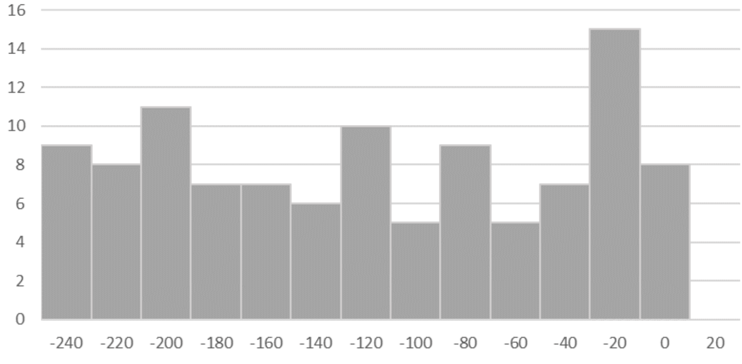

# LCPAC302_304

949 fragments

## Motif #5 (104 sites)

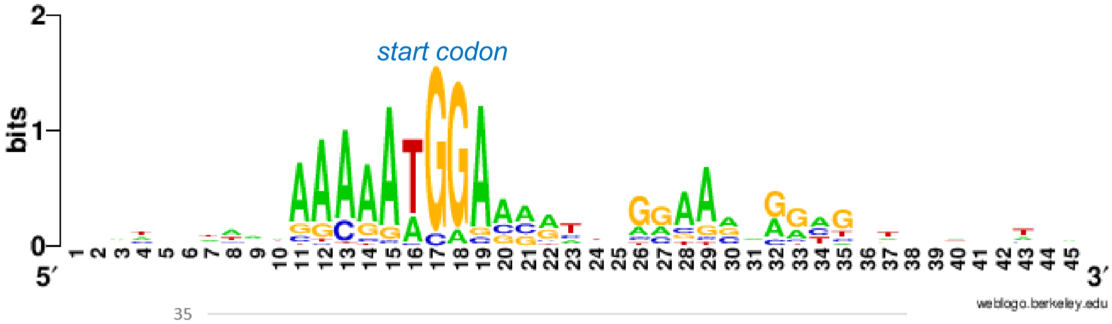


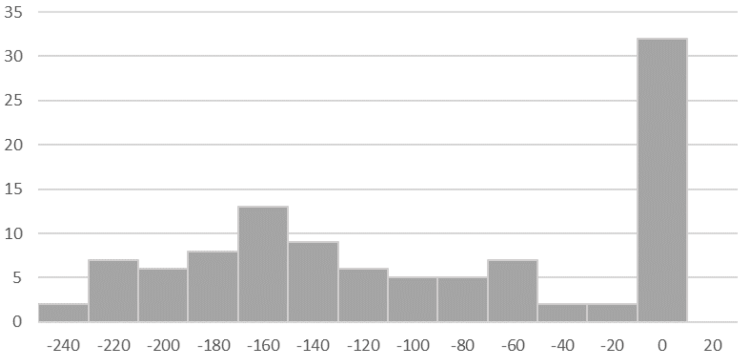


## Motif #6 (100 sites)

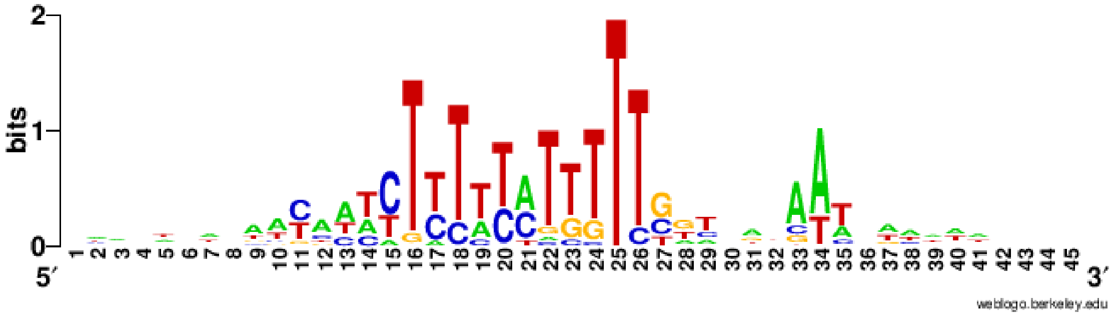


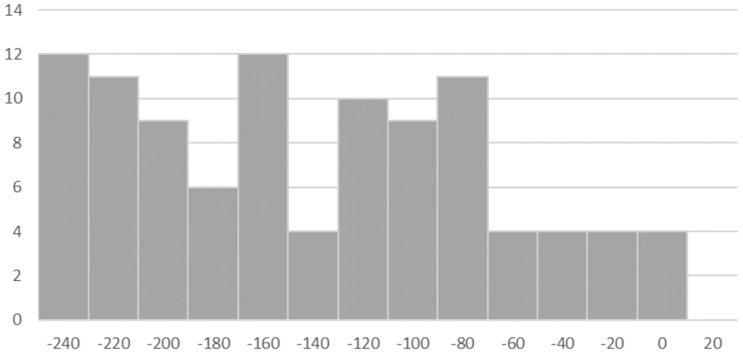

# LCPAC001

220 fragments

## Motif #1 (122 sites)

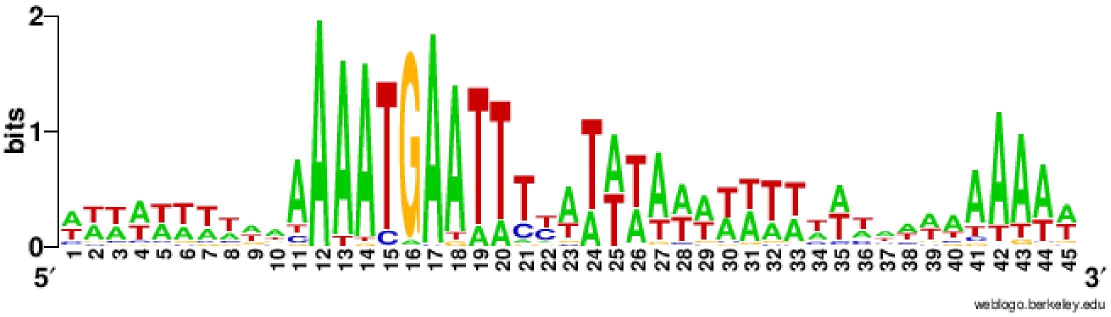


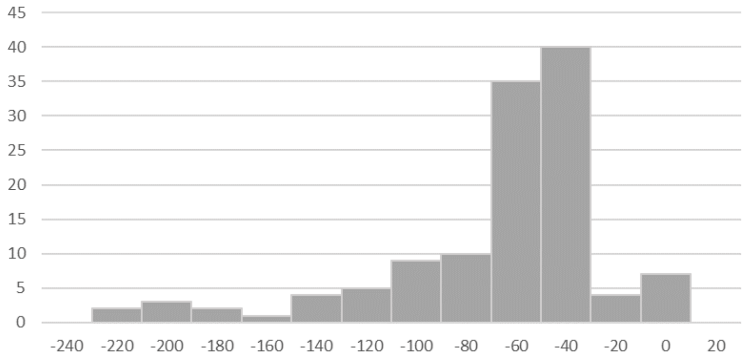


## Motif #2 (61 sites)

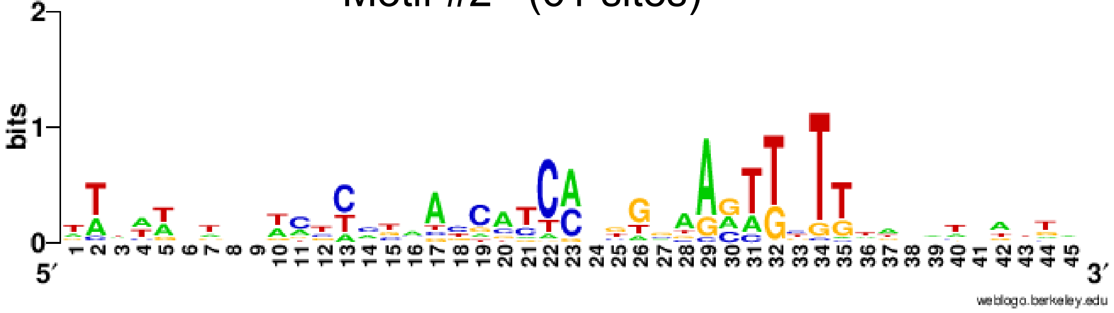


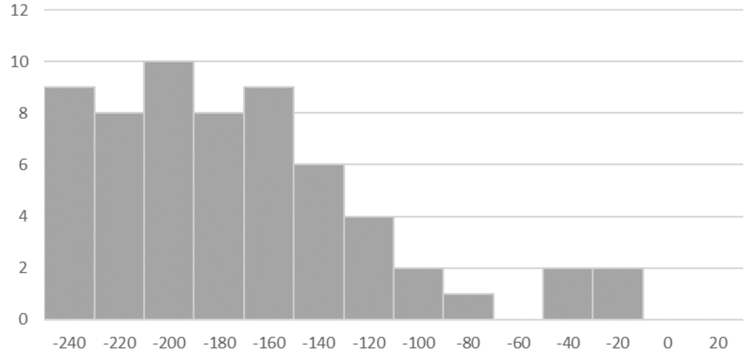

**LCPAC001**

220 fragments

Motif #3 (50 sites)

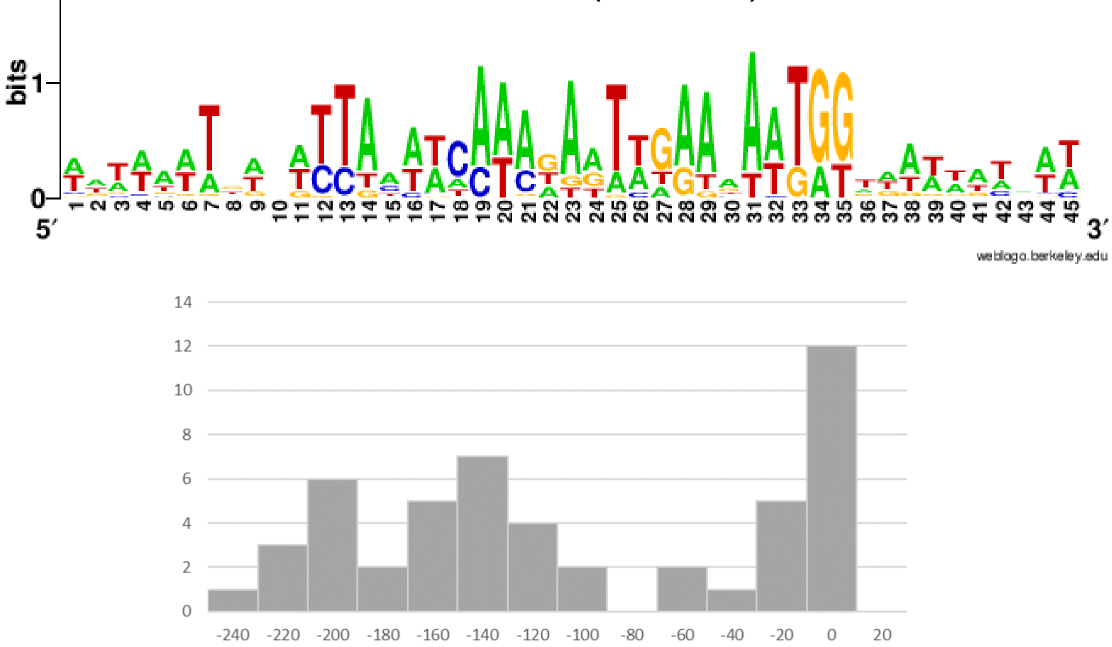


Motif #4 (64 sites)

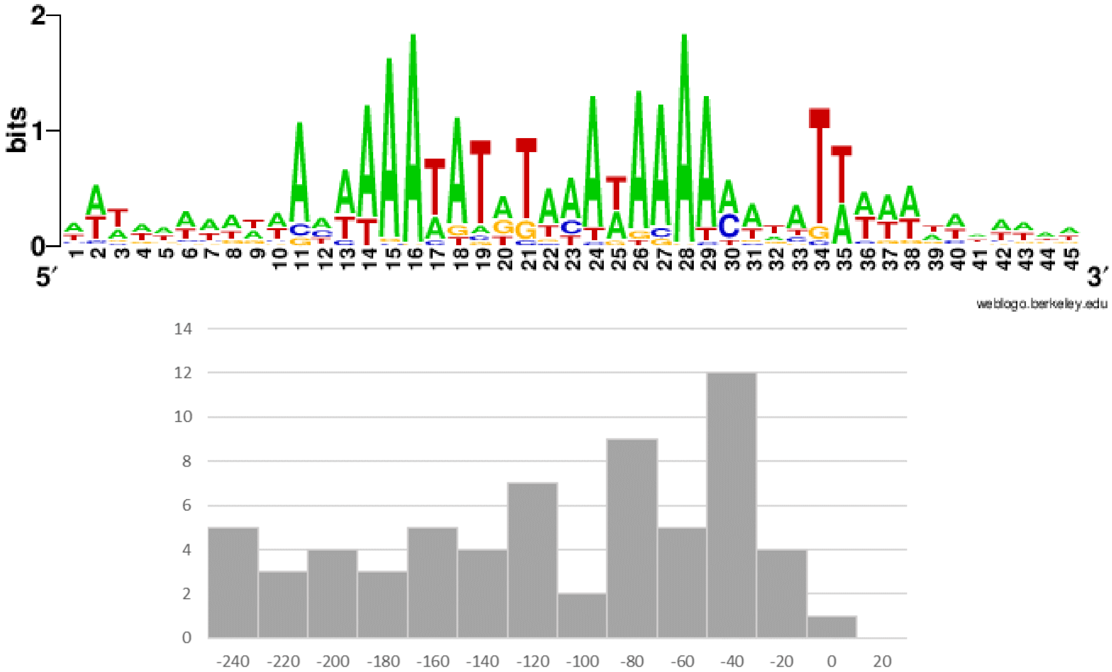

**LCPAC001**
220 fragments

Motif #5 (50 sites)

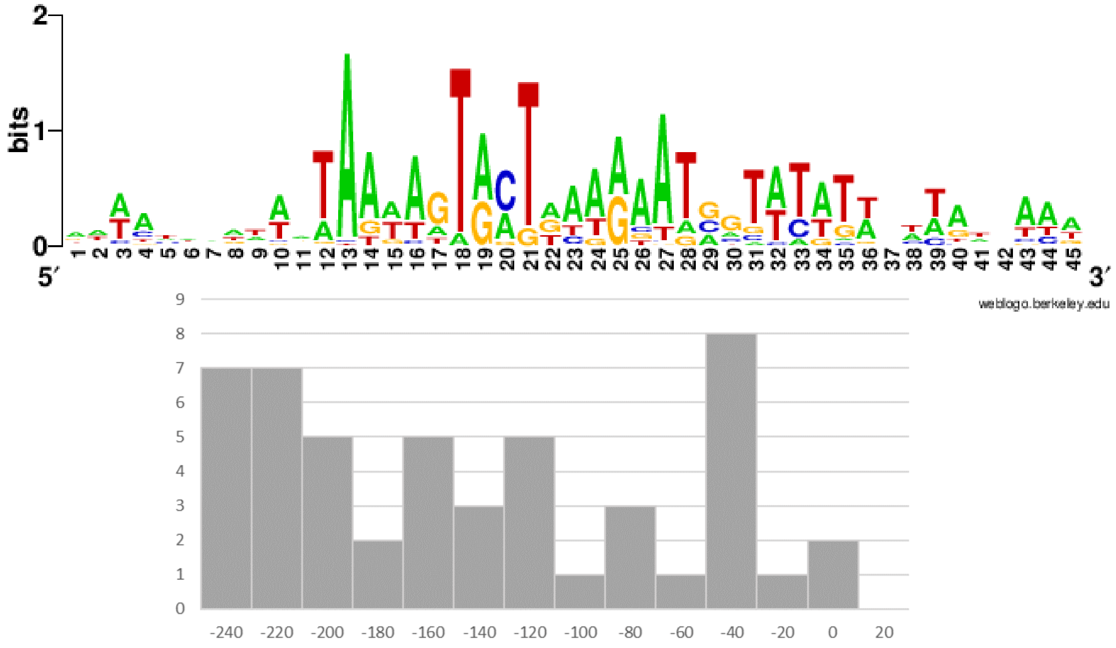


Motif #7 (58 sites)

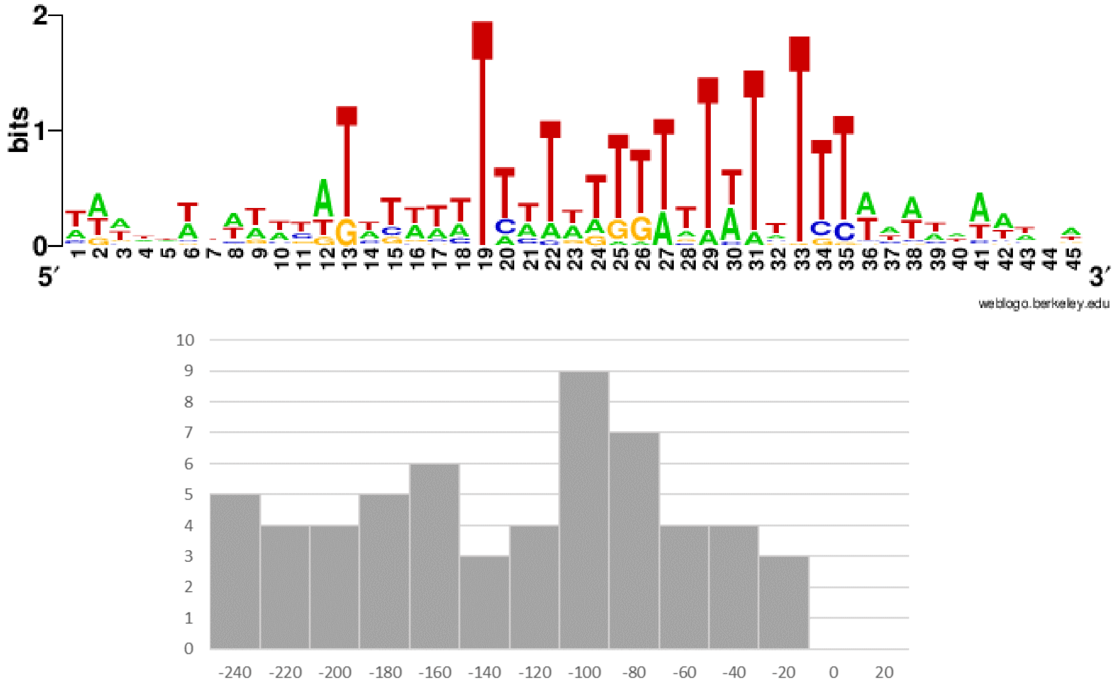

**Pithovirus sibericum**

467 fragments

## Motif #1 (99 sites)

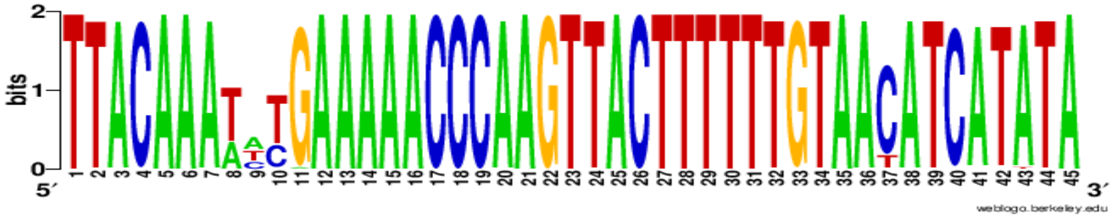


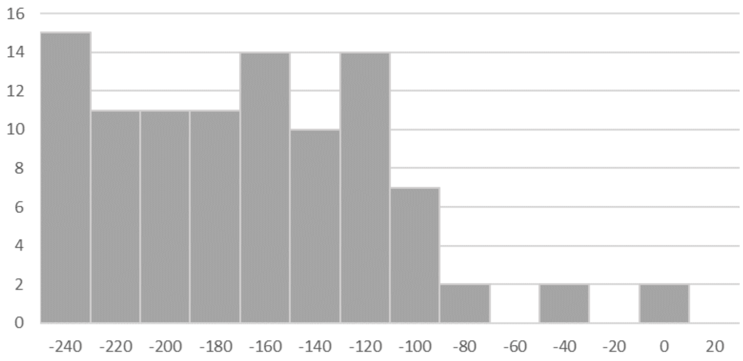


## Motif #2 (99 sites)

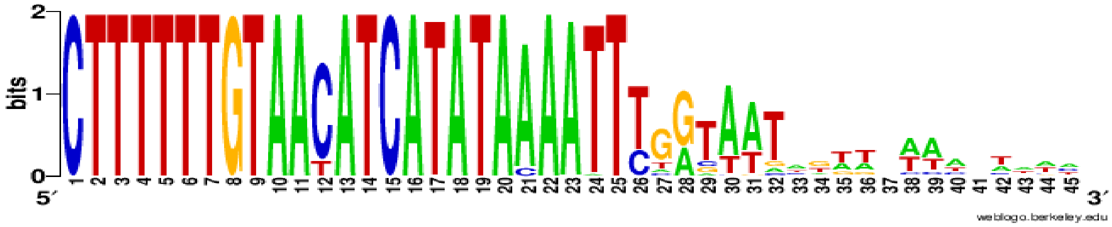


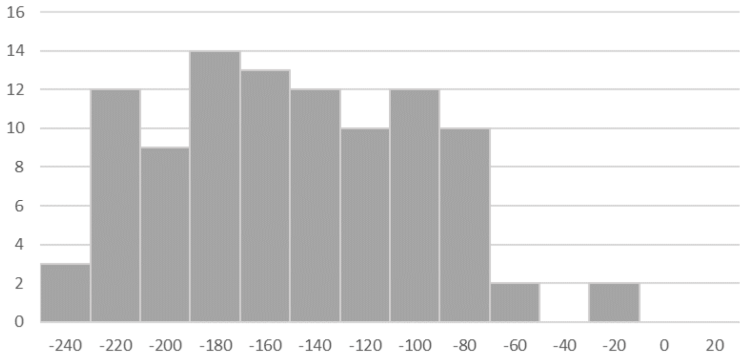

# Pithovirus sibericum

467 fragments

## Motif #3 (60 sites)

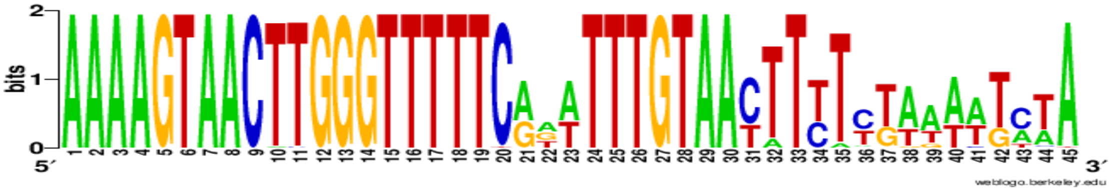


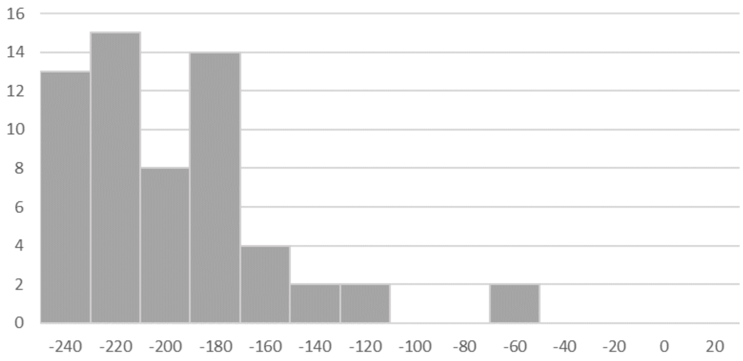


## Motif #4 (72 sites)

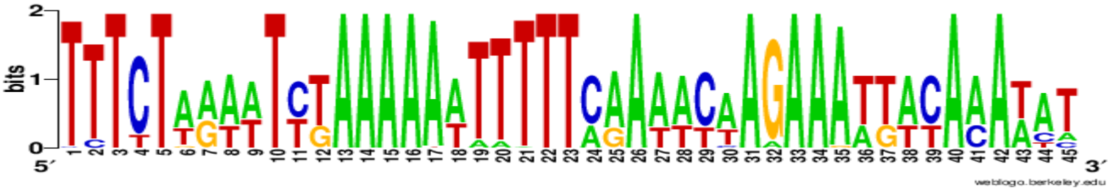


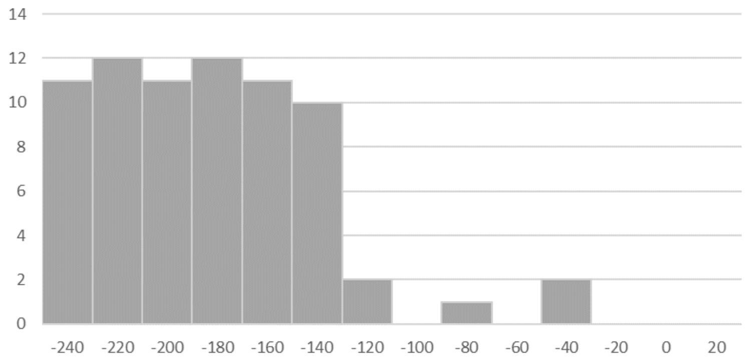

**Pithovirus sibericum**

467 fragments

Motif #5 (50 sites)

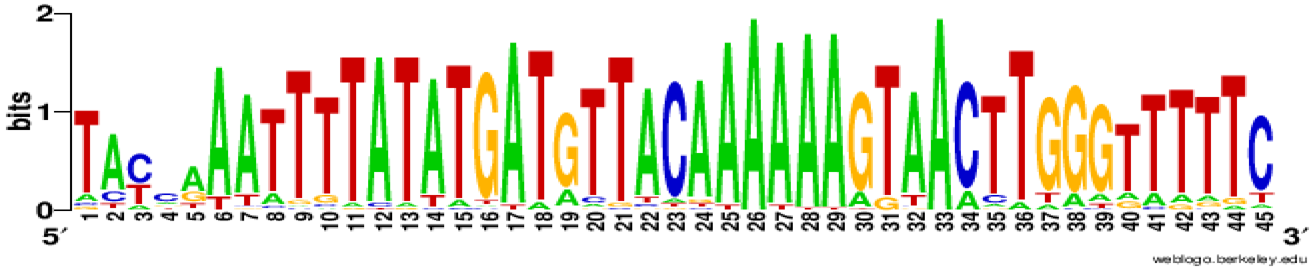


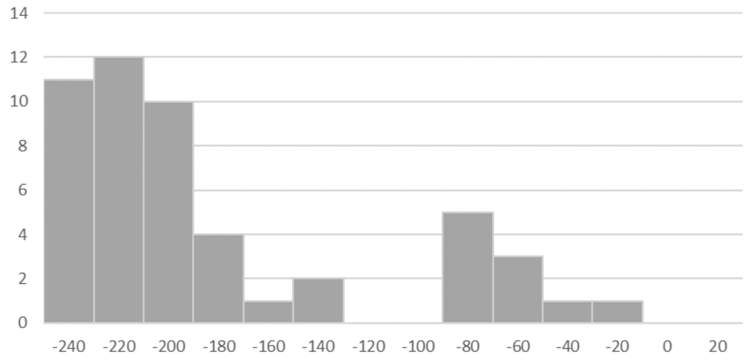


Motif #6 (443 sites)

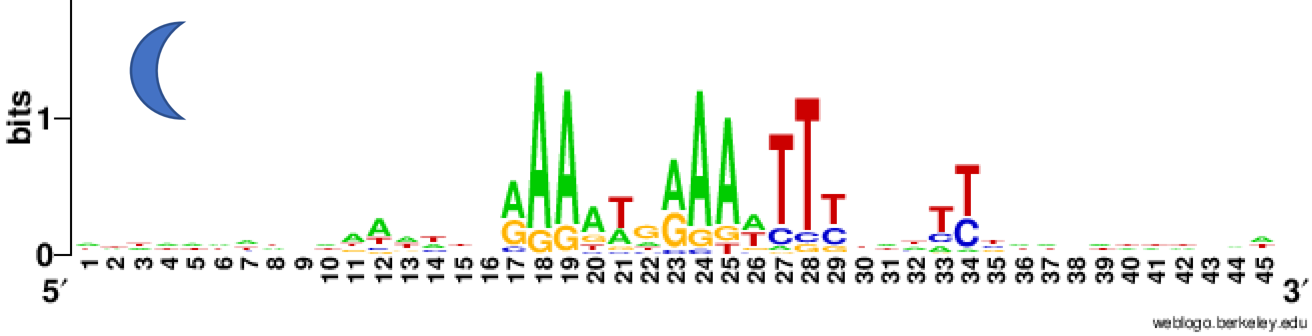


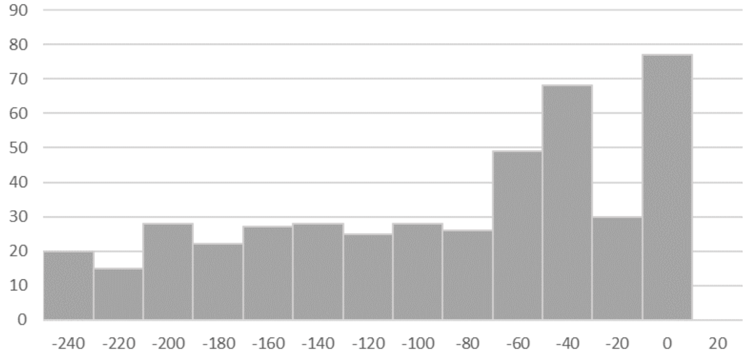

# Pithovirus sibericum

467 fragments

## Motif #7 (50 sites)

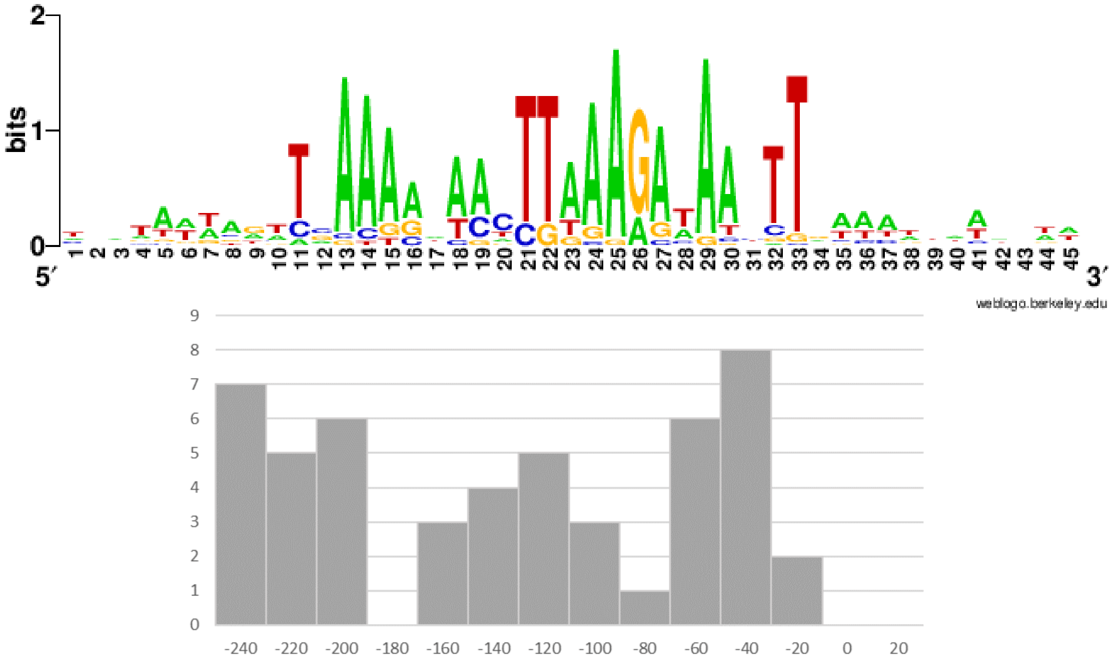


## Motif #8 (113 sites)

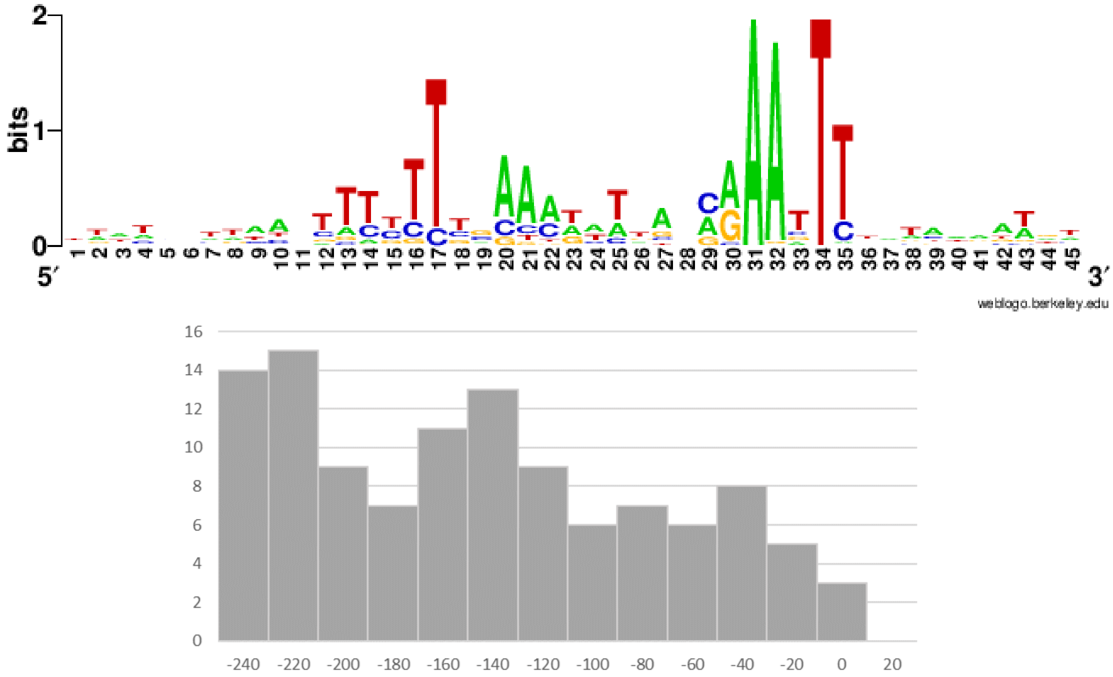

# Pithovirus sibericum

467 fragments

## Motif #9 (68 sites)

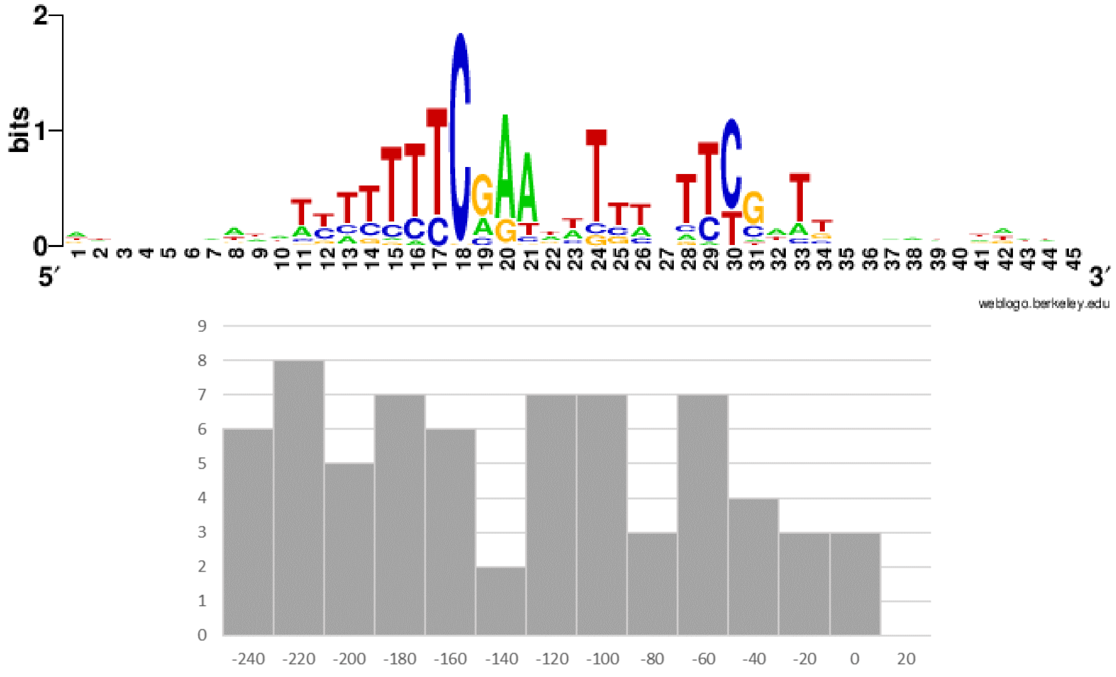


## Motif #10 (78 sites)

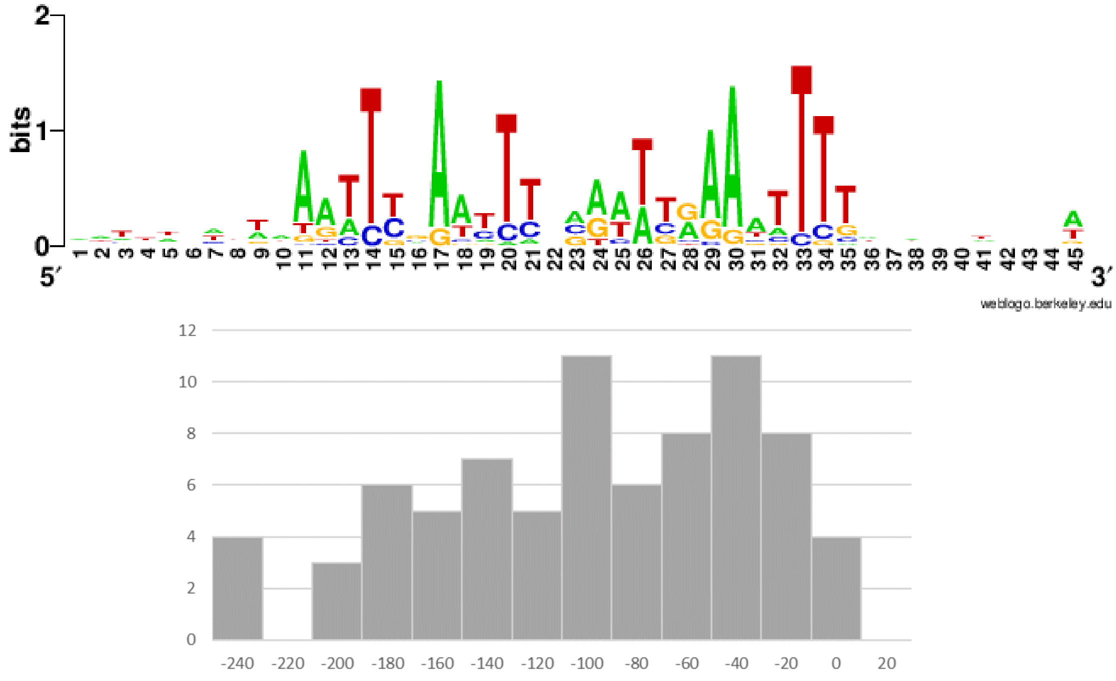

**Cedratvirus A11**

574 fragments

Motif #1 (50 sites)

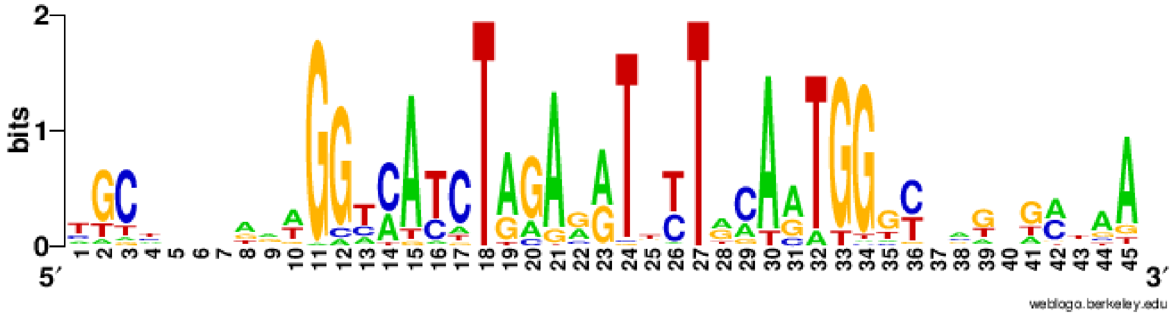


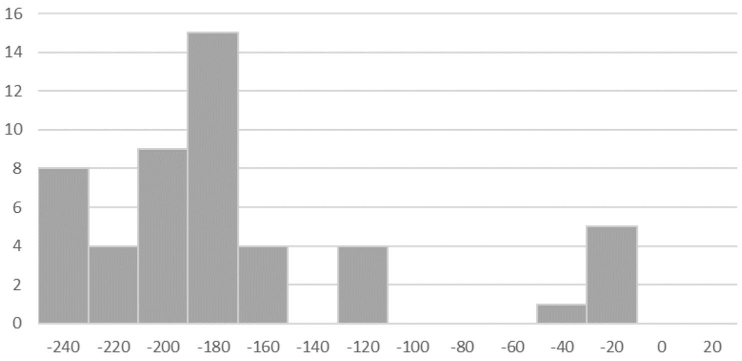


Motif #2 (50 sites)

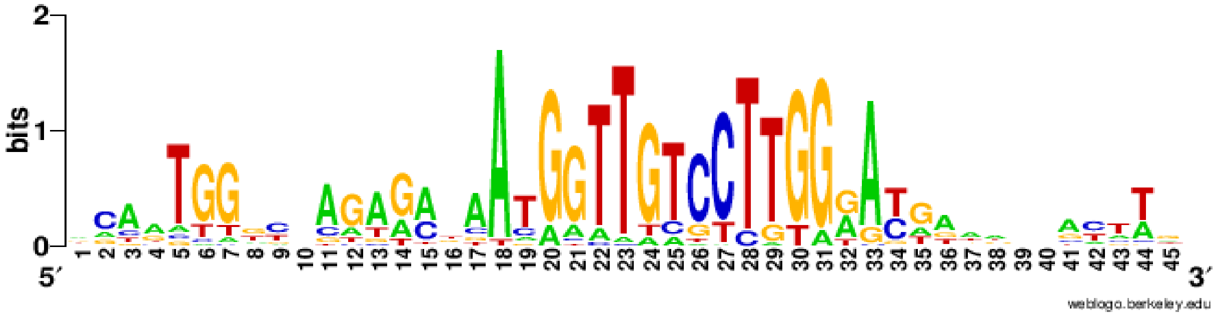


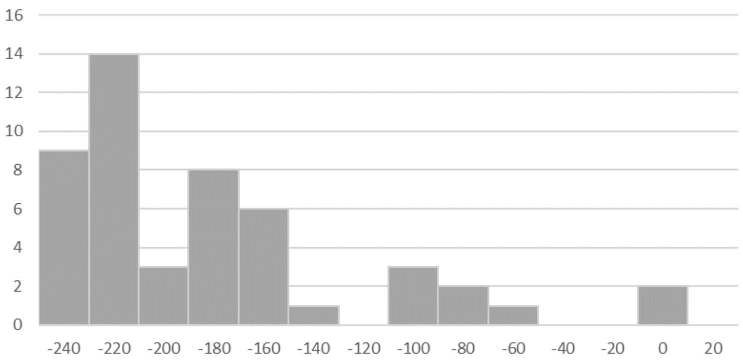

# Cedratvirus A11

574 fragments

## Motif #3 (42 sites)

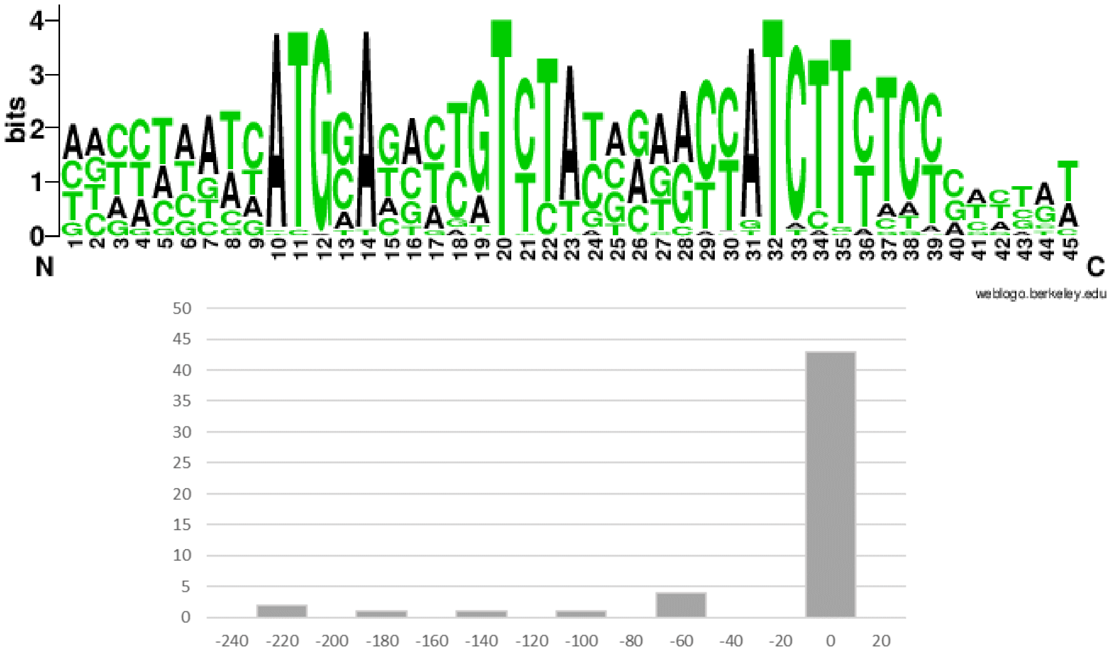


## Motif #4 (148 sites)

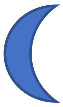

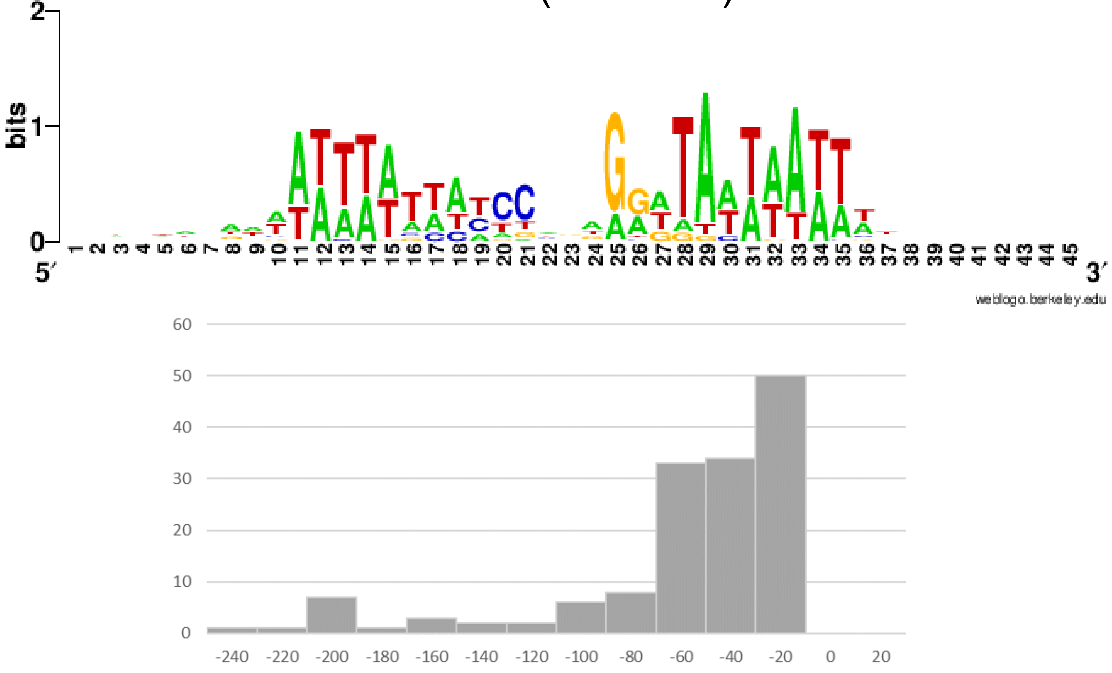

# Cedratvirus A11

574 fragments

Motif #5  (270 sites)

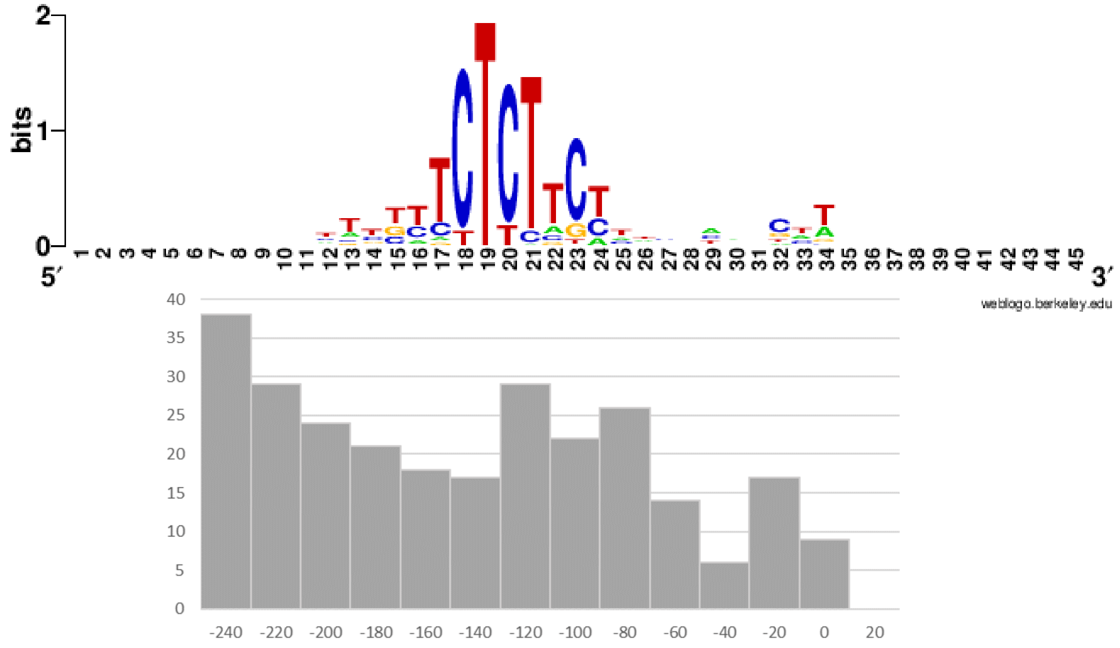


Motif #6   (163 sites)

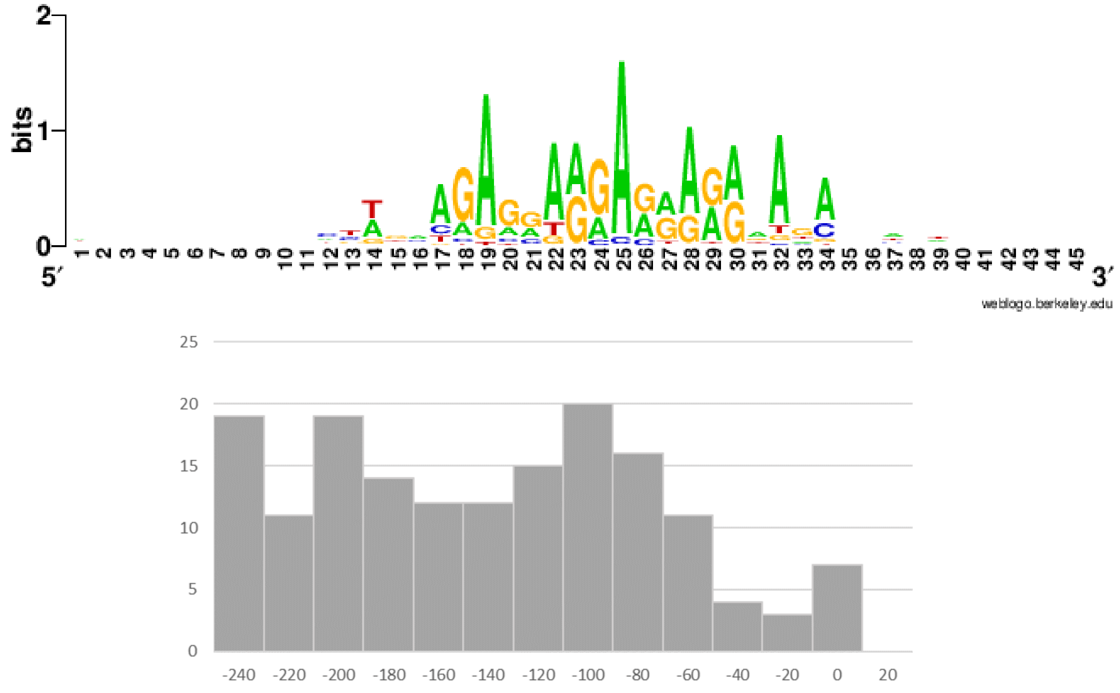

# Cedratvirus A11

574 fragments

Motif #7 (52 sites)

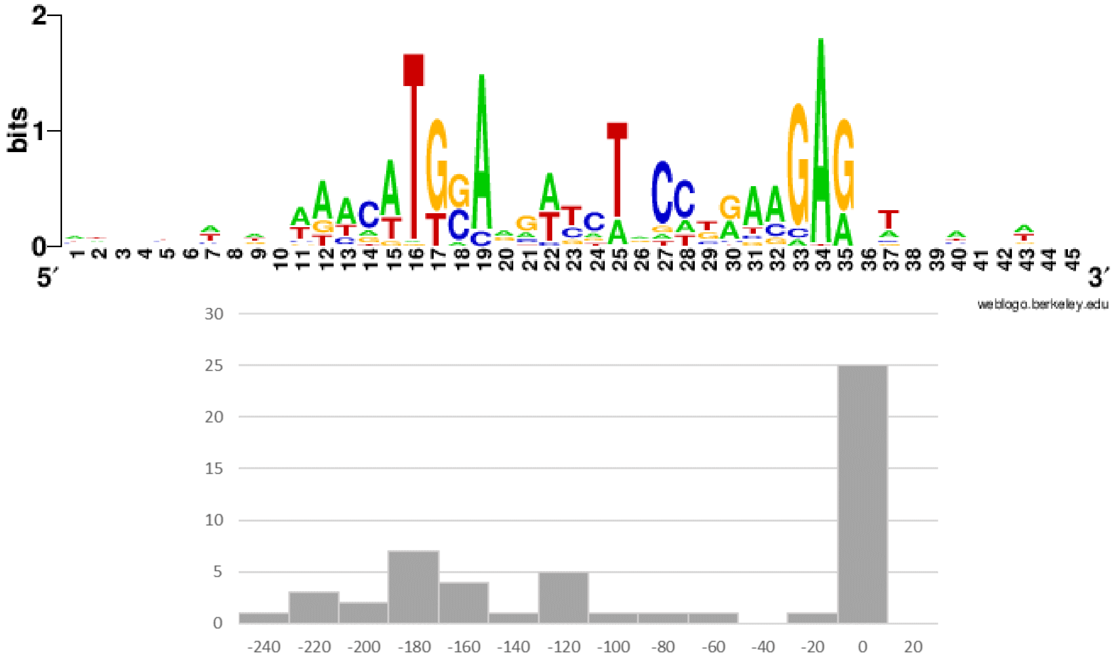


Motif #8 (52 sites)

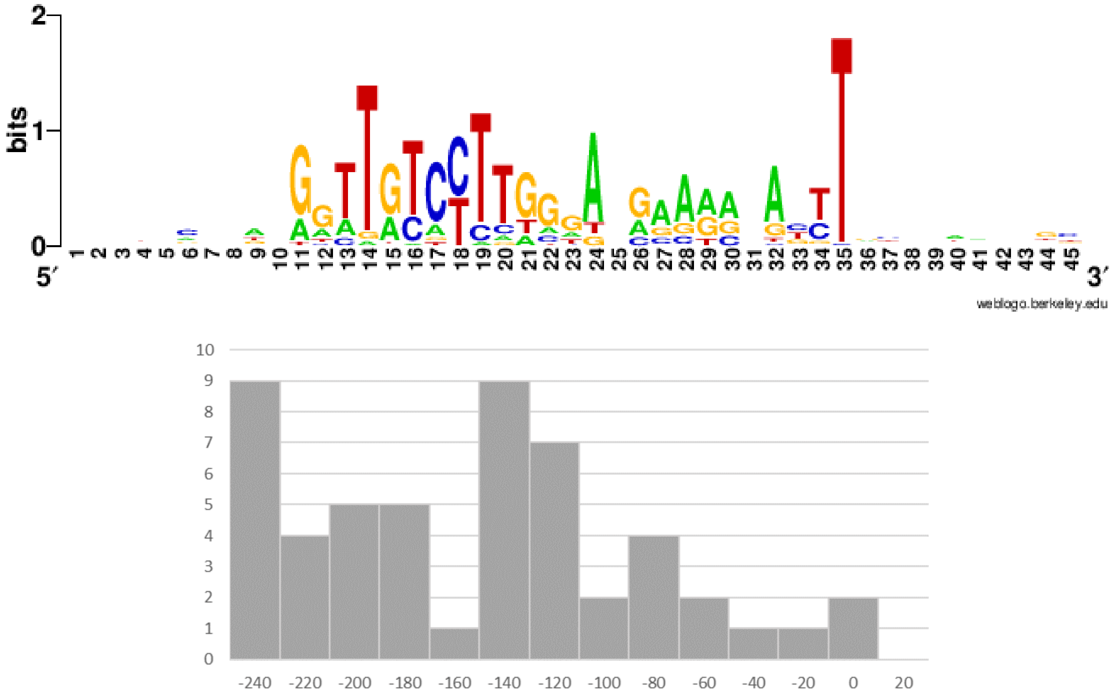

# Cedratvirus A11

574 fragments

Motif #9 (82 sites)

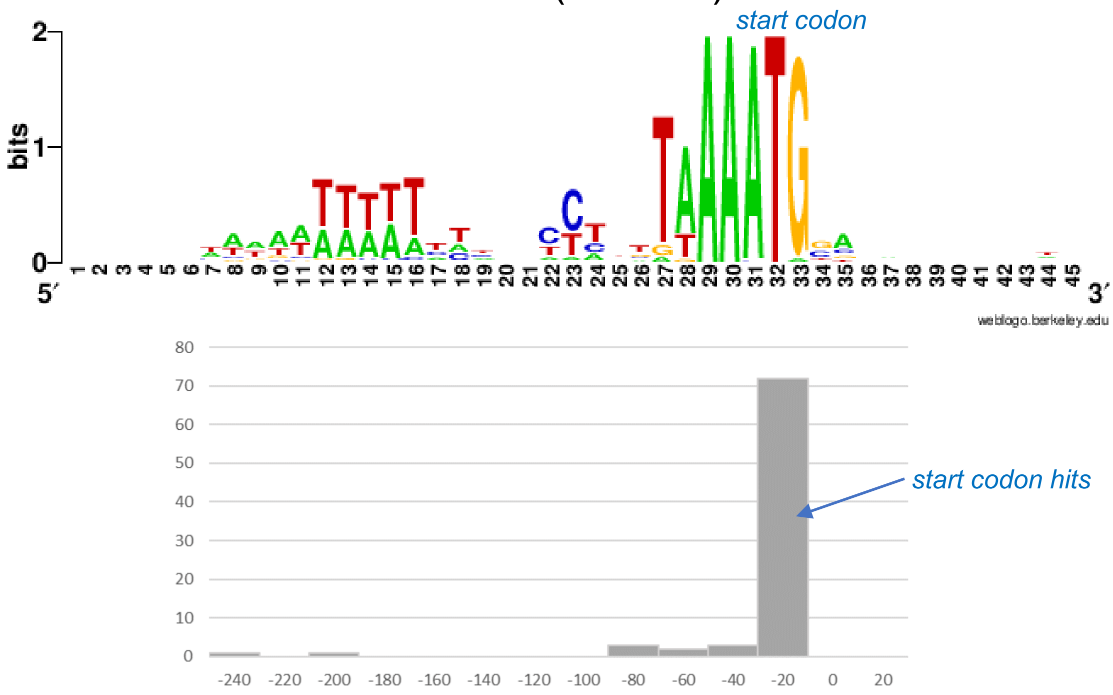


Motif #10 (50 sites)

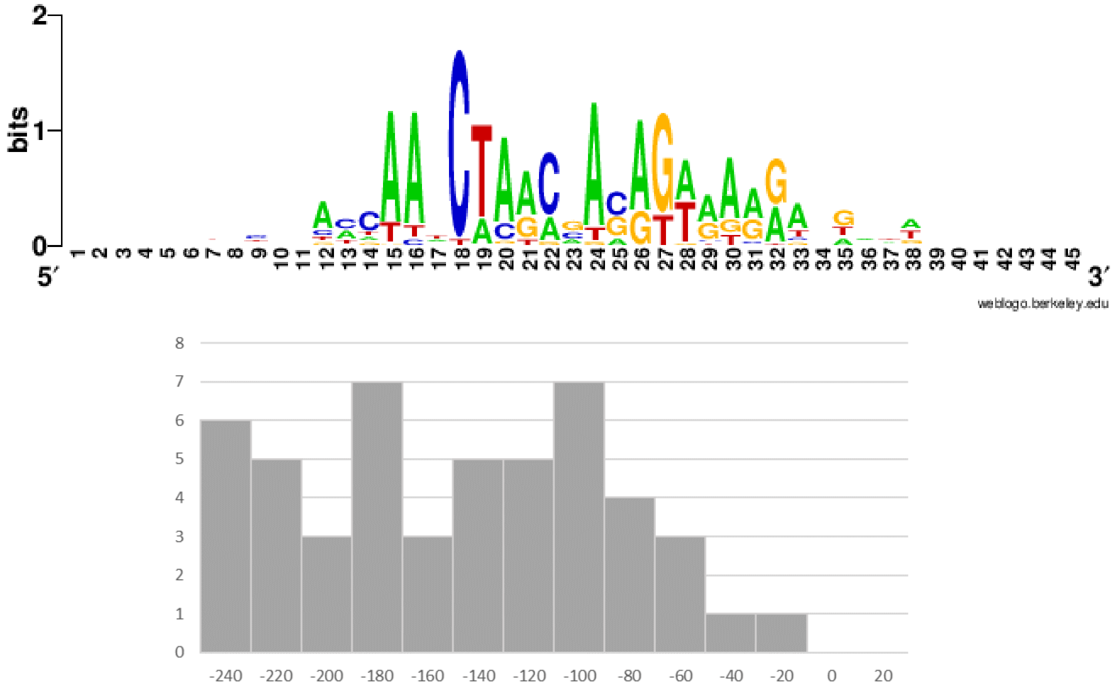

# Pithovirus and Cedratvirus

1041 fragments

## Motif #6 (254 sites)

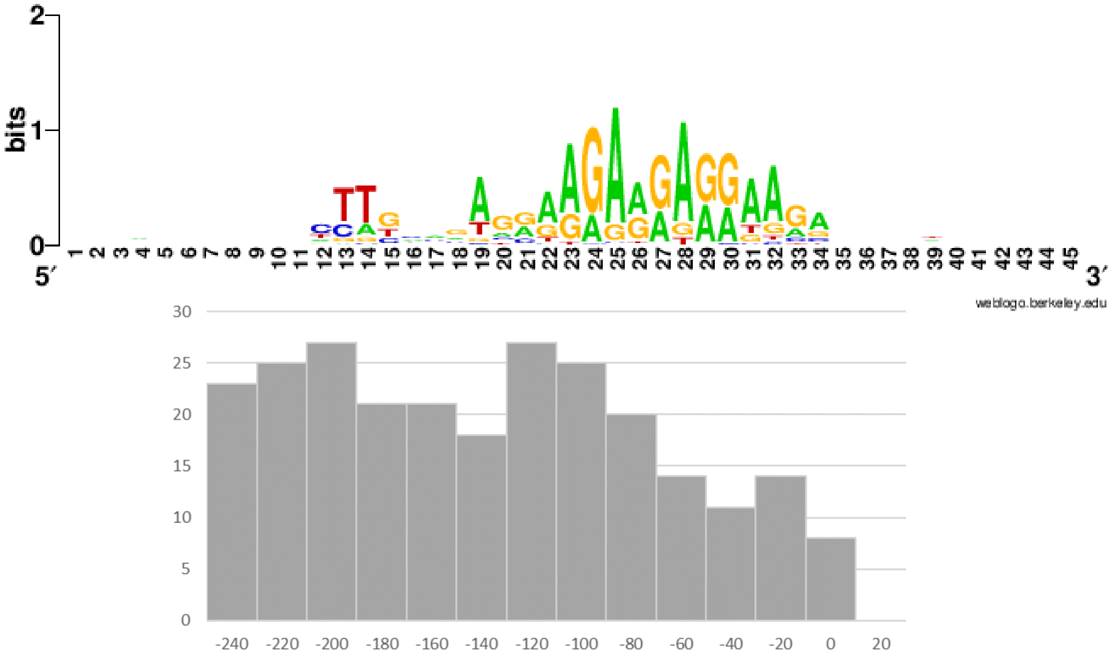


## Motif #7 (255 sites)

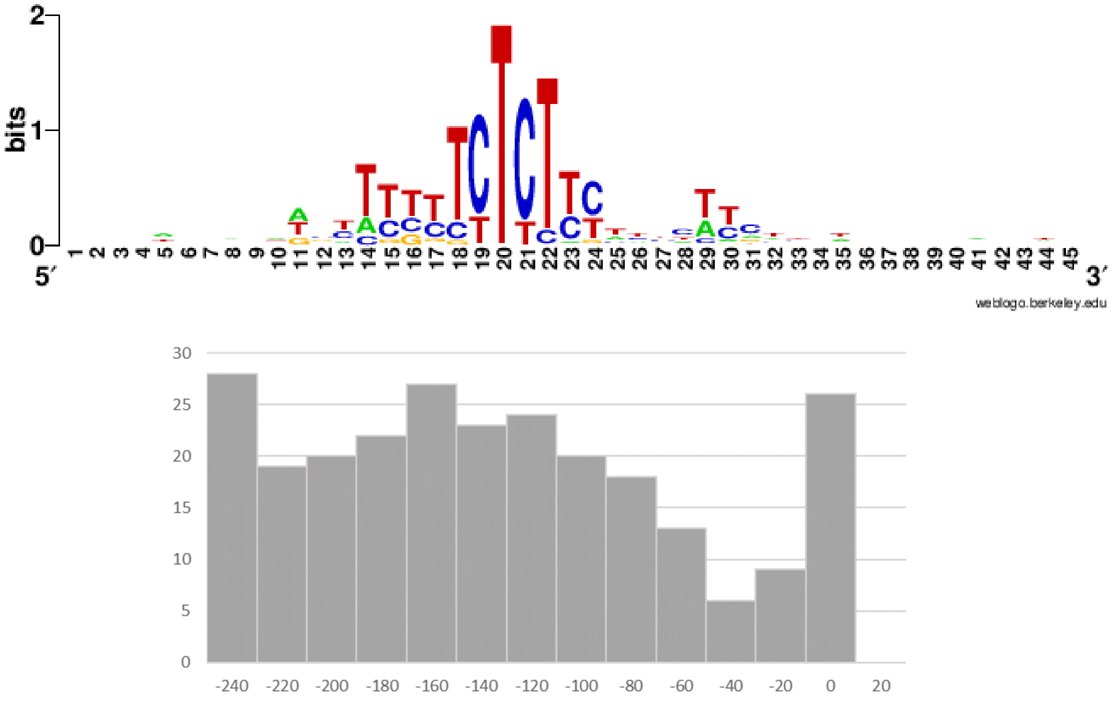

# Pithovirus and Cedratvirus

1041 fragments

## Motif #13 (128 sites)

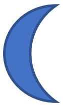

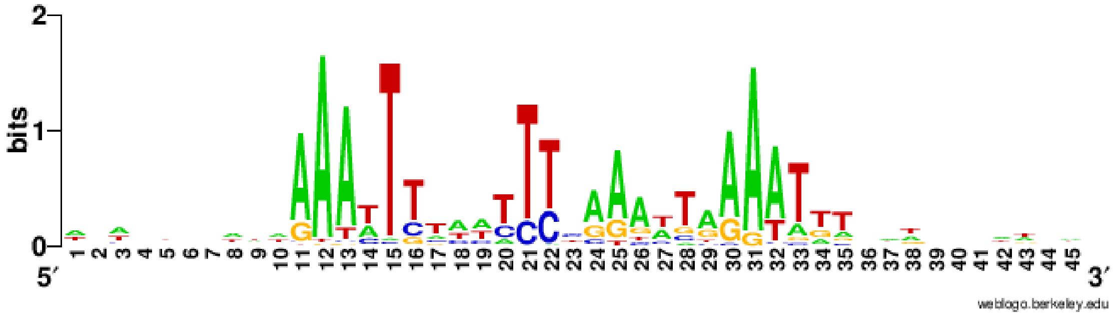


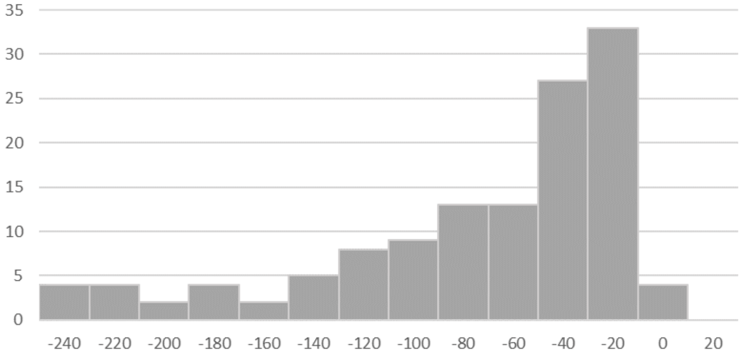


## Motif #15 (122 sites)

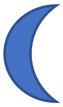

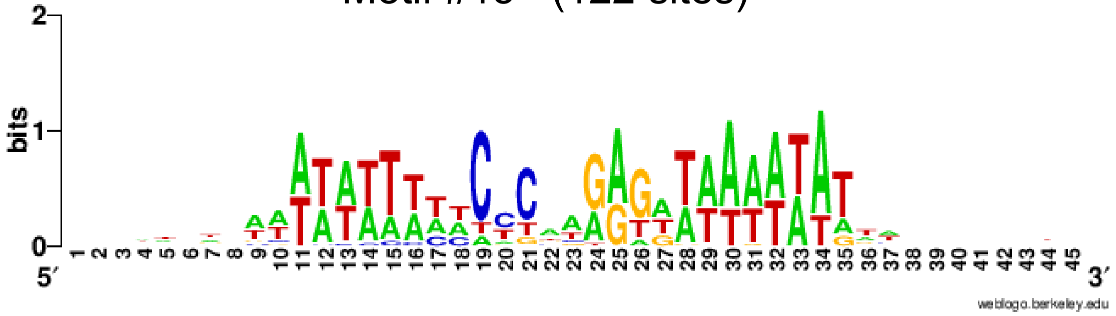


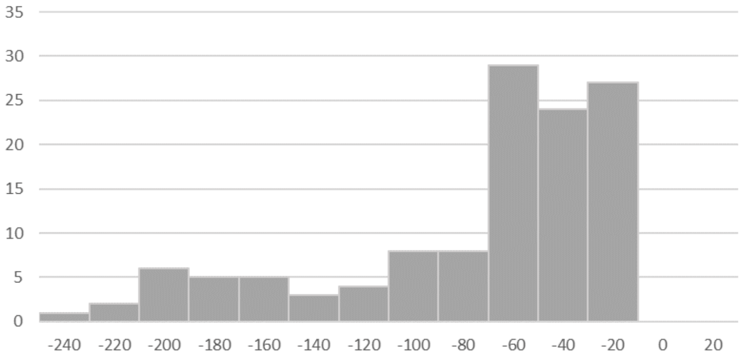

**Orpheovirus**
1199 fragments

Motif #1 (304 sites)

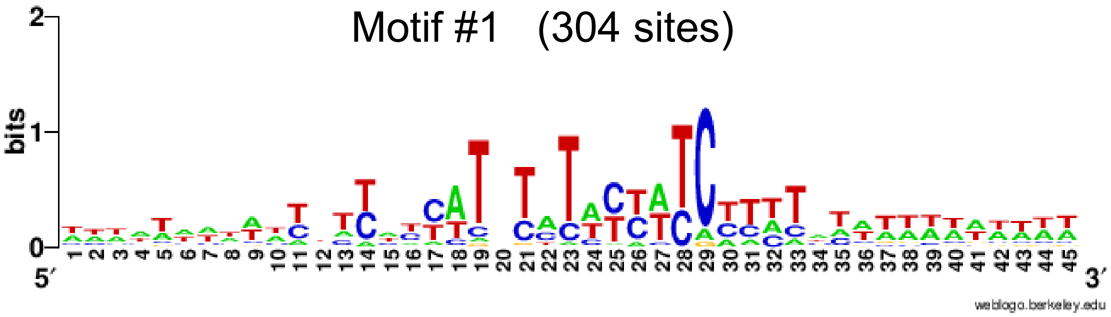


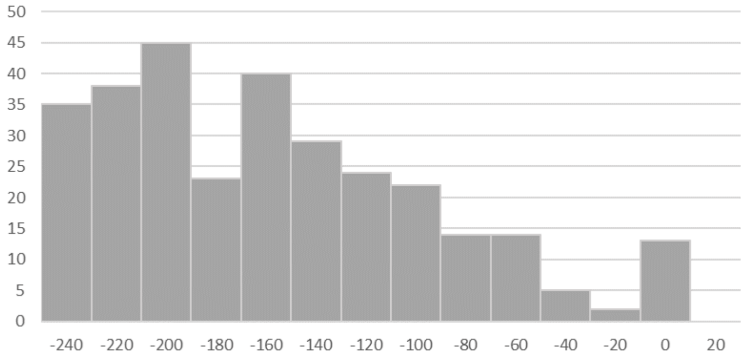

Motif #2 (320 sites)

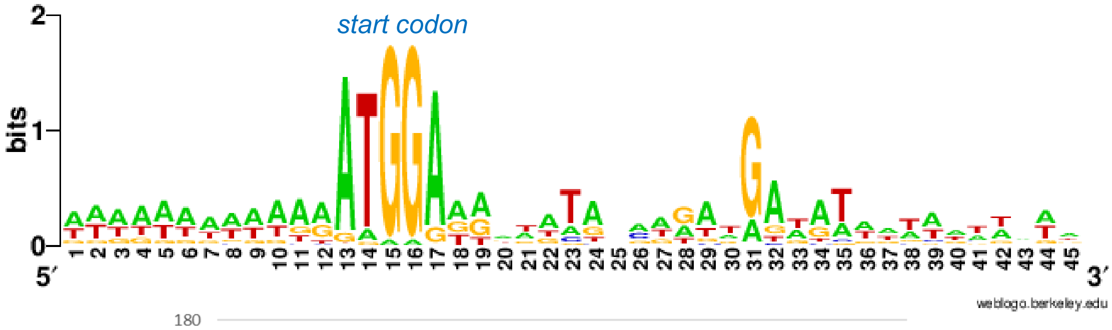


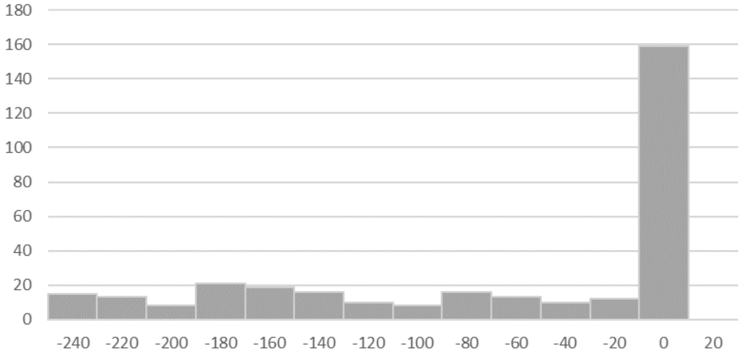

**Orpheovirus**

1199 fragments

## Motif #3 (407 sites)

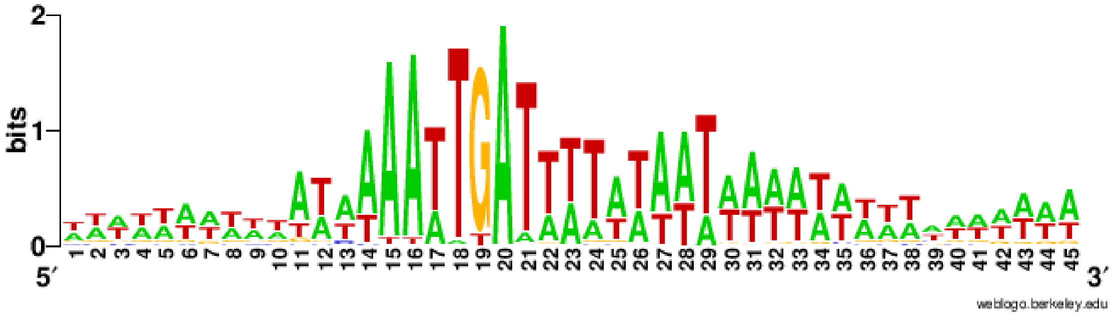


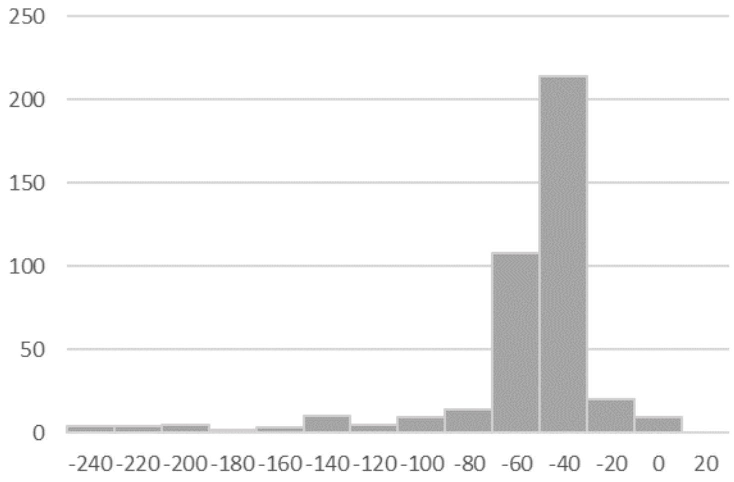


## Motif #4 (152 sites)

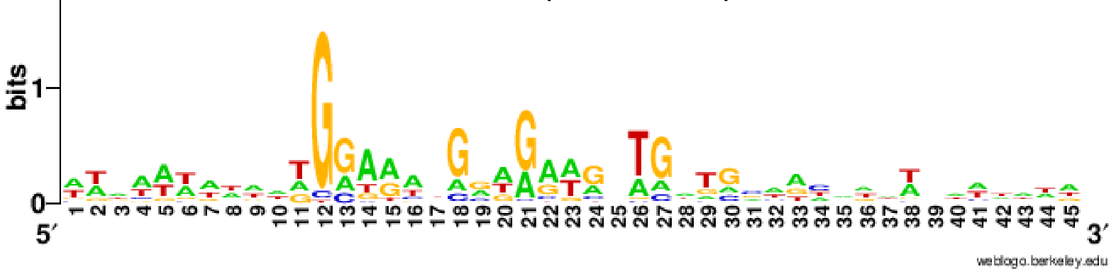


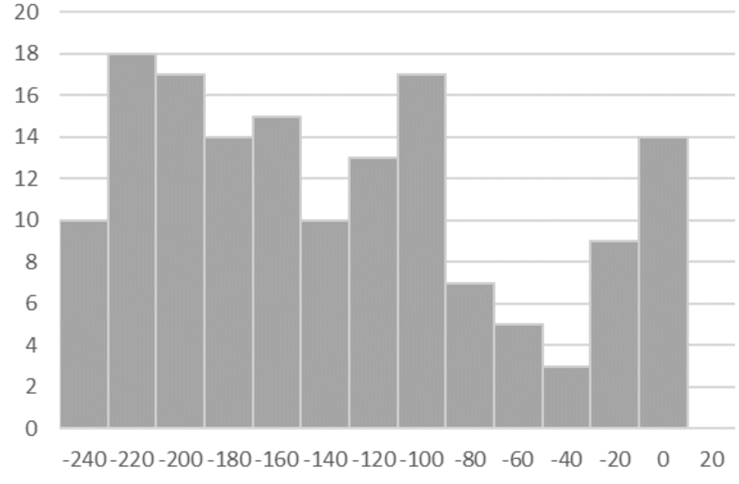

**Orpheovirus**
1199 fragments

Motif #5 (264 sites)

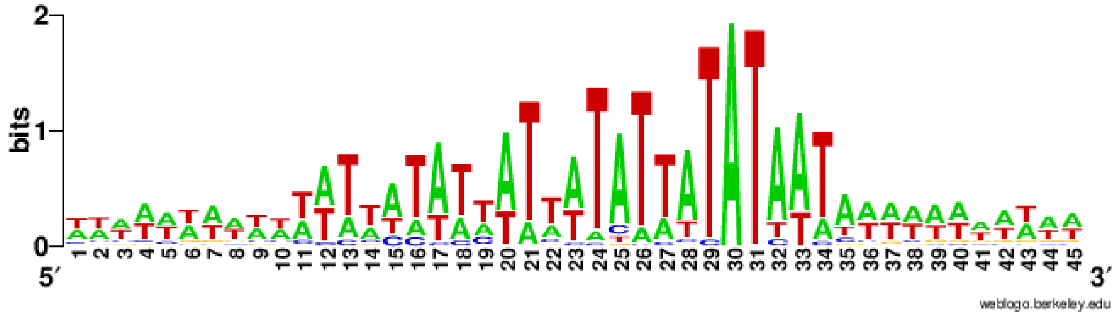


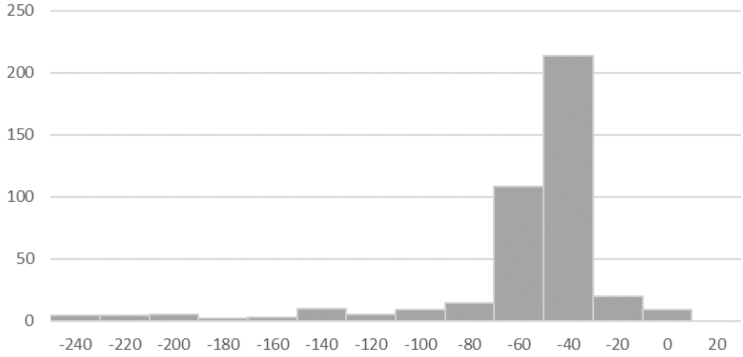


Motif #6 (150 sites)

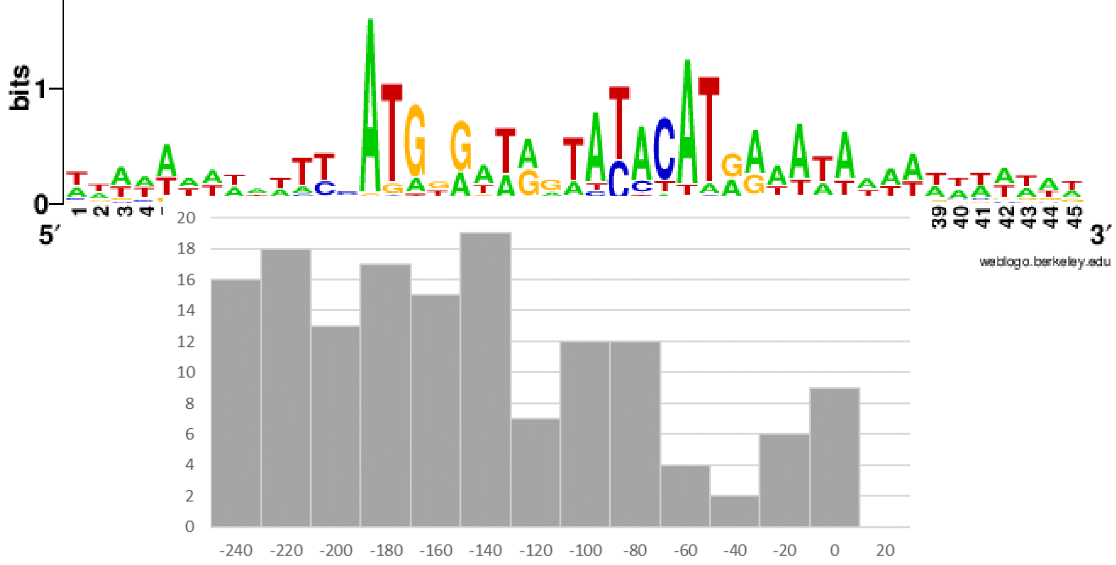

**LCPAC401_403_404_406**

1708 fragments

## Motif #1 (888 sites)

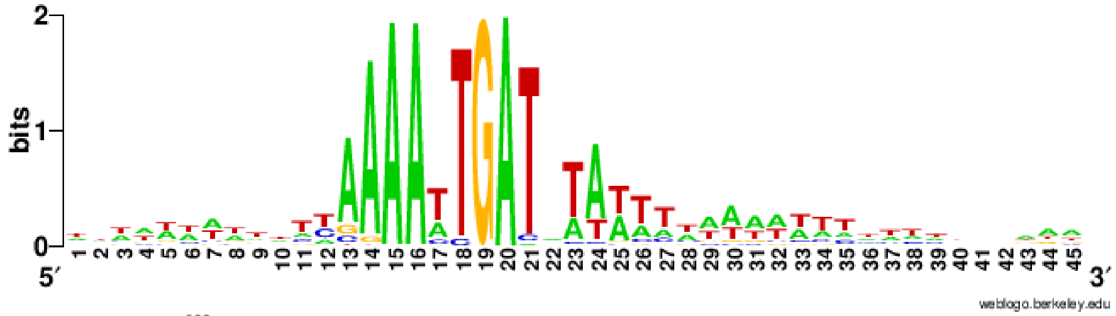


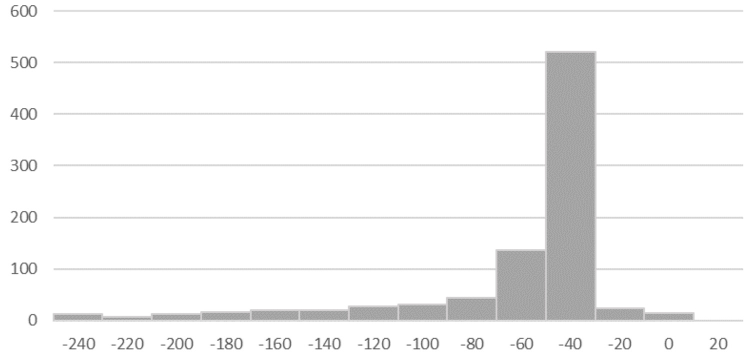


## Motif #2 (170 sites)

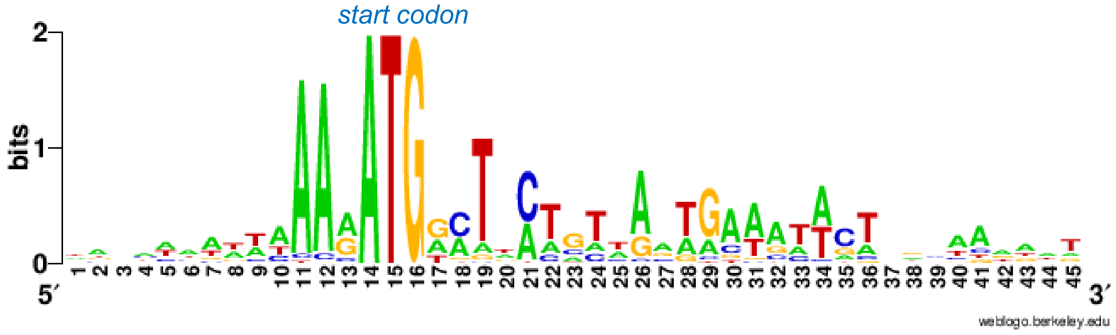


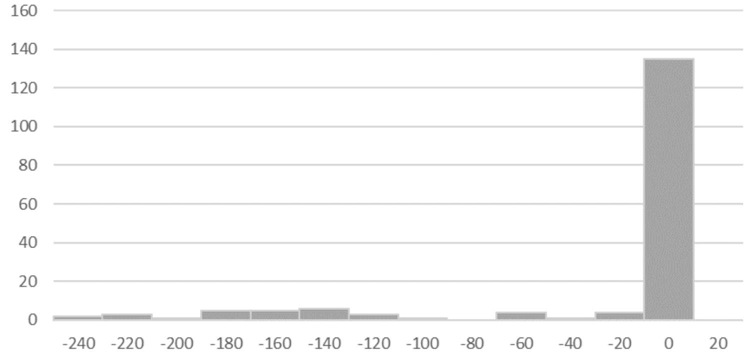

# LCPAC401_403_404_406

1708 fragments

## Motif #3 (170 sites)

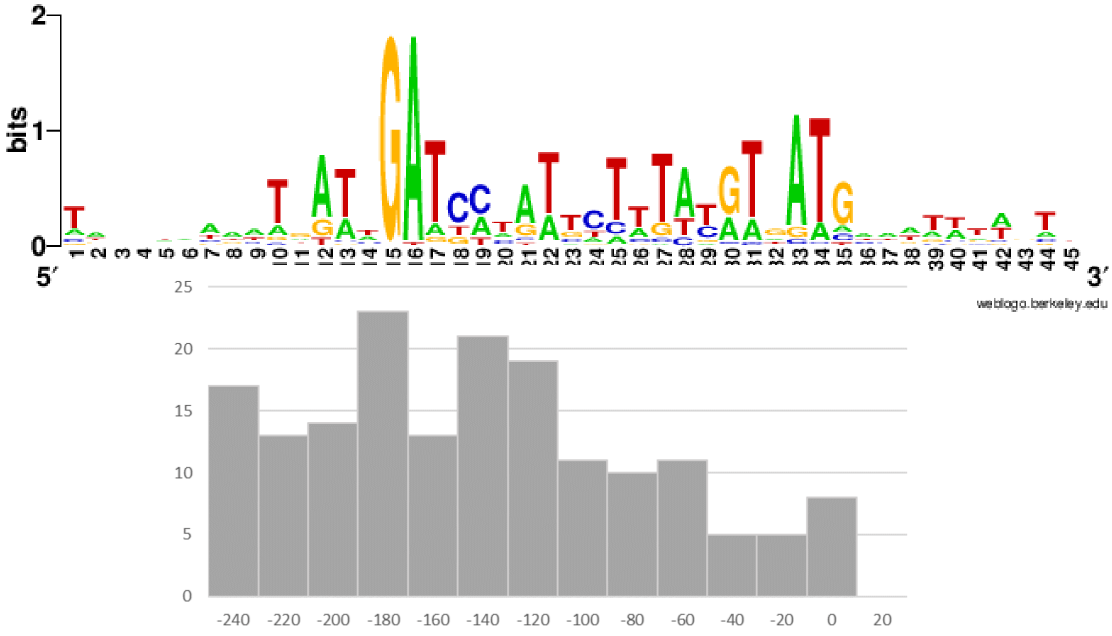


## Motif #4 (258 sites)

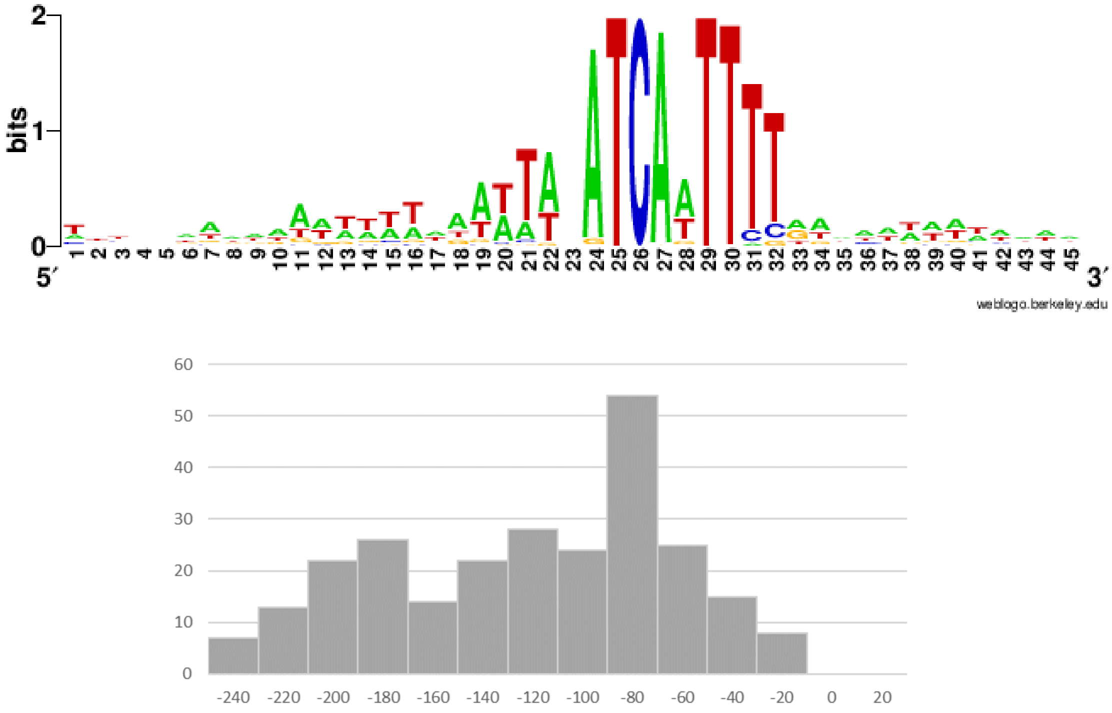

# LCPAC401_403_404_406

1708 fragments

## Motif #5 (172 sites)

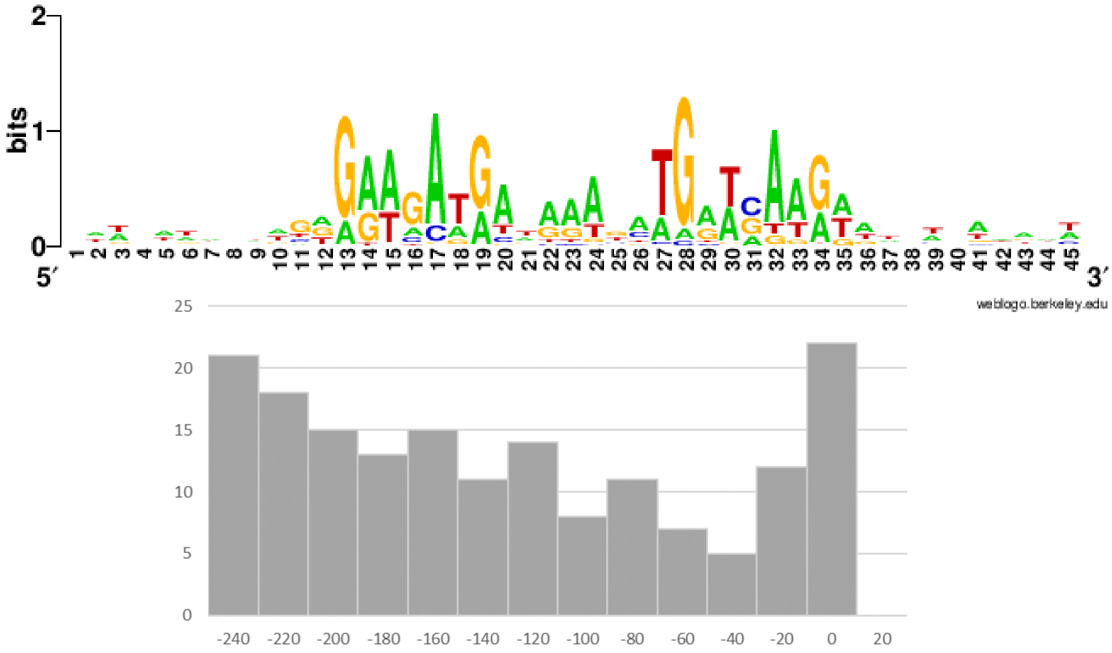


## Motif #10 (260 sites)

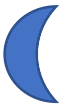

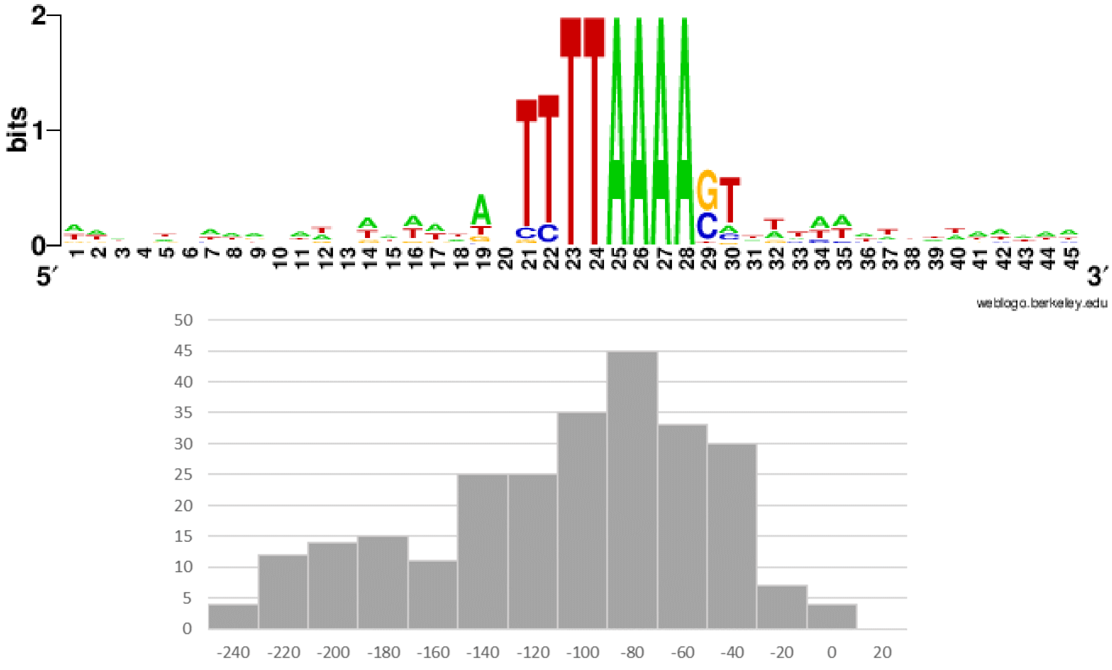

**LCDPAC01**

269 fragments

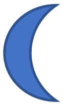

## Motif #1 (72 sites)

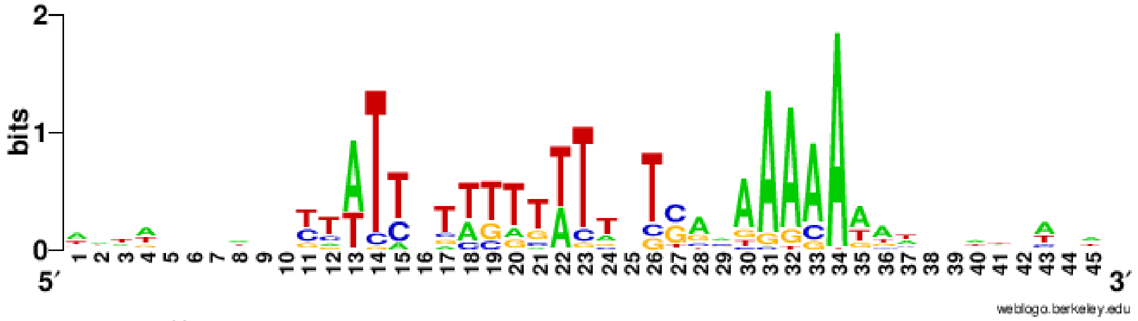


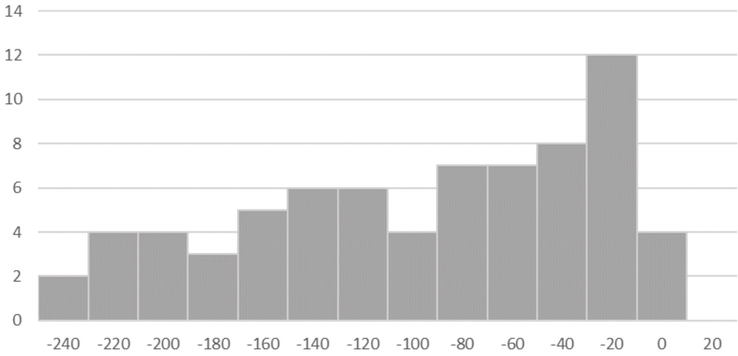


## Motif #2 (57 sites)

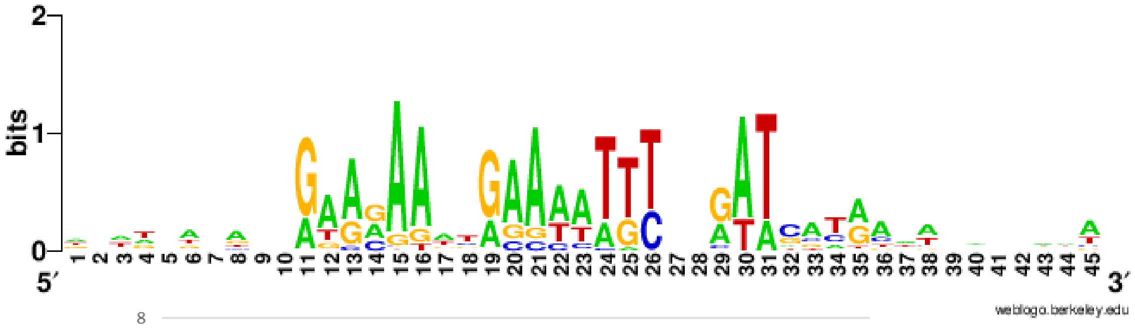


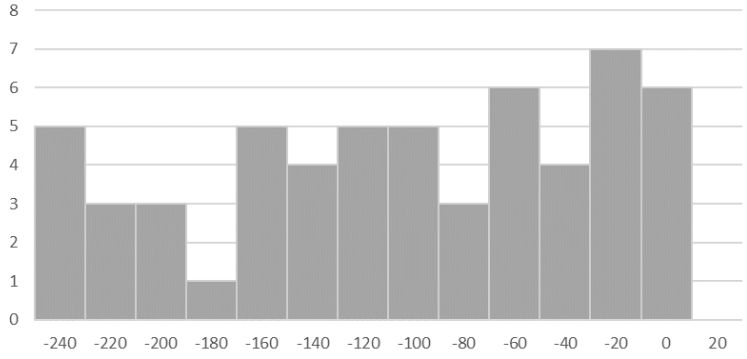

**LCDPAC01**

269 fragments

Motif #3 (50 sites)

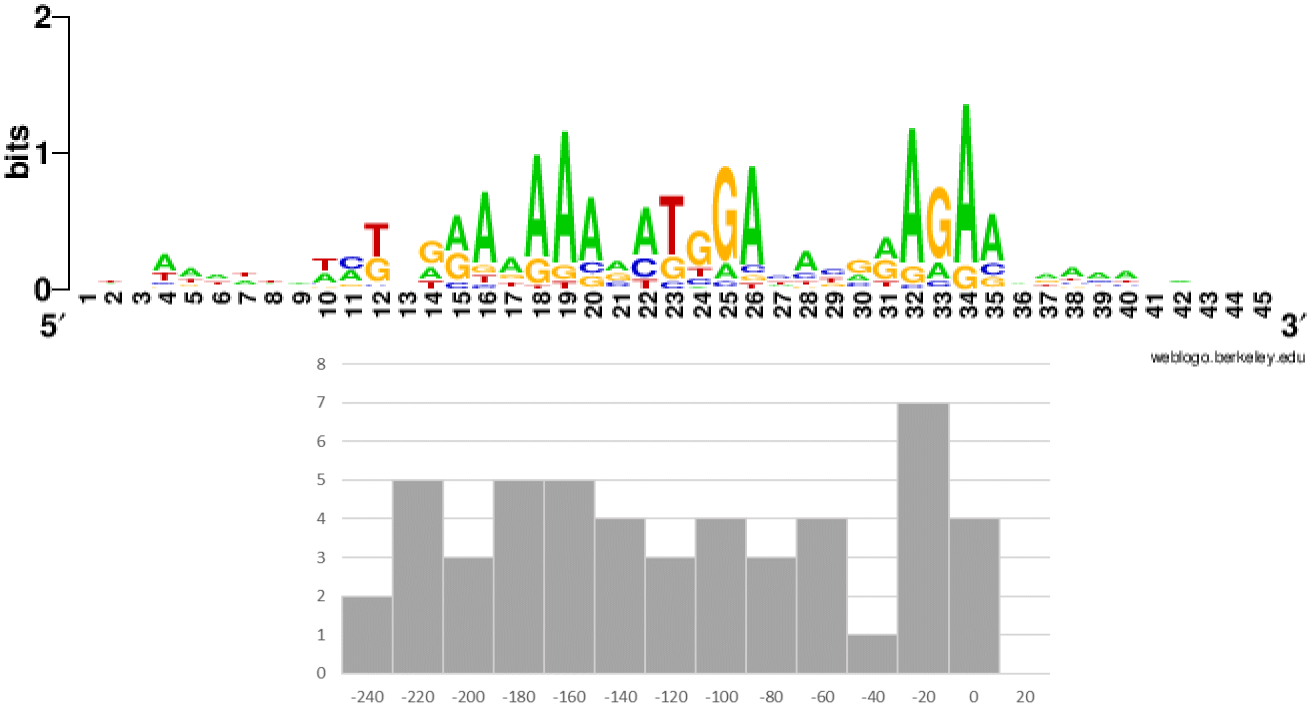


Motif #5 (67 sites)

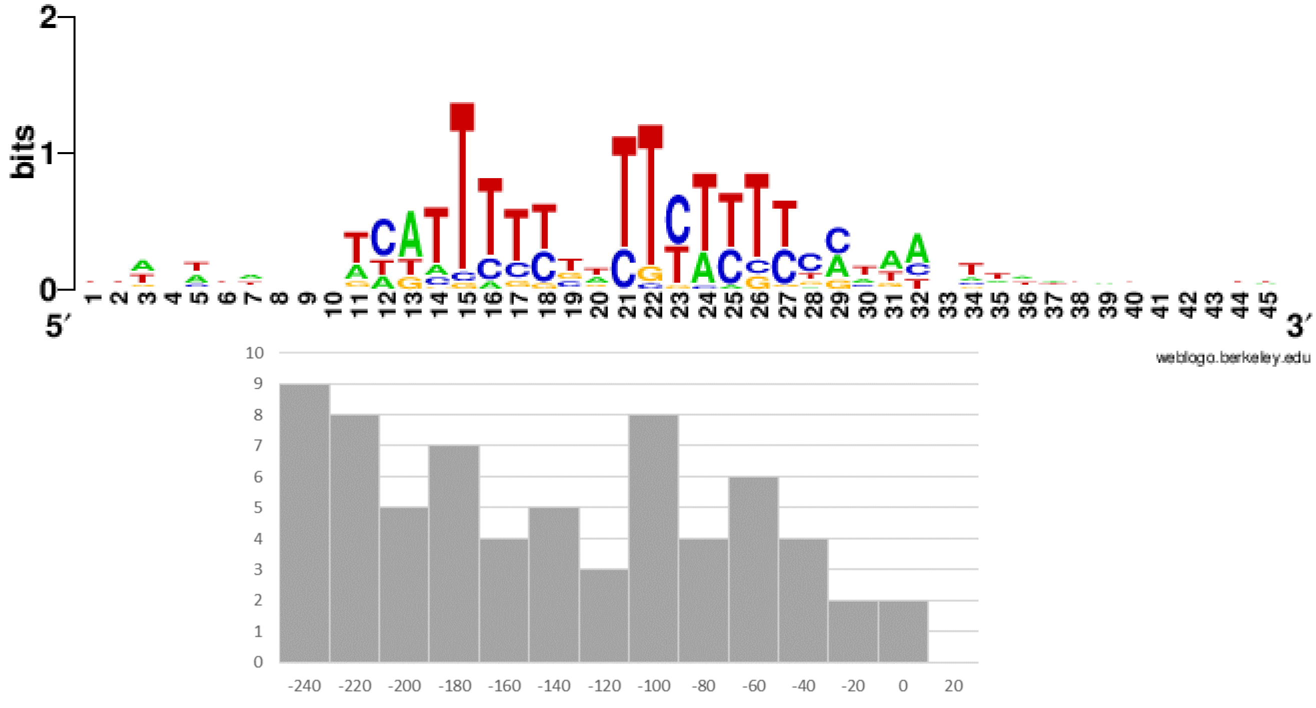

**LCDPAC02**

386 fragments

## Motif #1 (266 sites)

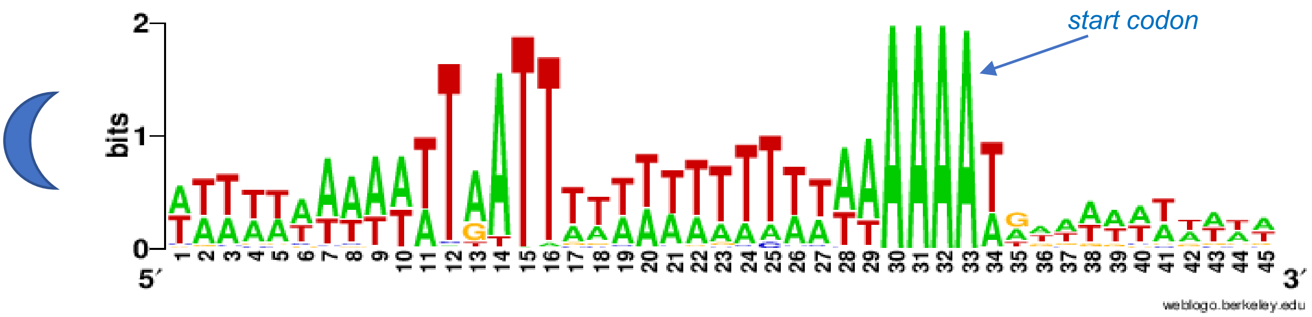


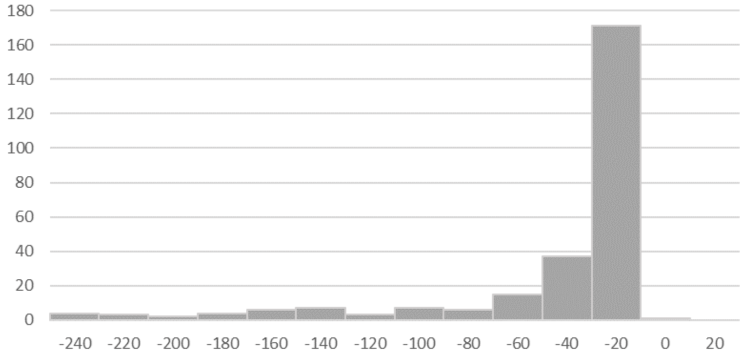


## Motif #2 (87 sites)

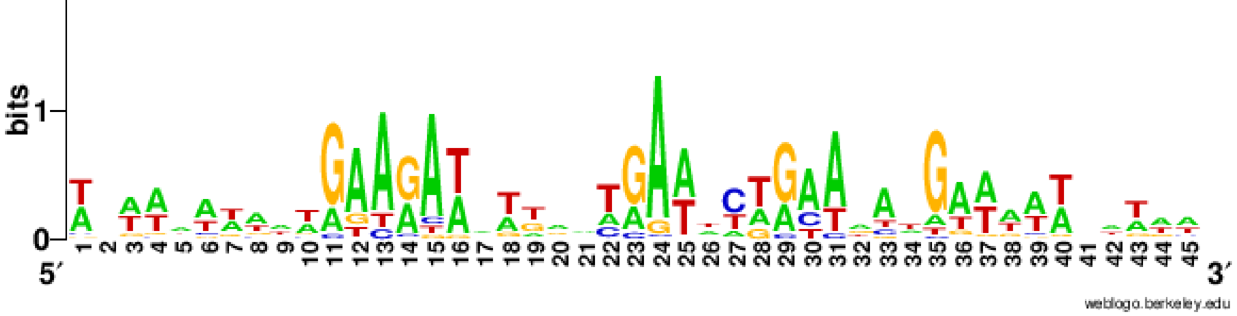


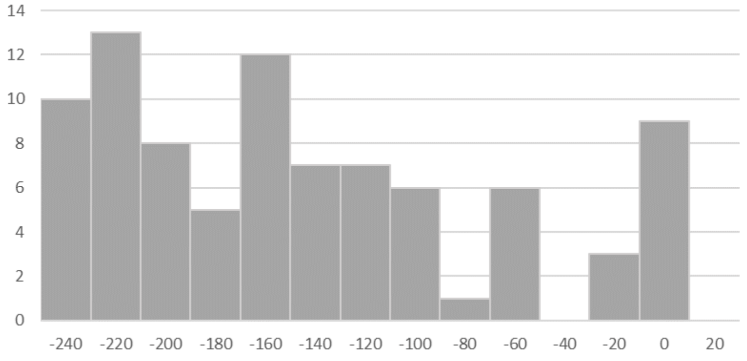

# LCDPAC02

386 fragments

## Motif #3 (52 sites)

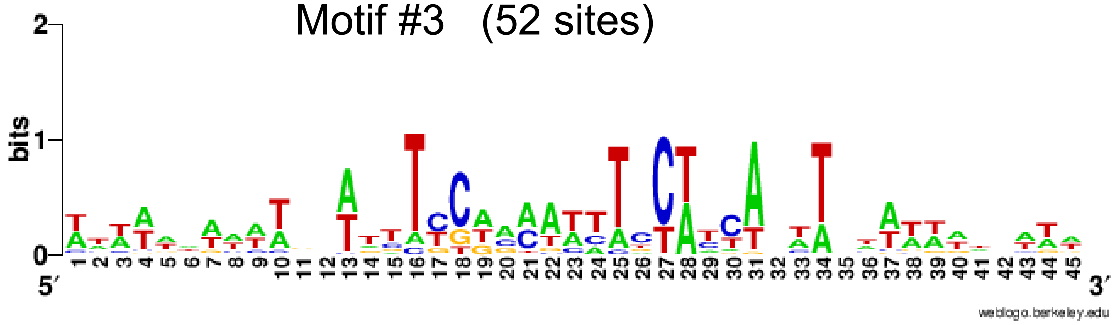


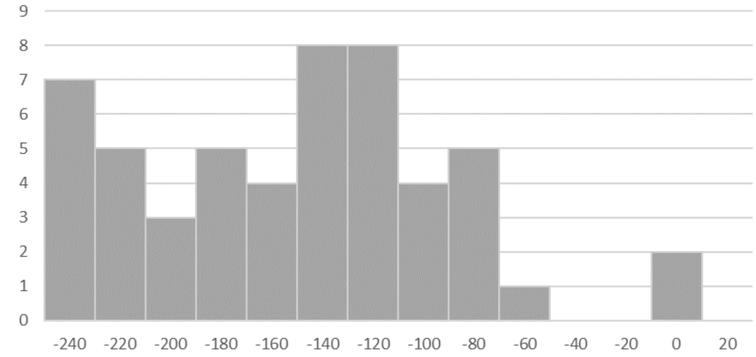


## Motif #6 (67 sites)

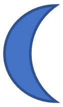

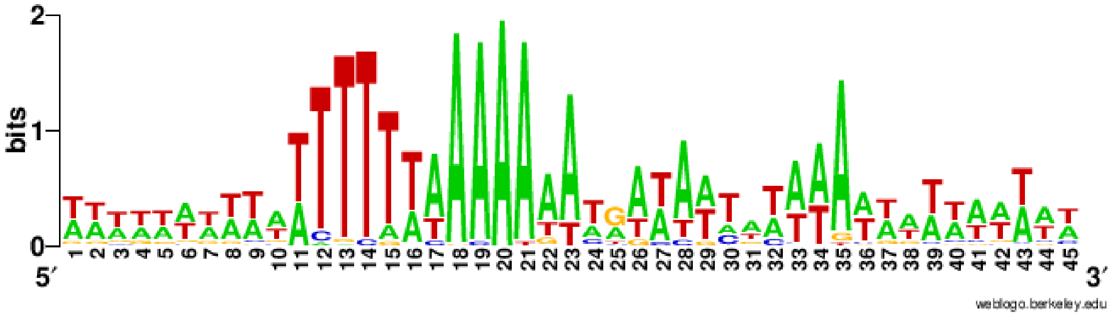


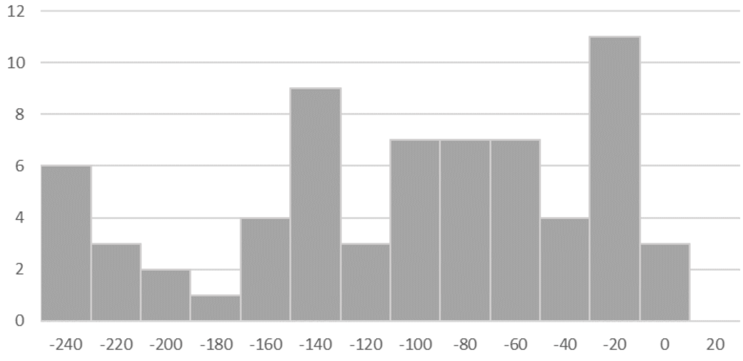

**All pithoviruses**
7379 fragments

Motif #2 (941 sites)

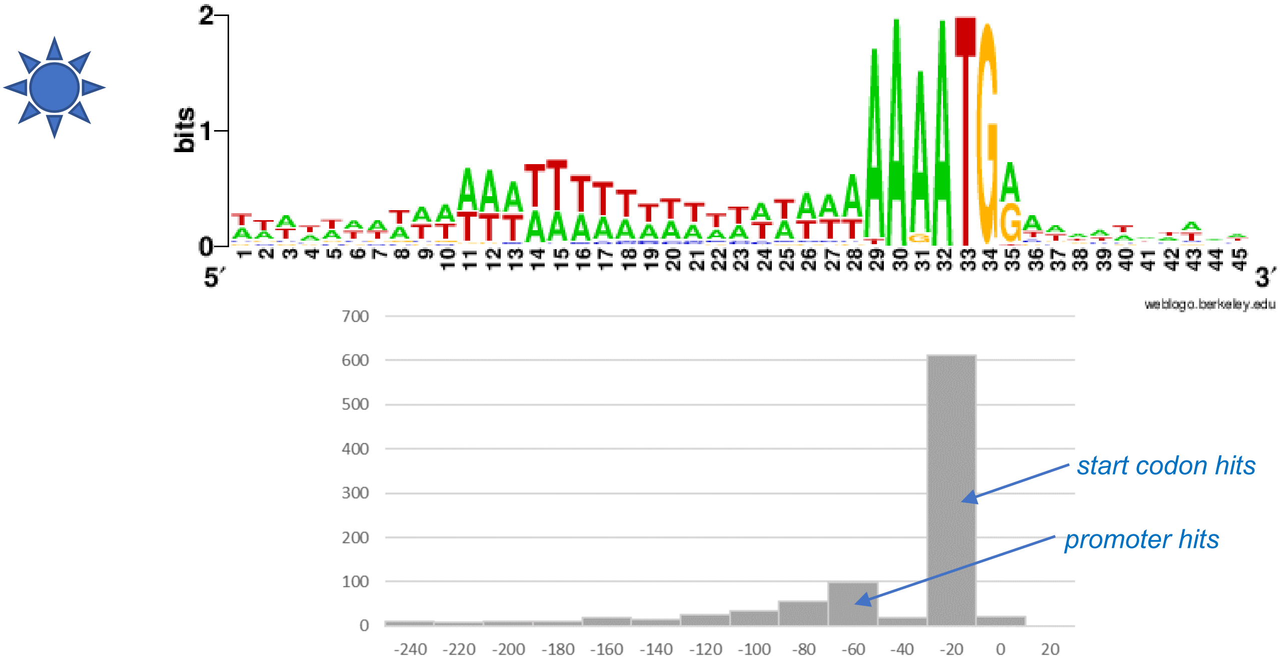


Motif #5 (1510 sites)

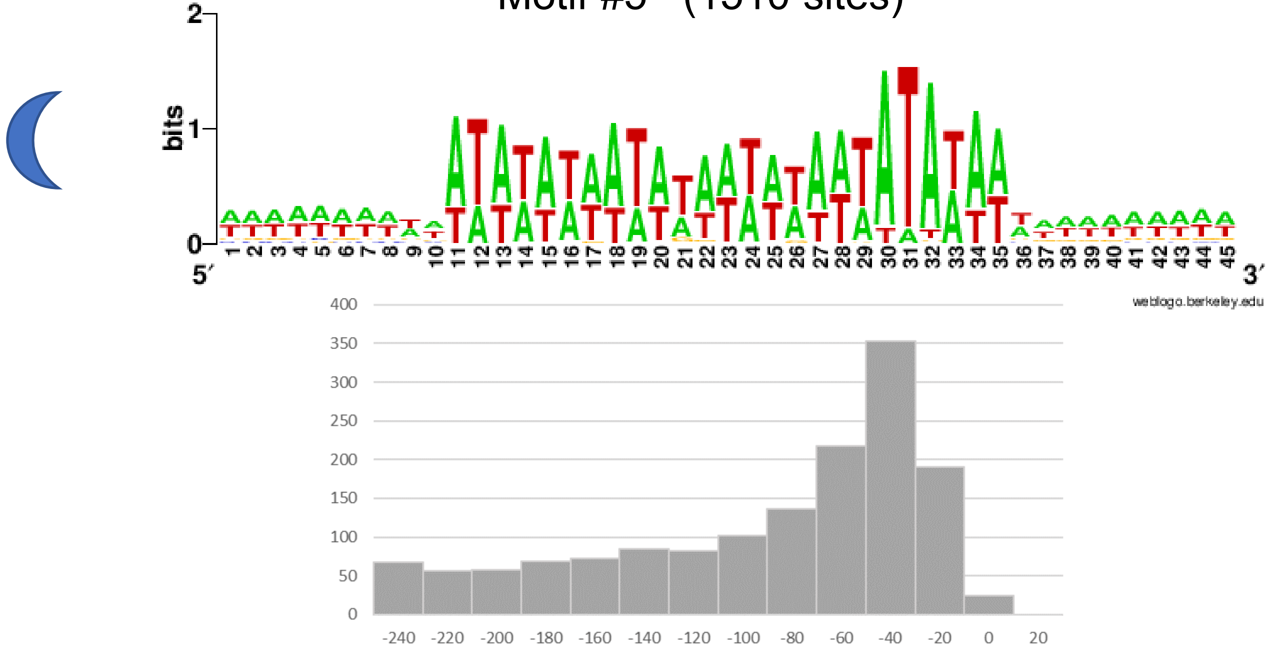

Supplement: TEXT S7 [file mBio.02497-18-s0007.pdf]
