# Supplementary material for: Virus Genomes from Deep Sea Sediments Expand the Ocean Megavirome and Support Independent Origins of Viral Gigantism
Source: mBio. 2019 Mar 5;10(2):e02497-18. doi: 10.1128/mBio.02497-18 (PMC6401483; doi:10.1128/mBio.02497-18)

This file contains meme motif search results for Loki Castle Viruses of Marseillevirus and Iridovirus groups

Marseillevirus marseillevirus (NC_013756.1) is included

The ‘upstream’ regions are from -250 to 30 nt related to the start codon; the regions shorter than 50 nt are filtered out

Meme search was set to 25 nt of motif width

Upstream region sequences and meme output files can be found at: ftp://ftp.ncbi.nih.gov/pub/yutinn/Loki_Castle_NCLDV_2018/meme_motif_search

Conserved motifs AAA(T/A)TGA are marked with the ‘sun’ sign
Conserved AT-rich motifs are marked with the ‘moon’ sign

note: on all Logo images, the detected motif starts from position #11

LCMAC201

550 fragments

Motif #1 (186 sites)

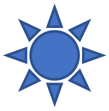

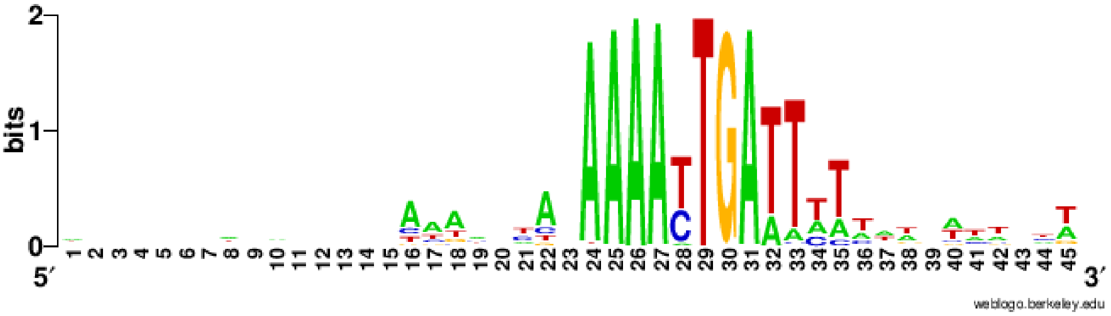

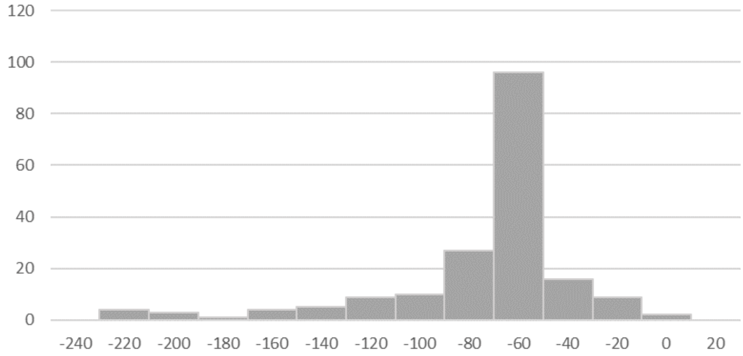

Motif #2 (136 sites)

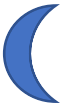

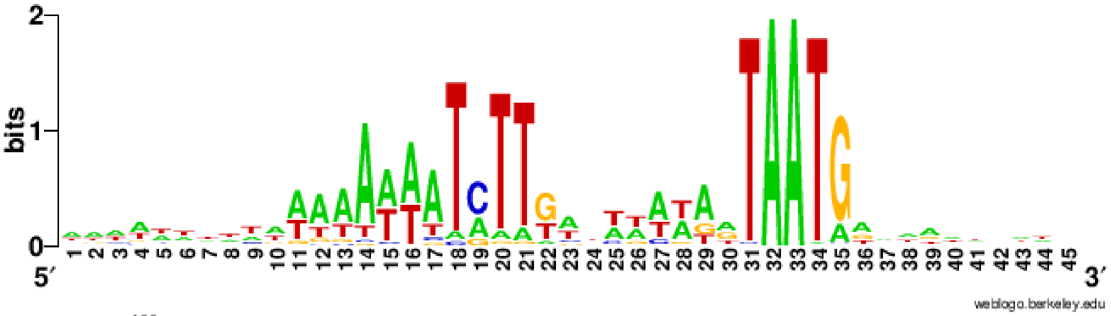

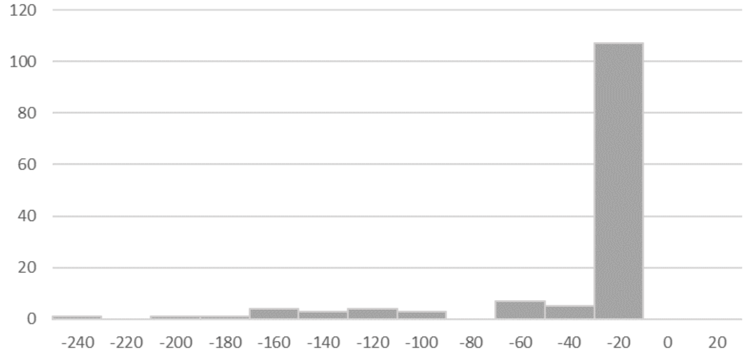

LCMAC201

550 fragments

Motif #3 (85 sites)

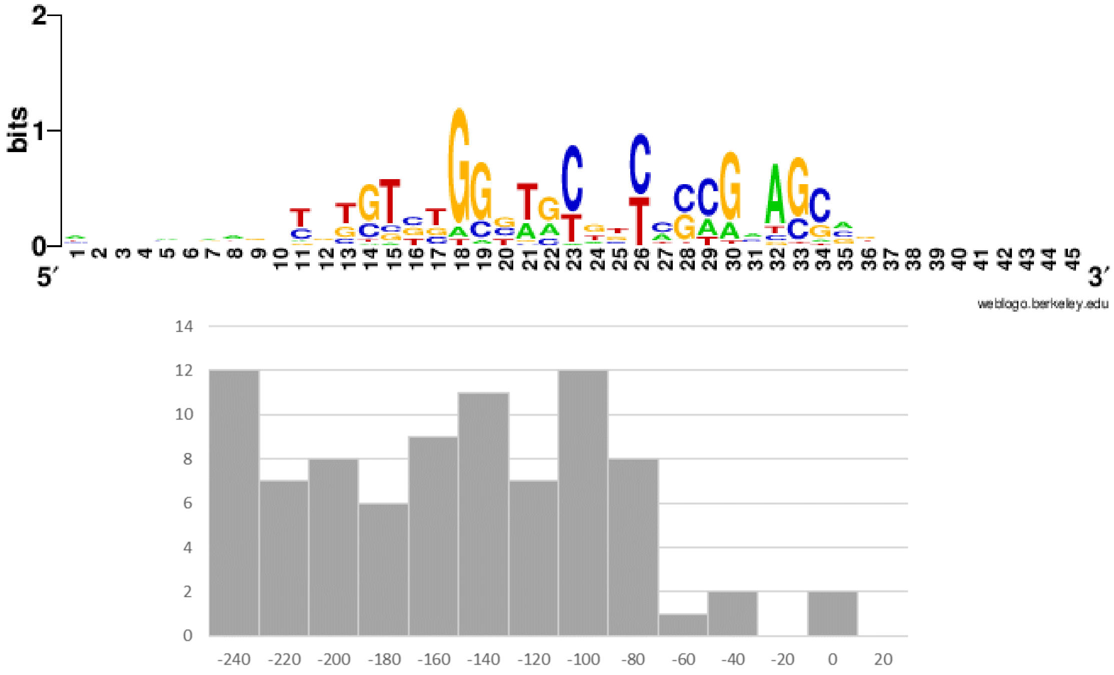

Motif #4 (60 sites)

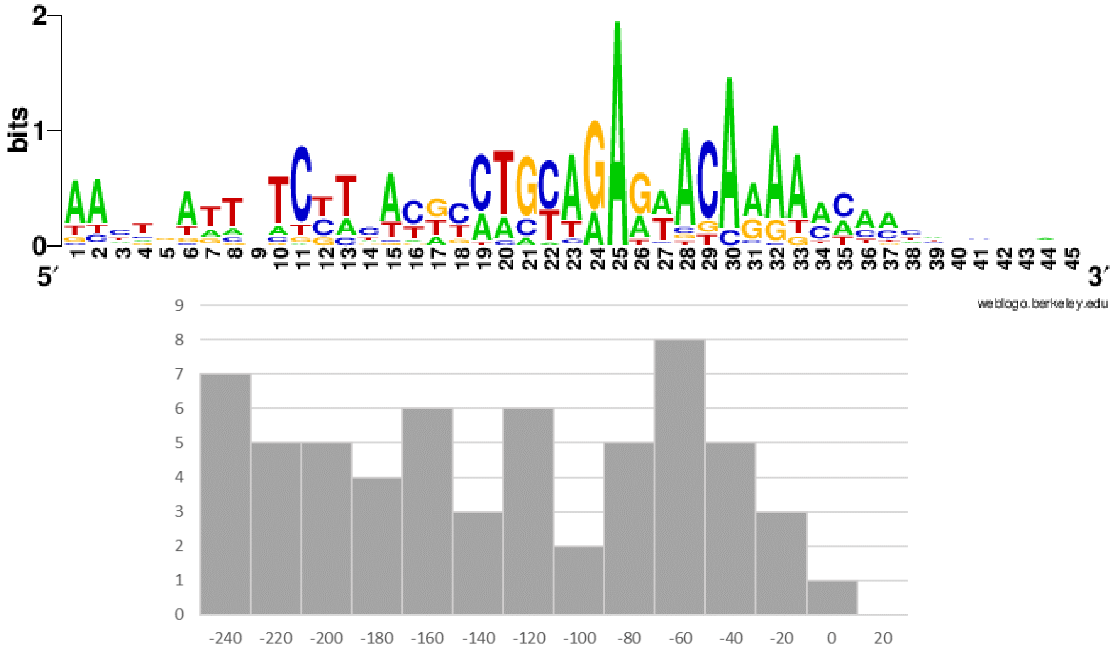

LCMAC202

658 fragments

Motif #1 (108 sites)

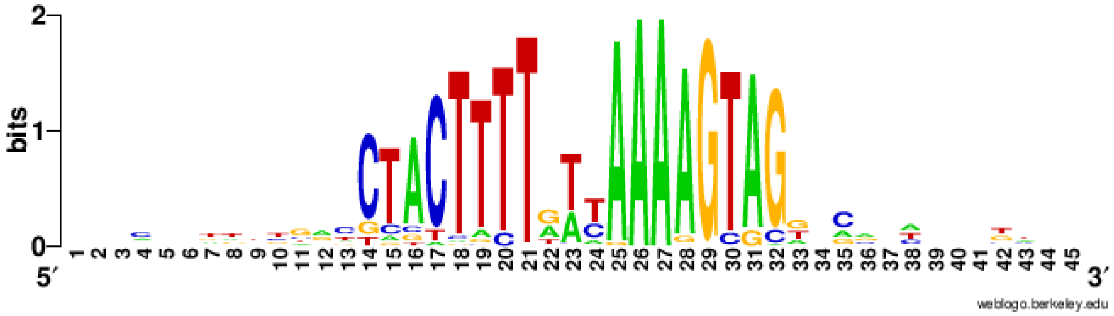

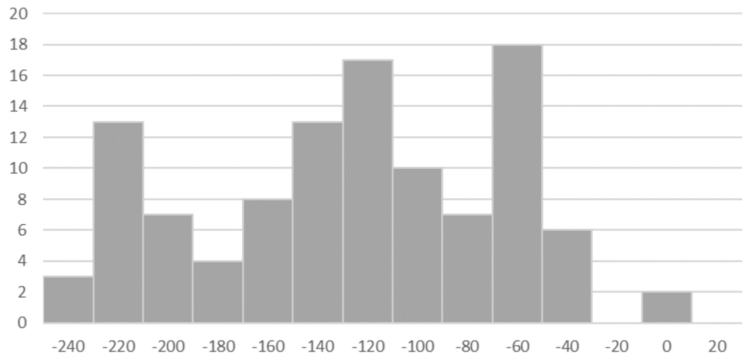

Motif #2 (87 sites)

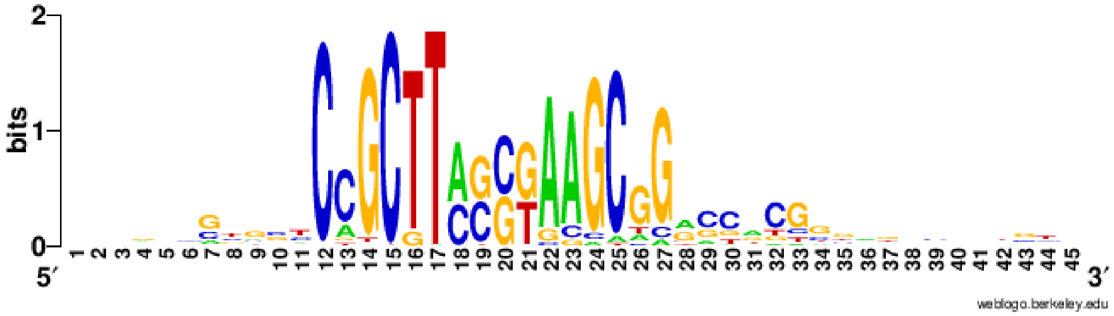

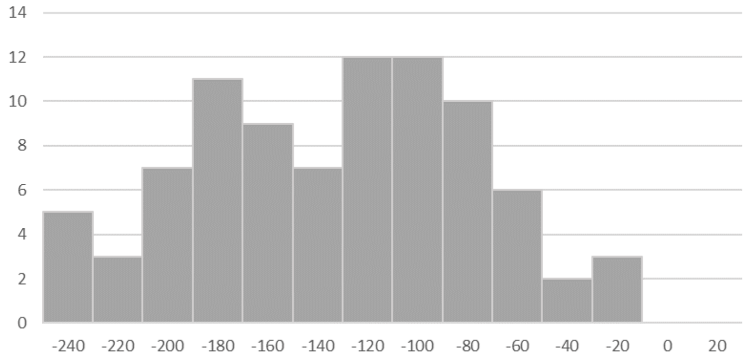

LCMAC202

658 fragments

Motif #3 (174 sites)

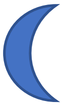

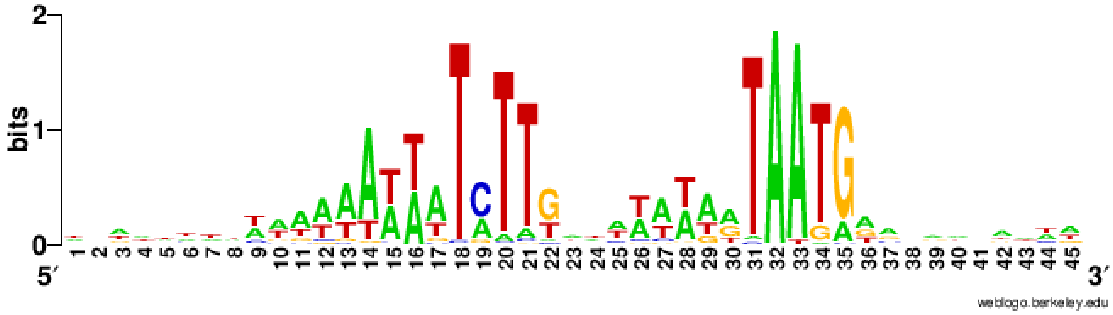

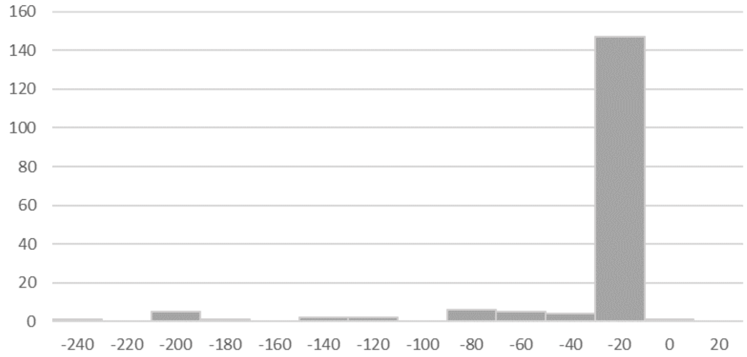

Motif #5 (200 sites)

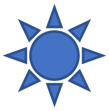

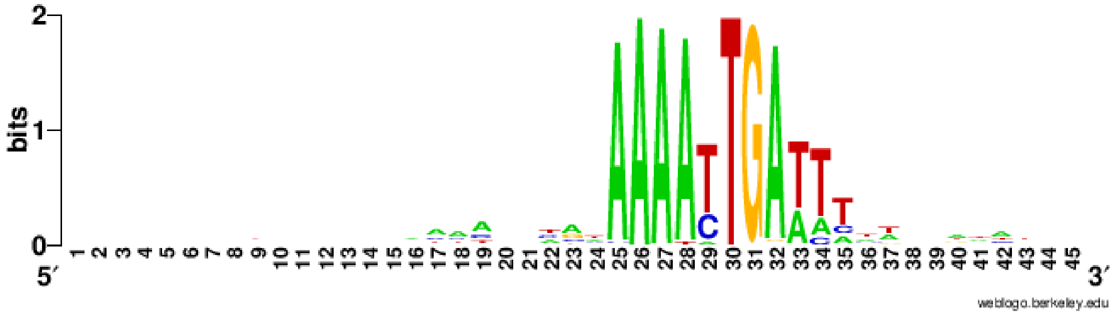

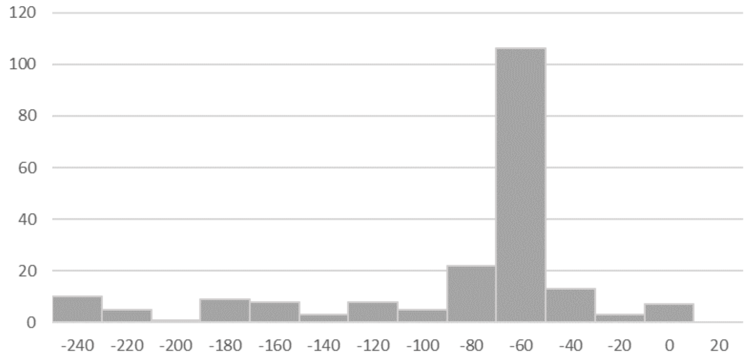

LCMAC101

772 fragments

Motif #1 (258 sites)

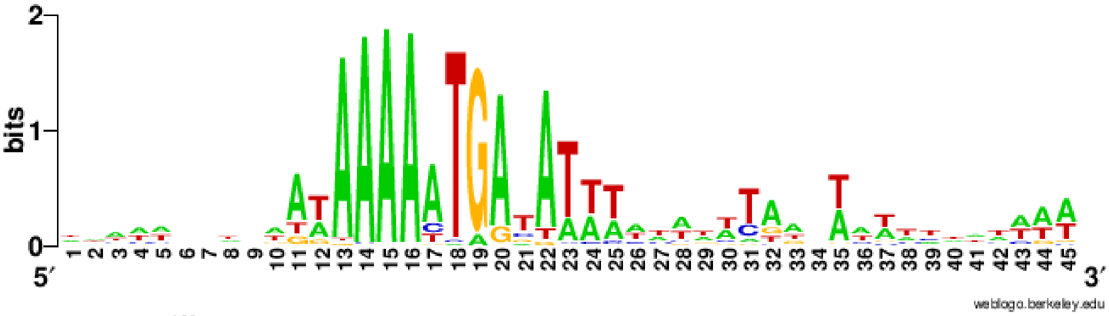

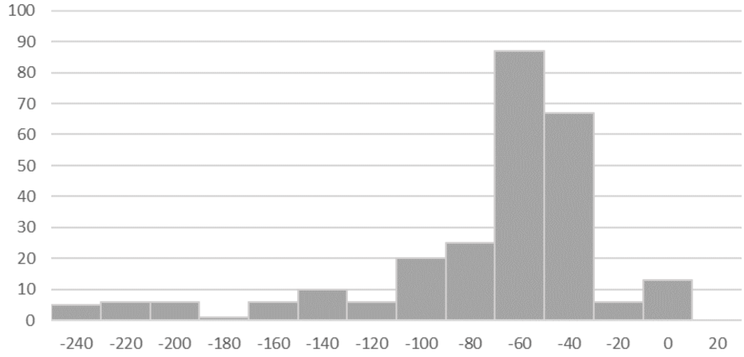

Motif #2 (159 sites)

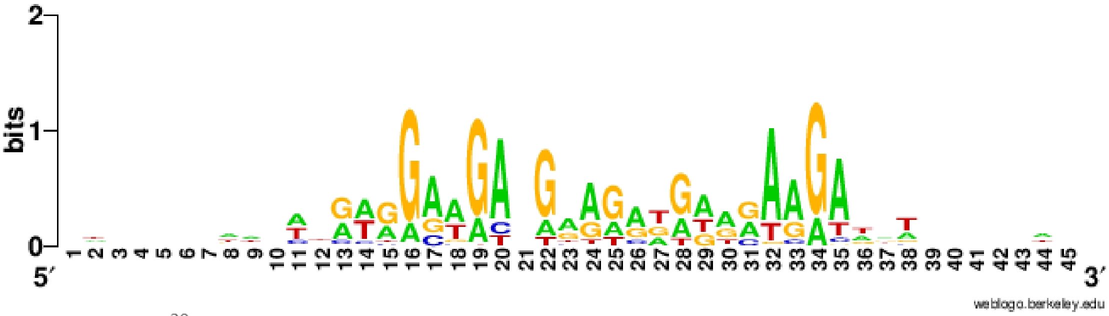

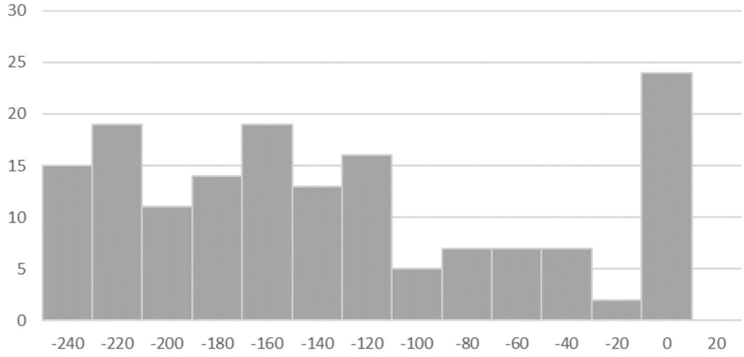

LCMAC102

464 fragments

Motif #1   (208 sites)

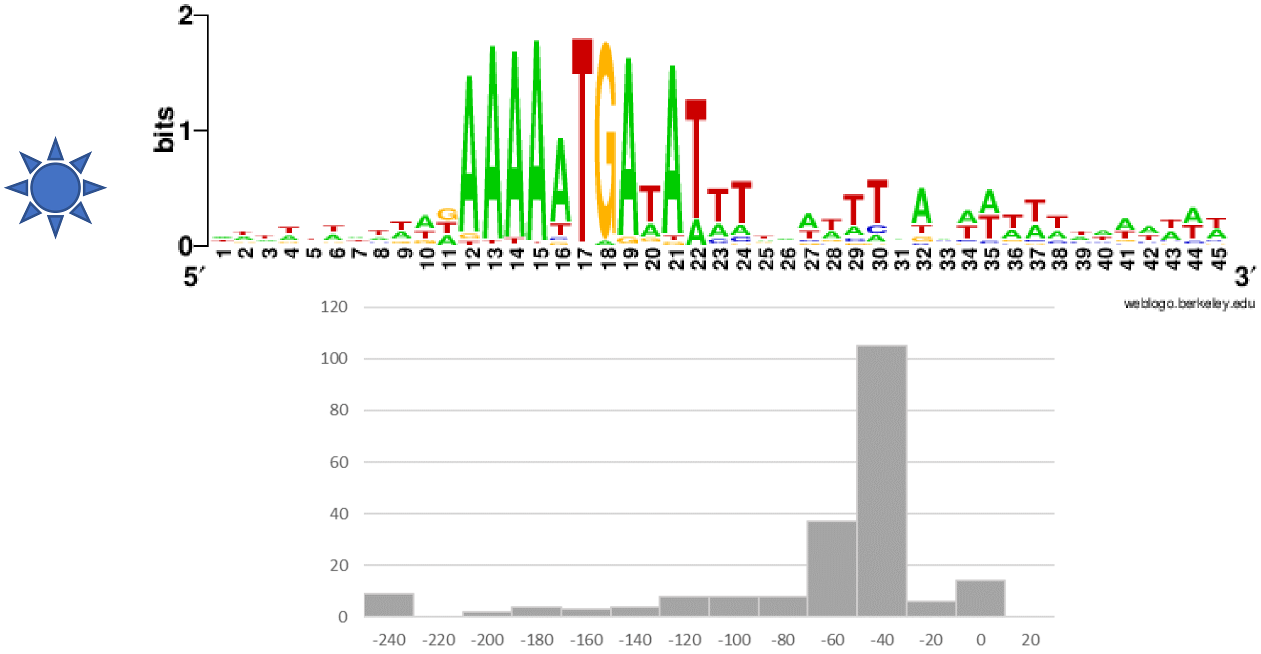

Motif #2   (81 sites)

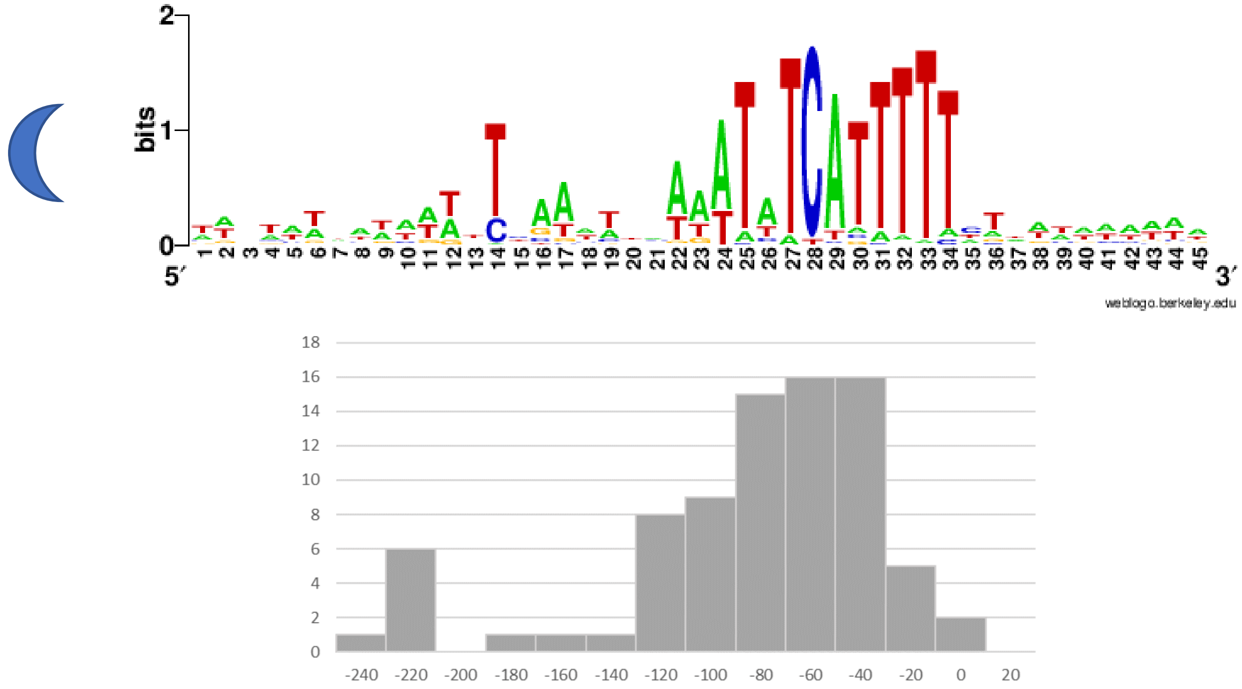

LCMAC103

422 fragments

Motif #1 (218 sites)

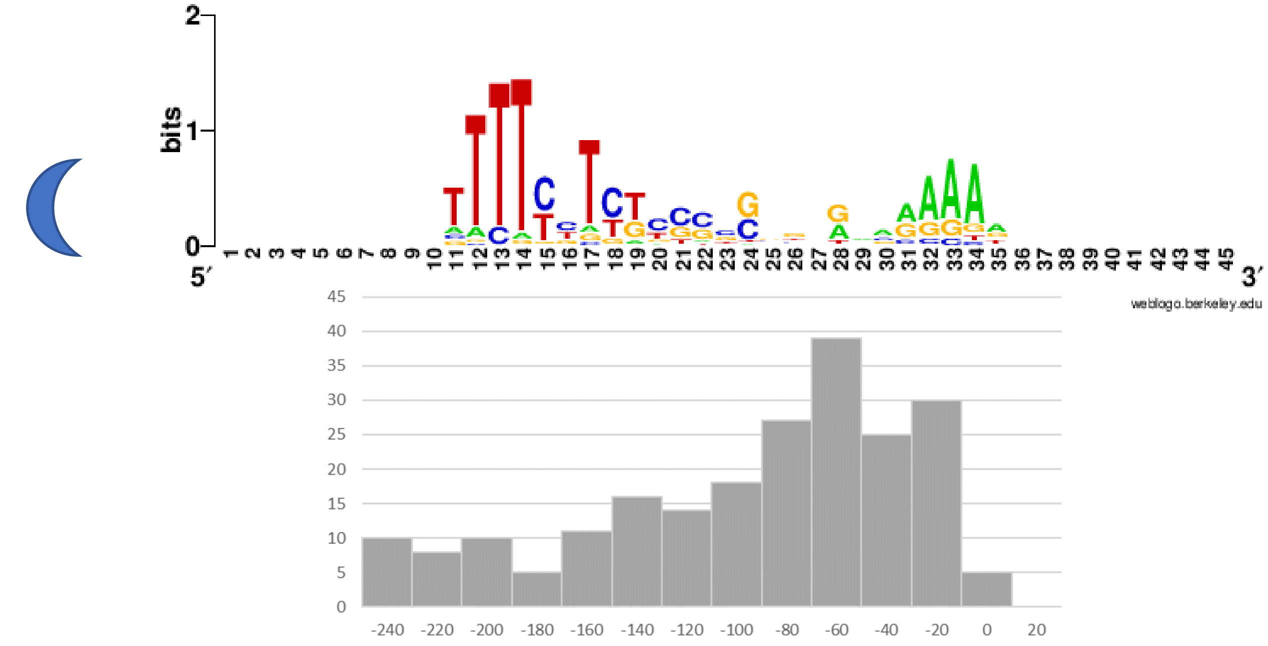

Motif #2 (192 sites)

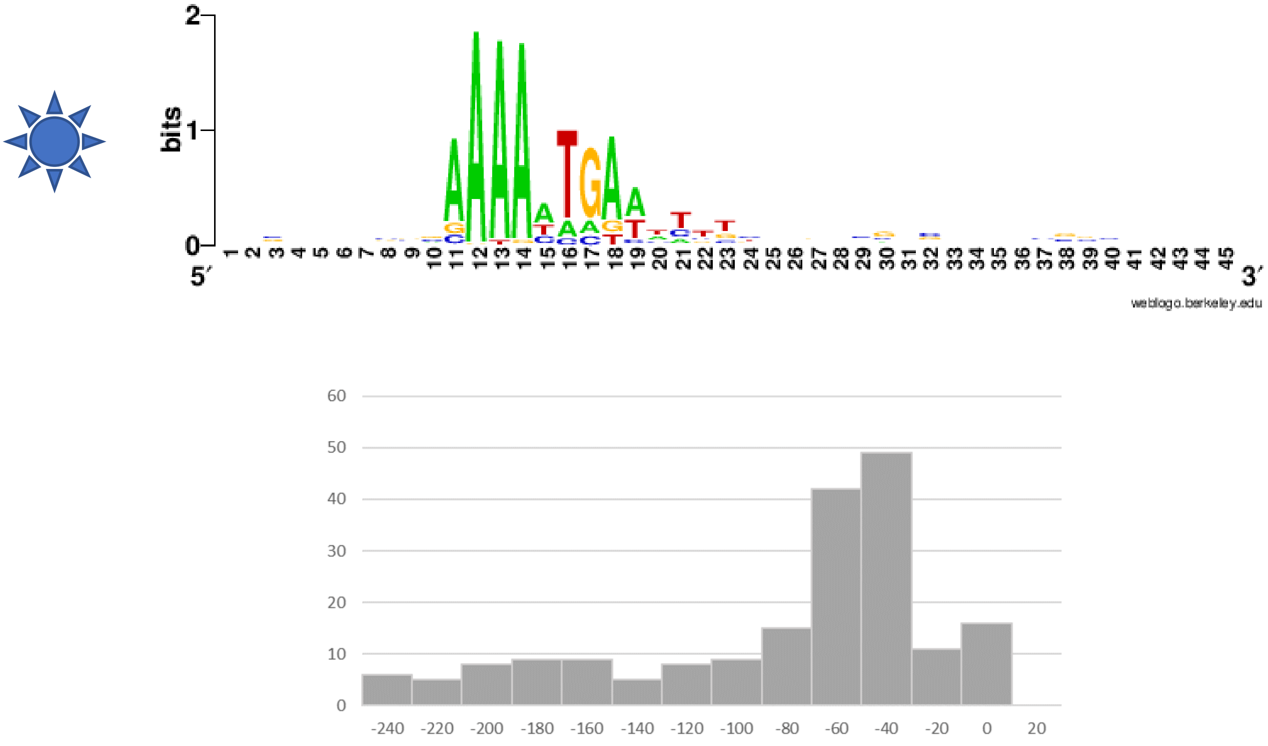

Marseillevirus marseillevirus

428 fragments

Motif #1 (305 sites)

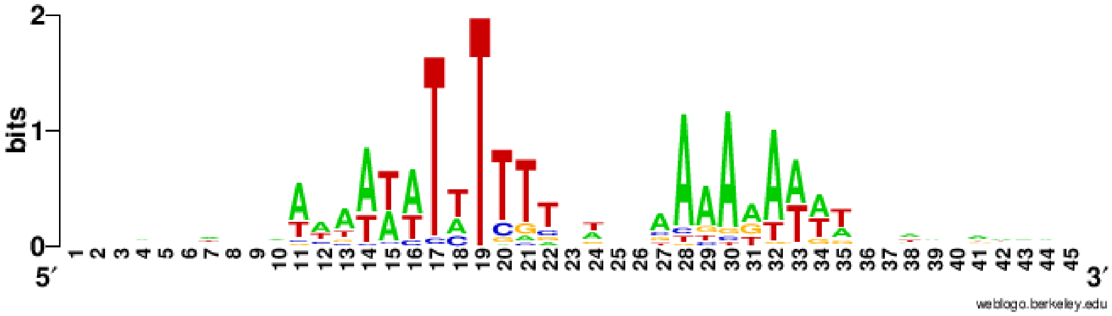

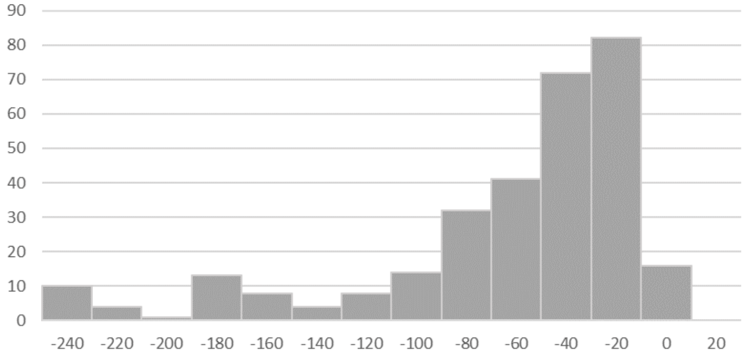

Motif #2 (426 sites)

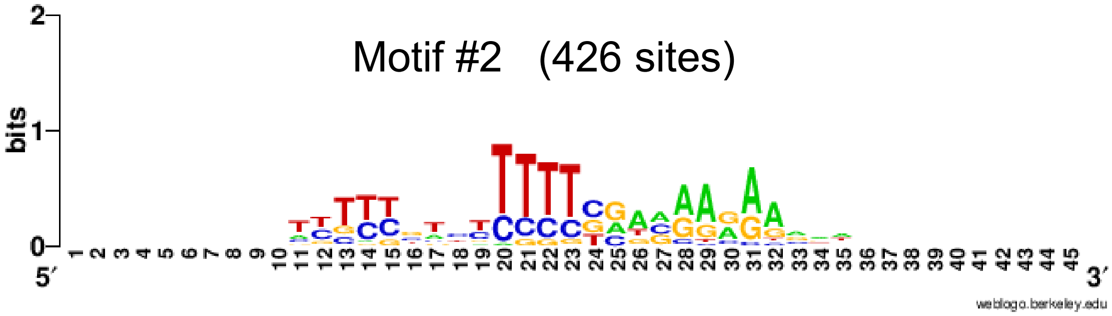

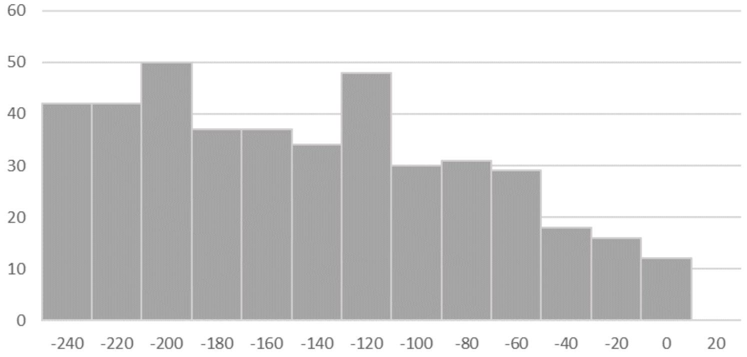

Marseillevirus marseillevirus

428 fragments

Motif #3 (334 sites)

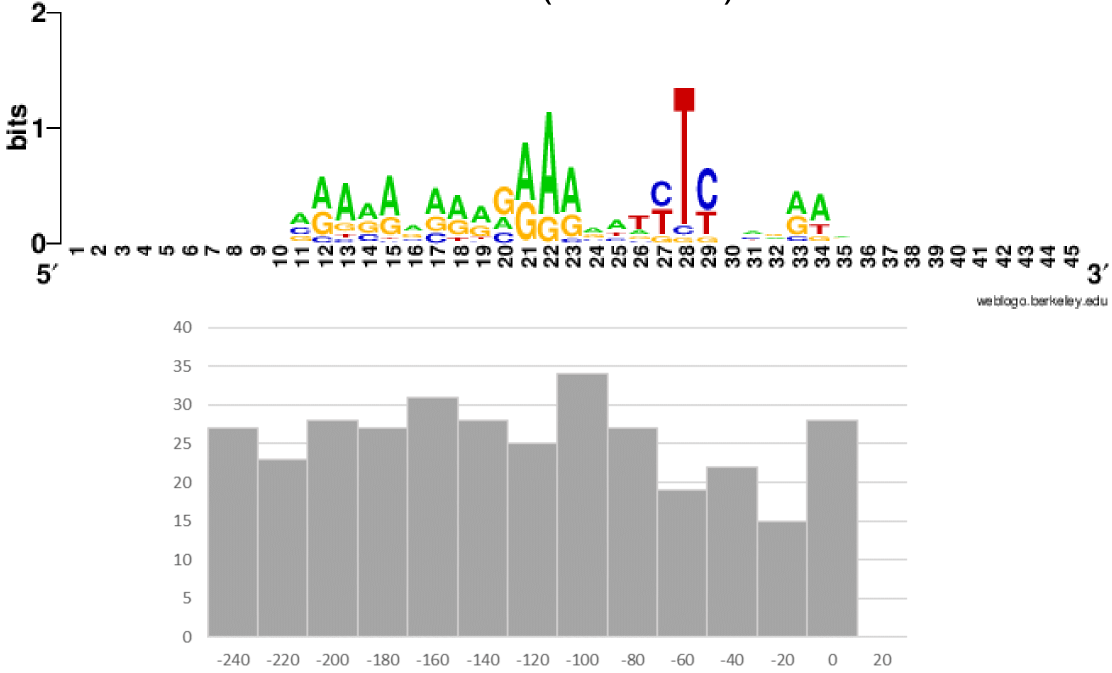

Motif #4 (150 sites)

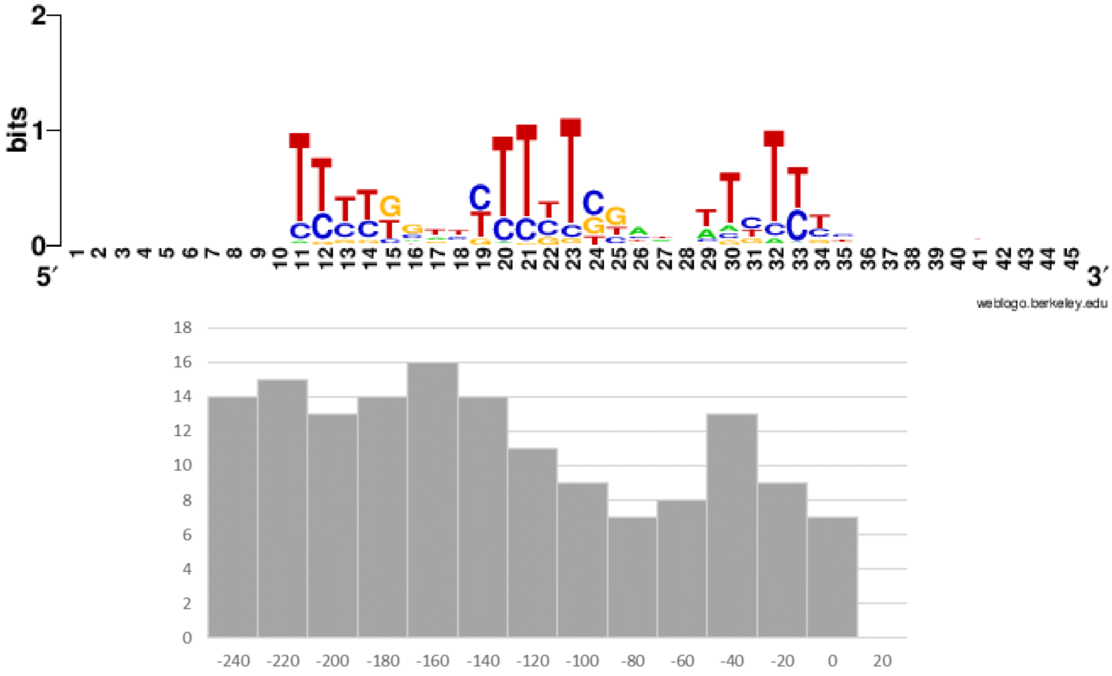

Marseillevirus marseillevirus

428 fragments

Motif #5 (122 sites)

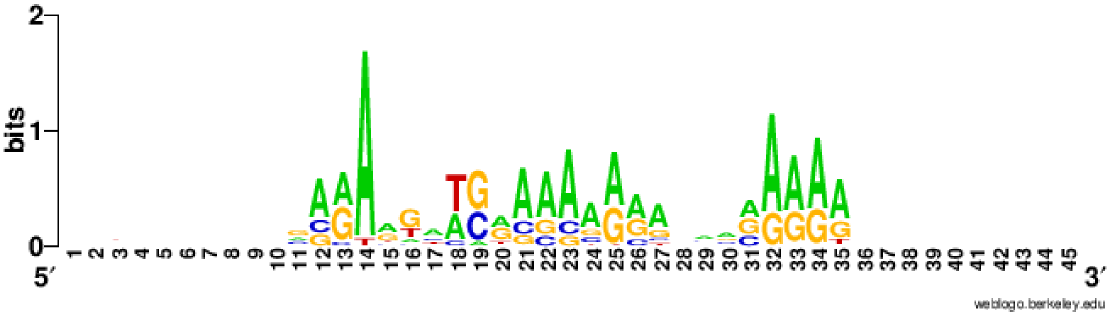

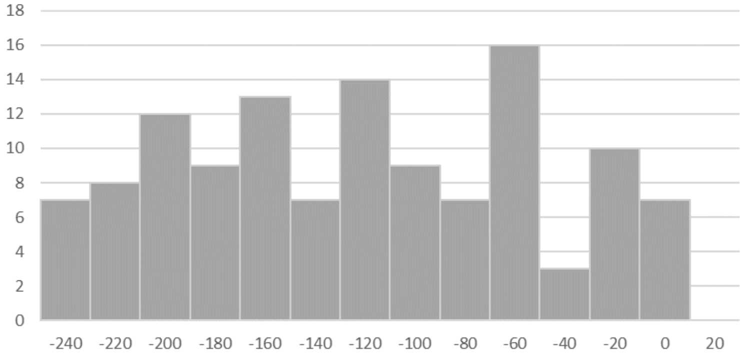

Motif #6 (129 sites)

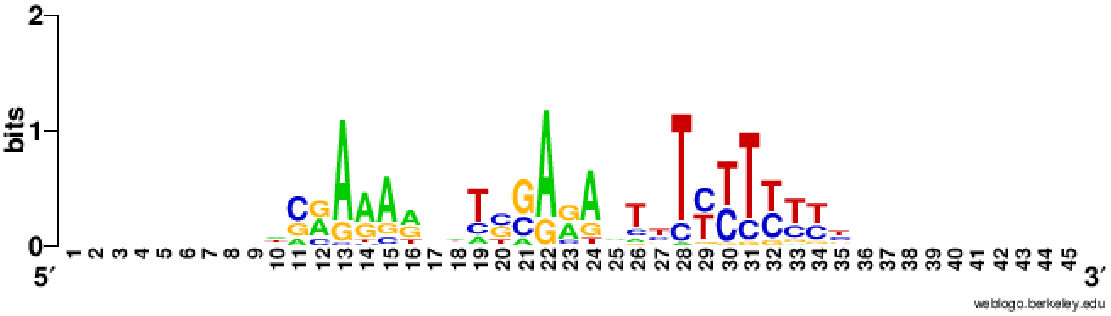

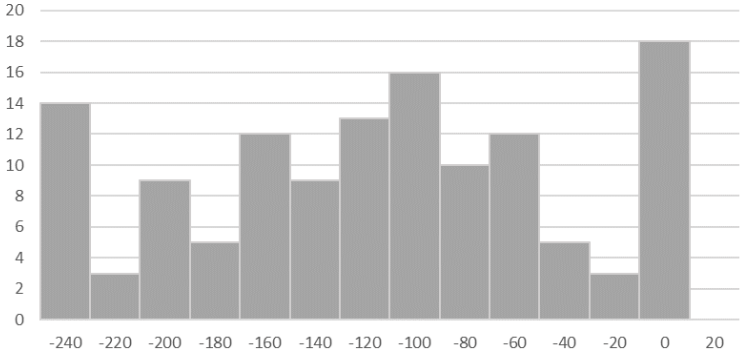

LCIVAC

211 fragments

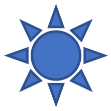

Motif #1 (76 sites)

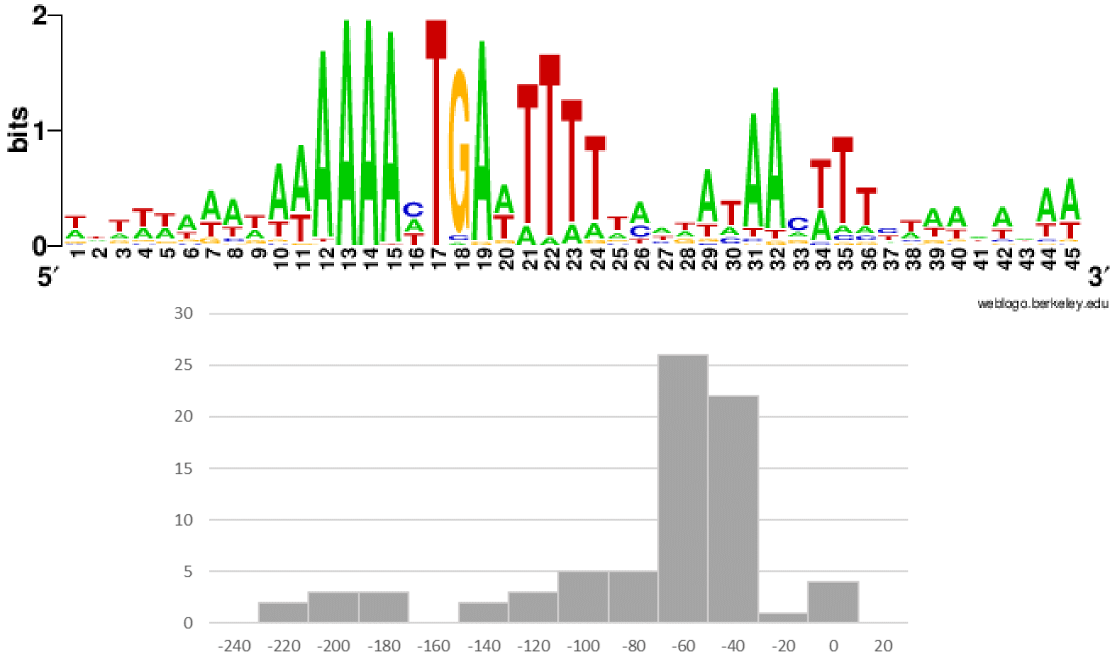

Motif #3 (55 sites)

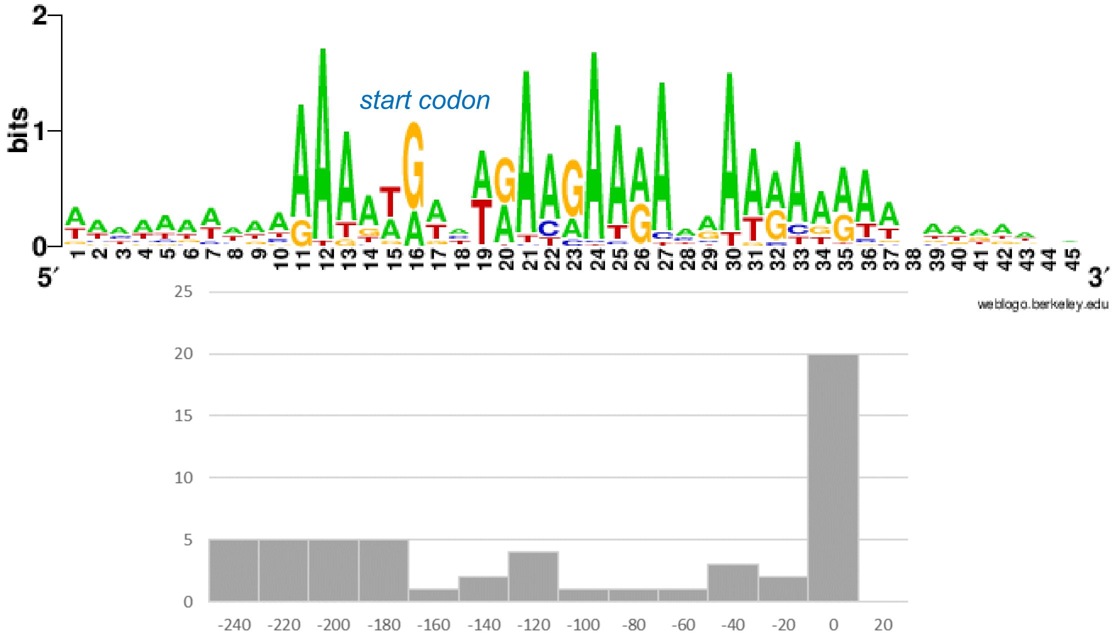

Supplement: TEXT S8 [file mBio.02497-18-s0008.pdf]
